# Vertebrate Alpha2,8-Sialyltransferases (ST8Sia): A Teleost Perspective

**DOI:** 10.3390/ijms21020513

**Published:** 2020-01-14

**Authors:** Marzia Tindara Venuto, Mathieu Decloquement, Joan Martorell Ribera, Maxence Noel, Alexander Rebl, Virginie Cogez, Daniel Petit, Sebastian Peter Galuska, Anne Harduin-Lepers

**Affiliations:** 1 Institute of Reproductive Biology, Leibniz Institute for Farm Animal Biology (FBN), Wilhelm-Stahl-Allee 2, 18196 Dummerstorf, Germany; venuto@fbn-dummerstorf.de (M.T.V.); galuska.Sebastian@fbn-dummerstorf.de (S.P.G.); 2 Université de Lille, CNRS, UMR 8576-UGSF-Unité de Glycobiologie Structurale et Fonctionnelle, F-59000 Lille, France; mathieu.decloquement.etu@univ-lille.fr (M.D.); maxence.noel@univ-lille.fr (M.N.); virginie.cogez@univ-lille.fr (V.C.); 3 Institute of Genome Biology Leibniz Institute for Farm Animal Biology (FBN), Wilhelm-Stahl-Allee 2, 18196 Dummerstorf, Germany; martorell-ribera@fbn-dummerstorf.de (J.M.R.); rebl@fbn-dummerstorf.de (A.R.); 4 Glycosylation et Différenciation Cellulaire, EA 7500, Laboratoire PEIRENE, Université de Limoges, 123 Avenue Albert Thomas, 87060 Limoges CEDEX, France; daniel.petit@unilim.fr

**Keywords:** molecular phylogeny, α2,8-sialyltransferases, polySia motifs, evolution, ST8Sia, functional genomics

## Abstract

We identified and analyzed α2,8-sialyltransferases sequences among 71 ray-finned fish species to provide the first comprehensive view of the Teleost ST8Sia repertoire. This repertoire expanded over the course of Vertebrate evolution and was primarily shaped by the whole genome events R1 and R2, but not by the Teleost-specific R3. We showed that duplicated *st8sia* genes like *st8sia7*, *st8sia8*, and *st8sia9* have disappeared from Tetrapods, whereas their orthologues were maintained in Teleosts. Furthermore, several fish species specific genome duplications account for the presence of multiple poly-α2,8-sialyltransferases in the Salmonidae (ST8Sia II-r1 and ST8Sia II-r2) and in *Cyprinus* *carpio* (ST8Sia IV-r1 and ST8Sia IV-r2). Paralogy and synteny analyses provided more relevant and solid information that enabled us to reconstruct the evolutionary history of *st8sia* genes in fish genomes. Our data also indicated that, while the mammalian ST8Sia family is comprised of six subfamilies forming di-, oligo-, or polymers of α2,8-linked sialic acids, the fish ST8Sia family, amounting to a total of 10 genes in fish, appears to be much more diverse and shows a patchy distribution among fish species. A focus on Salmonidae showed that (i) the two copies of *st8sia2* genes have overall contrasted tissue-specific expressions, with noticeable changes when compared with human co-orthologue, and that (ii) *st8sia4* is weakly expressed. Multiple sequence alignments enabled us to detect changes in the conserved polysialyltransferase domain (PSTD) of the fish sequences that could account for variable enzymatic activities. These data provide the bases for further functional studies using recombinant enzymes.

## 1. Introduction

Glycoproteins and glycolipids can be modified with numerous different glycans during their transit to the cell surface. Here, these glycoconjugates form a dense meshwork, the glycocalyx, influencing several essential processes, such as adhesion and migration mechanisms in addition to cell signaling. Intriguingly, all living cells are surrounded by such a sugar-coat, which demonstrates the importance of glycans for all living organisms [1]. However, glycoconjugates are not only found on the cellular membranes, but also on released extracellular vesicles and soluble glycoconjugates; likewise, various physiological and pathological can be targeted by their released forms. Several different monosaccharides are utilized for the formation of glycans. Nevertheless, a very special position among the building blocks of glycans takes the family of sialic acids [2,3]. These α-keto acids consist of a nine-carbon backbone with a carboxylic acid group at C1 and a ketone group at C2 [4]. Remarkably, more than 50 derivatives are known in nature. Besides N-acetylneuraminic acid (Neu5Ac), N-glycolylneuraminic acid (Neu5Gc) is the most common sialic acid and the hydroxyl groups of both can be additionally substituted, for example, by acetylation. The same applies for a further common sialic acid, which is mainly used in lower vertebrates, deaminated neuraminic acid (KDN, 2-keto-3-deoxy-D-glycero-D-galacto-nononic acid) [5]. All three of these sialic acids are frequently added by α2,3- and α2,6-sialyltransferases (ST3Gal, ST6Gal and ST6GalNAc) to nascent glycans. However, in contrast to other commonly utilized monosaccharides of glycans, an attached sialic acid residue can only be used to add another sialic acid residue, which explains their outermost position on sialylated glycans. The elongation at position C8 of α2,3- or α2,6-linked sialic acid residues is catalyzed by sialyltransferases belonging to the group of α2,8-sialyltransferases (ST8Sia) and long polymers of sialic acids can be enzymatically synthesized in this way [6,7,8].

All those animal sialyltransferases (α2,3-, α2,6- α2,8-sialyltransferases) belong to the CAZy glycosyltransferase family GT29, which indicates their common modular organization (GT-A-like fold) and their common ancestral origin [8,9]. These protein sequences are characterized by the presence of four consensus motifs called sialylmotifs (L (Large), S (Small), III, and VS (Very Small)) involved in 3D structure maintenance, substrate binding, and catalysis [10,11]. The sialylmotifs are very useful for in silico identification of sialyltransferases-related sequences [12]. On the basis of their sugar acceptor specificity and glycosidic linkage formed, GT29 is subdivided into four families ST3Gal, ST6Gal, ST6GalNAc, and ST8Sia in vertebrates [7,13], each of which is characterized by family motifs likely involved in linkage specificity [14,15,16]. The biosynthesis of α2,8-sialylated molecules is an ancient pathway achieved by the ST8Sia, a group of enzymes that emerge in the first eukaryotes [8] and expanded very early in animal evolution [14]. Up to now, the ST8Sia enzymes have been studied and characterized in mammalian tissues and primarily in the adult brain. The human and mouse genomes show six ST8Sia subfamilies: ST8Sia I, ST8Sia V, and ST8Sia VI are mono-α2,8-sialyltransferases and constitute a first group of ST8Sia enzymes involved in di-sialylation of glycoconjugates, while ST8Sia III in addition to ST8Sia II and ST8Sia IV form a second group of oligo- and poly-α2,8-sialyltransferases implicated in the polysialylation of glycoproteins [15].

Interestingly, our recent studies pointed to the fact that the *st8sia* gene family appears to be much larger in teleost fish genomes [14,17]. The emergence of several novel vertebrate mono-α2,8-sialyltransferases subfamilies like ST8Sia VII and ST8Sia VIII was described in this first group of ST8Sia and their enzymatic specificities remain to be determined. These mono-α2,8-sialyltransferase genes have arisen as a consequence of whole genome duplications (WGDs, R1 and R2) at the base of vertebrates and were maintained in fish, whereas some others such as *st8sia6*, maintained in Tetrapods, have disappeared in fish [17,18]. In the second ST8Sia group, the enzymes responsible for the biosynthesis of sialic acid polymers, the poly-α2,8-sialyltransferases ST8Sia II and ST8Sia IV and the oligo- α2,8-sialyltransferase ST8Sia III, have been cloned and characterized from mammalian tissues, essentially the brain, where they act on α2,3-sialylated N-glycans of the neural cell adhesion molecule (NCAM), leading to an increased neuronal plasticity in embryos [19,20,21,22,23,24,25,26]. From a structural point of view, the poly-α2,8-sialyltransferases share a high degree of similarity in their sequence and structure [27,28,29] and are characterized by two additional sequence motifs, termed the polysialyltransferase domain (PSTD), of 32 amino acids located upstream of the sialylmotif S, and the polybasic region (PBR), of 35 amino acids located in the stem region of the enzymes involved in protein-specific polysialylation [30,31]. The oligo-α2,8-sialyltransferases ST8Sia III also show additional broadly conserved motifs with respect to ST8Sia II and ST8Sia IV (motifs III-1 and III-2) [14] with potential implication in the oligosialylation activity [32]. Their fish orthologues have been identified, cloned, and characterized in zebrafish (*Danio rerio*) in addition to rainbow trout (*Oncorhynchus mykiss*) [18,33,34].

Our previous phylogenetic studies also identified novel α2,8-sialyltransferases-related sequences like the ST8Sia III-related (ST8Sia III-r) found in a few fish orders like Perciformes, Tetraodontiformes, and Beloniformes, whereas the ST8Sia IV disappeared from the Neognathi fish [14]. It has long been appreciated that gene-, segmental-, and genome duplication, as well as gene loss events, have played important role in evolution, providing new genetic materials, which may facilitate new adaptation for the organism [35,36].

In this study, we used a BLAST strategy to identify over 700 ST8Sia-related sequences from ray-finned fish genomes and performed phylogenetic analyses and sequences alignments to reevaluate their evolutionary relationships and fate, focusing on those responsible for polysialic acid (polySia) biosynthesis with implications for the evolution of nervous system, immunological system, and cell–cell interactions. Our findings point to a particular distribution of ST8Sia in fish, revealing novel *st8sia* gene members and further suggesting their functional divergence in vertebrates.

## 2. Results and Discussion

### 2.1. In Silico Identification and Phylogenetic Reconstruction of ST8Sia Sequences

**Figure 1:**
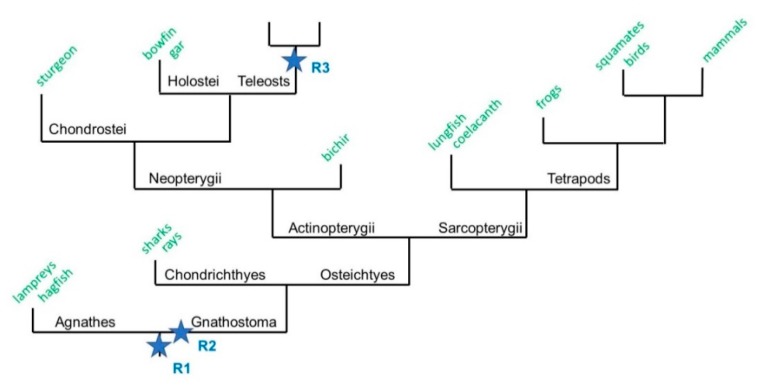
A schematic phylogenetic tree of vertebrate evolution. A simplified phylogenetic tree depicting the evolution of the jawed vertebrates Gnathostomes after the two rounds of whole genome duplication (WGD, R1 and R2). It is hypothesized here that WGD-R2 occurred after the Gnathostomes-Agnathes (jawless vertebrates) split. The Gnathostomes branch is divided into two categories: the cartilaginous fish Chondrychtyes (sharks and rays) and the bony fish Osteichthyes. The Osteichthyes are split into the lobe-finned fish Sarcopterygii that contain Tetrapods, and the ray-finned fish Actinopterygii that contain Neopterygii (Chondrostei, Holostei, and Teleosts).

To investigate *st8sia* genes’ expansion and distribution in vertebrates, we performed public database screenings in the National Center for Biotechnology Information (NCBI), ENSEMBL, and Phylofish databases [37] using a BLAST strategy [38]. The obtained results led to the identification of more than 700 ST8Sia-related sequences (Supplemental Data 1) in chordate genomes, including 71 ray-finned fish genomes (68 Teleosts genomes). Putative ST8Sia sequences with significant similarity to the known human ST8Sia based on the presence of the sialylmotifs L, S, III and VS found in all GT29 sialyltransferases, and of family motifs characteristic for the ST8Sia family were selected, and multiple sequence alignments were performed to select the complete open reading frame. The orthologues of ST8Sia I and ST8Sia V involved in gangliosides biosynthesis are identified in all the investigated genomes, suggesting a high conservation of the gangliosides biosynthetic pathways in vertebrates (Supplemental Table S1). Similarly, the ST8Sia III and the recently described fish ST8Sia VIII [17] could be found in all the Actinopterygii (ray-finned fishes) genomes (Figure 1; Supplemental Table S1). Intriguingly, multiple copies of *st8sia*-related gene sequences were identified in Teleost genomes and their number varied considerably from one fish order or species to another. For example, there are 6 *st8sia*-related genes in the medaka (*Oryzias latipes*), 8 in the clownfish (*Amphiprion oscellaris*) and the common carp (*C. carpio*), and up to 10 in the rainbow trout. Indeed, multiple copies of ST8Sia VIII (>3) were found in Perciformes, Cichliiformes, and Cyprinodontiformes; that is, two copies of the ST8Sia VII in Cypriniformes, two copies of the ST8Sia II in Salmoniformes, and two copies of the ST8Sia IV were found in the Cypriniforme *C. carpio* (Supplemental Table S1). In addition, some other *st8sia* genes could not be found like ST8Sia VI in Teleosts and Chondrostei [17]; ST8Sia IV in Neoteleostei genomes [14]; or ST8Sia II in Esociformes, Siluriformes, or Gymnotiformes (except *Electrophorus electricus*) genomes (Table 1). This resulted in a particular distribution of ST8Sia observed in the Actinopterygii compared with the Sarcopterygii (lobbed-finned fishes and Tetrapods) and Chondrichtyes (sharks) (Figure 1; Supplemental Table S1), which might have facilitated the acquisition of evolutionary innovations during vertebrate evolution [35]. These observations prompted us to re-examine the genetic events, which have shaped α2,8-sialylation in Teleosts.

**Figure 2:**
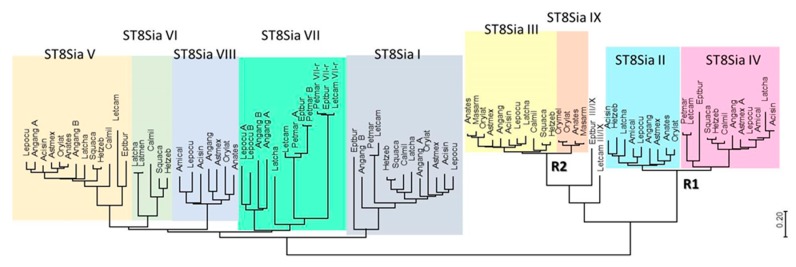
Minimum evolution phylogenetic tree of 89 chordates ST8Sia. The evolutionary history of 89 ST8Sia (see names and sequences in Supplemental Data 1) was inferred using the minimum evolution (ME) method. The optimal tree drawn to scale with the sum of branch length = 16.02931149 is shown. The evolutionary distances were computed using the JTT (Jones-Taylor-Thornton) matrix-based method and the rate variation among sites was modeled with a gamma distribution (shape parameter = 5). The ME tree was searched using the close-neighbor-interchange (CNI) algorithm at a search level of 1. The neighbor-joining algorithm [39] was used to generate the initial tree. The analysis involved 89 amino acid sequences and all positions with less than 95% site coverage were eliminated. A total of 226 positions were in the final dataset (see multiple sequence alignments in Supplemental Data 2). Evolutionary analyses were conducted in MEGA7.0 [40]. The nine Vertebrate subfamilies of ST8Sia (ST8Sia I to ST8Sia IX) are indicated by various colors.

To determine whether the expansion of *st8sia* genes observed in Actinopterygii could be associated to WGD or smaller scale duplication events, we took advantage of the improved genome sequencing of several critical species for basal Vertebrates as Agnathans (Lampreys and Hagfish) and for Actinopterygii as Chondrostei (Sturgeons) and Holostei (Gars and Bowfin) (Figure 1). A simplified dataset was constructed including sequences of Agnathans (*Lethenteron camtschaticum*, *Petromyzon marinus*, *Eptatretus burgii*), Chondrichthyans (*Callorhinchus milii*, *Squalus acanthias*, and *Heterodontus zebra*), basal Actinopterygians (*Acipenser sinensis*, *Amia calva*, and *Lepisosteus oculatus*) and basal Teleosteans such as the Elopomorphs *Anguilla anguilla* and *Mastacembelus armatus*, in addition to two Teleosts, the Beloniforme *O. latipes* (medaka) and the Characiforme *Astyanax mexicanus* (cave fish). The potential orthology of the selected sequences was assessed through the construction of phylogenetic trees (Figure 2). The topology of these trees indicated two major phylogenetic groups of mono-α2,8-sialyltransferases on one hand, and oligo- and poly-α2,8-sialyltransferases on the other, as previously described [7,14].

In the mono-α2,8-sialyltransferases group, a series of Agnathan sequences are found at the base of each of ST8Sia I and ST8Sia V. The results corroborate previous findings suggesting the emergence of these two subfamilies around 596 and 563 million years ago (MYA), well before vertebrates emergence and prior WGD R1 and R2 [14]. Consistent with our previous data [17], we identified *st8sia7* genes in the jawless vertebrates *Lethenteron camtschaticum*, *Petromyzon marinum*, and *Eptatretus burgeri* genomes. Thus, these genes might have arisen from the ancestral *st8sia6/7/8* gene after the first WGD R1 event (~552 MYA), although timing of these events with respect to the divergence of agnathans is still a matter of debate [41,42]. Interestingly, Agnathans possess two copies of this later enzyme, named ST8Sia VII and ST8Sia VII-r in Figure 2, likely resulting from species specific large-scale gene duplication events. Similarly, in Teleosts, the eel *A. anguilla* (Elopomorphes, see the work of [43]) also harbors two copies of ST8Sia VII, ST8Sia I, and ST8Sia V enzymes (Figure 2). This observation is in favor of a large-scale genome duplication event different from the Teleost specific third round of WGD R3 (TGD) [44,45], which may have taken place in a common ancestor of freshwater eels sometime after the split of Elopomorpha and Osteoglossomorpha [46]. The ST8Sia VI and ST8Sia VIII subfamilies likely have arisen from the second WGD at the base of Vertebrates; the first one was maintained in Sarcopterygii and disappeared in Actinopterygii, and vice versa for ST8Sia VIII [17]. The many gene copies of *st8sia7* and *st8sia8* identified in Teleosts genomes (Supplemental Table S1) are likely the result of single gene duplication events because they were identified on the same piece of chromosome (data not shown), and were thus noted with -A, -B, or -C extension. However, it is difficult to infer the origin of these segmental duplications as they have occurred in many, but not all terminal branches of clades.

The second branch encompasses both oligo- and poly-α2,8-sialyltransferases. Regarding poly-α2,8-sialyltransferases, the Agnathan sequences were attributable only to ST8Sia IV, indicative of a divergence between ST8Sia II and ST8Sia IV dating back to WGD-R1 (Figure 2) [14] followed by *st8sia2* gene loss in Agnathans. In contrast, the Agnathan sequences of oligo-α2,8-sialyltransferases are at the base of the ST8Sia III and ST8Sia III-r subfamilies, while there are orthologues to the ST8Sia III from sharks to Tetrapod lineages, suggesting a genome duplication event linked to WGD-R2 consistent with previous dating around 474 MYA [14]. Despite the fact that the ST8Sia III-r sequences appear to be restricted to Teleosteans, including Elopomorphes, and are lost in Chondrichthyans and Tetrapods lineages, they were not issued from the Teleost specific WGD, and thus were renamed ST8Sia IX according to the previously described nomenclature [12].

### 2.2. Identification and Phylogenetic Analysis of the Fish St8sia Genes (st8sia2, st8sia4, st8sia3, and st8sia9)

Interestingly, in the oligo- and poly-α2,8-sialyltransferases group, the ST8Sia II and ST8Sia IV appeared to be duplicated or lost in several Teleost lineages after divergence of Actinopterygii from Sarcopterygii [47,48], whereas the ST8Sia III was found in all the Actinopterygii. In the basal Elopomorphes and Osteoglossiformes branches, the four *st8sia* genes (*st8sia2*, *st8sia3*, *st8sia4*, and *st8sia9*) could be identified. The results indicate that these genes already existed in the common ancestor of the 68 Teleost fishes examined. All Otocephalan lineages lack the *st8sia9* gene and the Siluriformes lack both the *st8sia9* and *st8sia2* genes. Consequently, the *st8sia9* gene was lost shortly after Otocephala emergence around 176.2 MYA and the *st8sia2* gene was lost more recently (~82.6 MYA) during siluriformes evolution [49]. As previously observed, all Neoteleostei fish lack the *st8sia4* gene [14], which was lost at the basis of Neoteleostei lineage. Finally, the Esociformes lack the *st8sia2* gene only (Table 1). Furthermore, two ST8Sia II-related sequences were identified in all the investigated Salmoniformes (*Oncorhynchus*, *Coregonus*, *Salmo*, *Salvelinus*, and *Thymallus*) and two ST8Sia IV-related sequences were identified only in the Cypriniformes *C. carpio* and *Sinocyclocheilus anhuiensis* (Supplemental Table S1). We took advantage of the improved genome and transcriptome sequencing of several fish [37,50], selected several representative Salmoniformes and Cypriniformes ST8Sia sequences, and constructed phylogenetic trees (Supplemental Figure S1). The topology of these trees indicated that the later duplications of *st8sia* genes were not associated to the Teleost specific genome duplication (TGD, WGD R3), but rather to more recent lineage-specific genome duplication events described in Salmonidae (SGD) lineage [51] and in *C. carpio* species [52].

### 2.3. Synteny and Paralogy Analyses of the st8sia2, st8sia4, st8sia3, and st8sia9 Gene Loci

**Figure 3:**
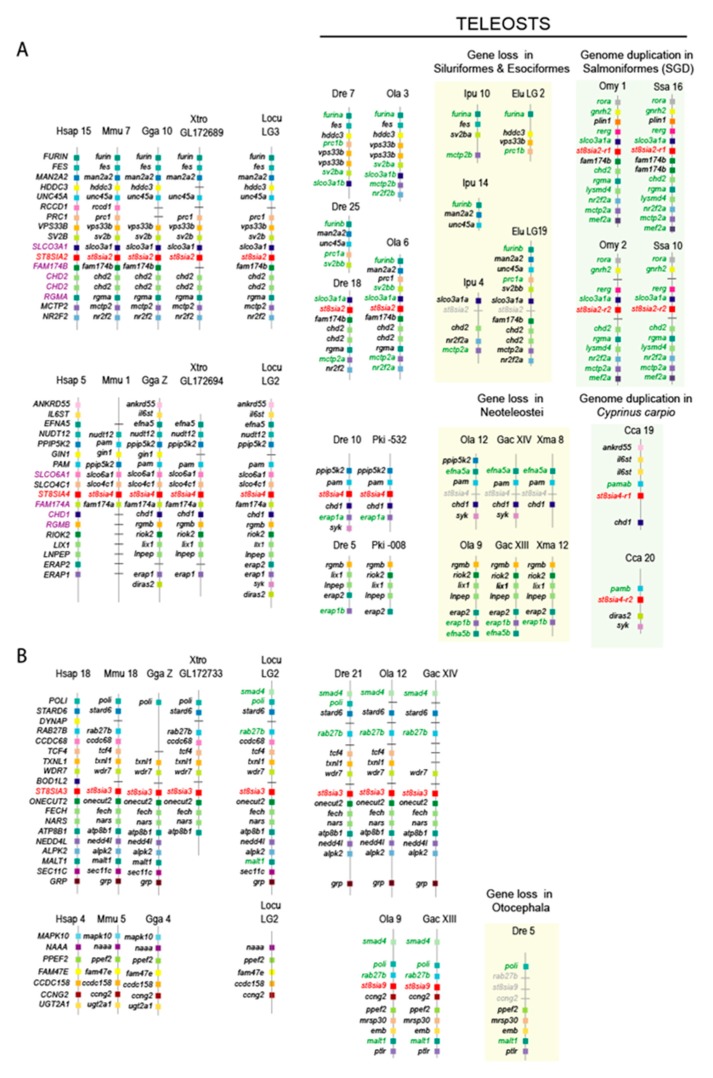
Syntenic relationships of the oligo- and poly-α2,8-sialyltransferases gene loci in vertebrates. Chromosomal locations of the *st8sia* genes and neighboring gene loci were determined in the human (*Homo sapiens*, Hsa), the mouse (*Mus musculus* (Mmu), the chicken (*Gallus gallus*, Gga), the spotted gar (*L. oculatus*, Locu), the western clawed frog (*Xenopus tropicalis*, Xtro), the zebrafish (*D. rerio*, Dre), the Japanese medaka (*O. latipes*, Ola), the channel catfish (*I. punctatus*, Ipu), the northern pike (*E*. *lucius*, Elu), the rainbow trout (*O. mykiss*, Omy), the Atlantic salmon (*Salmo salar*, Ssa), the African weakly electric fish (*Paramormyrops kingsleyae*, Pki), the three-spined stickleback (*G. aculeatus*, Gac), the southern platyfish (*X. maculatus*, Xma), and the European carp (*C. carpio*, Cca). Information from the National Center for Biotechnology Information (NCBI) and ENSEMBL release 97 was used to identify putative orthologues, which were visualized using the Genomicus 97.01 [53]. Paralogous genes in the fish genomes are indicated in green and in purple in the human genome. The *st8sia* genes are indicated in red or in grey when lost. (**A**) Syntenic relationships of the *st8sia2* and *st8sia4* gene loci in vertebrates. (**B**) Syntenic relationships of the *st8sia3* and *st8sia9* gene loci in vertebrates.

To explain the gain or loss of ST8Sia subfamilies, we further analyzed the evolutionary relationships between these *st8sia* genes. The kind of event that created duplication was characterized by analyzing the conserved synteny between ST8Sia paralogues. It was expected that the *st8sia* genes created by a WGD would be far apart on different chromosomes in one genome, but surrounded by similar genes in each of the duplicated regions (i.e., paralogons). Significant Tetrapod paralogons containing *st8sia2* and *st8sia4* genes were found and a well conserved synteny could be established for *st8sia2* and *st8sia4* gene loci in Tetrapods (i.e., human, mouse, chicken, and xenopus) genomes (Figure 3A) as previously described [14]. However, in the fish genomes, as the *st8sia2* gene was absent in Esociformes and Siluriformes, we considered the neighboring *furin*, *fes*, *sv2b*, *fam147b*, *mctp2*, and *chd2* genes around *st8sia2* on the medaka chromosome 6 to retrieve the synteny on *Esox lucius* LG19 and on *Ictularus punctatus* chromosome 4. Similarly, *ppip5k2*, *pam*, *chd1 erap1a*, and *syk* genes conserved around the *st8sia4* locus were used to retrieve the synteny on *O. latipes* chromosome 12, *Gasterosteus oculatus* chromosome XIV, and *Xiphophorus maculatus* chromosome 8. Interestingly, paralogues of these genes could be identified on other chromosomes in the various fish genomes indicative of an ancient Teleost specific WGD (TGD) followed by intense gene rearrangements. This further suggests that the *st8sia* genes have undergone the TGD and the duplicated *st8sia* genes were rapidly lost during Teleost evolution. In the Salmoniformes, a highly conserved synteny was found around the two *st8sia2-r* gene loci corresponding to one ohnologous region in the spotted gar (*L. oculatus*), likely resulting from the fourth round of WGD (SGD) that took place more recently in the Salmoniforme genomes [51]. The two *st8sia4-r* genes were localized on two distinct chromosomes in *C. carpio* genome, supporting the hypothesis of a more recent species-specific genome duplication event in *C. carpio* [52] in spite of a weak synteny conservation (Figure 3A).

The synteny around the *st8sia3* gene locus including *wdr7*, *onecut2*, and *fech* genes is highly conserved in vertebrate lineages from fish to mammals (Figure 3B). Synteny around *st8sia9* locus is less conserved and is limited to a smaller syntenic block with *ccng2* and *ppef2* genes, which is reminiscent of ancient WGD followed by intrachromosomal rearrangement in the ancestral fish genome.

**Figure 4:**
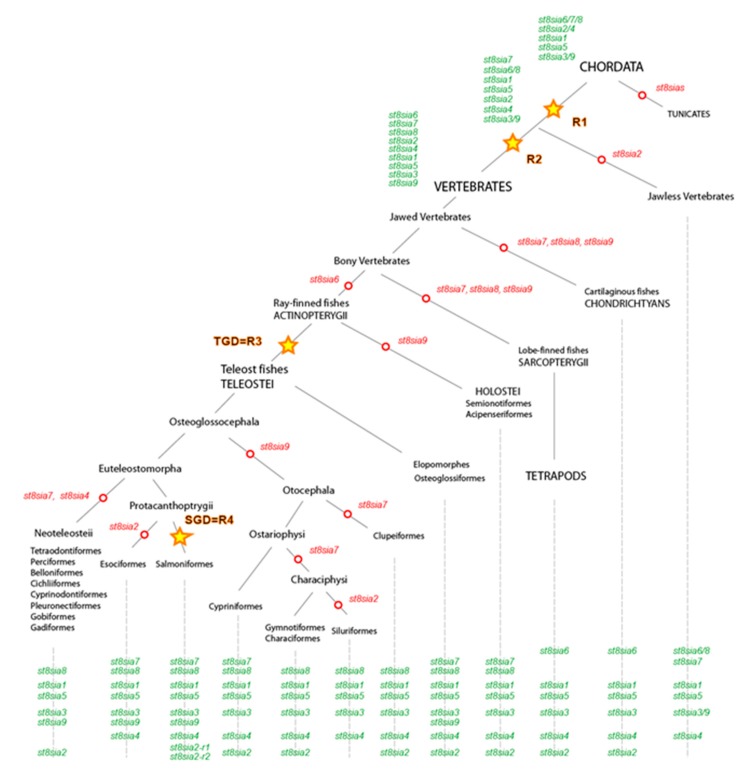
Schematic representation of the ST8Sia family evolution in the ray-finned fishes. This model for the evolution of *st8sia* genes is based on the evidence from protein sequence phylogeny, conserved synteny of genomic *st8sia* loci in vertebrate species and their paralogous relationships in fish genomes. The model takes into account the evolution of five ancestral groups of ST8Sia (*st8sia6/7/8*, *st8sia2/4*, *st8sia1*, *st8sia5*, and *st8sia3/9*) indicated in green and present in the ancestor of Chordates that predate the WGD R1 and WGD R2. Open red circles depict gene losses on the phylogenetic tree and yellow stars correspond to the WGDs R1, R2, R3 (teleost specific duplication, TGD), and R4 (salmonids specific duplication, SGD).

Altogether, our phylogenetic analyses enabled us to refine the evolutionary history of the fish ST8Sia and to propose a model of their evolution illustrated in Figure 4, which agrees with the fish phylogenetic tree of life [54]. It is interesting to note that, while Braasch and Postlethwait (2012) determined duplicated gene retention rates of 12–24% after the TGD 320 MYA [55], we observed no remaining *st8sia* gene copy from this event and no modification on the fish ST8Sia repertoire. However, more recent polyploidization events were recorded in several families (Salmonidae, 80 MYA), genera (Anguilla) or species (*C. carpio*, 8 MYA), which impacted the overall poly-α2,8-sialyltransferases repertoire. In Salmonidae, we described only two remaining *st8sia2* duplicates after the Ss4R among the eight ancestral *st8sia* genes (12% duplicate retention), while Lien et al. (2016) revealed a global retention rate around 55% [56]. In the carp *C. carpio*, two *st8sia4* genes were retained as duplicates among the seven *st8sia* genes (14% duplicate retention), while Li et al. (2015) calculated a global value of 92% [57]. Furthermore, these studies highlighted the fact that the retained genes after tetraploidization were specifically involved in signal transduction, protein complex formation, and immune system, which prompted us to focus on the functional divergence of these poly-α2,8-sialyltransferase duplicated genes (neofunctionalization) and on their expression divergence (subfunctionalization).

### 2.4. Molecular Evolution of the Poly-α2,8-Sialyltransferases

A remarkable difference between α2,8-linked polySia chains found in mammals and salmonid fish seems to be the structural diversity of polySia in fish [58,59,60]. Whereas in mammals, homopolymers of Neu5Ac residues are typically formed [61], in rainbow trout eggs, polymers can consist of Neu5Ac, Neu5Gc, and KDN in addition to their O-acetylated forms [62]. One explanation could be a better accessibility to different sialic acids in fish, because, in transgenic mice—showing a Neu5Gc overexpression in brain—besides Neu5Ac, Neu5Gc also seems to be utilized to build polySia [63].

**Figure 5:**
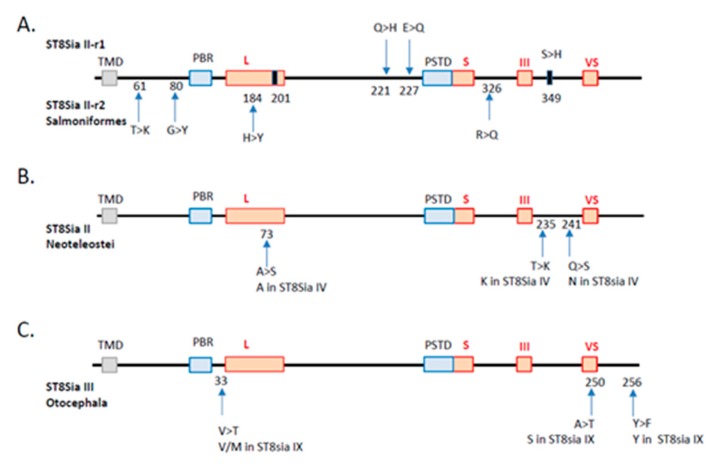
Substitution rate analysis of the impact of *st8sia* gene duplications and losses. The sialylmotifs are indicated by red boxes and the transmembrane domain by a grey box. (**A**) Duplication of *st8sia2* genes in Salmoniformes. The substitutions observed in ST8Sia II-r1 and ST8Sia II-r2 are indicated by an arrow above and below, respectively. The position of the substitutions corresponds to the alignment in Supplemental Data 2. The black rectangles correspond to convergent mutations retrieved in both sequences. In T > K, for example, T is the ancestral state and K is the derived one. (**B**) Impact of *st8sia4* gene loss in Neoteleostei on the remaining fish ST8Sia II sequences. The code for substitution is the same as in A. The corresponding amino acid present in the paralogue ST8Sia IV sequence is given below. (**C**) Impact of *st8sia9* gene loss in Otocephala on the remaining fish ST8Sia III sequences (same abbreviations as in B).

Another explanation might be the occurrence of structural changes of the protein backbone during the evolution of the polysialyltransferases. We thus investigated the potential consequences of specific-lineages’ *st8sia* gene loss and duplication on the functional fate of duplicates, an issue that is still poorly understood [64,65]. Substitution rate analysis of the duplicated *st8sia2* genes maintained in Salmoniformes genome after the SDG event indicated four amino acid substitutions in the ST8Sia II-r2 coding sequences compared with ST8Sia II-r1 and the rest of Teleost ST8Sia II sequences, while there were only two substitutions in the ST8Sia II-r1 sequence. Of particular note, among the four substitutions found in ST8Sia II-r2, the H → Y is recorded in sialylmotif L, and the R → Q between sialylmotifs S and III, whereas the two substitutions in the ST8Sia II-r1 sequence are located nearby the PSTD motif (Figure 5A). In addition, two convergent substitutions leading to the same amino acid were identified near the end of sialylmotif L (i.e., acquisition of a G from a Q) and beyond the sialylmotif III (i.e., acquisition of an H from an S), respectively. These drastic modifications in amino acid properties in functionally important locations in the catalytic domain of these salmonid ST8Sia II let us suggest profound changes in both ST8Sia II functions (i.e., neofunctionalization). Likewise, we examined the impact of *st8sia4* loss on the remaining *st8sia2* gene in Neoteleostei using parsimony analysis. We found two substitutions, A → S and Q → S, located in the sialylmotif L and between the sialylmotifs III and VS that of Neosteleostei ST8Sia II, respectively (Figure 5B). Interestingly, we also found a convergent T → K substitution located between the sialylmotifs III and VS that of Neosteleostei ST8Sia II that restores the K amino acid characteristic of all the ST8Sia IV sequences (Figure 5B), further suggesting changes in ST8Sia II functions in Neoteleotei. No substitution could be detected in ST8Sia IV sequences after the loss of *st8sia2* gene in Esociformes and Osmeriformes. Finally, we recorded the substitutions on the ancestral sequence of ST8Sia III after ST8Sia IX loss in Otocephala. We observed three substitutions in ST8Sia III sequence: V → T near the sialylmotif L, A → T in the sialylmotif VS, and Y → F beyond (Figure 5C).

**Figure 6:**
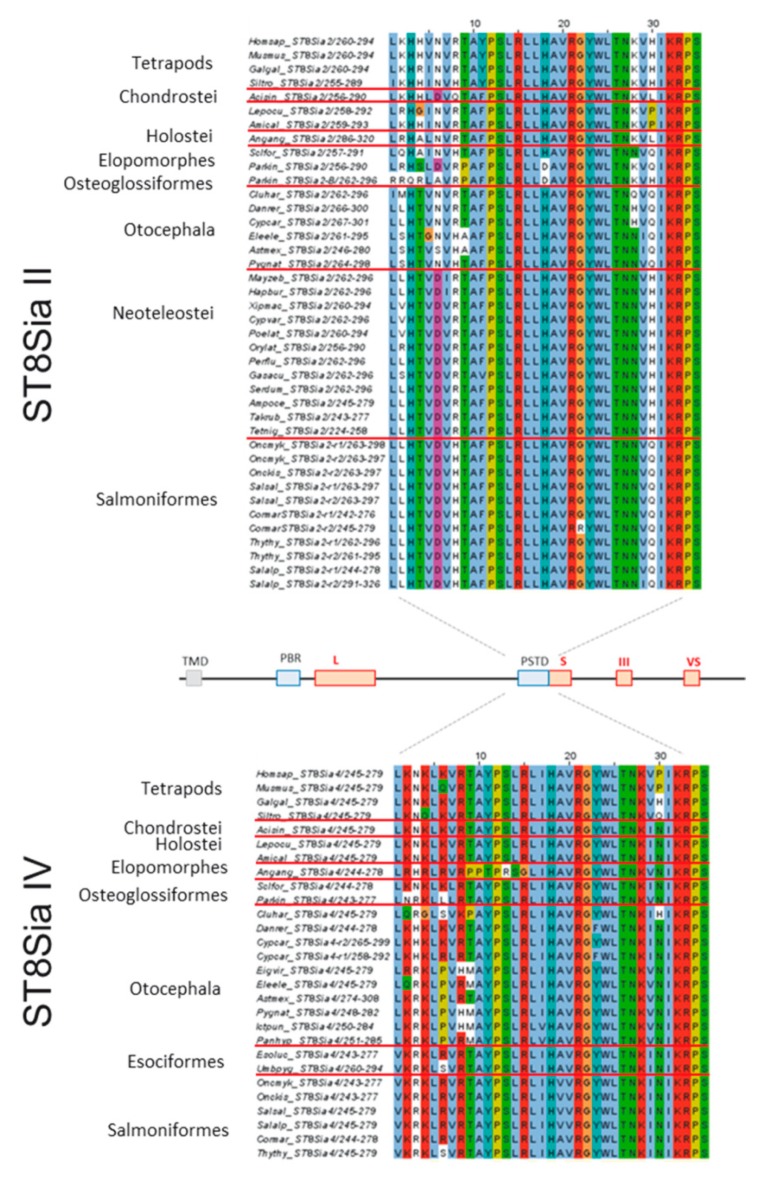
Sequence-based analysis of the polysialyltransferase domain (PSTD) in fish ST8Sia II and ST8Sia IV. Multiple sequence alignment of PSTD were performed with CLUSTAL OMEGA of EMBL-EBI by MUSCLE (3.8) edited and annotated in Jawa Alignment Jalview [67]. The used protein entries from different species are listed in Supplemental Table S1. The different colors from Clustal X scheme codes indicate the following characteristics: hydrophobic (blue), positive charge (red), negative charge (magenta), polar (green), cysteine (pink), glycine (orange), proline (yellow), aromatic (cyan), and gap (white). It should be noted that one additional amino acid was added to the N-terminus and two additional amino acids to the C-terminus of PSTD.

The most striking domain of both polysialyltransferases—ST8Sia II and ST8Sia IV—is PSTD, which is essential for the polysialylation of NCAM [31,66]. This motif contains a high number of basic amino acids and is important for substrate binding and the catalytic activity. Troy and co-workers exchanged several of these amino acids to determine their distinct impact on the enzymatic activity of human ST8Sia IV [31]. Doubled substituent mutants with an exchange of the first basic residues (declared as K2 and K4 in Figure 6) by neutral amino acids retained approximately 80% of the enzyme activity and comparable values were determined, when only K6 was replaced. Stronger effects were observed in single substituted mutants where R8, H18, K28, K32, or R33 was replaced by a neutral amino acid. All these changes reduced activity by more than 50%. Their experiments demonstrated that, in addition to the neutral amino acid I31 (mutants retained only 6% of their activity), especially the basic amino acids of PSTD were key elements for polysialylation. Most of these important amino acids of the human ST8Sia IV are also highly conserved in the fish enzyme. Changes occurred sporadically at K2, K4, K6, and R8 in individual fish species (Figure 6). On the basis of the work of Troy and co-workers [31], the R8 change may have the highest impact on the general enzyme activity, as a replacement of this amino acid reduced the activity to less than 25%. However, we observed an exchange of R8 only in three fish species including *I. punctatus*. Nevertheless, as mentioned above, other substituted amino acids may also influence the interaction with the nascent sialic acid chain, depending on the composition (Neu5Ac, Neu5Gc, KDN, and O-acetylated variations) of the polySia chain.

More consistent variations were observed when ST8Sia II sequences were compared. In addition to the mentioned K2 and H4 (K instead of H in ST8Sia IV), an exchange of a basic amino acid occurs more frequently and is often highly conserved within one family. For instance, in Salmoniformes, lysine residues at position 2 and 28 are changed with apolar amino acids and the strongly basic R8 residue is exchanged with histidine, which is only partly positively charged at neutral pH. On the basis of the studies of Nakata et al. using human ST8Sia IV, we can also assume remarkable changes in the enzymatic activity of ST8Sia II [31]. For instance, ST8Sia IV mutants with a neutral amino acid at position K28 retained less than 25% of their enzymatic activity. This is in line with studies by Kitajima and co-workers demonstrating that rainbow trout ST8Sia II isoforms show only low enzymatic activity in vitro [33]. Intriguingly, in Neoteleostei, the very important lysine at position 28 was also exchanged with a neutral amino acid. Notably, in contrast to Salmoniformes, in Neoteleostei, ST8Sia II is the only polysialyltransferase because ST8Sia IV is absent. The presence of only one polysialyltransferase in Neoteleostei, which additionally includes such a striking mutation, suggests that polysialylation significantly changed in Neoteleostei in comparison with other vertebrates.

**Figure 7:**
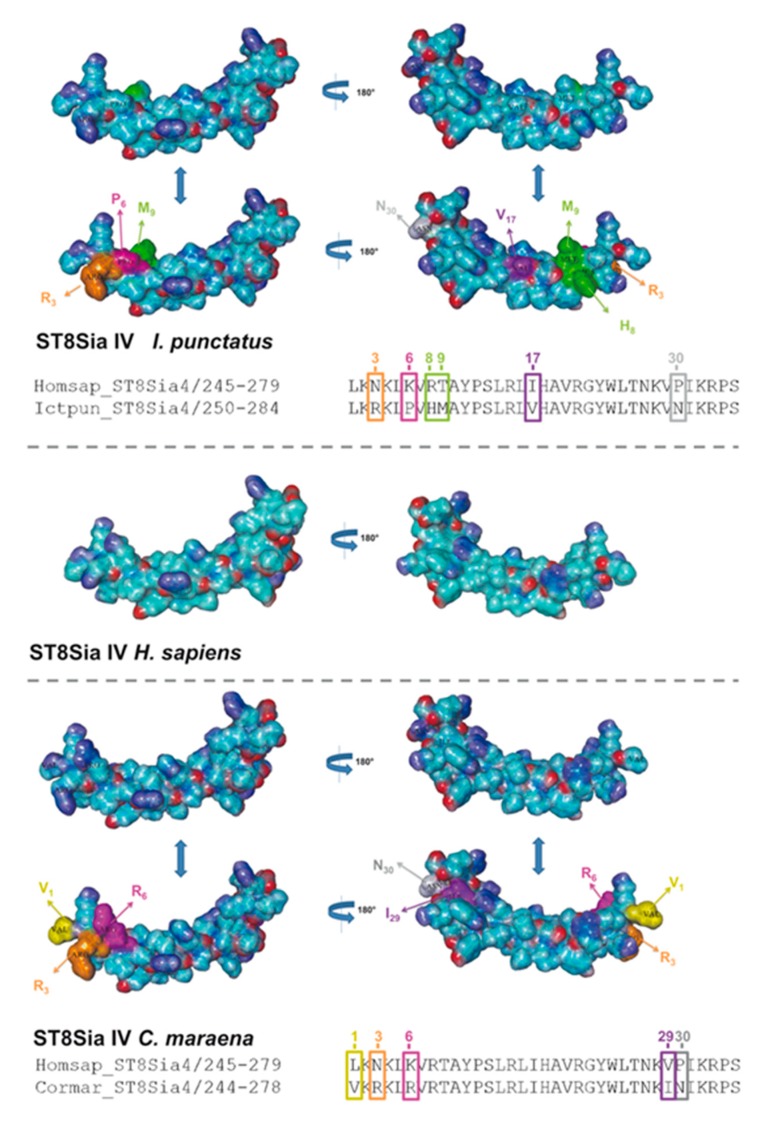
Three-dimensional (3D) structure of PSTD motifs in fish ST8Sia IV. The 3D model of human ST8Sia IV PSTD (Protein Data Bank entry 6AHZ)—electrostatic potential surfaces—is displayed in addition to the simulated structure of PSTD from I. punctatus and *C. maraena* using YASARA. The exchanged amino acids are colored in an additional version of the 3D structure to highlight the position of the exchange: N3 → R3 (orange), K6 → P6 (magenta), R8 → H8 (green), T9 → M9 (green), I17 → V17 (violet), and P30 → N30 (grey) for *I. punctatus* and L1 → V1 (yellow), N3 → R3 (orange), K6 → R6 (magenta), V29 → I29, and P30 → N30 (grey) for *C. maraena*. It should be noted that, for the determination of the 3D structure of human ST8Sia IV PSTD, a peptide was used with one additional amino acid on the N-terminus and two additional amino acids on the C-terminus of PSTD [66].

**Figure 8:**
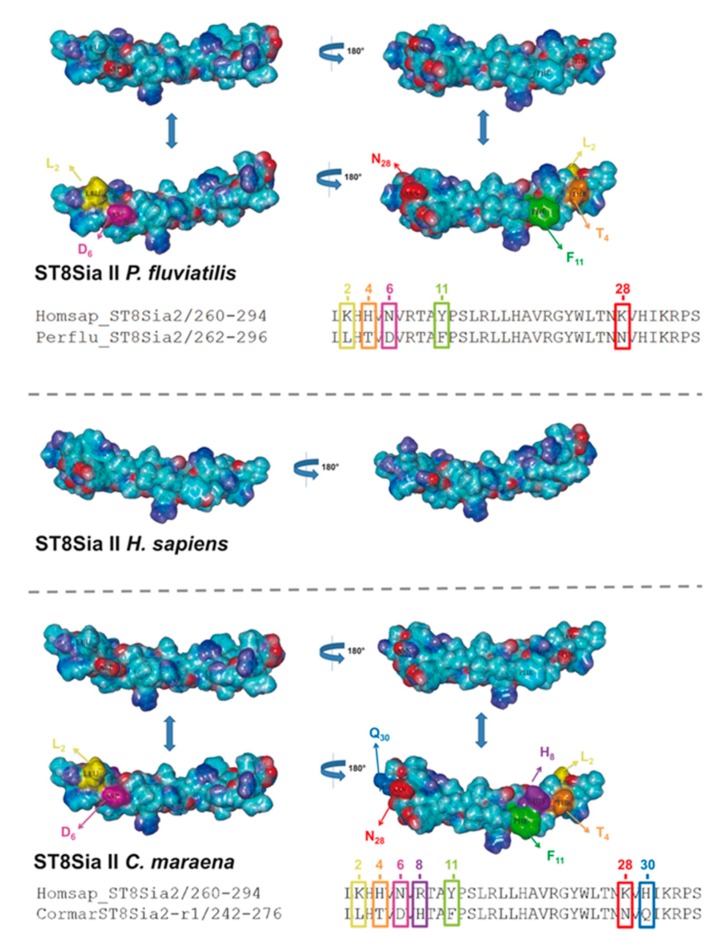
Three-dimensional (3D) structure of PSTD motifs in fish ST8Sia II. The 3D model of human ST8Sia II PSTD in addition to PSTD from *P. fluviatilis* and *C. maraena* was simulated, based on the 3D model of human ST8Sia IV PSTD (Protein Data Bank entry 6AHZ) using YASARA. The electrostatic potential surfaces are displayed. The exchanged amino acids are colored in an additional version of the 3D structure to highlight the position of the exchange: K2 → L2 (yellow), H4 → T4 (orange), N6 → D6 (magenta), Y11 → F11 (green), and K28 → N28 (red) for *P. fluviatilis* and K2 → L2 (yellow), H4 → T4 (orange), N6 → D6 (magenta), R8 → H8 (violet), Y11 → F11 (green), K28 → N28 (red), and H30 → Q30 (light blue) for *C. maraena*. For the determination of the 3D structure of human ST8Sia IV PSTD, a peptide with one additional amino acid on the N-terminus and two additional amino acids on the C-terminus of PSTD were used [66].

In addition to sequence alignments, we simulated the PSTD 3D structure of fish ST8Sia II and ST8Sia IV, based on the determined 3D structure of human ST8Sia IV PSTD (PDB 6AHZ) (Figure 7), which were published by Peng and colleagues [66]. Volkers et al. described that PSTD acts as a basic furrow, leading the nascent sialic acid chain to the active site of the polysialyltransferase [32]. The 3D simulation of the human ST8Sia IV PSTD shows that only significant differences between the electrostatic potential surfaces are detectable at the N-terminal region. Especially the orientation of the basic areas changed between the species. In contrast, the central and C-terminal area exhibited only minor changes. In the case of ST8Sia II, the most prominent alterations also occurred at the N-terminal domain (Figure 8). However, exchanging the N6 with aspartate, an exposed acidic segment is formed in Salmoniformes and Neoteleostei, which may influence the interaction between PSTD and the negatively charged sialic acid polymers. However, regarding the 3D simulation of PSTD, it has to be noted that a simulation is only a simulation and crystal structures of PSTD in addition to the whole enzymes are necessary for the generation of unambiguous 3D models.

Taken together, our sequence alignments and 3D simulations demonstrate that, in fish, characteristic alterations of the amino acid sequences occurred within PSTD and that several of these replaced amino acids are important for the enzymatic activity in the case of human ST8Sia IV, as demonstrated by Troy and co-workers [31]. These variations might also influence the ability of PSTD to interact with sialic acid chains consisting of other sialic acids than Neu5Ac, such as Neu5Gc and KDN, as well as their O-acetylated forms. However, to definitively proof this hypothesis of neofunctionalization of fish polysialyltransferases, their enzymatic activity has to be characterized in more detail.

### 2.5. Expression of Polyα2,8-Sialyltransferase Genes in C. Maraena Tissues

**Figure 9:**
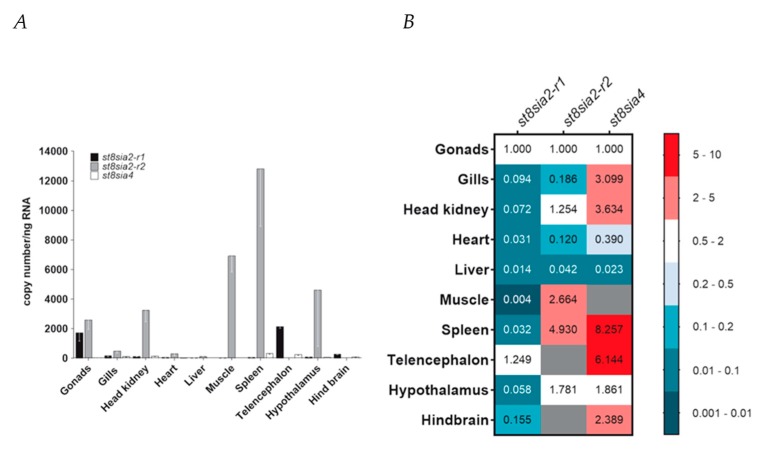
Expression profiling of poly-α2,8-sialyltransferase-encoding genes in maraena whitefish. (**A**) Transcript levels of *st8sia2-r1* (black bars), *st8sia2-r2* (gray), and *st8sia4* (blank) were determined in ten tissues from maraena whitefish (*n* = 4), as indicated on the abscissa. Bars represent the averaged copy numbers normalized against three reference genes; error bars represent the standard deviation. (**B**) A heat map represents the same copy numbers per target gene as shown in (**A**) relative to the expression in gonads (set as 1.0). These relative expression values are colored according to the code given at the right. Non-detectable transcript numbers are indicated by gray fields.

Having characterized the chromosomal localization, evolutionary history, and structure of the poly-α2,8-sialyltransferases ST8Sia II and ST8Sia IV encoded by the *st8sia2* and *st8sia4* genes, respectively, we eventually profiled their expression in ten organs and tissues of *C. maraena* as a representative of the Salmoniformes (Figure 9A,B). As *st8sia2* is duplicated in salmonid fishes, we investigated whether the expression of both genes is tissue-specific, and thus possibly function-specific. To this end, discriminating primer pairs for *st8sia2-r1* and *st8sia2-r2* as well as for *st8sia4* transcripts were designed. The RT-qPCR analysis revealed that *st8sia2-r1* transcripts were on low levels in liver, heart, spleen, head kidney, gills, hypothalamus, and hind brain (>300 copies/ng RNA), and almost absent in muscle (>10 copies/ng RNA) (Figure 9A). In stark contrast, the copy numbers of *st8sia2-r1* were at a high level in gonads (~1700 copies/ng RNA) and telencephalon (~ 2140 copies/ng RNA) (Figure 9B). The transcript levels of the gene copy *st8sia2-r2* were generally higher compared with its paralogue, ranging from a 1.5-fold difference in gonads to a 233-fold difference in spleen (Figure 9A). While the expression of *st8sia2-r2* was not detectable in hind brain and telencephalon, it exceeded the expression level of *st8sia2-r1* by 4622-fold in the hypothalamus.

The expression level of *st8sia4* was at a similarly low or even significantly lower level compared with that of *st8sia2-r1* with the highest copy numbers in spleen (~330 copies/ng RNA). No or only very few *st8sia4* transcripts were detectable in liver, muscle, and heart (Figure 9B). The results are partially different in comparison with the determined mRNA levels in rainbow trout using Northern blot analysis and semi-quantitative PCR [33]. For instance, spleen samples were negative for *st8sia2* transcripts, which might not only be the result of differences in the applied methods, but also in general differences between these two Salmoniformes.

Taken together, profiling the expression of the poly-α2,8-sialyltransferase genes revealed a tissue-specific expression pattern of *st8sia2* genes in *C. maraena* tissues indicative of their subfunctionalization. Probably one of the most striking differences between the expression profiles in maraena whitefish and humans is the presence of *st8sia2* and *st8sia4* transcripts in the reproductive tract. Whereas in humans, only a weak signal for *st8sia2* mRNA and no signal for *st8sia4* mRNA could be detected by Northern blotting [68], in *C. maraena,* the gonads belongs to the tissues with the highest expression levels of polysialyltransferases. This was already described by Kitajima and colleagues using rainbow trout ovaries [33]. Besides the gonads, remarkable differences were also observed in spleen. Contrary to humans, where no *st8sia2* mRNA was detectable [68], *st8sia2-r2* expression was extremely high in the spleen of *C. maraena*, indicating that ST8Sia II-r2 might play a role during immunologic reactions in maraena whitefish. Altogether, these results let us suggest that, in addition to the number of active polysialyltransferases, as well as their enzymatic activity, the physiological roles of these polysialyltransferases may have changed during the evolution of vertebrates.

## 3. Materials and Methods

### 3.1. Materials and Animals

Maraena whitefish were provided by the Institute for Fisheries of the State Research Centre for Agriculture and Fishery Mecklenburg-Western Pomerania (Born, Germany), and BiMES, Binnenfischerei GmbH (Friedrichsruhe, Germany). Fish were held in fresh-water recirculation systems with a 12:12 day-and-night cycle at 18 °C. Water quality was maintained by automated purification and disinfection (bio-filter and UV light). In addition, the concentrations of selected chemical and physical water parameters were constantly determined. 

Sampling of ten organs or tissues (gills; gonads; head kidney; heart; liver; muscle; spleen; and the brain regions hypothalamus, hind brain, and telencephalon) from four maraena whitefish followed the standards described in the German Animal Welfare and was approved by the Landesamt für Landwirtschaft, Lebensmittelsicherheit und Fischerei, Mecklenburg-Vorpommern, Germany (LALLF M-V/TSD/7221.3-1-069/18) in November 2018. The tissues were sampled rapidly and immediately frozen in liquid nitrogen to be kept at −80 °C until RNA extraction.

### 3.2. In Silico Identification and Phylogenetic Analysis of ST8Sia Sequences

A local alignment BLAST approach was used to retrieve the vertebrate *st8sia* nucleotide sequences with significant homology to the mammalian sequences from the genomic and Transcriptome Shotgun Assembly (TSA) divisions of the GenBank/EBI databases at the National Center for Biotechnology Information (NCBI) (last accessed on 27 September 2019), ENSEMBL (release 97) and from the PhyloFish database [7,14,37]. The protein sequence analysis was performed using the Expert Protein Analysis System (ExPASy; Swiss Institute of Bioinformatics, Switzerland; website (https://www.expasy.org/)). Sequence alignments were performed using the clustalW (PRABI; https://npsa-prabi.ibcp.fr/cgi-bin/npsa_automat.pl?page=/NPSA/npsa_clustalw.html). Phylogeny was determined aligning the known vertebrate ST8Sia sequences with MUSCLE in MEGA7.0 [40]. The multiple sequence alignments of the selected vertebrate ST8Sia sequences were conducted using MUSCLE and Clustal Omega algorithms in MEGA7.0 and manually refined (see Supplementary Data 1 and 2). Phylogenetic trees were produced by the neighbor-joining (NJ), maximum likelihood, and minimum evolution method in MEGA 7.0 [40,69]. 

To determine the consequences of duplication or loss of genes of a given order of Actinopterygian, we considered what happened on its closest paralogue. The amino acid substitutions that occurred at its base were deduced using the parsimony method implemented in Protpars program (PHYLIP package vers. 3.69) [70].

### 3.3. Synteny Analysis, Paralogon Detection, and Ancestral Genome Reconstruction

Synteny between the *st8sia* gene loci and neighbour genes in vertebrate genomes was assessed by manual chromosome walking and reciprocal BLAST. Detection of paralogous blocks was visualized with Genomicus (version 97.01) http://www.genomicus.biologie.ens.fr/genomicus-92.01/cgi-bin/search.pl, last accessed August 2019 [53]. When the *st8sia* gene of interest was not found in a genome, physically close genes were used as a seed to identify syntenic segments.

### 3.4. Sequence Alignments, Motifs Analysis, and 3D Simulation of PSTD

Multiple sequence alignments were performed with MUSCLE of EMBL-EBI (version 3.8; https://www.ebi.ac.uk/Tools/msa/muscle/) from selected species using published sequences (accession numbers in Supplemental Data 1). The sequences were conducted, edited, and annotated in Jawa Alignment Viewer Jalview 2.11.0, and manually refined [67]. The 3D structure of the human PSTD of ST8Sia II as well as ST8Sia IV was generated in YASARA (Version 19.9.17) using the following Protein Data Bank (PDB) entries: ST8Sia IV (code: 6AHZ) (PDB, https://www.rcsb.org/pdb/home/sitemap.do https://www.wwpdb.org/pdb?id=pdb_00006ahz). The ST8Sia II was generated in YASARA changing the amino acid in the positions N3, K4, L5, and K6, corresponding to H3, H4, V5, and N6, respectively. The human amino acid sequences of ST8Sia II and ST8Sia IV were modified at the positions with the following amino acids according to the different fish PSTD sequences: *C. maraena* ST8Sia II, L2, T4, D6, H8, F11, N28, and Q30; *P. fluviatilis* ST8Sia II, L2, T4, D6, F11, and N28; I. punctatus ST8Sia IV, R3, P6, H8, M9, V17, and N30; and *C. maraena* ST8Sia IV, V1, R3, R6, I29, and N30. We designed only one PSTD motif of *C. maraena* because there is only one difference at position R22 between ST8Sia II-r1 and ST8Sia II- r2.

### 3.5. RNA Extraction, cDNA Synthesis, Primer Design, and RT-qPCR

Total RNA was isolated from the individually homogenized organs and tissues using TRIzol (Invitrogen/Thermo Fisher Scientific, Darmstadt Germany), followed by an additional purification step (RNeasy Mini Kit, Qiagen). The quantity and integrity of the isolated RNA were determined using the NanoDrop 2000 photometer (Thermo Fisher Scientific) and agarose gel electrophoresis. Subsequently, we reverse-transcribed the total RNA using the SuperScript II Reverse Transcriptase (Thermo Fisher Scientific) and a mixture of oligo-d(T) and random hexanucleotides. This reaction was carried out at 42 °C (50 min), followed by an inactivation step (70 °C, 15 min). The resulting cDNA was diluted in 100 µL distilled water. 

Real-time fluorescence-based quantitative RT-PCR (RT-qPCR) was used to determine the mRNA abundance of the two *st8sia2* gene variants in the above ten organs and tissues of maraena whitefish (*n* = 4). To this end, we identified discriminating sequence motifs to derive the oligonucleotides for *st8sia2-r1* (sense, 5′-AGCCTCATCAGGAAGAACATCC-3′; antisense, 5′-TTCCCTACGATGGCACAGCGT-3′) and *st8sia2-r2* (sense, 5′-CGTTCAACAGGAGCCTCTCTAA-3′; antisense, 5′-TTCCCTACGATGGCACAGCGC-3′). Moreover, we designed a *st8sia4*-specific primer pair (sense, 5′-ATGATAAGGAAGGACGTGCTGC-3′; antisense, 5′-TGTTGAGCGTTCGGCGTCTGT-3′). These RT-qPCR primers were designed (Pyrosequencing Assay Design software v.1.0.6; Biotage, Uppsala, Sweden) to synthesize amplicons between 121 bp and 226 bp. *eef1a1a2* (encoding eukaryotic translation elongation factor, variant a2), *rpl9*, and *rpl32* (ribosomal proteins L9 and L32) were selected as reference genes [71]. The RT-qPCR analyses were conducted with the LightCycler 96 System (Roche, Mannheim, Germany) using the SensiFAST SYBR No-ROX Kit (Bioline, Luckenwalde, Germany). We only considered crossing point (CP) values >35 for The expression analysis of the *st8sia2-r1 st8sia2-r2*, and *st8sia4*. The calculation of their copy numbers was based on standard curves having been generated on 10-fold dilutions of the respective PCR-generated fragments (1 × 10^3^ to 1 × 10^6^ copies). Melting-curve analyses validated the amplification of the distinct products. Amplicons were visualized on 3% agarose gels in order to assess product size and quality.

### 3.6. Data Availability

To identify the maraena whitefish ST8Sia II sequences, the orthologous sequences from rainbow trout and Atlantic salmon were aligned with the software Bowtie2 (v 2.2.4) to our RNA-seq read collection from maraena whitefish [72]. The alignments were then indexed and sorted with the software package Samtools (v.16) and the final consensus sequences were obtained with the Ugene software (v 1.29).

## 4. Conclusions

In this study, we highlighted an expansion and particular distribution of the ray-finned fish ST8Sia repertoire owing to several duplications and loss events of *st8sia* genes, and we refined their evolutionary history. Our analyses of the molecular evolution in ST8Sia sequences and in key functional motifs (i.e., motif L and PSTD) let us suggest that the polysialyltransferases might evolved new enzymatic activities and/or specificities in the course of Vertebrate evolution. Their expression profiles in Salmonid tissues differ from those observed in mammals and further point to a subfunctionalization of these poly-α2,8-sialylatransferases. Altogether, we have laid the foundation for further studies towards understanding of the remarkable differences between α2,8-linked polySia chains found in mammals and fish.

## Figures and Tables

**Table 1:** Fish orders that have lost *st8sia* genes.

Missing Sialyltransferase	Fish Order
*st8sia2*	Siluriformes, Gymnotiformes, Esociformes
*st8sia4*	Perciformes, Tetraodontiformes, Beloniformes, Cichliiformes, Cyprinodontiformes, Gadiformes, Gobiiformes, Pleuronectiformes, Anabantiformes, Syngnathiforme, Synbranchiformes
*st8sia9*	Cypriniformes, Siluriformes, Clupeiformes, Gymnotiformes, Characiformes, Lepisosteiformes, Amiiformes
*st8sia7*	Osteoglossiformes, Cichliiformes, Clupeiformes, Cyprinodontiformes, Gadiformes, Gobiiformes, Pleuronectiformes, Siluriformes

## Supplementary Materials

Supplementary materials can be found at https://www.mdpi.com/1422-0067/21/2/513/s1.

## Abbreviations

DP	degree of polymerization
Neu5Ac	N-acetylneuraminic acid
Neu5Gc	N-glycolylneuraminic
oligoSia	oligosialic acid
polySia or PSA	polysialic acid
PDB	Protein Data Bank
PSGP	salmonid egg polysialoglycoprotein
ST8Sia	α2,8-sialyltransferase
WGD	whole genome duplication
KDN	2-keto-3-deoxynononic acid
MSA	multiple sequence alignment
