# Supplementary material for: Vertebrate Alpha2,8-Sialyltransferases (ST8Sia): A Teleost Perspective
Source: Int J Mol Sci. 2020 Jan 14;21(2):513. doi: 10.3390/ijms21020513 (PMC7014012; doi:10.3390/ijms21020513)
Supplement: Supplementary file 1 [file ijms-21-00513-s001.zip › ijms-668545-supplementary - final/Supplemental data 2 Venuto.pdf]

Vertebrate alpha2,8-sialyltransferases (ST8Sia): a teleost perspective.

Marzia Tindara Venuto[1], Mathieu Decloquement[2], Joan Martorell Ribera[3], Maxence Noel[2], Alexander Rebl[3], Virginie Cogez[2], Daniel Petit[4], Sebastian Peter Galuska[1], Anne Harduin-Lepers[2‡]

[1]Institute of Reproductive Biology, Leibniz Institute for Farm Animal Biology (FBN), Wilhelm-Stahl-Allee 2, 18196 Dummerstorf, Germany

[2]Université de Lille, CNRS, UMR 8576 - UGSF - Unité de Glycobiologie Structurale et Fonctionnelle, F-59000 Lille, France

[3]Institute of Genome Biology Leibniz Institute for Farm Animal Biology (FBN), Wilhelm-Stahl-Allee 2, 18196, Dummerstorf, Germany

[4]Glycosylation et différenciation cellulaire, EA 7500, Laboratoire PEIRENE, Université de Limoges, 123 avenue Albert Thomas, 87060 Limoges Cedex, France

[‡]Corresponding author : Anne Harduin-Lepers, Unité de Glycobiologie Structurale et Fonctionnelle, UMR CNRS 8576, Université de Lille, Faculté des sciences et Technologies, 59655 Villeneuve d'Ascq, France. Phone: +33 320 33 62 46; Fax: +33 320 43 65 55; E-mail: anne.harduin-lepers@univ-lille.fr

orcid.org/0000-0002-1233-3799

Supplementary data 2: Multiple sequence alignments (clustalX) of ST8Sia sequences used to draw: A) supplemental figure 1; B) figure 2; C) figure 5

A) Multiple sequence alignments (ClustalX) of 324 ST8Sia sequences used in supplemental figure 1

Mega

```
CLUSTAL X (1.81) multiple sequence alignment
               1
Latcal_3       IVFSIGENFRSLLPE----VSPILN---KHYNVCAVVGNSGILTGSRCGP
Perflu_3       IVFSIGENFHSLLPA----VSPILN---KHYNVCAVVGNSGILTGSHCGA
Serdum_3       IVFSIGENFRSLLPE----VSPILN---KHYNVCAVVGNSGILTGSRCGP
Stepar_3       IVFSIGENFRSLLPE----ASPILN---KHYNVCAVVGNSGILTGSRCGP
Tetcal_3       VVFSVSNSFRSLLPE----VPPILK---MHYNTCAVVGNSGILTGSSCGA
Monalb_3       IVFSISENFRSLLPK----VSPVLN---KHYNVCAVVGNSGILTGSRCGP
Molmol_3       IVFSIGENFRSLLPE----TSPILN---KHFNVCAVVGNSGILTGSRCGA
Takrub_3       VVFSIGENFRSLLPE----TSPILQ---KHYNVCAVVGNSGILTGSRCGQ
Canlupfam_3    VVFSISNNFRSLLPD----VSPIVN---KHFNICAVVGNSGILTGSRCGQ
Bostau_3       VVFSISNNFRSLLPD----VSPIVN---KRYNICAVVGNSGILTGSRCGP
Homsap_3       VVFSISNNFRSLLPD----VSPIMN---KHYNICAVVGNSGILTFIQCGR
Siltro_3       VVFSISNNFRSLLPD----TSPVMN---KRYNVCAVIGNSGILTESQCGA
Anocar_3       VVFSISNSFRMLLPD----VSPIQN---KHYNICAVVGNSGILVGSQCGQ
Musmus_3       VVFSISNNFRSLLPD----VSPIMN---KRYNVCAVVGNSGILTGSQCGQ
Pantro_3       VVFSISNNFRSLLPD----VSPIMN---KHYNICAVVGNSGILTGSQCGQ
Ratnor_3       VVFSISNNFRSLLPD----VSPILN---KRYNICAVVGNSGILTGSQCGQ
Galgal_3       VVFSISNNFRSLLPD----VSPILN---KHYNVCAVVGNSGILTGSQCGQ
Lepocu_3       VVFSISNNFRSLLPD----TSPILN---KQYNVCAVVGNSGILTGSQCGP
Tetnig_3       IVFSIGENFRSLLPE----VSPIRQ---KHYNVCAVVGNSGILTGSRCGQ
Squaca_3       VVFSVSNSFRSLLPE----VPPILK---MHYNTCAVVGNSGILTGSGCGA
Perfla_3       IVFSIGENFHSLLPA----VSPILN---KHYNVCAVVGNSGILTGSRCGA
Salsal_3       IVFSIGENFKSLLPE----ASPIVN---KHYNVCAVVGNSGILTGSRCGP
Serlaldor_3    IVFSIGENFRSLLPE----ASPILN---KHYNVCAVVGNSGILTGSRCGP
Treber_3       IVFSISNNLKSLLPD----TSPIRN---KHYSMCAVVGNSGILTGSHCGP
Cynsem_3       IVFSIGENFRSLLPE----VSPIVN---KHYNVCAVVGNSGILTGSRCGP
Paroli_3       IVFSISNNFKSLLPD----ASPVLN---KHYSVCAVVGNSGILTGSHCGP
Paroli_3b      IVFSIGENFRSLLPD----ASPILN---KHYNVCAVVGNSGILTGSRCGP
Plaste_3       IVFSISNNFKSLLPD----ASPLLN---KHYSVCAVVGNSGILTGSHCGP
Plaste_3b      IVFSIGENFRSLLPE----ASPILN---KHYNVCAVVGNSGILTGSRCGP
Erpcal3        VVFSISNNFKSLLPD----ASPVLN---KHYNVCAVVGNSGILTGSQCGP
Calmil_3b      VVFSISNNFRMLLPE----VSPLLK---MHYNTCAVVGNSGILRGSRCGD
Hetzeb_3       IVFSVSNSFRSLLPD----VPPILK---MHYNTCAVVGNSGILIGSRCGA
Latcha_3       VVFSISNHFKSLLPD----VSPILN---KCYNICAVVGNSGILTDSQCGA
Anates_3       IVFSIGENFRSLLPD----ASPILN---KHYNVCAVVGNSGILTGSRCGP
Ampcit3        IVFSISENFRSLLPE----VSPILN---KHYNVCAVVGNSGILTGSRCGP
Auslim_3       IVYSIGENFRSLLPE----ASPILN---KRYNVCAVVGNSGILTGSRCGA
Cypvar_3       IVYSIGENFRSLLPE----VSPILN---KQYNVCAVVGNSGILTGSRCGA
Krymar_3       IVYSIGENFRSLLPE----VSPILD---KHYNVCAVVGNSGILTGSRCGA
Funhet_3       IVYSIGENFRSLLPE----ASPVLN---KRYNVCAVVGNSGILTGSRCGA
Gamaff3        IVYSIGDNFRSLLPD----VSPILN---MRYNVCAVIGNSGILTGSRCGA
Orylat_3       MVYSIGENFRSLLPE----VSPILN---RHYNVCAVVGNSGILTGSLCGA
Orenil_3       IVFSISENFRSLLPE----VSPILN---KHYNVCAVIGNSGILTGSRCGP
Mayzeb_3       IVFSISDNFRSLLPE----VSPILN---KHYNVCAVIGNSGILTGSRCGP
Punnye_3       IVFSISDNFRSLLPE----VSPILN---KHYNVCAVIGNSGILTGSRCGP
Punnye_3a      IVFSISDNFRSLLPE----VSPILN---KHYNVCAVIGNSGILTGSRCGP
Neobri_3       IVFSISDNFRSLLPE----VSPILN---KHYNVCAVIGNSGILTGSRCGP
Misang_3       VVFSIGENLRSLLPD----SSPIIN---KRYNTCAVIGNSGILTGSRCGP
Sinrhi3        IVFSIGENLRSLLPD----SSPVLN---KHFNTCAVVGNSGILTGSRCGA
Singra_3       VVFSIGENLRSLLPD----SSPVLN---KRFNSCAVVGNSGILTGSRCGT
Hapbur_3       IVFSISDNFRSLLPE----VSPILN---KHYNVCAVIGNSGILTGSRCGP
Astcal_3       IVFSISDNFRSLLPE----VSPILN---KHYNVCAVIGNSGILTGSRCGP
Notfur_3       IVYSIGENFRSLLPN----VSPILN---KHYNVCAVVGNSGILTGSRCGT
Cypcar_3       YVFSIGENLRSLLPD----SSPVLN---KRFNSCAVVGNSGILTGSRCGA
Poefor3        IVYSIGENFRSLLPE----VSPVLN---KRYNICAVIGNSGILTGSRCGA
Poelat_3       IVYSIGENFRSLLPE----VSPVLN---KRYNICAVIGNSGILTGSRCGA
Poeret_3       IVYSIGENFRSLLPE----VSPVLN---KRYNVCAVIGNSGILTGSRCGA
Angang_3       VVFSIGENLMSLLPE----SSPIVPE--RRFNVCALVGNSGVLTGSRCGP
Xipmac_3       IVYSIGENFRSLLPE----VSPVLN---KRYNVCAVIGNSGILTGSRCGA
Masarm_3       IVFSIGENFYSLVPE----VSPILN---KHYNICAIIGNSGILSGSRCGS
Permag_3       VVYSIGENFYSLLPE----ASPILH---RHYKRCAVVGNSGILTGSKCGT
Gnapet3        VVFSISSNFKSLLPE----SSPIQN---KHYNVCAVVGNSGILTGSSCGP
Ostbic_3       VVFSVSSSFRSLLPD----SSPIEK---KHYNVCAVVGNSGILTGSSCGP
```

```
Gadmor_3        IVFSIGQNFHSLLPE----ASPILN---KHYNVCAVVGNSGILTGSKCGP
Sclfor_3        VVFSVSGSFRSLLPD----SSPVEK---KHYNVCAVVGNGGILTGSSCGP
Acapol3         IVFSIGENFRSLLPD----VSPILN---KHYNVCAVIGNSGILTGSRCGP
Ampoce_3        IVFSIGENFRSLLPD----VSPILN---KHYNVCAVVGNSGILTGSRCGP
Sclfor_3b       VVFAISNNFKSLLPV----TSPIQN---KHYNTCAVVGNSGILTSSHCGH
Ostbic_3b       VVFAISNNFKSLLPE----TSPIQN---KHYNTCAVVGNSGILTSSHCGH
Parkin_3        VVFSISSNFKSLLPE----SSPIQN---KHYNVCAVVGNSGILTGSSCGH
Ampper3         IVFSIGENFRSLLPD----VSPILN---KHYNVCAVVGNSGILTGSRCGP
Oncmyk_3        IVFSIGENFKSLLPE----ASPIVN---KHYNVCAVVGNSGVLTGSRCGP
Gasacu_3        IVFSIGENFRSLLPE----VSPILN---KHYNVCAVVGNSGILTGSRCGP
Labber_3        IVFSIGENFRSLLPE----VSPILN---KHYNVCAVIGNSGILTGSRCGP
Panbuc_3        VVFSISSSFRSLLPD----HSPILN---KHYNTCAVVGNSGILIGSRCGP
Parhas3         VVFSIGENFRSLLPD----SSPVLN---KRYNTCAVVGNSGILTGSRCGA
Cluhar_3        VVFSIGENFRSLLPE----LSPILN---KRYNTCAVVGNSGILTGSRCGP
Aloalo_3        VVFSIGENFRSLLPE----ISPILN---KRYNTCAVVGNSGILTGSRCGP
Panhyp_3        VVFSIGENFRSLLPD----SSPILN---KRYNTCAVVGNSGILTGSRCGA
Eleele_3        VVFSIGENFRSLLPD----SSPILN---KLYNTCAVVGNSGILTGSRCGA
Denclu_3        IVFSIGENFKSLLPE----TSPVMN---KLYNTCAVVGNSGILMGSRCGP
Sarpil_3        VVFSIGENLRSLLPE----ISPILN---KRYNTCAVVGNSGILTGSRCGP
Eigvir_3        VVFSIGENFRSLLPD----SSPILN---KRYNTCAVVGNSGILTDSRCGS
Aptalb_3        VVFSIGENFRSLLPD----SSPVLN---KRYNTCAVVGNSGILTGSRCGA
Sinans_3        VVFSIGENLRSLLPD----SSPVLN---RRFNTCAVVGNSGILTGSRCGA
Pygnat_3        VVFSIGENFRSLLPD----SSPILN---KRYNTCAVIGNSGILTGSRCGP
Astmex_3        IVFSIGENFRSLLPD----SSPVLN---KRYNTCAVVGNSGILTGSRCGP
Caraur_3        VVFSIGENLRSLLPD----SSPVLN---KRFNSCAVVGNSGILTGSRCGA
Danrer_3        VVFSIGENLRSLLPD----ASPVLN---KRYNTCAVVGNSGILTGSRCGP
Ictpun_3        YVFSIGENFRSLLPD----SSPILN---KRYNTCAVVGNSGILTGSRCGA
Plealt_3        YVFSIGENFKSLLPE----ASPVVN---KHYNVCAVVGNSGILTGSRCGA
Plealt_9        YVFSISNNFKSLLPE----TSPILN---KHYNVCAVVGNSGILTGSHCGS
Gasacu_9        IVFSISNNLKSLLPD----ASPIRN---KHYSVCAVVGNSGILTGSHCGP
Parkin_9        VVFAISGNFKSLLPD----SSPILN---KHYNMCAVVGNSGILTGSRCGP
Acapol_9        IVFSISNNFKSLLPE----TSPIHN---KHYSMCAVVGNSGILTGSHCGP
Larcro_9        IVFSISNNFKSLLPE----TSPIHN---KHYSICSVVGNSGILTGSHCGP
Notcor_9        IVFSISNNLKSLLPD----TSPIRN---KHYSMCAVVGNSGILTGSHCGP
Latcal_9        IVFSISNNFKSLLPD----TSPILN---KHYSMCAVVGNSGILTGSHCGP
Serlaldor_9     IVFSISNNFKSLLPD----TSPILN---KHYSMCAVVGNSGILTGSHCGP
Monalb_9        IVFSISNSFKSLLPD----TSPILN---KHYSMCAVVGNSGILTGSHCGP
Serdum_9        IVFSISNNFKSLLPD----TSPILN---KHYSMCAVVGNSGILTGSHCGP
Perflu_9        IVFSISNNLKSLLPD----TSPIHN---KHYSMCAVVGNSGILTGSHCGP
Perfla_9        IVFSISNNLKSLLPD----TSPIHN---KHYSMCAVVGNSGILTGSHCGP
Stepar_9        IVFSISNNFKSLLPE----TSPIHN---KHYSVCAVVGNSGILTGSHCGP
Molmol_9        IVFSISNNFKSLLPD----TSPIRN---KHYSICSVVGNSGILTGSHCGP
Hipcom_9        IVFSISNNFKSLLPD----ASPILD---KHYGTCAVVGNSGILTGSHCGS
Takrub_9 B      IVFSISNNFKSLLPD----TSPIHN---KHYSLCSVVGNSGILTGSHCGD
Takrub_9        IVFSISNNFKSLLPD----TSPIHN---KHYSLCSVVGNSGILTGSHCGA
Cynsem_9        IVFSISNNFKSLLPD----ISPILN---KHYSMCAVVGNSGILTGSHCGP
Scomax_9        IVFSISNNLKSLLPD----ISPILN---KHYSVCAVVGNSGILTGSHCGP
Corlav_9        VVFSISNNLKSLLPD----ASPIQN---KHYNVCAVVGNSGILTGSHCGP
Oncmyk_9        VVFSISNNLKSLLPD----ASPIQN---KHYNVCAVVGNSGILTGSHCGP
Salsal_9        VVFSISNNLKSLLPD----ASPIQN---KHYNVCAVVGNSGILTGSHCGP
Onctsh_9        VVFSISNNLKSLLPD----ASPIQN---KHYNVCAVVGNSGILTGSHCGP
Salalp_9        VVFSISNNLKSLLPD----ASPIQN---KHYNVCAVVGNSGILTGSHCGP
Ampoce_9        IVFSISNNFKSLLPE----TSPIHN---KHYSMCAVVGNSGILTGSHCGP
Ampper9         IVFSISNNFKSLLPE----TSPIHN---KHYSMCAVVGNSGILTGSHCGP
Labber_9        IVFSISNNFKSLLPD----TSPIRN---KHYSICSVVGNSGILTGSHCGP
Umbpyg_3B       IVFSIGENFQSLLPE----VSPIVD---KHYNVCAIVGNSGILTGSRCGP
Esoluc_3b       VVFSIGENFKSLLPE----ASPIAN---KHYNVCAVVGNSGILTGSRCGP
Gadmor_9        IVFSISNNFKSLLPD----MSPIYN---KHYNVCAVVGNSGILTGSRCGN
Umbpyg_9        VVFSISNNLKSLLPD----ASPIQN---KHYNVCAVVGNSGILTGSHCGP
Esoluc_9        VVFSISNNLKSLLPD----ASPIQN---KHYNVCAVVGNSGILTGSHCGP
Permag_9        IVFSISNNFKSLLPD----TSPILN---KHYSVCSVVGNSGILTDSHCGP
Bolpec_9        IVFSISNNFKSLLPD----TSPILN---KHYSVCSVVGNSGILTDSHCGP
Poefor9         IVFSISNNFKSLLPE----ASPILN---KHYSTCAVVGNSGILTGSHCGP
Masarm_9        IVFSISNNFKSLLPD----TSPILN---KHYSMCAVVGNSGILTGSHCGP
Angjap_9        VVFSIGENFMSLLPE----SSPIVPE--RRFNVCALVGNSGVLTGSRCGP
Poelat_9        IVFSISNNFKSLLPE----ASPILN---KHYSTCAVVGNSGILTGSHCGP
Poeret_9        IVFSISNNFKSLLPE----ASPILN---KHYSTCAVVGNSGILTGSHCGP
Xipmac_9        IVFSISNNFKSLLPD----ASPIRN---KHYSTCAVVGNSGILTGSHCGP
Hapbur_9        IVFSISNNFKSLLPD----TSPILN---KHYSMCAVVGNSGILTGSHCGP
Orylat_9        IVFSISNNFKSLLPE----ISPILN---KHYSVCAVVGNSGILTGSHCGP
Gamaff_9        IVFSISNNFKSLLPD----ASPIRN---KHYSTCAVVGNSGILTGSHCGP
Neobri_9        IVFSISNNFKSLLPD----TSPILN---KHYSMCAVVGNSGILTGSHCGP
Funhet_9        IVFSISNNFKSLLPE----TSPILN---KHYSTCAVVGNSGILTGSHCGP
Punnye_9        IVFSISNNFKSLLPD----TSPILN---KHYSMCAVVGNSGILTGSHCGP
Cypvar_9        IVFSISNNFKSLLPE----TSPILN---KHYSTCAVVGNSGILTGSHCGP
Krymar_9        IVFSISNNFKSLLPD----ISPIFN---KHYSMCAVVGNSGILTGSHCGP
Notfur_9        IVFSISNNFKSLLPD----TSPILN---KHYSMCAVVGNSGILTGSHCGP
```

```
Notpie_9       IVFSISNNFKSLLPD----TSPILN---KHYSMCAVVGNSGILTGSHCGP
Orenil_9       IVFSISNNFKSLLPD----TSPILN---KHYSMCAVVGNSGILTGSHCGP
Auslim_9       IVFSISNNFKSLLPE----TSPITN---RHYSVCAVVGNSGILTGSHCGP
Anates_9       IVFSISNNFKSLLPD----TSPIHN---KHYSMCAVVGNSGILTGSHCGP
Astcal_9       IVFSISNNFKSLLPD----TSPILN---KHYSMCAVVGNSGILTGSHCGP
Mayzeb_9       IVFSISNNFKSLLPD----TSPILN---KHYSMCAVVGNSGILTGSHCGP
Orymel_9       IVFSISNNFKSLLPD----ISPILN---KHYSVCAVVGNSGILTGSHCGP
Ampcit9        IVFSISNNFKSLLPD----TSPILN---KHYSMCAVVGNSGILTGSHCGP
Macfas_2       ATMNVSQNLYELLPR----TSPLKN---KHFGTCAIVGNSGVLLNSGCGQ
Macmul_2       ATMNVSQNLYELLPR----TSPLKN---KHFGTCAIVGNSGVLLNSGCGQ
Macnem_2       ATMNVSQNLYELLPR----TSPLKN---KHFGTCAIVGNSGVLLNSGCGQ
Musmus_2       ATMNVSQNLYELLPR----TSPLKN---KHFQTCAIVGNSGVLLNSGCGQ
Ponabe_2       ATMNVSQNLYELLPR----TSPLKN---KHFGTCAIVGNSGVLLNSGCGQ
Papanu_2       ATMNVSQNLYELLPR----TSPLKN---KHFGTCAIVGNSGVLLNSGCGQ
Nomleu_2       ATMNVSQNLYELLPR----TSPLKN---KHFRTCAIVGNSGVLLNSGCGQ
Pantro_2       ATMNVSQNLYELLPR----TSPLKN---KHFGTCAIVGNSGVLLNSGCGQ
Latcha_2       ATMNISQNLYKLLPR----VSPLKN---RHFQNCAIVGNSGILLNSNCGR
Gnapet2        ATMNISDSLYQLLPT----VSPMKN---QHHRRCAIVGNSGILLNSSCGP
Panbuc_2b      ATTNVSENLYQLLPS----VSPLKH---QHYGRCAIVGNSGILLNSSCGQ
Ostbic_2       ATMNVSNELYELLPK----VSPLKN---QFHKRCAIVGNSGILLNSSCGK
Sinrhi2        ATTNISENLYHLLPT----VSPMKN---QHYKQCAIVGNSGILLNSSCGR
Hetzeb_2       ATLNISHSLYELLPR----TSPMKN---KHFKQCAIVGNSGILLNSGCGQ
Hipcom_2       PTTNISENLHRLLPS----VSPLKN---RHYRLCAIVGNSGILLNSSCGS
Prigla_2       ATLNVSHSLYELLPR----ISPMKN---KHYKQCAIVGNSGILLNSGCGQ
Scytor_2       ATLNISHSLYELLPR----TSPMKN---KHFKQCAIVGNSGILLNSGCGQ
Tetcal_2       ATLNISYSLYELLPR----TSPMKN---KHFKQCAIVGNSGILLNSGCGQ
Rhityp_2       ATLNISHSLYELLPR----TSPMKN---KHFKECAIVGNSGILLKSGCGQ
Parkin_2b      ATTNVSENLHALLPT----TSPMKN---RHYQRCAIVGNSGILLNSSCGK
Angang_2       ATVNVSESLYRLLPT----VSPMKD---RHYQRCAIVGNSGILLNSSCGR
Pygnat_2       ATTNISENLYRLLPT----ASPMKN---QHYEQCAIVGNSGILLNSSCGA
Parkin_2       ATMNVSASLYQLLPT----VSPMKN---QHHKRCAIVGNSGILLNSSCGP
Gorgor_2       ATMNVSQNLYELLPR----TSPLKN---KHFGTCAIVGNSGVLLNSGCGQ
Thegel_2       ATMNVSQNLYELLPR----TSPLKN---KHFGTCAIVGNSGVLLNSGCGQ
Tupchi_2       ATMNVSQNLYELLPR----TSPLKN---KHFRTCAIVGNSGVLLNSGCGQ
Galgal_2       ATMNVSQNLYELLPR----TSPLKG---KQFPSCAIVGNSGVLLGSGCGP
Eleele_2       ATTNISENLYRLLPT----VSPMKN---QHYKRCAIVGNSGILLNSSCGP
Danrer_2       ATTNISENRYHLLPT----VSPMKN---QHYRKCAIVGNSGILLNSSCGR
Canlupfam_2    ATMNVSQNLYDLLPR----TSPLKN---KHFRTCAIVGNSGVLLNSGCGQ
Aloalo_2       PTMNVSENLYRLLPT----ASPMKN---QRHKRCAIVGNSGILLNSSCGS
Astmex_2       ATTNISENLYRLLPT----VSPMKN---QHYERCAIVGNSGILLNSSCGP
Sinans_2       ATTNISENLYHLLPT----VSPMKN---QHYKQCAIVGNSGILLNSSCGR
Sclfor_2       ATLNVPNELYELLPR----VSPLKN---QFYKRCAIVGNSGILLNSSCGQ
Ceraty_2       ATMNVSQNLYELLPR----TSPLKN---KHFGTCAIVGNSGVLLNSGCGQ
Anocar_2       ATMNVSQNLYALLPR----TSPLKG---KHFRTCAIVGNSGILLNSGCGE
Anates_2       PTTNISENLYRLLPT----VSPMKN---QHHRRCAIVGNSGILLNSSCGH
Ampbic_2       PTTNISENLYRLLPT----VSPMKN---QHHRRCAIVGNSGILLNSSCGL
Sarpil_2       PTMNVSENLYSLLPT----ASPMKN---QHHKRCAIVGNSGILLNSSCGS
Xenlae_2       ATMNISKNLYELLPR----TSPLKN---KHFKTCAIVGNSGILLNSGCGK
Cluhar_2       PTMNVSENLYRLLPT----ASPMKN---QHHKSCAIVGNSGILLNSSCGP
Cypcar_2       ATTNISENLYNLLPT----VSPMKN---QHYKQCAIVGNSGILLNSSCGR
Denclu_2       ATMNISENLYRLLPT----ASPMKN---QHHRLCAIVGNSGILLNSSCGP
Notpie_2       PTTNISENLYRLLPT----ASPMKN---QHHRKCAIVGNSGILLNSSCGP
Erpcal2        ATMNISKSLYQLIPK----QSPLKG---QHFQKCAIVGNSGVLLNSGCGK
Amical_2       ATMNISETLYQLIPK----VSPMKQ---QHYRKCAIVGNSGILLNSRCGE
Lepocu_2       ATMNVSETLYQLIPK----VSPMKK---QHYRQCAIVGNSGILLKSRCGA
Homsap_2       ATMNVSQNLYELLPR----TSPLKN---KHFGTCAIVGNSGVLLNSGCGQ
Bostau_2       ATMNVSQNLYELLPR----TSPLKN---KHFGTCAIVGNSGVLLNSGCGQ
Caraur_2       ATTNISENLYNLLPT----VSPMKN---RHYKQCAIVGNSGILLNSSCGR
Thythy_2B      PTTNISENLYRLLPT----TSPMKN---QYHRRCAIVGNSGVLLNSSCGP
Onctsh_2B      PTTNISENLYQLLPT----ASPMKN---QHHRHCAIIGNSGILLNSSCGP
Salsal_2B      PTTNISENLYQLLPT----VSPMKN---QYHRRCGIIGNSGILLNSSCGP
Oncmyk_2B      PTTNISENLYQLLPT----ASPMKN---QHHRHCAIIGNSGILLNSSCGP
Onckis_2B      PTTNISENLYQLLPT----ASPMKN---QHHRHCAIIGNSGILLNSSCGP
Salsal_2A      PTTNISENLYRLLPT----ASPMKN---QHHRRCAIVGNSGILLNSSCGP
Thythy_2A      PTTNISENLYRLLPT----ASPMKN---QHHRRCAIVGNSGILLNSSCGP
Salalp_2A      PTTNISENLYRLLPT----ASPMKN---QHHRRCAIVGNSGILLNSSCGP
Corlav_2A      PTTNISENLYRLLPT----ASPMKN---QHHRRCAIVGNSGILLNSSCGP
Cormar_2A      PTTNISENLYRLLPT----ASPMKN---QHHRRCAIVGNSGILLNSSCGP
Onctsh_2A      PTTNISENLYRLLPT----ASPMKN---QHHRSCAIVGNSGILLNSSCGP
Oncmyk_2A      PTTNISENLYQLLPT----ASPMKN---QHHRSCAIVGNSGIQLNSSCGP
Neobri_2       PTTNISENLYRLLPT----VSPMKN---QHYRRCAIVGNSGILLNSSCGP
Takrub_2       PTTNVSETLYHFLPT----VSPMKN---QHHRRCAIVGNSGILLNSSCGP
Tetnig_2       PTTNISETLYHFLPS----VSPMKN---QHHRRCAIVGNSGVLLNSSCGP
Orenil_2       PTTNISENLYRLLPT----ISPMKN---QHYRRCAIVGNSGILLNSSCGP
Orylat_2       PTTNISENLYRLLPT----VSPMKN---QHHRRCAIVGNSGILLNSSCGS
Krymar_2       PTTNISENLYRLLPT----ASPMRR---QHHRRCAIVGNSGVLLNSSCGP
Notfur_2       PTTNISENLYRLLPT----ASPMKN---QHHRKCAIVGNSGILLNSSCGP
```

```
Poelat_2       PTTNISENLYRLLPT----ASPMKN---QYHRRCAIVGNSGILLNSSCGP
Gadmor_2       PTTNISENLYRLLPT----VSPMRN---QHHRKCAIVGNSGILLNSSCGQ
Xipmac_2       PTTNISENLYRLLPT----ASPMKN---QYHRRCAIVGNSGILLNSSCGP
Poeret_2       PTTNISENLYRLLPT----ASPMKN---QYHRRCAIVGNSGILLNSSCGS
Astcal_2       PTTNISENLYRLLPT----VSPMKN---QHYRRCAIVGNSGILLNSSCGP
Hapbur_2       PTTNISENLYRLLPT----VSPMKN---QHYRRCAIVGNSGILLNSSCGP
Acapol_2B      PTTNISENLYRLLPT----VSPMKN---QHHRRCAIVGNSGILLNSSCGL
Ampoce_2A      PTTNISENLYRLLPT----VSPMKN---QHHRRCAIVGNSGILLNSSCGL
Acapol_2A      PTTNISENLYRLLPT----VSPMKN---QHHRRCAIVGNSGILLNSSCGL
Mayzeb_2       PTTNISENLYRLLPT----VSPMKN---QHYRRCAIVGNSGILLNSSCGP
Punnye_2       PTTNISENLYRLLPT----VSPMKN---QHYRRCAIVGNSGILLNSSCGP
Latcal_2       PTTNISENLYRLLPT----VSPMKN---QHHRRCAIVGNSGILLNSSCGP
Singra_2       ATTNISENLYHLLPT----VSPMKN---QHYKQCAIVGNSGILLNSSCGR
Auslim_2       PTTNVSENLYRLLPT----VSPLKH---QHHRSCAIVGNSGVLLNSSCGP
Monalb2        PTTNISENLYRLLPT----VSPMKN---QHX-----TGKK----NSSCGP
Ampoce_2B      PTTNISENLYRLLPT----VSPMKN---QHHRRCAIVGNSGILLNSSCGL
Chiham_2       PTTNISENLYRLLPT----VSPMKN---QHHKRCAIVGNSGILLNSSCGP
Gasacu_2       PTTNVSENLYRLLPT----ASPMKN---QHHRRCAIVGNSGILLNSSCGP
Gymacu_2       PTTNISENLYRLLPT----VSPMKN---QHHKRCAIVGNSGILLNSSCGP
Labber_2       PTTNISENLYRLLPT----ASPLKN---QHHRRCAIVGNSGILLNSSCGP
Cypvar_2       PTTNISENLYRLLPT----ASPLKN---QYHKRCAIVGNSGILLNSSCGP
Funhet_2       PTTNISEDLYRLLPT----ASPMKN---QYHRRCAIVGNSGILLNSSCGP
Larcro_2       PTTNISQNLYRLLPT----VSPMKN---QHHRRCAIVGNSGILLNSSCGS
Paroli_2       PTTNISENLYRLLPT----VSPMKN---QHHRRCAIVGNSGILLNSSCGH
Perflu_2       PTINISENLYRLLPT----VSPMKN---QHHRRCAIVGNSGILLNSSCGP
Serdum_2       PTTNISENLYRLLPT----VSPMKN---QHHRRCAIVGNSGILLNSSCGP
Stepar_2       PTTNISENLYRLLPT----VSPMKN---QHHRRCAIVGNSGILLNSSCGL
Notcor_2       PTTNISENLYRLLPT----VSPMKN---QHHKRCAIVGNSGILLNSSCGP
Treber_2       PTTNISENLYRLLPT----VSPMKN---QHHKRCAIVGNSGILLNSSCGP
Serlaldor_2    PTTNISENLYRLLPT----ISPMKN---QHHRRCAIVGNSGILLNSSCGP
Plaste_2       PTTNISENLYRLLPT----VSPMKN---QHHRRCAIVGNSGVLLNSSCGQ
Clabat_4       ATLNVSHTLHSLLPE----VSPLKN---KRFKTCAVVGNSGVLLKSGCGK
Ictpun_4A      ATLNVSHTLHSLLPD----VSPLKN---KRFKTCAVVGNSGVLLKSGCGK
Panhyp_4       ATLNVSHTLHSLLPE----VSPLKN---KRFKTCAVVGNSGVLLKSGCGK
Umbpyg_4       ATLNISHTLHSLLPD----VSPLKN---KRFRTCALVGNSGVLLRSGCGR
Esoluc_4       ATLNVSRTLHSLLPD----VSPLKN---KRFRTCAVVGNSGVLLNSGCGR
Plealt_4       ATLNVSQTLHSLLPE----VSPLKN---KRFNTCAVVGNSGVLLNSSCGK
Onckis_4B      ATLNVSHTLHSLLPD----VSPLKN---KRFRTCAVVGNSGVLLNSGCGK
Onckis_4A      ATLNVSHTLHSLLPD----VSPLKN---KRFRTCAVVGNSGVLLNSGCGK
Onctsh_4       ATLNVSHTLHSLLPD----VSPLKN---KRFRTCAVVGNSGVLLNSGCGK
Oncmyk_4       ATLNVSHTLHSLLPD----VSPLKN---KRFRTCAVVGNSGVLLNSGCGK
Corlav_4       ATLNISHTLHSLLPD----VSPLKN---KRFKTCAVVGNSGVLLNSGCGK
Astmex_4A      ATLNVSHTLHSLLPE----VSPLKN---KRFKTCAVVGNSGVLLKSGCGK
Pygnat_4       ATLNVSHTLHSLLPE----VSPLKN---KRFRTCAVVGNSGILLKSGCGK
Aloalo_4       ATLNVSQTLHKLLPE----VSPLKN---RRFKTCAVVGNSGVLLSSGCGQ
Cluhar_4       ATLNVSQTLHKLLPE----VSPLKN---RRFKTCAVVGNSGVLLSSGCGH
Denclu_4       ATLNISRTLHNLLPE----VSPLKN---RRFKTCAVVGNSGILLNSGCGR
Konpun4        ATLNVSQTLHKLLPE----VSPLKN---RRFKTCAVVGNSGVLLNSGCGQ
Sarpil_4       ATLNVSQTLHKLLPE----VSPLKN---RRFKTCAVVGNSGVLLSSGCGQ
Caraur_4       ATFNISQTLHSLLPE----VSPMKN---KTFKTCAVVGNSGVLLKSGCGK
Cteide_4       ATFNVSHTLHSLLPE----VSPLKN---KMFKTCAVVGNSGILLKSGCGK
Petmar_4       ATTNVSHSLHALLPT----APPLRG---GRYGSCAVVGNSGILEGSACGE
Cypcar_4       ATFNISQTLHSLLPE----VSPLKN---KTFKTCAVVGNSGVLLKSRCGK
Danrer_4       ATFSVSQTLHSLLPE----VSPLKN---KTFKTCAVVGNSGILLKSGCGK
Misang_4       ATFNVTHTLHSLLPE----VSPLKN---KKFKTCAVVGNSGILLKSRCGK
Angjap_4       ATLNVSRALHSLLPA----VSPMKN---RRFRTCAVVGNSGVLLNSGCGP
Cypcar_4A      ATFNISQTLHSLLPE----VSPLKN---KTFKTCAVVGNSGVLLKSRCGK
Angang_4       ATLNVSRALHSLLPA----VSPMKN---RRFRTCAVVGNSGVLLNSGCGR
Cypcar_4B      ATFNVSQTLHSLLPE----VSPLKN---KTFKTCAVVGNSGVLLKSGCGK
Sinans_4A      VTFNISQTLHSLLPE----VSPLKN---KTFKTCAIVGNSGILLKSRCGK
Sinans_4B      ATFNVSQTLHSLLPE----VSPLKN---KTFKTCSVVGNSGVLLKSGCGK
Singra_4A      ATFNISQTLHSLLPE----VSPLKN---KTFKTCAVVGNSGILLKSRCGK
Aptalb_4       AKLSVSHTLHSLLPE----VSPLKN---RRFKTCAVVGNSGVLLKSGCGK
Eigvir_4       ATLNISHTLHSLLPE----VSPLKN---KRFGTCAVVGNSGVLLKSGCGK
Eleele_4       ATLNVSHTLHSLLPE----VSPLKN---KRFRTCAVVGNSGVLLKSGCGK
Parhas_4       AKLNVSHTLHSLLPE----VSPLKN---RRFKTCAVVGNSGVLLKSGCGK
Sclfor_4       ATLSISQSLHSLLPD----VSPMKN---RRFKTCAVVGNSGILLKSGCGK
Gnapet4        ATLNVSHTLHSLLPD----TSPMKN---RRFNTCAVVGNSGVLLNSGCGK
Ostbic_4       ATLTISQTLHSLLPD----VSPLKN---KRFKTCAVVGNSGILLNSGCGK
Parkin_4       ATLNISHTLHSLLPD----ASPMKN---RRFNTCAVVGNSGVLLNSGCGK
Panbuc_4       ATFNVSHTLHSLLPD----VSPMKN---KRFKTCAVVGNSGVLLNSGCGR
Erpcal4        ATLNISHNLHSLLPE----VSPMKN---RRFKTCAVVGNSGILLGSGCGR
Salsal_4       ATLNVSHTLHSLLPD----VSPLKN---KRFRTCAVVGNSGVLLNSGCGK
Saltru_4       ATLNVSHTLHSLLPD----VSPLKN---KRFRTCAVVGNSGVLLNSGCGK
Salalp_4       ATLNVSHTLHSLLPD----VSPLKN---KRFRTCAVVGNSGVLLNSGCGK
Salfon_4       ATLNVSHTLHSLLPD----VSPLKN---KRFRTCAVVGNSGVLLNSGCGK
Thythy_4       ATLNVSHTLHSLLPD----VSPLKN---KRFKTCAVVGNSGVLLHSGCGK
```

```
Calmil_4       ATLNISRNLHSLLPE----VSPMKN---KRFNACAVVGNSGILLGSGCGK
Hetzeb_4       ATLNISHNLHSLLPE----VSPMKN---KRFSTCAVVGNSGILLGSGCGK
Rhityp_4       ATLNISHNLHRLLPE----VSPMKN---KRFSSCAVVGNSGILLGSGCGK
Scytor_4       ATLNISRNLHSLLPD----VSPMKN---KRFSTCAVVGNSGILLGSGCGK
Squaca_4       ATLNISQNLHSLLPE----VSPMKN---KRFSTCAVVGNSGILLGSGCGK
Amical_4       ATLNISHNLHSLLPE----VSPMKN---RRFKTCAVVGNSGILINSGCGR
Lepocu_4       ATLNISHNLHSLLPE----VSPMKN---RRFKTCAVVGNSGILINSGCGK
Agema_4        ATLNVSHTLHSLLPE----VSPLKN---KRFKTCAVVGNSGVLLKSGCGK
Anocar_4       ATLNISQDLHSLLPE----VSPMKN---RRFKMCAVVGNSGILLDSGCGK
Canlupfam_4    ATLNISQDLHSLLPE----VSPMKN---RRFKTCAVVGNSGILLDSECGK
Crigri_4       ATLNISHDLHSLLPE----VSPMKN---RRFKTCAVVGNSGILLDSGCGK
Galgal_4       ATLNISQDLHSLLPE----VSPMKN---RRFKTCAVVGNSGILLDSGCGK
Mesaur_4       ATLNISHDLHSLLPE----VSPMKN---RRFKTCAVVGNSGILLDSECGK
Homsap_4       ATLNISHDLHSLLPE----VSPMKN---RRFKTCAVVGNSGILLDSECGK
Notscuscu_4    ATLNISQDLHSLLPE----VSPMKN---RRFKTCAVVGNSGILLDSGCGK
Psetextex_4    ATLNISQDLHSLLPE----VSPMKN---RRFKTCAVVGNSGILLDSGCGK
Musmus_4       ATLNISHNLHSLLPE----VSPMKN---RRFKTCAVVGNSGILLDSGCGK
Pogvit_4       ATLNISQDLHRLLPE----VSPMKN---RRFKTCAVVGNSGILLDSGCGK
Promuc_4       ATLNISQDLHSLLPE----VSPMKN---RRFKTCSVVGNSGILLDSGCGK
Pantro_4       ATLNISHDLHSLLPE----VSPMKN---RRFKTCAVVGNSGILLDSECGK
Pytbiv_4       ATLNISQDLHSLLPE----VSPMKN---RRFKTCAVVGNSGILLDSGCGK
Ratnor_2       ATMNVSQNLYELLPR----TSPLKN---KHFQTCAIVGNSGVLLNSGCGQ
Ratnor_4       ATLNISHDLHSLLPE----VSPMKN---RRFKTCAVVGNSGILLDSGCGK
Siltro_2       ATMNISKNLYELLPR----TSPLKN---KHFKTCAIVGNSGILLNSGCGK
Siltro_4       ATLNISQNLHSLLPE----VSPMKS---RRFRTCAVVGNSGILLNSGCGK
Susscr_4       ATLNISQDLHSLLPE----VSPMKN---RRFKTCAVVGNSGILLDSECGK
Taegut_4       ATLNISQDLHSLLPE----VSPMKN---RRFKTCAVVGNSGILLNSGCGK
Termextri_4M   ATLNISQDLHSLLPE----VSPMKN---RRFKTCAVVGNSGILLDSACGK
Thasirsir_4    ATLNISQDLHRLLPE----VSPMKN---RRFKACAVVGNSGILLDSGCGK
Xenlae_4B      ATLNISQNLHSLLPE----VSPMKS---RRFRTCAVVGNSGILLNSGCGN
Xenlae_4A      ATLNISQNLHSLLPE----VSPMKS---RRFRTCAVVGNSGILLSSGCGK
               51
Latcal_3       QIEKFDYVFR--CNFAP-TEIFKKDVGRRTNMTTFN-PSILEKYYNNLLT
Perflu_3       QIEKFDFVFR--CNFAP-TEIFKKDVGRRTNMTTFN-PSILEKYYNNLLT
Serdum_3       QIEKFDFVFR--CNFAP-TEIFKKDVGRRTNMTTFN-PSILEKYYNNLLT
Stepar_3       QIEKFDFVFR--CNFAP-TEIFKKDVGRRTNMTTFN-PSILEKYYNNLLT
Tetcal_3       DIDKSDFVFR--CNFAP-TETFENDVGRKTNLTTFN-PSILEKYYNNLLT
Monalb_3       QIEKFDFVFR--CNFAP-TEVFKKDVGRRTNMTTFN-PSILEKYYNNLLT
Molmol_3       QIEKFDFVLR--CNFAP-TEIFKKDVGRRTNMTTFN-PSILEKYYNNLLT
Takrub_3       QIDRFDFVFR--CNFAP-TEIFKKDVGRRTNMTTFN-PSILEKYYNNLLT
Canlupfam_3    EIDKSDFVFR--CNFAP-TEAFQRDVGRKTNLTTFN-PSILEKYYNNLLT
Bostau_3       QIDKSDFVFR--CNFAP-TEAFQRDVGRKTNLTTFN-PSILEKYYNNLLT
Homsap_3       EIDKSDFVFR--CNFAP-SEAFQRDVGRKTNLTTFN-PSILEKYYNNLLT
Siltro_3       EIDKADFVFR--CNFAP-TEGFQKDVGRKTNLTTFN-PSILEKYYNNLLT
Anocar_3       EIDKYDFVFR--CNFAP-TEAFHKDVGRKTNITTFN-PSILEKYYNNLLT
Musmus_3       EIDKSDFVSR--CNFAP-TEAFHKDVGRKTNLTTFN-PSILEKYYNNLLT
Pantro_3       EIDKSDFVFR--CNFAP-TEAFQRDVGRKTNLTTFN-PSILEKYYNNLLT
Ratnor_3       EIDKSDFVFR--CNFAP-TEAFHKDVGKKTNLTTFN-PSILEKYYNNLLT
Galgal_3       EIDKSDFVFR--CNFAP-TEAFQKDVGRKTNLTTFN-PSILEKYYNNLLT
Lepocu_3       EIDKSDFIFR--CNFAP-TEIFHKDVGKKTNLTTFN-PSILEKYYNNLLT
Tetnig_3       EIDSLDFVFR--CNFAP-TELFKKDVGRRTNMTTFN-PSILEKYYNNLLT
Squaca_3       EIDKSDFVFR--CNFAP-TETFEKDVGKKTNLTTFN-PSILEKYYNNLLT
Perfla_3       QIEKFDFVFR--CNFAP-TEIFKKDVGRRTNMTTFN-PSILEKYYNNLLT
Salsal_3       EIEKFDFVFR--CNFAP-TEIFRRDVGRRTNLTTFN-PSILEKYYNNLLT
Serlaldor_3    QIEKFDFVFR--CNFAP-TEIFKKDVGRRTNMTTFN-PSILEKYYNNLLT
Treber_3       EIDQADFVFR--CNFAP-TEIYSKDVGKKTNLTTFN-PSILERYYNNLLT
Cynsem_3       QIEKFDFVFR--CNFAP-TEVFKKDVGRRTNMTTFN-PSILEKYYNNLLT
Paroli_3       EIDQADFVFR--CNFAP-TEVYSKDVGRKTNLTTFN-PSILERYYNNLLT
Paroli_3b      QIEKFDFVFR--CNFAP-TEIFKKDVGRRTNMTTFN-PSILEKYYNNLLT
Plaste_3       EIDQADFVFR--CNFAP-TEVYSKDVGRKTNLTTFN-PSILERYYNNLLT
Plaste_3b      QIEKFDFVFR--CNFAP-TELFKKDVGRQTNMTTFN-PSILEKYYNNLLT
Erpcal3        EIDKSDFIFR--CNFAP-TEIFQKDVGRKTNLTTFN-PSILEKYYNNLLT
Calmil_3b      SIDKSDFVFR--CNFAP-TEFFEKDVGRKTNLTTFN-PSILEKYYNNLMA
Hetzeb_3       DIDKSDFVFR--CNFAP-TESFEKDVGKKTNLTTFN-PSILEKYYNNLLT
Latcha_3       EIDQSDFVFR--CNFAP-TEIFHKDVGRKTNLTTFN-PSILEKYYNNLLT
Anates_3       QIEKFDFVFR--CNFAP-TEIFKKDVGRRTNLTTFN-PSILEKYYNNLLT
Ampcit3        QIEKYDFVFR--CNFAP-TEIFKKDVGRRTNMTTFN-PSILEKYYNNLLT
Auslim_3       HIERYDYVFR--CNFAP-TEIFRKDVGRRTNMTTFN-PSILEKYYNNLLT
Cypvar_3       QIEKYDFVFR--CNFAP-TEIFKKDVGRRTNMTTFN-PSILEKYYNNLLT
Krymar_3       QIERYDYVFR--CNFAP-TEIFKKDVGRRTNMTTFN-PSILEKYYNNLLT
Funhet_3       QIEKYDFVFR--CNFAP-TEIFKKDVGRRTNMTTFN-PSILEKYYNNLLT
Gamaff3        QIEKYDFVLR--CNFAP-TEIFKKDVGRRTNMTTFN-PSILEKYYNNLLT
Orylat_3       QIEKYDFVFR--CNFAP-TEIFKKDVGRRTNMTTFN-PSILEKYYNNLLT
Orenil_3       QIEKYDFVFR--CNFAP-TEIFKKDVGRRTNMTTFN-PSILEKYYNNLLT
Mayzeb_3       QIEKYDFVFR--CNFAP-TEIFKKDVGRRTNMTTFN-PSILEKYYNNLLT
Punnye_3       QIEKYDFVFR--CNFAP-TEIFKKDVGRRTNMTTFN-PSILEKYYNNLLT
Punnye_3a      QIEKYDFVFR--CNFAP-TEIFKKDVGRRTNMTTFN-PSILEKYYNNLLT
```

```
Neobri_3       QIEKYDFVFR--CNFAP-TEIFKKDVGRRTNMTTFN-PSILEKYYNNLLT
Misang_3       EIDKYDFVFR--CNFAP-TEVFRKDVGRRTNLTTFN-PSILEKYYNNLLT
Sinrhi3        QIDSYDFVFR--CNFAP-TEVFRRDVGRRTNLTTFN-PSILEKYYNNLLT
Singra_3       QIDSYDFVFR--CNFAP-TEAFRRDVGRRTNLTTFN-PSILEKYYNNLLT
Hapbur_3       QIEKYDFVFR--CNFAP-TEIFKKDVGRRTNMTTFN-PSILEKYYNNLLT
Astcal_3       QIEKYDFVFR--CNFAP-TEIFKKDVGRRTNMTTFN-PSILEKYYNNLLT
Notfur_3       QIEKYDFVFR--CNFAP-TEIFKKDVGRRTNMTTFN-PSILEKYYNNLLT
Cypcar_3       QIDSYDYVFR--CNFAP-TEVFRRDVGRRTNLTTFN-PSILEKYYNNLLT
Poefor3        QIEKYDFVLR--CNFAP-TEIFKKDVGRRTNMTTFN-PSILEKYYNNLLT
Poelat_3       QIEKYDFVLR--CNFAP-TEIFKKDVGRRTNMTTFN-PSILEKYYNNLLT
Poeret_3       QIEKYDFVLR--CNFAP-TEIFKKDVGRRTNMTTFN-PSILEKYYNNLLT
Angang_3       EIDRSDFVFR--CNFAP-TEIFHRDVGRRTNLTTFN-PSILEKYYNNLLT
Xipmac_3       QIEKYDFVLR--CNFAP-TEIFKKDVGRRTNMTTFN-PSILEKYYNNLLT
Masarm_3       QIEKFDFVFR--CNFAP-TEIFKKDVGRRTNMTTFN-PSILEKYYNNLLT
Permag_3       QIDSFDFVFR--CNFAP-TEVFKKDVGRRTNMTTFN-PSILEKYYNNLLT
Gnapet3        QIDKSDFVFR--CNFAP-TEVFHKDVGHKTNLTTFN-PSILEKYYNNLLT
Ostbic_3       RIDSSDFVFR--CNFAP-TELFRKDVGRKTNLTTFN-PSILEKYYNNLLT
Gadmor_3       QIEKFDYVFR--CNFAP-TEIFKKDVGRHTNMTTFN-PSILEKYYNNLLT
Sclfor_3       RIDGSDFVFR--CNFAP-TELFHRDVGRKTNLSTFN-PSILEKYYNNLLT
Acapol3        QIEKFDFVFR--CNFAP-TEIFKKDVGRRTNMTTFN-PSILEKYYNNLLT
Ampoce_3       QIEKFDFVFR--CNFAP-TEIFKKDVGRRTNMTTFN-PSILEKYYNNLLT
Sclfor_3b      EIDRFDFVFR--CNFAP-TELFHKDVGHKTNITTFN-PSILEKYYNNLLT
Ostbic_3b      EIDRFDFVFR--CNFAP-TELFHKDVGHKTNITTFN-PSILEKYYNNLLT
Parkin_3       QIDESDFVFR--CNFAP-TEVFHKDVGHKTNLTTFN-PSILEKYYNNLLT
Ampper3        QIEKFDFVFR--CNFAP-TEIFKKDVGRRTNMTTFN-PSILEKYYNNLLT
Oncmyk_3       EIEKFDFVFR--CNFAP-TEIFHRDVGRRTNLTTFN-PSILEKYYNNLLT
Gasacu_3       QIEKFDFVFR--CNFAP-TEIFKKDVGRRTNMTTFN-PSILEKYYNNLLT
Labber_3       QIEKFDYVFR--CNFAP-TEVFKKDVGRQTNMTTFN-PSILEKYYNNLLT
Panbuc_3       EIDRYDFVFR--CNFAP-TEAFRKDVGRKTNLTTFN-PSILEKYYNNLLT
Parhas3        AIDKYDFVFR--CNFAP-TEAFRKDVGRRTNLTTFN-PSILEKYYNNLLT
Cluhar_3       EIDKFDFVFR--CNFAP-TEVFRKDVGRHTNLTTFN-PSILEKYYNNLLT
Aloalo_3       EIDKFDFVFR--CNFAP-TEVFRKDVGRHTNLTTFN-PSILEKYYNNLLT
Panhyp_3       AIDAYDFVFR--CNFAP-TEVFRKDVGRRTNLTTFN-PSILEKYYNNLLT
Eleele_3       TIDQYDFVFR--CNFAP-TEVFRKDVGRRTNLTTFN-PSILEKYYNNLLT
Denclu_3       DIDKYDFIFR--CNFAP-TELFRKDVGRRTNLTTFN-PSILEKYYNNLLT
Sarpil_3       EIDKFDYVFR--CNFAP-TEVFRKDVGRRTNLTTFN-PSILEKYYNNLLT
Eigvir_3       AIDKYDFVFR--CNFAP-TEVFRKDVGRRTNLTTFN-PSILEKYYNNLLT
Aptalb_3       AIDRYDFVFR--CNFAP-TEAFRKDVGRRTNLTTFN-PSILEKYYNNLLT
Sinans_3       QIDSYDFVFR--CNFAP-TEVFRRDVGRRTNLTTFN-PSILEKYYNNLLT
Pygnat_3       TIDKYDFVFR--CNFAP-TEVFRRDVGRRTNLTTFN-PSILEKYYNNLLT
Astmex_3       TIDKYDFVFR--CNFAP-TEVFRRDVGRRTNLTTFN-PSILEKYYNNLLT
Caraur_3       QIDSYDFVFR--CNFAP-TEIFRRDVGRRTNLTTFN-PSILEKYYNNLLT
Danrer_3       EIDKYDFVFR--CNFAP-TEVFRRDVGRRTNLTTFN-PSILEKYYNNLLT
Ictpun_3       AIDAYDFVFR--CNFAP-TEVFRRDVGRRTNLTTFN-PSILEKYYNNLLT
Plealt_3       EIEKFDFVFR--CNFAP-TEIFRRDVGRRTNMTTFN-PSILEKYYNNLLT
Plealt_9       EIDKADFIFR--CNFAP-TEVFYKDVGRKTNMTTFN-PSILEHYYNNLLT
Gasacu_9       EIDQADFVFR--CNFAP-TEVYSKDVGKKTNLTTFN-PSILERYYNNLLT
Parkin_9       EIDKFNFIFR--CNFAP-TEIYHRDVGRKTNMTTFN-PSILERYYNNLLT
Acapol_9       EIDEADFVFR--CNFAP-TEVYSKDVGKKTNLTTFN-PSILERYYNNLLT
Larcro_9       EIDQADFVFR--CNFAP-TEVYSKDVGRKTNLTTFN-PSILERYYNNLLT
Notcor_9       EIDQADFVFR--CNFAP-TEIYSKDVGKKTNLTTFN-PSILERYYNNLLT
Latcal_9       EIDQADFVFR--CNFAP-TEVYSKDVGRKTNLTTFN-PSILERYYNNLLT
Serlaldor_9    EIDQADFVFR--CNFAP-TEVYSKDVGRKTNLTTFN-PSILERYYNNLLT
Monalb_9       EIDQADFVFR--CNFAP-TEVYFKDVGRKTNLTTFN-PSILERYYNNLLT
Serdum_9       EIDQADFVFR--CNFAP-TEVYSKDVGRKTNLTTFN-PSILERYYNNLLT
Perflu_9       EIDQADFVFR--CNFAP-TEFYSKDVGKKTNLTTFN-PSILERYYNNLLT
Perfla_9       EIDQADFVFR--CNFAP-TEFYSKDVGKKTNLTTFN-PSILERYYNNLLT
Stepar_9       EIDQADFVFR--CNFAP-TEVYSKDVGKKTNLTTFN-PSILERYYNNLLT
Molmol_9       EIDQADFVFR--CNFAP-TDIYSKDVGRKTNLTTFN-PSILERYYNNLLT
Hipcom_9       EIDRADFVFR--CNFAP-TEVYAKDVGRKTNLTTFN-PSILERYYNNLLT
Takrub_9 B     NIDQADFVFR--CNFAP-TEVYSKDVGRKTNMTTFN-PSILERYYNNLLT
Takrub_9       NIDQADFVFR--CNFAP-TEVYSKDVGRKTNMTTFN-PSILERYYNNLLT
Cynsem_9       EIDQADFVFR--CNFAP-TEVYSKDVGKKTNLTTFN-PSILERYYNNLLT
Scomax_9       EIDQADFVFR--CNFAP-TEVYSKDVGRKTNLTTFN-PSILERYYNNLLT
Corlav_9       EIDSADFIFR--CNFAP-TDSYYKDVGRKTNLTTFN-PSILERYYNNLLT
Oncmyk_9       EIDSADFIFR--CNFAP-TDSYYKDVGRKTNLTTFN-PSILERYYNNLLT
Salsal_9       EIDSSDFIFR--CNFAP-TDSYYKDVGRKTNLTTFN-PSILERYYNNLLT
Onctsh_9       EIDSADFIFR--CNFAP-TDSYYKDVGRKTNLTTFN-PSILERYYNNLLT
Salalp_9       EIDSADFIFR--CNFAP-TDSYYKDVGRKTNLTTFN-PSILERYYNNLLT
Ampoce_9       EIDEADFVFR--CNFAP-TEVYSKDVGKKTNLTTFN-PSILERYYNNLLT
Ampper9        EIDEADFVFR--CNFAP-TEVYSKDVGKKTNLTTFN-PSILERYYNNLLT
Labber_9       EIDQADFVFR--CNFAP-TDVYSKDVGKKTNLTTFN-PSILERYYNNLLT
Umbpyg_3B      EIEKFDFVFR--CNFAP-TEIFRRDVGRRTNLTTFN-PSILEKYYNNLLT
Esoluc_3b      EIEKFDFVFR--CNFAP-TEVFRRDVGRRTNLTTFN-PSILEKYYNNLLT
Gadmor_9       EIDQADFVFR--CNFAP-TEVYYKDVGRKTNMTTFN-PSILERYYNNLLT
Umbpyg_9       EIDSADFIFR--CNFAP-IDSYYKDVGRKTNLTTFN-PSILERYYNNLLT
Esoluc_9       EIDSADFIFR--CNFAP-IESYYKDVGRKTNLTTFN-PSILERYYNNLLT
```

```
Permag_9       EIDQADFVFR--CNFAP-TEVYSKDVGKKTNLTTFN-PSILERYYNNLLT
Bolpec_9       EIDQADFVFR--CNFAP-TEVYSKDVGKKTNLTTFN-PSILERYYNNLLT
Poefor9        EIDQADFVFR--CNFAP-TEVYFKDVGKKTNLTTFN-PSILERYYNNLLT
Masarm_9       EIDQADFVFR--CNFAP-TEVYSKDVGKKTNLTTFN-PSILERYYNNLLT
Angjap_9       EIDRSDFVFR--CNFAP-TEVFHRDVGRRTNLTTFN-PSILEKYYNNLLT
Poelat_9       EIDQADFVFR--CNFAP-TEVYFKDVGKKTNLTTFN-PSILERYYNNLLT
Poeret_9       VIDQADFVFR--CNFAP-TEFYFKDVGKKTNLTTFN-PSILERYYNNLLT
Xipmac_9       EIDQADFVFR--CNFAP-TEIYFKDVGKKTNLTTFN-PSILERYYNNLLT
Hapbur_9       EIDQADFVFR--CNFAP-TDIYSKDVGKKTNLTTFN-PSILERYYNNLLT
Orylat_9       EIDQADFVFR--CNFAP-TDVYSKDVGRKTNMTTFN-PSILERYYNNLLT
Gamaff_9       EIDQADFVFR--CNFAP-TEVYFKDVGKKTNLTTFN-PSILERYYNNLLT
Neobri_9       EIDQADFVFR--CNFAP-TDIYSKDVGKKTNLTTFN-PSILERYYNNLLT
Funhet_9       EIDQADFVFR--CNFAP-TEVYSKDVGKKTNLTTFN-PSILERYYNNLLT
Punnye_9       EIDQADFVFR--CNFAP-TDIYSKDVGKKTNLTTFN-PSILERYYNNLLT
Cypvar_9       EIDQADFVFR--CNFAP-TDVYSKDVGKKTNLTTFN-PSILERYYNNLLT
Krymar_9       EIDQADFVFR--CNFAP-TEVYSKDVGKKTNLTTFN-PSILERYYNNLLT
Notfur_9       EIDQADFVFR--CNFAP-TEIYSKDVGKKTNMTTFN-PSILERYYNNLLT
Notpie_9       EIDQADFVFR--CNFAP-TEIYSKDVGKKTNMTTFN-PSILERYYNNLLT
Orenil_9       EIDQADFVFR--CNFAP-TDIYSKDVGKKTNLTTFN-PSILERYYNNLLT
Auslim_9       EIDQADFVFR--CNFAP-TEIYSKDVGRKTNLTTFN-PSILERYYNNLLT
Anates_9       EIDQADFVFR--CNFAP-TDVYSKDVGKKTNLTTFN-PSILERYYNNLLT
Astcal_9       EIDQADFVFR--CNFAP-TDIYSKDVGKKTNLTTFN-PSILERYYNNLLT
Mayzeb_9       EIDQADFVFR--CNFAP-TDIYSKDVGKKTNLTTFN-PSILERYYNNLLT
Orymel_9       EIDQADFVFR--CNFAP-TDVYSKDVGRKTNMTTFN-PSILERYYNNLLT
Ampcit9        EIDQADFVFR--CNFAP-TDIYSKDVGKKTNLTTFN-PSILERYYNNLLT
Macfas_2       EIDAHSFVIR--CNLAP-VQEYARDVGLKTDLVTMN-PSVIQRAFEDLVN
Macmul_2       EIDAHSFVIR--CNLAP-VQEYARDVGLKTDLVTMN-PSVIQRAFEDLVN
Macnem_2       EIDAHSFVIR--CNLAP-VQEYARDVGLKTDLVTMN-PSVIQRAFEDLVN
Musmus_2       EIDTHSFVIR--CNRAP-VQEYARDVGLKTDLVTMN-PSVIQRAFEDLVN
Ponabe_2       EIDAHSFVIR--CNLAP-VQEYARDVGLKTDLVTMN-PSVIQRAFEDLVN
Papanu_2       EIDAHSFVIR--CNLAP-VQEYARDVGLKTDLVTMN-PSVIQRAFEDLVN
Nomleu_2       EIDAHSFVIR--CNLAP-VQEYARDVGLKTDLVTMN-PSVIQRAFEDLVN
Pantro_2       EIDAHSFVIR--CNLAP-VQEYARDVGLKTDLVTMN-PSVIQRAFEDLVN
Latcha_2       EIDDHQFVIR--CNLAP-VQEYASDVGTKTDFVTMN-PSVIQRAFEDLVN
Gnapet2        EIDSHDFVIR--CNLAP-VEEYERDVGLRSSLVTMN-PSVVQRAFRDLAS
Panbuc_2b      EIDACDYVIR--CNLAP-VQEYVTDVGQRTSMVTMN-PSVVQRAFQDLAS
Ostbic_2       EIDSNDFVIR--CNLAP-VDEYEQDVGRRTSLVTMN-PSVVQRAFQDLAS
Sinrhi2        EIDSHDFVIR--CNLAP-VEEYATDVGLRTSLVTMN-PSVVQRAFQDLNS
Hetzeb_2       EIDAHEFVIR--CNLAP-VEEYAHDVGLKTDLVTMN-PSVVQRAFEDLKN
Hipcom_2       QIDSHDFVIR--CNLAP-VEEYADDVGHRTDLVTMN-PSVVQRAFRDLVD
Prigla_2       EIDTHEFVIR--CNLAP-VEEYAQDVGLKTDLVTMN-PSVVQRAFEDLKN
Scytor_2       EIDTHEFVIR--CNLAP-VEEYAQDVGLKTDLVTMN-PSVVQRAFEDLKN
Tetcal_2       EIDAHEFVIR--CNLAP-VEEYVQDVGMKTDLVTMN-PSVVQRAFEDLKN
Rhityp_2       EIDDHQFVIR--CNLAP-VEEYAQDVGLKTDLVTMN-PSVVQRAFEDLKN
Parkin_2b      EIDSHDFVIR--CNLAP-VQEFELDVGLRTGLVTMN-PSVVQRAFQDLGS
Angang_2       QIDSHDFVIR--CNLAP-VEEYAADVGQRTSLVTMN-PSVVERAFRDLSS
Pygnat_2       EIDSHDFVIR--CNLAP-VEEYTNDVGLRTSLVTMN-PSVVQRAFQDLNS
Parkin_2       EIDSHDFVIR--CNLAP-VEEYEHDVGLRSSLVTMN-PSVVQRAFRDLAS
Gorgor_2       EIDAHSFVIR--CNLAP-VQEYARDVGPKTDLVTMN-PSVIQRAFEDLVN
Thegel_2       EIDAHSFVIR--CNLAP-VQEYARDVGLKTDLVTMN-PSVIQRAFEDLVN
Tupchi_2       EIDTHSFVIR--CNLAP-VQEYAQDVGLKTDLVTMN-PSVIQRAFEDLVN
Galgal_2       EIDTHSFVIR--CNLAP-VQEYSQDVGTKTDLVTMN-PSVIQRAFEDLMN
Eleele_2       EIDSHDFIIR--CNLAP-IEHYVKDVGVRTGLVTMN-PSVVERVFQDLSS
Danrer_2       EIDSHDFVIR--CNLAP-VEEYAADVGLRTSLVTMN-PSVVQRAFQDLNS
Canlupfam_2    EIDTHSFVIR--CNLAP-VQEYGRDVGLKTDLVTMN-PSVIQRAFEDLVN
Aloalo_2       EIDSYDFVIR--CNLAP-VDEFTADVGHRTNLVTMN-PSVVQRAFQDLSS
Astmex_2       EIDSHDFVIR--CNLAP-VEEYAEDVGLRSSLVTMN-PSVVQRAFQDLSS
Sinans_2       EIDSHDFVIR--CNLAP-VEEYATDVGLRTSLVTMN-PSVVQRAFQDLNS
Sclfor_2       EIDSNDFVIR--CNLAP-VEEYEQDVGHRTSLVTMN-PSVVQRAFQDLAS
Ceraty_2       EIDAHSFVIR--CNLAP-VQEYARDVGLKTDLVTMN-PSVIQRAFEDLVN
Anocar_2       EIDAHSFVIR--CNLAP-VQEYSRDVGTKMDLVTMN-PSVIQRAFEDLVN
Anates_2       EIDSHDFIIR--CNLAP-VEEYSRDVGWQTNLVTMN-PSVVQRAFQDLVS
Ampbic_2       EIDSHDFVIR--CNLAP-VEDYSRDVGRQTDLVTMN-PSVVQRAFQDLVS
Sarpil_2       EIDSYDFVIR--CNLAP-VDEFSADVGHRTSLVTMN-PSVVQRAFQDLSS
Xenlae_2       EIDSHDFVIR--CNLAP-VEEYAKDVGTKTNLVTMN-PSVVQRAFEDLVN
Cluhar_2       EIDAHDFVIR--CNLAP-VNEFSADVGHRTNLVTMN-PSVVQRAFQDLSS
Cypcar_2       EIDSHDFVIR--CNLAP-VEEYATDVGLRTSLVTMN-PSVVQRAFQDLNS
Denclu_2       EIDSHDFVIR--CNLAP-VEEYAADVGHRTDLVTMN-PSVVQRAFQDLAN
Notpie_2       EIDSHDFVIR--CNLAP-VEEYSQDVGWRTNLVTMN-PSVVQRAFQDLVT
Erpcal2        EIDTHEFVIR--CNLAP-VQEFASDVGIRTNLVTMN-PSVVQRAFEDLAN
Amical_2       EIDSHDFVIR--CNLAP-VEEYADDVGLRTSLVTMN-PSVVQRAFQDLVS
Lepocu_2       EIDSHDFVIR--CNLAP-VEEYRADVGRRTSLVTMN-PSVVQRAFQDLAS
Homsap_2       EIDAHSFVIR--CNLAP-VQEYARDVGLKTDLVTMN-PSVIQRAFEDLVN
Bostau_2       EIDTHSFVIR--CNLAP-VQEYARDVGLKTDLVTMN-PSVVQRAFEDLVN
Caraur_2       EIDSHDFVIR--CNLAP-VEEYATDVGLRTSLVTMN-PSVVQRAFQDLNS
Thythy_2B      EIDSHDFVIR--CNLAP-VEEYAGDVGRHTNLVTMN-PSVVQRAFQDLAS
Onctsh_2B      EIDSYDFVIR--CNLAP-VEEYAGDVGRRTNLVTMN-PSVVQRAFQDLAS
```

```
Salsal_2B      EIDSYDFVIR--CNLAP-VEEYAGDVGRRTNLVTMN-PSVVQRAFQDLAS
Oncmyk_2B      EIDSYDFVIR--CNLAP-VEEYAGDVGRRTNLVTMN-PSVVQRAFQDLAS
Onckis_2B      EIDSYDFVIR--CNLAP-VEEYAGDVGRRTNLVTMN-PSVVQRAFQDLAS
Salsal_2A      EIDSHDFVIR--CNLAP-VEEYAGDVGQRTNLVTMN-PSVVQRAFHDLAS
Thythy_2A      EIDSHDFVIR--CNLAP-VEEYAGDVGRRTNLVTMN-PSVVQRAFHDLAS
Salalp_2A      EIDSHDFVIR--CNLAP-VEEYAGDVGRRTNLVTMN-PSVVQRAFHDLAS
Corlav_2A      EIDSHDFVIR--CNLAP-VEEYAGDVGRRTNLVTMN-PSVVQRAFHDLAS
Cormar_2A      EIDSHDFVIR--CNLAP-VEEYAGDVGRRTNLVTMN-PSVVQRAFHDLAS
Onctsh_2A      EIDSHDFVIR--CNLAP-VEEYAGDVGRRTNLVTMN-PSVVQRAFHDLAS
Oncmyk_2A      EIDSHDFVIR--CNLAP-VEEYAGDVGRRTNLVTMN-PSVVQRAFHDLAS
Neobri_2       EIDSHDFVIR--CNLAP-VEEYFQDVGWRTNLVTMN-PSVVQRAFQDLVT
Takrub_2       EIDSHDFVIR--CNLAP-VEDYYKDVGWRTNLVTMN-PSVVQRAFRDLAS
Tetnig_2       EIDSHDFVIR--CNLAP-VEDFHNDVGWRTNLVTMN-PSVVQRAFRDLAS
Orenil_2       EIDSHDFVIR--CNLAP-VEEYFQDVGWRTNLVTMN-PSVVQRAFQDLVT
Orylat_2       EIDAHDFVIR--CNLAP-VDEYSQDVGRQTNLVTMN-PSVVQRAFQDLVS
Krymar_2       EIDSHDFVIR--CNLAP-VEEYSRDVGRRTDLVTMN-PSVVQRAFQDLLT
Notfur_2       EIDSHDFVIR--CNLAP-VEEYSQDVGWRTNLVTMN-PSVVQRAFQDLVT
Poelat_2       EIDSHDFVIR--CNLAP-VEEYSQDVGRRTNLVTMN-PSVVQRAFQDLVS
Gadmor_2       EIDSYDFVIR--CNLAP-VKEYSVDVGRRTNLVTMN-PSVVQRAFQDLAS
Xipmac_2       EIDSHDFVIR--CNLAP-VEEYSQDVGRRTNLVTMN-PSVVQRAFQDLVS
Poeret_2       EIDSHDFVIR--CNLAP-VEEYSQDVGRRTNLVTMN-PSVVQRAFQDLVS
Astcal_2       EIDSHDFVIR--CNLAP-VEEYFQDVGWRTNLVTMN-PSVVQRAFQDLVT
Hapbur_2       EIDSHDFVIR--CNLAP-VEEYFQDVGWRTNLVTMN-PSVVQRAFQDLVT
Acapol_2B      EIDSHDFVIR--CNLAP-VDDYSRDVGRRTNLVTMN-PSVVQRAFQDLVS
Ampoce_2A      EIDSHDFVIR--CNLAP-VEDYSRDVGRQTNLVTMN-PSVVQRAFQDLVN
Acapol_2A      EIDSHDFVIR--CNLAP-VDDYSRDVGRRTNLVTMN-PSVVQRAFQDLVS
Mayzeb_2       EIDSHDFVIR--CNLAP-VEEYFQDVGWRTNLVTMN-PSVVQRAFQDLVT
Punnye_2       EIDSHDFVIR--CNLAP-VEEYFQDVGWRTNLVTMN-PSVVQRAFQDLVT
Latcal_2       EIDSHDFVIR--CNLAP-VEEYSRDVGWRTNLVTMN-PSVVQRAFQDLVS
Singra_2       EIDSHDFVIR--CNLAP-VEEYATDVGLRTSLVTMN-PSVVQRAFQDLNS
Auslim_2       EIDSHDFVIR--CNLAP-VEEYSEDVGRRTNLVTMN-PSVVQRAFQDLVT
Monalb2        EIDSHDFVIR--CNLAP-VEEYSRDVGRRTNLVTMN-PSVVQRAFQDLVT
Ampoce_2B      EIDSHDFVIR--CNLAP-VEDYSRDVGRQTNLVTMN-PSVVQRAFQDLVN
Chiham_2       DIDSHDFVIR--CNLAP-VDEFSQDVGRRTNLVTMN-PSVVQRAFQDLVS
Gasacu_2       DIDSHDFVIR--CNLAP-VEDYSLDVGRRTNLVTMN-PSVVQRAFQDLVS
Gymacu_2       DIDSHDFVIR--CNLAP-VDEFSQDVGRRTNLVTMN-PSVVQRAFQDLVS
Labber_2       EIDSHDFVIR--CNLAP-VADYYQDVGWRTNLVTMN-PSVVQRAFQDLVS
Cypvar_2       EIDSHDFVIR--CNLAP-VEEYSQDVGLRTNLVTMN-PSVVQRAFQDLIS
Funhet_2       EIDSHDFVIR--CNLAP-VEEYSQDVGRRTNLVTMN-PSVVQRAFQDLVS
Larcro_2       EIDSHDFVIR--CNLAP-VEEYYRDVGWRTNLVTMN-PSVVQRAFQDLVS
Paroli_2       EIDSHDFVIR--CNLAP-VEEFSWDVGRRTNLVTMN-PSVVQRAFQDLVS
Perflu_2       DIDSHDFVIR--CNLAP-VEEYFQDVGWRTNLVTMN-PSVVQRAFQDLVS
Serdum_2       EIDSHDFVIR--CNLAP-VEEYSRDVGRRTNLVTMN-PSVVQRAFMDLVS
Stepar_2       EIDSHDFVIR--CNLAP-VEDYSRDVGRRTNLVTMN-PSVVQRAFQDLVS
Notcor_2       DIDSHDFVIR--CNLAP-VDEFSQDVGRRTNLVTMN-PSVVQRAFQDLVS
Treber_2       DIDSHDFVIR--CNLAP-VDEFSQDVGRRTNLVTMN-PSVVQRAFQDLVS
Serlaldor_2    EIDSHDFVIR--CNLAP-VEEYSRDVGRRTNLVTMN-PSVVQRAFMDLVS
Plaste_2       EIDSHDFVIR--CNLAP-VEEYSWDVGTRTSLVTMN-PSVVQRAFQDLVS
Clabat_4       EIDIHDFVIR--CNLAP-LAEFAEDVGLRSDFTTMN-PSVIQRVYGGLSN
Ictpun_4A      EIDSHDFVIR--CNLAP-LAEFADDVGLRSDFTTMN-PSVIQRVYGGLLN
Panhyp_4       EIDSHDFVIR--CNLAP-LAEFAEDVGLRSDFTTMN-PSVIQRVYGGLLN
Umbpyg_4       EIDSHDFVIR--CNLAP-LAEFSEDVGLRSDFTTMN-PSVIQRAYGGLKN
Esoluc_4       EIDSHDFVIR--CNLAP-LAQFAEDVGLRSDFTTMN-PSVIQRAYGGLRN
Plealt_4       EIDSHDFVIR--CNLAP-LSEYADDVGVRSDFTTMN-PSVIQRAYGGLKN
Onckis_4B      EIDSHDFVIR--CNLPP-LSEFAEDVGLRSDFTTMN-PSVIQRAYGGLKN
Onckis_4A      EIDSHDFVIR--CNLPP-LSEFAEDVGLRSDFTTMN-PSVIQRAYGGLKN
Onctsh_4       EIDSHDFVIR--CNLPP-LSEFAEDVGLRSDFTTMN-PSVIQRAYGGLKN
Oncmyk_4       EIDSHDFVIR--CNLPP-LSEFAEDVGLRSDFTTMN-PSVIQRAYGGLKN
Corlav_4       EIDRHDFVIR--CNLAP-LAEFAEDVGLRSDFTTMN-PSVIQRVYGGLKN
Astmex_4A      EIDGHDFVIR--CNLAP-LAEFAEDVGLKSDFTTMN-PSVIQRVYGGLRN
Pygnat_4       EIDSHDFVIR--CNLAP-LVEFAEDVGLKSDFTTMN-PSVIQRVYGGLRN
Aloalo_4       EIDSHDFVIR--CNLAP-LAEFAADVGLRSDFTTMN-PSVIQRAYGGLRN
Cluhar_4       EIDSHDFVIR--CNLAP-QAEFAADVGLRSDFTTMN-PSVIQRAYGGLRN
Denclu_4       EIDSHDFIIR--CNLAP-LKEFAEDVGLRSDFTTMN-PSVIHRVYGGLQK
Konpun4        EIDSHDFVIR--CNLAP-LVEFAADVGLRSDFTTMN-PSVIQRAYSGLRN
Sarpil_4       EIDSHDFVIR--CNLAP-LAEFADDVGLRSDFTTMN-PSVIQRAYGGLRN
Caraur_4       EIDSHGFVIR--CNLAP-LVEFADDVGLRSDFTTMN-PSVIPRVYGALRD
Cteide_4       EIDNHDFVIR--CNLAP-LKDFADDVGLRSDFTTMN-PSVIQRVYKGLRD
Petmar_4       EIDQHDFVIR--CNLAP-VEGYERDVGSRVDFVTMN-PSVVERTYGGLRT
Cypcar_4       EIDSHGFVIR--CNLAP-LEEFADDVGLRSDFTTMN-PSVIPRVYGGLQD
Danrer_4       EIDNHSFVIR--CNLAP-LEGFADDVGLRSDFTTMN-PSVIQRVYGGLRE
Misang_4       EIDNHDFVIR--CNLAP-LEEFSDDVGLKSDFTTMN-PSVIQRVYGGLHN
Angjap_4       EIDGHDFVIR--CNLAP-LEEFAEDVGLRSDFTTMN-PSVIQRAYGGLRN
Cypcar_4A      EIDSHGFVIR--CNLAP-LEEFADDVGLRSDFTTMN-PSVIPRVYGGLQD
Angang_4       EIDGHDFVIR--CNLAP-LEEFAEDVGLRSDFTTMN-PSVIQRAYGGLRN
Cypcar_4B      EIDSHGFVIR--CNLAP-LEDFADDVGLRSDFTTMN-PSVIPRVYGGLRD
Sinans_4A      EIDSHGFVIR--CNLAP-LEEFADDVGLRSDFTTMN-PSVIPRVFGGLRD
```

```
Sinans_4B      EIDSHGFVIR--CNLAP-LEEFADDVGSRSDFTTMN-PSVIPRVFGGLRD
Singra_4A      EIDSHGFVIR--CNLAP-LEEFADDVGLRSDFTTMN-PSVIPRVFGGLQD
Aptalb_4       EIDSHDFVIR--CNLAP-LAEFIKDVGLKSDFTTMN-PSVIQRMYGGLRN
Eigvir_4       EIDDHDFVIR--CNLAP-LAEFIKDVGWKSDFTTMN-PSVIQRVYHGLRN
Eleele_4       EIDSHDFVIR--CNLAP-LAKFAEDVGLKSDFTTMN-PSVIQRMYGGLRN
Parhas_4       EIDSHDFVIR--CNLAP-LAEFIKDVGLKSDFTTMN-PSVIQRMYGGLRN
Sclfor_4       EIDNHNFVIR--CNLAP-LVEFSEDVGLQSDFTTMN-PSVIQRAFGGLKN
Gnapet4        EIDSNDFVIR--CNLAP-LANFSEDVGLRSDFTTMN-PSVIQRAFGGLRN
Ostbic_4       EIDSHDFVIR--CNLAP-LVEFSEDVGLRSDFTTMN-PSVIQRVFGSLKN
Parkin_4       EIDSNDFVIR--CNLAP-LANFSEDVGLRSDFTTMN-PSVIQRAFGGLRN
Panbuc_4       EIDSHDFIIR--CNLAP-VTEFAVDVGQRSHFITMN-PSVIQRAYGSLRN
Erpcal4        EIDNHDFVIR--CNLAP-VVEFAEDVGNRSDFITMN-PSVIQRAFGGFQN
Salsal_4       EIDSHDFVIR--CNLPP-LSEFAEDVGLRSDFTTMN-PSVIQRAYGGLKN
Saltru_4       EIDSHDFVIR--CNLPP-LSEFAEDVGLRSDFTTMN-PSVIQRAYGGLKN
Salalp_4       EIDSHDFVIR--CNLPP-LSEFAEDVGLRSDFTTMN-PSVIQRVYGGLKN
Salfon_4       EIDSHDFVIR--CNLPP-LSEFAEDVGLRSDFTTMN-PSVIQRAYGGLKN
Thythy_4       EIDSHDFVIR--CNLAP-LADFAEDVGLRSDFTTMN-PSVIQRAYGGLKN
Calmil_4       EIDSHEFVIR--CNLAP-LVEYTDDVGSKSDFVTMN-PSVVQRAFGSLQN
Hetzeb_4       EIDSHEFVIR--CNLAP-LMEYTDDVGLKSDFVTMN-PSVVQRAFGSLRS
Rhityp_4       EIDSHEFVIR--CNLAP-LVEYADDVGLKSDFVTMN-PSVVQRAFGSLRS
Scytor_4       EIDSHEFVIR--CNLAP-LVEYADDVGLKSDFVTMN-PSVVQRAFGSLRS
Squaca_4       EIDSHEFVIR--CNLAP-LVEYADDVGLKSDFVTMN-PSVVQRAFGSLQS
Amical_4       EIDSHDFVIR--CNLAP-LAEFSEDVGLRSDFITMN-PSVIQRAFGGFKN
Lepocu_4       EIDNHDFVIR--CNLAP-LAEFSEDVGLRSDFITMN-PSVIQRAFGGFKN
Agema_4        EIDSHDFVIRR-CNLAP-LSEFAEDVGLRSDFTTMN-PSVIQRVYSGLLN
Anocar_4       EIDSHEFVIR--CNLAP-VVEFAADVGTKSDFITMN-PSVVQRAFGGFRN
Canlupfam_4    EIDSHNFVIR--CNLAP-VVEFAADVGTKSDFITMN-PSVVQRAFGGFRN
Crigri_4       EIDSHNFVIR--CNLAP-VVEFAADVGTKSDFITMN-PSVVQRAFGGFRN
Galgal_4       EIDTHDFVIR--CNLAP-VVEFAADVGNKSDFITMN-PSVVQRAFGGFRN
Mesaur_4       EIDSHNFVIR--CNLAP-VVEFAADVGTKSDFITMN-PSVVQRAFGGFRN
Homsap_4       EIDSHNFVIR--CNLAP-VVEFAADVGTKSDFITMN-PSVVQRAFGGFRN
Notscuscu_4    EIDSHEFVIR--CNLAP-VVEFAADVGTKSDFITMN-PSVVQRAFGGFRN
Psetextex_4    EIDSHEFVIR--CNLAP-VVEFAADVGTKSDFITMN-PSVVQRAFGGFRN
Musmus_4       EIDSHNFVIR--CNLAP-VVEFAADVGTKSDFITMN-PSVVQRAFGGFRN
Pogvit_4       EIDNHEFVIR--CNLAP-VVEFAADVGTKSDFITMN-PSVVQRAFGGFRN
Promuc_4       EIDNHEFVIR--CNLAP-VVEFAADVGTKSDFITMN-PSVVQKAFGGFRN
Pantro_4       EIDSHNFVIR--CNLAP-VVEFAADVGTKSDFITMN-PSVVQRAFGGFRN
Pytbiv_4       EIDNHEFVIR--CNLAP-VVEFAADVGTKSDFITMN-PSVVQRAFGGFRN
Ratnor_2       EIDTHSFVIR--CNLAP-VQEYARDVGLKTDLVTMN-PSVIQRAFEDLVN
Ratnor_4       EIDSHNFVIR--CNLAP-VVEFAADVGTKSDFITMN-PSVVQRAFGGFRN
Siltro_2       EIDSHDFVIR--CNLAP-VEEYATDVGTKTNLVTMN-PSVVQRAFEDLVN
Siltro_4       EIDSHDFVIR--CNLAP-VVEFAAHVGTKSDFITMN-PSVVQRAFGGFRN
Susscr_4       EIDSHNFVIR--CNLAP-VVEFAADVGTKSDFITMN-PSVVQRAFGGFRN
Taegut_4       EIDSHDFVIR--CNLAP-VVEYAADVGTKSDFITMN-PSVVQRAFGGFRN
Termextri_4M   EIDNHDFVIR--CNLAP-VVEFAADVGTKSDFITMN-PSVVQRAFGGFRN
Thasirsir_4    EIDSHEFVIR--CNLAP-VVEFAADVGTKSDFITMN-PSVVQRAFGGFRN
Xenlae_4B      EIDSHDFVIR--CNLAP-VVEFAADVGTKSDFITMN-PSVVQRAFGGFRN
Xenlae_4A      EIDSHDFVIR--CNLAP-VVEFAADVGTKSDFITMN-PSVVQRAFGGFRN
               101
Latcal_3       VQDR-NNFFLSLKKLDGAILWIPAFFFHTSATVTRTLVDFFVEHRGQ---
Perflu_3       VQDR-NNFFLSLKKLDGAILWIPAFFFHTSATVTRTLVDFFVEHRGQ---
Serdum_3       VQDR-NNFFLSLKKLDGAILWIPAFFFHTSATVTRTLVDFFVEHRGQ---
Stepar_3       VQDR-NNFFLSLKKLDGAILWIPAFFFHTSATVTRTLVDFFVEHRGQ---
Tetcal_3       IQDR-NKFFLNLKRLDGAILWIPAFFFHTSAPVTRTLVDFFIEHRAQ---
Monalb_3       VQDR-NNFFLSLKKLDEAILWIPAFFFHTSATVTRTLVDFFVEHQGQ---
Molmol_3       VQDR-NNFFLSLKKLDGVILWIPAFFFHTSATVTRTLVDFFVEHRGQ---
Takrub_3       VQDR-NNFFLSLKKLDTTILWIPAFFFHTSATVTRTLVDFFVEHRGQ---
Canlupfam_3    IQDR-NNFFLSLKKLDGAILWIPAFFFHTSATVTRTLVDFFVEHRGQ---
Bostau_3       IQDR-NNFFLSLKKLDGAILWIPAFFFHTSATVTRTLVDFFVEHRGQ---
Homsap_3       IQDR-NNFFLSLKKLDGAILWIPAFFFHTSATVTRTLVDFFVEHRGQ---
Siltro_3       IQDR-NNFFLSLKKLDGAILWIPAFFFHTSASVTRTLVDFFVEHRDQ---
Anocar_3       IQDR-NNFFLSLKKLDGAILWIPAFFFHTSATITRTLVDFFIEHRAQ---
Musmus_3       IQDR-NNFFLSLKKLDGAILWIPAFFFHTSATVTRTLVDFFVEHRGQ---
Pantro_3       IQDR-NNFFLSLKKLDGAILWIPAFFFHTSATVTRTLVDFFVEHRGQ---
Ratnor_3       IQDR-NNFFLSLKKLDGAILWIPAFFFHTSATVTRTLVDFFVEHRGQ---
Galgal_3       IQDR-NNFFLSLKKLDGAILWIPAFFFHTSATVTRTLVDFFVEHRGQ---
Lepocu_3       IQDR-NNFFLSLKKLDGAILWIPAFFFHTSATVTRTLVDFFVEHKGQ---
Tetnig_3       VQDR-NNFFLSLKRLDPAILWIPAFFFHTSATVTRTLVDFFVEPRGQ---
Squaca_3       IQDR-NKFFLNLKKLDGAILWIPAFFFHTSAPVTRTLVDFFMEHRAQ---
Perfla_3       VQDR-NNFFLSLKKLDGAILWIPAFFFHTSATVTRTLVDFFVEHRGQ---
Salsal_3       IQDR-NNFFLSLKKLDRAILWIPAFFFHTSATVTRTLVDFFVEHRGQ---
Serlaldor_3    VQDR-NNFFLSLKKLDGAILWIPAFFFHTSATVTRTLVDFFVEHRGQ---
Treber_3       IQDR-NNFFLNLKKLEGAILWIPAFFLHTSATVTRTLVDFFVEHKGQ---
Cynsem_3       VQDR-NNFFLSLKKLDNAILWIPAFFFHTSATVTRTLVDFFVEHRGQ---
Paroli_3       IQDR-NNFFLNLKKLEGAILWIPAFFLHTSATVTRTLVDFFVEHKGQ---
Paroli_3b      VQDR-NNFFLSLKKLDNAILWIPAFFFHTSATVTRTLVDFFVEHRGQ---
Plaste_3       IQDR-NNFFLNLKKLESAILWIPAFFLHTSATVTRTLVDFFVEHKGQ---
```

```
Plaste_3b      VQDR-NNFFLSLKKLDSAILWIPAFFFHTSATVTRTLVDFFVEHRGQ---
Erpcal3        IQDR-NNFFLSLKKLDGAILWIPAFFFHTSATVTRTLVDFFVEHKGQ---
Calmil_3b      IQDR-NKFFLNLKKLDGAILWIPAFFFHSSALVTRTLVDFFVEHRRQ---
Hetzeb_3       IQDR-NKFFLNLKKLDGAILWIPAFFFHTSAPVTRTLVDFFIEHRAQ---
Latcha_3       IQDR-NNFFLSLKKLDGAILWIPAFFFHTSATVTRTLVDFFVEHKGQ---
Anates_3       VQDR-NNFFLSLKKLDGAILWIPAFFFHTSATVTRTLVDFFVEHRGQ---
Ampcit3        VQDR-NNFFLSLKKLDGVILWIPAFFFHTSATVTRTLVDFFVEHRDQ---
Auslim_3       VQDR-NNFFLSLKKLDGAILWIPAFFFHTSATVTRTLVDFFVEHRGQ---
Cypvar_3       VQDR-NNFFLSLKKLDRAILWIPAFFFHTSATVTRTLVDFFVEHRGQ---
Krymar_3       VQDR-NNFFLSLKKLDSAILWIPAFFFHTSATVTRTLVDFFVEHRGQ---
Funhet_3       VQDR-NNFFLSLKKLDRAILWIPAFFFHTSATVTRTLVDFFVEHRGQ---
Gamaff3        VQDR-NNFFLSLKKLDRAILWIPAFFFHTSATVTRTLVDFFVEHRGQ---
Orylat_3       VQDR-NNFFLSLKKLDGAILWIPAFFFHTSATVTRTLVDFFVEHRGQ---
Orenil_3       VQDR-NNFFLSLKKLDGAILWIPAFFFHTSATVTRTLVDFFVEHRGQ---
Mayzeb_3       VQDR-NNFFLSLKKLDGAILWIPAFFFHTSATVTRTLVDFFVEHKGQ---
Punnye_3       VQDR-NNFFLSLKKLDGAILWIPAFFFHTSATVTRTLVDFFVEHKGQ---
Punnye_3a      VQDR-NNFFLSLKKLDGAILWIPAFFFHTSATVTRTLVDFFVEHKGQ---
Neobri_3       VQDR-NNFFLSLKKLDGAILWIPAFFFHTSATVTRTLVDFFVEHKGQ---
Misang_3       IQDR-NNFFLSLKKLDGAILWIPAFFFHTSATVTRTLVDFFVEHKGE---
Sinrhi3        IQDR-NNFYLSLKKLDGAILWIPAFFFHTSATVTRTLVDFFVEHKGQ---
Singra_3       IQDR-NNFFLSLKKLDGAILWIPAFFFHTSATVTRTLVDFFVEHKGQ---
Hapbur_3       VQDR-NNFFLSLKKLDGAILWIPAFFFHTSATVTRTLVDFFVEHKGQ---
Astcal_3       VQDR-NNFFLSLKKLDGAILWIPAFFFHTSATVTRTLVDFFVEHKGQ---
Notfur_3       VQDR-NNFFLSLKKLDGAVLWIPAFFFHTSATVTRTLVDFFVEHRGQ---
Cypcar_3       IQDR-NNFYLSLKKLDGAILWIPAFFFHTSATVTRTLVDFFVEHKGQ---
Poefor3        VQDR-NNFFLSLKKLDRAILWIPAFFFHTSATVTRTLVDFFVEHRGQ---
Poelat_3       VQDR-NNFFLSLKKLDRAILWIPAFFFHTSATVTRTLVDFFVEHRGQ---
Poeret_3       VQDR-NNFFLSLKKLDTAILWIPAFFFHTSATVTRTLVDFFVEHRGQ---
Angang_3       IQDR-NNFFLSLKKLDGVVLWIPAFFFHTSATVTRTLVDFFVEHRGQ---
Xipmac_3       VQDR-NNFFLSLKKLDRAILWIPAFFFHTSATVTRTLVDFFVEHRGQ---
Masarm_3       VQDR-NNFFLSLKKLDGAILWIPAFFFHTSATVTRTLVDFFVEHRGQ---
Permag_3       VQDR-NNFFLSLKRLDGAILWIPAFFFHTSATVTRTLVDFFVEHRGK---
Gnapet3        IQDR-NNFFLSLKKLDSAILWIPAFFFHTSATVTRTLVDFFVEHKGQ---
Ostbic_3       IQDR-NNFFLSLKKLDDAILWIPAFFFHTSATVTRTLVDFFVEHKDQ---
Gadmor_3       VQDR-NNFFLSLKRLDGAILWIPAFFFHTSATVTRTLVDFFVEHRGQ---
Sclfor_3       IQDR-NNFFLSLKKLDDAILWIPAFFFHTSATVTRTLVDFFVEHKGQ---
Acapol3        VQDR-NNFFLSLKKLDGAILWIPAFFFHTSATVTRTLVDFFVEHRGQ---
Ampoce_3       VQDR-NNFFLSLKKLDGAILWIPAFFFHTSATVTRTLVDFFVEHRGQ---
Sclfor_3b      IQDR-NNFFLSLKKLDGAILWIPAFFFHTSATVTRTLVDFFVEHKGQ---
Ostbic_3b      IQDR-NNFFLSLKKLDGAILWIPAFFFHTSATVTRTLVDFFVEHKGQ---
Parkin_3       IQDR-NNFFLSLKKLDSAILWIPAFFFHTSATVTRTLVDFFVEHKGQ---
Ampper3        VQDR-NNFFLSLKKLDGAILWIPAFFFHTSATVTRTLVDFFVEHRGQ---
Oncmyk_3       LQDR-NNFFLSLKKLDGAILWIPAFFFHTSATVTRTLVDFFVEHRGQ---
Gasacu_3       VQDR-NNFFLSLKKLDGVILWIPAFFFHTSATVTRTLVDFFVEHRGQ---
Labber_3       VQDR-NNFFLSLKRLDGAILWIPAFFFHTSATVTRTLVDFFVEHRGQ---
Panbuc_3       IQDR-NNFFLSLKKLDGAVLWIPAFFFHTSATVTRTLVDFFVEHQGQ---
Parhas3        IQDR-NNFFLSLKKLDGAILWIPAFFFHTSATVTRTLVDFFVEHRGQ---
Cluhar_3       IQDR-NNFFLSLKKLDGAILWIPAFFFHTSATVTRTLVDFFVEHKGQ---
Aloalo_3       IQDR-NNFFLSLKKLDGAILWIPAFFFHTSATVTRTLVDFFVEHKGQ---
Panhyp_3       IQDR-NNFFLSLKKLDGAILWIPAFFFHTSATVTRTLVDFFVEHRGQ---
Eleele_3       IQDR-NNFFLSLKKLDGAILWIPAFFFHTSATVTRTLVDFFVEHRGQ---
Denclu_3       IQDR-NNFFLSLKKLDGAVLWIPAFFFHTSATVTRTLVDFFVEHKGQ---
Sarpil_3       IQDR-NNFFLSLKKLDGAILWIPAFFFHTSATVTRTLVDFFVEHKGQ---
Eigvir_3       IQDR-NNFFLSLKKLDGAILWIPAFFFHTSATVTRTLVDFFVEHQGQ---
Aptalb_3       IQDR-NNFFLSLKKLDGAILWIPAFFFHTSATVTRTLVDFFVEHRGQ---
Sinans_3       IQDR-NNFYLSLKKLDGAILWIPAFFFHTSATVTRTLVDFFVEHKGQ---
Pygnat_3       IQDR-NNFFLSLKKLDRAILWIPAFFFHTSATVTRTLVDFFVEHRGQ---
Astmex_3       IQDR-NNFFLSLKKLDRAVLWIPAFFFHTSATVTRTLVDFFVEHQGQ---
Caraur_3       IQDR-NNFYLSLKKLDGAILWIPAFFFHTSATVTRTLVDFFVEHKGQ---
Danrer_3       IQDR-NNFFLSLKKLDGAILWIPAFFFHTSATVTRTLVDFFVEHKGQ---
Ictpun_3       IQDR-NNFFLSLKKLDGAILWIPAFFFHTSATVTRTLVDFFVEHRGQ---
Plealt_3       IQDR-NNFFLSLKKLDGAILWIPAFFFHTSATVTRTLVDFFVEHRGQ---
Plealt_9       IQDR-NNFFLNLKKLEGAILWIPAFFLHTSATVTRTLVDFFVEHKGQ---
Gasacu_9       IQDR-NNFFLNLKKLEGAILWIPAFFLHTSATVTRTLVDFFVEHKGQ---
Parkin_9       IQDR-NNFLLRLKKLDGAILWIPAFFFHTSAPVTRTLIDFFIEHKDQ---
Acapol_9       IQDR-NNFFLNLKKLEGAILWIPAFFLHTSATVTRTLVDFFVEHKGQ---
Larcro_9       IQDR-NNFFLNLKKLEGAILWIPAFFLHTSATVTRTLVDFFVEHKGQ---
Notcor_9       IQDR-NNFFLNLKKLEGAILWIPAFFLHTSATVTRTLVDFFVEHKGQ---
Latcal_9       IQDR-NNFFLNLKKLEGAILWIPAFFLHTSATVTRTLVDFFVEHKGQ---
Serlaldor_9    IQDR-NNFFLNLKKLEGAILWIPAFFLHTSATVTRTLVDFFVEHKGQ---
Monalb_9       IQDR-NNFFLSLKKLEGAILWIPAFFLHTSATVTRTLVDFFVEHKGQ---
Serdum_9       IQDR-NNFFLNLKKLEGAILWIPAFFLHTSATVTRTLVDFFVEHKGQ---
Perflu_9       IQDR-NNFFLNLKKLEGAILWIPAFFLHTSATVTRTLVDFFVEHKKQ---
Perfla_9       IQDR-NNFFLNLKKLEGAILWIPAFFLHTSATVTRTLVDFFVEHKKQ---
Stepar_9       IQDR-NNFFLNLKKLEGAILWIPAFFLHTSATVTRTLVDFFVEHKGQ---
Molmol_9       IQDR-NNFFLNLKKLEGAILWIPAFFLHTSATVTRTLVDFFVEHKGQ---
Hipcom_9       IQDR-NNFFLHLKKLEGAILWIPAFFLHTSATVTRTLVDFFVEHKGQ---
```

```
Takrub_9 B      IQDR-NNFFLNLKKLGGAILWIPAFFLHTSATVTRTLVDFFVEHKGQ---
Takrub_9        IQDR-NNFFLNLKKLGGAILWIPAFFLHTSATVTRTLVDFFVEHKGQ---
Cynsem_9        IQDR-NNFFLNLKKLEGAILWIPAFFLHTSATVTRTLVDFFVEHKSQ---
Scomax_9        IQDR-NNFFLSLKKLEEAILWIPAFFLHTSATVTRTLVDFFVEHKGQ---
Corlav_9        IQDR-NNFFLNLKKLEGAILWIPAFFLHTSATVTRTLVDFFVEHKGQ---
Oncmyk_9        IQDR-NNFFLNLKKLEGAILWIPAFFLHTSATVTRTLVDFFVEHKGQ---
Salsal_9        IQDR-NNFFLNLKKLEGAILWIPAFFLHTSATVTRTLVDFFVEHKGQ---
Onctsh_9        IQDR-NNFFLNLKKLEGAILWIPAFFLHTSATVTRTLVDFFVEHKGQ---
Salalp_9        IQDR-NNFFLNLKKLEGAILWIPAFFLHTSATVTRTLVDFFVEHKGQ---
Ampoce_9        IQDR-NNFFLNLKKLEGAILWIPAFFLHTSATVTRTLVDFFVEHKGQ---
Ampper9         IQDR-NNFFLNLKKLEGAILWIPAFFLHTSATVTRTLVDFFVEHKGQ---
Labber_9        IQDR-NNFFLNLKKLEGAILWIPAFFLHTSATVTRTLVDFFVEHKGQ---
Umbpyg_3B       IQDR-NNFFLSLKKLDGAILWI-AFFFHTSATVTRTLVDFFVEHRGQ---
Esoluc_3b       IQDR-NNFFLSLKKLDGAILWIPAFFFHTSATVTRTLVDFFVEHRGQ---
Gadmor_9        IQDR-NNFFLNVKKLEGAILWIPAFFLHTSATVTRTLVDFFVEHKGQ---
Umbpyg_9        IQDR-NNFFLNLKKLEGAILWIPAFFLHTSATVTRTLVDFFVEHKGQ---
Esoluc_9        IQDR-NNFFLNLKKLEGAILWIPAFFLHTSATVTRTLVDFFVEHKGQ---
Permag_9        IQDR-NNFFLNLKKLEGAILWIPAFFLHTSATVTRTLVDFFVEHKGQ---
Bolpec_9        IQDR-NNFFLNLKKLEGAILWIPAFFLHTSATVTRTLVDFFVEHKGQ---
Poefor9         IQDR-NNFFLNLKKLEGAILWIPAFFLHTSATVTRTLVDFFVEHKGQ---
Masarm_9        IQDR-NNFFLSLKKLEGAILWIPAFFLHTSATVTRTLVDFFVEHKGQ---
Angjap_9        IQDR-NNFFLSLKKLDGVVLWIPAFFFHTSATVTRTLVDFFVEHRGQ---
Poelat_9        IQDR-NNFFLNLKKLEGAILWIPAFFLHTSATVTRTLVDFFVEHKGQ---
Poeret_9        IQDR-NNFFLNLKKLEGAILWIPAFFLHTSATVTRTLVDFFVEHKGQ---
Xipmac_9        IQDR-NNFFLNLKKLEGAILWIPAFFLHTSATVTRTLVDFFVEHKGQ---
Hapbur_9        IQDR-NNFFLNLKKLEGAILWIPAFFLHTSATVTRTLVDFFVEHKGQ---
Orylat_9        IQDR-NNFFLHLKKLEGAILWIPAFFLHTSATVTRTLVDFFVEHKGQ---
Gamaff_9        IQDR-NNFFLNLKKLEGAILWIPAFFLHTSATVTRTLVDFFVEHKGQ---
Neobri_9        IQDR-NNFFLNLKKLEGAILWIPAFFLHTSATVTRTLVDFFVEHKGQ---
Funhet_9        IQDR-NNFFLNLKKLEGAILWIPAFFLHTSATVTRTLVDFFVEHKGQ---
Punnye_9        IQDR-NNFFLNLKKLEGAILWIPAFFLHTSATVTRTLVDFFVEHKGQ---
Cypvar_9        IQDR-NNFFLNLKKLEGAILWIPAFFLHTSATVTRTLVDFFVEHKGQ---
Krymar_9        IQDR-NNFFLNLKKLEGAILWIPAFFLHTSATVTRTLVDFFVEHKGQ---
Notfur_9        IQDR-NNFFLNLKKLEGAILWIPAFFLHTSATVTRTLVDFFVEHKGQ---
Notpie_9        IQDR-NNFFLNLKKLEGAILWIPAFFLHTSATVTRTLVDFFVEHKGQ---
Orenil_9        IQDR-NNFFLNLKKLEGAILWIPAFFLHTSATVTRTLVDFFVEHKGQ---
Auslim_9        IQDR-NNFFLNLKKLEGAILWIPAFFLHTSATVTRTLVDFFVEHKGQ---
Anates_9        IQDR-NNFFLNLKKLEGAILWIPAFFLHTSATVTRTLVDFFVEHKGQ---
Astcal_9        IQDR-NNFFLNLKKLEGAILWIPAFFLHTSATVTRTLVDFFVEHKGQ---
Mayzeb_9        IQDR-NNFFLNLKKLEGAILWIPAFFLHTSATVTRTLVDFFVEHKGQ---
Orymel_9        IQDR-NNFFLHLKKLEGAILWIPAFFLHTSATVTRTLVDFFVEHKGQ---
Ampcit9         IQDR-NNFFLNLKKLEGAILWIPAFFLHTSATVTRTLVDFFVEHKGQ---
Macfas_2        ATWR-EKLLQRLHSLNGSILWIPAFMARGGKERVEWVNELILKHH-----
Macmul_2        ATWR-EKLLQRLHSLNGSILWIPAFMARGGKERVEWVNELILKHH-----
Macnem_2        ATWR-EKLLQRLHSLNGSILWIPAFMARGGKERVEWVNELILKHH-----
Musmus_2        ATWR-EKLLQRLHGLNGSILWIPAFMARGGKERVEWVNALILKHH-----
Ponabe_2        ATWR-EKLLQRLHSLNGSILWIPAFMARGGKERVEWVNELILKHH-----
Papanu_2        ATWR-EKLLQRLHSLNGSILWIPAFMARGGKERVEWVNELILKHH-----
Nomleu_2        ATWR-EKLLQRLHSLNGSILWIPAFMARGGKERVEWVNELILKHH-----
Pantro_2        ATWR-EKLLQRLHSLNGSILWIPAFMARGGKERVEWVNELILKHH-----
Latcha_2        ETWK-EKFLQRLQSLNGSIFWIPAFMAKGGEERVEWVNDLIIKSG-----
Gnapet2         QQWR-DHFLQRLRSIAGGVLWIPAFMAKGGEERVELAARLILRHG-----
Panbuc_2b       ESWQ-ERFLQRLDQLGSSVLWIPAFMAKGGEERVEWALDLIRQHA-----
Ostbic_2        KQWK-GRFLQRLQGLNGSVLWIPAFMAKGGEERVAWAVRLILQHA-----
Sinrhi2         EEWV-QRFVHRLQSLSGSVLWIPAFMAKGGEERVEWAIRLILLHT-----
Hetzeb_2        ETWK-EKLLHRLKMLDGGILWIPAFMAKGGEERVEWVNNLIIKHK-----
Hipcom_2        DEWR-RRFARRLASLSGSVLWIPAFMAKGGEERVEWALRLILRHA-----
Prigla_2        ETWK-EKLLHRLKMLDDGILWIPAFMAKGGEERVEWVNKLIIEHK-----
Scytor_2        DTWK-EKLLHRLKMLDGGILWIPAFMAKGGEERVEWVNRLIIEHK-----
Tetcal_2        ETWK-EKLLHRLTMLDGAILWIPAFMAKGGEERVEWVNNLIIEHK-----
Rhityp_2        ETWK-EKLLHRLKMLDGGILWIPAFMAKGGEERVEWVNNLIIKHK-----
Parkin_2b       ARWR-EHFLQRLRGLGDAVLWIPAFMAKGGERRVELALDVIRRQR-----
Angang_2        EAWR-ERFVRRLRALEGSVLWIPAFMAKGGEDRVEWATRLILRHA-----
Pygnat_2        EEWR-QRFVQRLQALSGSVLWIPAFMAKGGEERVEWAVRLILSHT-----
Parkin_2        QLWR-DHFLQRLRSIAGGVLWIPAFMAKGGEERVELAARLILRHS-----
Gorgor_2        ATWR-EKLLQRLHSLNGSILWIPAFMARGGKERVEWVNELILKHH-----
Thegel_2        ATWR-EKLLQRLHSLNGSILWIPAFMARGGKERVEWVNELILKHH-----
Tupchi_2        ATWR-EKLLQRLHSLNGSILWIPAFMARGGKERVEWVNELILKHR-----
Galgal_2        ETWR-EKLLQRLHSLNGSILWIPAFMAKGGKERVEWVNELILKHR-----
Eleele_2        EEWR-QQFVQRLRELGSSVLWIPAFMAKGGEERVERAIRLILSHT-----
Danrer_2        EEWV-QRFVQRLQSLSGSVLWIPAFMAKGGEERVEWAIRLILLHT-----
Canlupfam_2     ATWR-EKLLQRLRSLNGSILWIPAFMARGGKERVEWVNELILKHH-----
Aloalo_2        EEWR-QRFIRRLQDLSGSVLWIPAFMAKGGEERVEWAIRLILTHT-----
Astmex_2        KEWR-QRFVRRLQALSGSVLWIPAFMAKSGEDRVEWAVHLILSHT-----
Sinans_2        EEWV-QRFVHRLQSLSGSVLWIPAFMAKGGEERVEWAIRLILLHT-----
Sclfor_2        EQWK-GRFLRRLQGLNGSVLWIPAFMAKGGEERVAWAVRLILQHA-----
Ceraty_2        ATWR-EKLLQRLHSLNGSILWIPAFMARGGKERVEWVNELILKHH-----
```

```
Anocar_2       DTWR-EKLLQRLHSLNGSILWIPAFMAKGGKERVEWVNELILKNH-----
Anates_2       EEWR-ARFLRRLQSLSGSVLWIPAFMAKGGEERVEWALRLILLHT-----
Ampbic_2       EEWT-ERFLQRLRSLSGSVLWIPAFMAKGGEERVEWALRLILLHT-----
Sarpil_2       EEWR-QRFIRRLQDLSGSVLWIPAFMAKGGEERVEWAIRLILTHT-----
Xenlae_2       DTWK-DKFLQRLKSLNESILWIPAFMAKGGEERVEWVNDLIIKHH-----
Cluhar_2       EEWR-QRFVRRLQDLSGSVLWIPAFMAKGGEERVEWAIRLIIMHT-----
Cypcar_2       EEWV-QRFVHRLQSLSGSVLWIPAFMAKGGEERVEWAIRLILLHT-----
Denclu_2       EEWR-ERFVQRLRALSGSVLWIPAFMAKGGEERVEWAIRLILLHT-----
Notpie_2       EEWK-ERFLQRLRNLSGSVLWIPAFMAKRGEDRVEWVLRLILLHT-----
Erpcal2        DTWK-DRLLQRLQNLNGSILWIPAFMAKGGEQRVELVNSLILKHQ-----
Amical_2       ESWR-ERFLQRLQSLNGSVLWIPAFMAKGGEERVEWANDLILKHH-----
Lepocu_2       SHWR-ERFLERLSSLNGSVLWIPSFMAKGGEERVQWASELILRHG-----
Homsap_2       ATWR-EKLLQRLHSLNGSILWIPAFMARGGKERVEWVNELILKHH-----
Bostau_2       ATWR-EKLLQRLHSLNGSILWIPAFMARGGKQRVEWVNELILKHH-----
Caraur_2       EEWV-QRFVHRLQSLSGSVLWIPAFMAKGGEERVEWAIRLILLHT-----
Thythy_2B      EEWR-ERFLQRLRGLSGSVLWIPAFMAKGGEERVEWAIRLILLHT-----
Onctsh_2B      EEWR-ERFLQRLRSLSGSVLWIPAFMAKGGEERVEWAIRLILLHT-----
Salsal_2B      EEWR-ERFLQRLRGLSGSVLWIPAFMAKGGEERVEWAIRLILLHT-----
Oncmyk_2B      EEWR-ERFLQRLRSLSGSVLWIPAFMAKGGEERVEWAIRLILLHT-----
Onckis_2B      EEWR-ERFLQRLQSLSGSVLWIPAFMAKGGEERVEWAIRLILLHT-----
Salsal_2A      EQWR-ERFLQRLRGLSGSVLWIPAFMAKGGEERVEWAIRLILLHT-----
Thythy_2A      EQWR-ERFLQRLRGLSGSVLWIPAFMAKGGEERVEWAIRLILLHT-----
Salalp_2A      EQWR-ERFLQRLRGLSGSVLWIPAFMAKGGEERVEWAIRLILLHT-----
Corlav_2A      EQWR-ERFLQRLRGLSGSVLWIPAFMAKGGEERVEWAIRLILLHT-----
Cormar_2A      EQWR-ERFLQRLRGLSGSVLWIPAFMAKGGEERVEWAIRLILLHT-----
Onctsh_2A      EQWR-ERFLQRLRGLSGSVLWIPAFMAKGGEERVEWAIRLILLHT-----
Oncmyk_2A      EQWR-ERFLQRLRGLSGSVLWIPAFMAKGGEERVEWAIRLILLHT-----
Neobri_2       NEWK-ERFLRRLRSLSGSVLWIPAFMAKGGEERVEWALRLILLHT-----
Takrub_2       EEWR-ARFLRRLQSLSGSVLWIPAFMAKGGEERVEWTLRLILLHT-----
Tetnig_2       EEWR-DRFLRRLQSLSGSILWIPAFMAKGGEERVDWTLRLILLHT-----
Orenil_2       DEWK-ERFLRRLRSLSGSVLWIPAFMAKGGEERVEWALRLILLHT-----
Orylat_2       EEWK-QRFLQRLQSLGGSVLWIPAFMAKGGEERVEWALRLILRHT-----
Krymar_2       EEWR-ERFLQRLRDLRGGVLWVPAFMAKGGEERVEWALRLILLHA-----
Notfur_2       EEWK-ERFLQRLRNLSGSVLWIPAFMAKRGEDRVEWVLRLILLHT-----
Poelat_2       DEWK-DRFLQRLQNLSGSVLWIPAFMAKGGEERVEWALRLILVHT-----
Gadmor_2       PEWR-ARFVARLQSLRGSVLWIPAFMAKGGEERVEWAARLILLHT-----
Xipmac_2       DEWK-DRFLQRLQNLSGSVLWIPAFMAKGGEERVEWALRLILVHT-----
Poeret_2       DEWK-DRFLRRLQNLSGSVLWIPAFMAKGGEERVEWALRLILVHT-----
Astcal_2       NEWK-ERFLRRLRSLSGSVLWIPAFMAKGGEERVEWALRLILLHT-----
Hapbur_2       NEWK-ERFLRRLRSLSGSVLWIPAFMAKGGEERVEWALRLILLHT-----
Acapol_2B      EEWT-ERFLQRLRSLSGSVLWIPAFMAKGGEERVEWALRLILLHT-----
Ampoce_2A      EEWT-ERFLQRLRSLSGSVLWIPAFMAKGGEERVEWALRLILLHT-----
Acapol_2A      EEWT-ERFLQRLRSLSGSVLWIPAFMAKGGEERVEWALRLILLHT-----
Mayzeb_2       NEWK-ERFLRRLRSLSGSVLWIPAFMAKGGEERVEWALRLILLHT-----
Punnye_2       NEWK-ERFLRRLRSLSGSVLWIPAFMAKGGEERVEWALRLILLHT-----
Latcal_2       EEWR-DRFLQRLRSLSGSVLWIPAFMAKGGEERVEWALRLILLHT-----
Singra_2       EEWV-QRFVHRLQSLSGSVLWIPAFMAKGGEERVEWAIRLILLHT-----
Auslim_2       EEWK-ERFLRRLRDLGGSVLWIPAFMAKGGEERVEWALRLILLHA-----
Monalb2        DEWR-ERFLQRLQSLSGSVLWIPAFMAKGGEERVEWALRLILLHT-----
Ampoce_2B      EEWT-ERFLQRLRSLSGSVLWIPAFMAKGGEERVEWALRLILLHT-----
Chiham_2       EEWR-DRFLQRLQSLSGSVLWIPAFMAKGGEERVEWALRLILSHT-----
Gasacu_2       DEWR-DRFLQRLQSLSGSVLWIPAFMAKGGEERVEWALRLILSHT-----
Gymacu_2       EEWR-DRFLQRLQSLSGSVLWIPAFMAKGGEERVEWALRLILSHT-----
Labber_2       EEWR-DRFLQRLQSLSGSVLWIPAFMAKGGEERVEWALRLILLHT-----
Cypvar_2       EEWK-DRFLQRLRDLSGSVLWIPAFMAKGGEERVEWALRLILVHT-----
Funhet_2       DEWK-ERFLQRLRNLSGSVLWIPAFMAKGGEERVEWALRLIRVHT-----
Larcro_2       EEWR-DRFLKRLQSLSGSVLWIPAFMAKGGEERVEWALRLILLHT-----
Paroli_2       DEWR-DRFLQRLQSLSGSVLWIPAFMAKGGEERVEWALRLILLHT-----
Perflu_2       EEWR-DRFLQRLQSLSGSVLWIPAFMAKGGEERVEWALRLILLHT-----
Serdum_2       EEWR-DRFLQRLQSLSGSVLWIPAFMAKGGEERVEWALRLILLHT-----
Stepar_2       EEWK-ERFLQRLRSLSGSVLWIPAFMAKGGEERVEWALRLILLHT-----
Notcor_2       EEWR-DRFLQRLQSLSGSVLWIPAFMAKGGEERVEWALRLILSHT-----
Treber_2       EEWR-DRFLQRLQSLSGSVLWIPAFMAKGGEERVEWALRLILSHT-----
Serlaldor_2    EEWR-DRFLQRLQSLSGSVLWIPAFMAKGGEERVEWALRLILLHT-----
Plaste_2       DEWR-NRFLQRLQSLSGSVLWIPAFMAKGGEERVEWALRLILLHT-----
Clabat_4       ETTR-ERFVQRLRLLNDSVLWIPAFMVKGGEKHVEGVNELILKKK-----
Ictpun_4A      ETVR-ENFVQRLRLLNDSVLWIPAFMVKGGEKHVEVVNELILKRK-----
Panhyp_4       ETVR-ELFVQRLSLLNDSVLWIPAFMVKGGEKHVEGVNELILKRK-----
Umbpyg_4       ATDT-ERFVQRLRMLKDSVLWIPAFMVKGGEQHVERVNELIVKRK-----
Esoluc_4       ASDM-ERFVQRLRMLNDSVLWIPAFMVKGGERHVESVNELIVKRK-----
Plealt_4       VSDA-ERFVRRLRMLNDSVLWIPAFMVKGGEKHVECVNELIVKRK-----
Onckis_4B      ATDT-ERFVQRLRGLNDSVLWIPAFMVKGGERHVESVNELIVKRK-----
Onckis_4A      ATDT-ERFVQRLRGLNDSVLWIPAFMVKGGERHVESVNELIVKRK-----
Onctsh_4       ATDT-ERFVQRLRGLNDSVLWIPAFMVKGGERHVESVNELIVKRK-----
Oncmyk_4       ATDT-ERFVQRLRGLNDSVLWIPAFMVKGGERHVESVNELIVKRK-----
Corlav_4       ATDT-ERFVQRLRMLNDSVLWIPAFMVKGGERHVESVNELIVKRK-----
Astmex_4A      ETQR-ERFVERLSMLNDSVLWIPAFMVKGGEKHVEGVNELILKRK-----
```

```
Pygnat_4        ETVR-EHFVERLGMLNDSVLWIPAFMVKGGEKHVEGVNELILKRK-----
Aloalo_4        ATDR-ERFVERLRALNDSVLWIPAFMVKGGEKHVEGVNELILQRS-----
Cluhar_4        ATDR-ERFVERLQALNDSVLWIPAFMVKGGEKHVEGVNELILQRG-----
Denclu_4        ESDR-EKFVHRLIALNDSVLWIPAFMVKGGEKHVEVVNNFILKQG-----
Konpun4         STDR-ERFVERLRALNDSVLWIPAFMVKGGEKHVEGVNELILQRG-----
Sarpil_4        ATDR-ERFVERLRALNDSVLWIPAFMVKGGEKHVEGVNELILQRG-----
Caraur_4        ERQQ-ERFIRRLQQLNNSVLWIPAFMVKGGERHVEIVNQLILKHK-----
Cteide_4        ETEQ-ENFILRLQQLNNSVLWIPAFTVKGSERHVEIVNELILKHK-----
Petmar_4        KADH-DRFGRRLRALNSSILWIPAFMAKGGEQHVEIVNNLLLTLG-----
Cypcar_4        ERQR-ERFIQRLQQLNNSVLWIPAFMVKGGERHVEIVNELILKHK-----
Danrer_4        ETQQ-ENLIQRLRQLNDSVLWIPAFMVKGGMKHVDTVNELILKHK-----
Misang_4        KTAQ-EHFIQRLRQLNDSVLWIPAFMVKGSEKHVEGVNELILKNK-----
Angjap_4        DSDR-ERFVRRLGALNDSVLWIPAFMVKGGERHVECVNDLILRHR-----
Cypcar_4A       ERQR-ERFIQRLQQLNNSVLWIPAFMVKGGERHVEIVNELILKHK-----
Angang_4        DSDR-ERFVRRLGALNDSVLWIPAFMVKGGERHVECVNDLILRHR-----
Cypcar_4B       ERQR-ERFIRRLQQLNNSVLWIPAFMVKGGERHVEIVNQLILKHK-----
Sinans_4A       ERQR-ESFILRLQQLNNSVLWIPAFMVKGGERHVEIVNELILKHK-----
Sinans_4B       ERQR-VQFIRRLQQLNNSVLWIPAFMVKGGERHVEIVNQLILKHR-----
Singra_4A       ERQQ-ESFIRRLQQLNNSVFWIPAFMVKGGERHVETVNELILKHK-----
Aptalb_4        ETVR-EGFLRRLAALNDSVLWIPAFMVKGGEKHVEGVNELILRRK-----
Eigvir_4        ETAR-ESFLRRLAALNDSILWIPAFMVKGGEKHVEGVNELILRRK-----
Eleele_4        ETAR-DCFLRRLAVLNDSVLWIPAFMVKGGEKHVEGVNELILQRK-----
Parhas_4        VTVR-EGFLRRLAALNDSVLWIPAFMVKGGEKHVEGVNELILRRK-----
Sclfor_4        ESDR-VRFVQRLTMLNESVLWIPAFMVKGGEQHVERVNELILKNK-----
Gnapet4         ESDR-ERFVQRLIMLNDSILWIPAFMVKGGEKHVEGVNELILNRK-----
Ostbic_4        ESDR-EKFVQRLIMLNDSILWIPAFMVKGGEQHVEHVSELILKNR-----
Parkin_4        ESDR-ERFVQRLIMLNDSILWIPAFMVKGGEKHVEGVNELILNRK-----
Panbuc_4        ATDR-QRFVQRLVALNDSVLWIPAFMVKGGEKHVESVNELILKNG-----
Erpcal4         ETDR-EKFVQRLAMLNDSVLWIPAFMVKGGEKHVEWVNELILKNK-----
Salsal_4        ATDT-ERFVQRLRGLNDSVLWIPAFMVKGGERHVESVNELIVKRK-----
Saltru_4        ATDT-ERFVQRLRGLNDSVLWIPAFMVKGGERHVESVNELIVKRK-----
Salalp_4        ATDT-ERFVERLRGLNDSVLWIPAFMVKGGERHVESVNELIVKRK-----
Salfon_4        ATDT-ERFVERLRGLNDSVLWIPAFMVKGGERHVESVNELIVKRK-----
Thythy_4        ATDT-ERFVHRLQVLNDSVLWIPAFMVKGGERHVESVNELIVKRK-----
Calmil_4        ETDR-ENFVRRLAVLNDSVLWIPAFMVKGGERHVEWVNELILKNK-----
Hetzeb_4        ETDR-EKFVDRLAVLNDSVLWIPAFMVKGGEKHVEYVNELILKNR-----
Rhityp_4        ETDR-EKFVDRLTVLNDSVLWIPAFMVKGGEKYVEYVNELILKNR-----
Scytor_4        ETDR-EKFVDRLAVLNDSVLWIPAFMVKGGEKHVEYVNELILKNR-----
Squaca_4        ETDR-EKFVDRLAVLNDSVLWIPAFMVKGGEKHVEYVNELILKNR-----
Amical_4        ETDR-EKFVRRLTMLNDSVLWIPAFMVKGGEKHVECVNELILKNK-----
Lepocu_4        ETDR-ERFVQRLTLLNDSVLWIPAFMVKGGEKHVECVNELILKNK-----
Agema_4         ETVR-ERFVQRLSLLNDSVLWIPAFMVKGGEKHVEGVNELILKRK-----
Anocar_4        ESDR-EKFVHRLSMLNDSVLWIPAFMVKGGEKHVEWVNALILKNK-----
Canlupfam_4     ESDR-EKFVHRLSMLNDSVLWIPAFMVKGGEKHVEWVNALILKNK-----
Crigri_4        ESDR-AKFVHRLSMLNDSVLWIPAFMVKGGEKHVEWVNALILKNK-----
Galgal_4        ESDR-EKFGHRLSMLNDSVLWIPAFMVKGGEKHLEWVNALILKNK-----
Mesaur_4        ESDR-EKFVHRLSMLNDSVLWIPAFMVKGGEKHVEWVNALILKNK-----
Homsap_4        ESDR-EKFVHRLSMLNDSVLWIPAFMVKGGEKHVEWVNALILKNK-----
Notscuscu_4     ESDR-EKFVHRLSMLNDSVLWIPAFMVKGGEKHVQWVNALILKNK-----
Psetextex_4     ESDR-EKFVHRLSMLNDSVLWIPAFMVKGGEKHVQWVNALILKNK-----
Musmus_4        ESDR-EKFVHRLSMLNDSVLWIPAFMVKGGEKHVEWVNALILKNK-----
Pogvit_4        ESDR-EKFVHRLSMLNDSVLWIPAFMVKGGEKHVEWVNALILKNK-----
Promuc_4        ESDR-EKFVHRLSMLNDSVLWIPAFMVKGGEKHVEWVNALILKNK-----
Pantro_4        ESDR-EKFVHRLSMLNDSVLWIPAFMVKGGEKHVEWVNALILKNK-----
Pytbiv_4        ESDR-EKFVHRLSMLNDSVLWIPAFMVKGGEKHVEWVNALILKNK-----
Ratnor_2        ATWR-EKLLQRLHGLNGSILWIPAFMARGGKERVEWVNALILKHH-----
Ratnor_4        ESDR-EKFVHRLSMLNDSVLWIPAFMVKGGEKHVEWVNALILKNK-----
Siltro_2        DTWK-DKFLQRLKSLNESILWIPAFMAKGGEERVEWVNDLIIKHH-----
Siltro_4        ESDR-EKFVHRLSMLNDSVLWIPAFMVKGGEKHVEWVNALILKNQ-----
Susscr_4        ESDR-EKFVHRLSMLNDSVLWIPAFMVKGGEKHVEWVNALILKNK-----
Taegut_4        ESDR-EKFVHRLSMLNDSVLWIPAFMVKGGEKHVEWVNALILKNK-----
Termextri_4M    ESDR-EKFVHRLSMLNDSVLWIPAFMVKGGEKHVEWVNALILKNK-----
Thasirsir_4     ESDR-EKFVHRLSMLNDSVLWIPAFMVKGGEKHVEWVNALILKNK-----
Xenlae_4B       ESDR-EKFVHRLSMLNDSVLWIPAFMVKGGEKHVEWVNALILKNQ-----
Xenlae_4A       ESDR-EKFVHRLSMLNDSVLWIPAFMVKGGEKHVEWVNALILKNQ-----
                151
Latcal_3        LKVQLAWPGN-IMQYINNYWKTKQLSPKRLSTGILMYT----LASSMCDQ
Perflu_3        LKVQLAWPGN-IMQYVNSYWKTKQLSPKRLSTGILMYT----LASSMCDQ
Serdum_3        LKVQLAWPGN-IMQYINNYWKTKQLSPKRLSTGILMYT----LASSMCDQ
Stepar_3        LKVQLAWPGN-IMQYINNYWKTKQLSPKRLSTGILMYT----LASSMCDQ
Tetcal_3        LKVQLAWPGN-IMQHINKYWKTKQLSPKRLSTGILMYT----LASAICEE
Monalb_3        LKVQLAWPGN-IMQYINNYWKTKQLSPKRLSTGILMYT----LASSMCDQ
Molmol_3        LKVQLAWPGN-IMQYINNYWKTKQLSPKRLSTGILMYT----LASSMCDQ
Takrub_3        LKVRLAWPGN-IMQYINNYWKTKQLSPKRLSTGILMYT----LASSMCDQ
Canlupfam_3     LKVQLAWPGN-IMQHVNRYWKNKHLSPKRLSTGILMYT----LASAICEE
Bostau_3        LKLQLAWPGN-IMQHVNRYWKNKHLSPKRLSTGILMYT----LASAVCEE
Homsap_3        LKVQLAWPGN-IMQHVNRYWKNKHLSPKRLSTGILMYT----LASAICEE
```

```
Siltro_3        LKVQLDWPGN-IMQHVNRYWKNKHLSPKRLSTGILMYT----LASSVCEE
Anocar_3        LKVQLAWPGN-IMQHVNRYWKNKHLAPKRLSTGILMYT----LASAICDE
Musmus_3        LKVQLAWPGN-IMQHVNRYWKNKHLSPKRLSTGILMYT----LASAICEE
Pantro_3        LKVQLAWPGN-IMQHVNRYWKNKHLSPKRLSTGILMYT----LASAICEE
Ratnor_3        LKVQLAWPGN-IMQHVNRYWKNKHLSPKRLSTGFLMYT----LASAICEE
Galgal_3        LKVQLAWPGN-IMQHVNRYWKNKHLSPKRLSTGILMYT----LASAICEE
Lepocu_3        LKVQLAWPGN-IMQYVNRYWKTKHLAPKRLSTGILMYT----LASAMCEE
Tetnig_3        LKVQLAWPGN-IMQYINNYWKTKQLSPKRLSTGILMYT----LASSMCER
Squaca_3        LKIQLAWPGN-IMQHINKYWKTKQLSPKRLSTGILMYT----LASAICEE
Perfla_3        LKVQLAWPGN-IMQYVNSYWKTKQLSPKRLSTGILMYT----LASSMCDQ
Salsal_3        LKVQLAWPGN-IMQYVNRYWKTKQLSPKRLSTGILMYT----LASSMCEE
Serlaldor_3     LKVQLAWPGN-IMQYINNYWKTKQLSPKRLSTGILMYT----LASSMCDQ
Treber_3        LKVELAWPGN-IMHDVNKYWKTKNLSPKRLSTGILMYT----LASAMCDE
Cynsem_3        LKVQLAWPGN-IMQYIN-------------STGILMYT----LASSMCDQ
Paroli_3        LKVKLAWPGN-IMHDVNKYWKTKNLSPKRLSTGILMYT----LAYAMCDE
Paroli_3b       LKVQLAWPGN-IMQYINNYWKTKQLSPKRLSTGILMYT----LASSMCDQ
Plaste_3        LKVKLAWPGN-IMHDVNKYWKTKNLSPKRLSTGILMYT----LAYAMCDE
Plaste_3b       LKVQLAWPGN-IMQYINSYWKTKQLSPKRLSTGILMYT----LASSMCDQ
Erpcal3         LKVQLAWPGN-IMQHINRYWKTKHLAPKRLSTGILMYT----LASAMCEE
Calmil_3b       LKVELAWPGN-IMQYVNKYWKTKQLSPKRLSTGILMYT----LASSVCEE
Hetzeb_3        LKVQLAWPGN-IMQHINKYWKTKQLSPKRLSTGILMYT----LASAICEE
Latcha_3        LKLQLAWPGN-IMQHVNRYWKLKHLSPKRLSTGILMYT----LASAMCQE
Anates_3        LNVQLAWPGN-IMQYINNYWKTKQLSPKRLSTGILMYT----LASSMCDQ
Ampcit3         LKVQLAWPGN-IMKYVNNYWKTKQLSPKRLSTGILMYT----LASSMCDQ
Auslim_3        LKVQLAWPGN-IMQYINNYWKTKRLSPKRLSTGILMYT----LASSMCDQ
Cypvar_3        LKVQLAWPGN-IMQYINNYWKTKQLSPKRLSTGILMYT----LASSMCDQ
Krymar_3        LKVQLAWPGN-IMQYINNYWKTKQLSPKRLSTGILMYT----LASSMCDQ
Funhet_3        LKVQLAWPGN-IMQYINNYWKTKQLSPKRLSTGILMYT----LASSMCDQ
Gamaff3         LKVQLAWPGN-IMQHINNYWKTKQLSPKRLSTGILMYT----LASSMCDQ
Orylat_3        LKVQLAWPGN-IMQYINNYWKTKQLSPKRLSTGILMYT----LASSMCDQ
Orenil_3        LKVQLAWPGN-IMKYVNNYWKTKQLSPKRLSTGILMYT----LASSMCDQ
Mayzeb_3        LKVQLAWPGN-IMKYVNNYWKTKQLSPKRLSTGILMYT----LASSMCDQ
Punnye_3        LKVQLAWPGN-IMKYVNNYWKTKQLSPKRLSTGILMYT----LASSMCDQ
Punnye_3a       LKVQLAWPGN-IMKYVNNYWKTKQLSPKRLSTGILMYT----LASSMCDQ
Neobri_3        LKVQLAWPGN-IMKYVNNYWKTKQLSPKRLSTGILMYT----LASSMCDQ
Misang_3        LKVKLAWPGN-IMQYVNRYWKTKHLSPKRLSTGILMFT----LASSMCEQ
Sinrhi3         LKVQLAWPGN-IMQYVNRYWKTKQLSPKRLSTGILMFT----LASSLCEQ
Singra_3        LKVQLAWPGN-IMQYVNRYWKTKQLSPKRLSTGILMFT----LASSLCEQ
Hapbur_3        LKVQLAWPGN-IMKYVNNYWKTKQLSPKRLSTGILMYT----LASSMCDQ
Astcal_3        LKVQLAWPGN-IMKYVNNYWKTKQLSPKRLSTGILMYT----LASSMCDQ
Notfur_3        LKVQLAWPGN-IMQYINNYWKTKQLSPKRLSTGILMYT----LASSMCDQ
Cypcar_3        LKVRLAWPGN-IMQYVNRKWTTASF-------------------------
Poefor3         LKVQLAWPGN-IMQYINNYWKTKQLSPKRLSTGILMYT----LASSMCDQ
Poelat_3        LKVQLAWPGN-IMQYINNYWKTKQLSPKRLSTGILMYT----LASSMCDQ
Poeret_3        LRVQLAWPGN-IMQYINNYWKTKQLSPKRLSTGILMYT----LASSMCDQ
Angang_3        LKVQLAWPGN-IMQYVNRYWKTRQLSPKRLSTGILMYT----LASAMCEE
Xipmac_3        LKVQLAWPGN-IMQHINNYWKTKHLSPKRLSTGILMYT----LASSMCDQ
Masarm_3        LKVQLAWPGN-IMQYINNYWKTKQLSPKRLSTGILMYT----LASSMCDQ
Permag_3        LNVQLAWPGN-IMQYINNYWKTKQLSPKRLSTGILMYT----LASS----
Gnapet3         LKVQLAWPGN-IMQYVNRYWKTKQLSPKRLSTGILVYT----LAAAMCEE
Ostbic_3        LKVQLAWPGN-IMQHVNRFWKTKQLSPKRLSTGILMYT----LASGMCDQ
Gadmor_3        LKVQLAWPGN-IMQYVNSYWKTKQLSPKRLSTGILMYT----LASSMCDQ
Sclfor_3        LKVQLAWPGN-IMQHIKRFWKTKHLSPKRLSTGILMYT----LASAMCDQ
Acapol3         LKVQLAWPGN-IMQYINNYWKTKQLSPKRLSTGILMYT----LASSMCDQ
Ampoce_3        LKVQLAWPGN-IMQYINNYWKTKQLSPKRLSTGILMYT----LASSMCDQ
Sclfor_3b       LKVQLAWPGN-IMKFFNRYWKTKQLSPKRLSTGILMYT----LASVMCEE
Ostbic_3b       LKVQLAWPGN-IMKFFNRYWKTKQLSPKRLSTGILMYT----LASAMCEE
Parkin_3        LKVQLAWPGN-IMQYVNRYWKTKQLSPKRLSTGILVYT----LAAAMCEE
Ampper3         LKVQLAWPGN-IMQYINNYWKTKQLSPKRLSTGILMYT----LASSMCDQ
Oncmyk_3        LKVQLAWPGN-IMQYINRYWKTKQLSPKRLSTGILMYT----LASSMCEE
Gasacu_3        LKVRLAWPGN-IMQYVNSYWKTKQLSPKRLSTGILMYT----LASTMCDQ
Labber_3        LKVQLAWPGN-IMQYINSYWKTKQLSPKRLSTGILMYT----LASSMCNQ
Panbuc_3        LKMRLAWPGN-IMQYFNSYWRTKQLSPKRLSTGILMYT----LASAMCEE
Parhas3         LRVQLAWPGN-IMKYINRYWKTRQLSPKRLSTGILMFT----LASSVCEQ
Cluhar_3        LKVQLAWPGN-IMQYVNRYWKNKQLSPKRLSTGILMYT----LASSLCDQ
Aloalo_3        LKVQLAWPGN-IMQYVNRYWKNKQLSPKRLSTGILMYT----LASSLCDQ
Panhyp_3        LRVQLAWPGN-IMQYVNRYWKTKQLSPKRLSTGILMFT----LASSLCEQ
Eleele_3        LRVQLAWPGN-IMTYINRYWKTRQLSPKRLSTGILMFT----LASSMCKQ
Denclu_3        LKVQLAWPGN-IMQYVNRYWKTKQLSPKRLSTGILMYT----LASSLCEQ
Sarpil_3        LKVQLAWPGN-IMQYVNRYWKNKQLSPKRLSTGILMYT----LASSLCDQ
Eigvir_3        LRVQLAWPGN-IMKYINRYWKTRQLSPKRLSTGILMFT----LASSVCEQ
Aptalb_3        LRVQLAWPGN-IMKYINRYWKTRQLSPKRLSTGILMFT----LASSVCEQ
Sinans_3        LKVQLAWPGN-IMQYVNRYWKTKQLSPKRLSTGILMFT----LASSLCEQ
Pygnat_3        LRVQLAWPGN-IMQYVNRYWKTKQLSPKRLSTGILMFT----LASSLCEQ
Astmex_3        LRVQLAWPGN-IMQYVNRYWKTKQLSPKRLSTGILMFT----LASSLCEQ
Caraur_3        LKVQLAWPGN-IMQYVNRYWKTKQLSPKRLSTGILMFT----LASSLCEQ
Danrer_3        LKVQLAWPGN-IMQYVNKYWKTKQLSPKRLSTGILMFT----LASSLCEQ
```

```
Ictpun_3        LRVQLAWPGN-IMQYVNRYWKTKQLAPKRLSTGILMFT----LASSLCDQ
Plealt_3        LKVQLAWPGN-IMQYVNKYWKTKQLSPKRLSTGILMYT----LASSMCDE
Plealt_9        LKIELAWPGN-IMQDVNKYWKTKNLSPKRLSTGILMYT----LASAMCEE
Gasacu_9        LKVELAWPGN-IMHDVNKYWKTKNLSPKRLSTGILMYT----LASAMCDE
Parkin_9        LNVQLAFPGN-IMHYINSYWKTKQLSPKRLSTGILMYT----LASAMCEE
Acapol_9        LKVELAWPGN-IMHDVNKYWKTKNLSPKRLSTGILMYT----LASAMCDE
Larcro_9        LKVELAWPGN-IMHDVNKYWKTKNLSPKRLSTGILMYT----LASAMCDE
Notcor_9        LKVELAWPGN-IMHDVNKYWKTKNLSPKRLSTGILMYT----LASAMCDE
Latcal_9        LKVELAWPGN-IMHDVNKYWKTKNLSPKRLSTGILMYT----LAYAMCDE
Serlaldor_9     LKVELAWPGN-IMHDVNKYWKTKNLSPKRLSTGILMYT----LAYAMCDE
Monalb_9        LKVDLAWPGN-IMHNINKYWKTKSLSPKRLSTGILMYT----LATAMCDE
Serdum_9        LKVELAWPGN-IMHDVNKYWKTKNLSPKRLSTGILMYT----LAYAMCDE
Perflu_9        LKIELAWPGN-IMHDVNKYWKTKNLSPKRLSTGILMYT----LASAMCDE
Perfla_9        LKIELAWPGN-IMHDVNKYWKTKNLSPKRLSTGILMYT----LASAMCDE
Stepar_9        LKVELAWPGN-IMHDVNKYWKTKNLSPKRLSTGILMYT----LASAMCDE
Molmol_9        LKVELAWPGN-IMHDVNKYWKTKNLSPKRLSTGILMYT----LASAMCDE
Hipcom_9        LKVELAWPGN-IMHDVNKYWKTKNLSPKRLSTGILMYT----LASAMCDE
Takrub_9 B      LKVELAWPGN-IMHDVNKYWKTKNLSPKRLSTGILMYT----LASAMCDE
Takrub_9        LKVELAWPGN-IMHDVNKYWKNKNLSPKRVSTGIFMYT----LASAMCDE
Cynsem_9        LKVKLAWPGN-IMHDVNKYWKTKNLSPKRLSTGILMYT----LAYAMCDE
Scomax_9        LKVKLAWPGN-IMHDVNKYWKTKNLSPKRLSTGILMYT----LAYAMCDE
Corlav_9        LKIELAWPGN-IMQDVNKYWKTKNLSPKRLSTGILMYT----LASAMCEE
Oncmyk_9        LKIELAWPGN-IMQDVNKYWKTKNLSPKRLSTGILMYT----LASAMCEE
Salsal_9        LKIELAWPGN-IMQDVNKYWKTKNLSPKRLSTGILMYT----LASAMCEE
Onctsh_9        LKIELAWPGN-IMQDVNKYWKTKNLSPKRLSTGILMYT----LASAMCEE
Salalp_9        LKIELAWPGN-IMQDVNKYWKTKNLSPKRLSTGILMYT----LASAMCEE
Ampoce_9        LKVELAWPGN-IMHDVNKYWKTKNLSPKRLSTGILMYT----LASAMCDE
Ampper9         LKVELAWPGN-IMHDVNKYWKTKNLSPKRLSTGILMYT----LASAMCDE
Labber_9        LKVELAWPGN-IMHDVNKYWKTKNLSPKRLSTGILMYT----LASAMCDE
Umbpyg_3B       LKVKLAWPGN-IMHYVNRYWKTKHLSPKRLSTGILMYT----LASAMCEE
Esoluc_3b       LKVQLAWPGN-IMHYINRYWKTKQLSPKRLSTGILMYT----LASAMCEE
Gadmor_9        LKIELAWPGN-IMQDVNKYWKTKNLSPKRLSTGILMYT----LASAMCEE
Umbpyg_9        LKVELAWPGN-IMQDVNKYWKTKNLSPKRLSTGILMYT----LASAMCEE
Esoluc_9        LKVELAWPGN-IMQDVNKYWKTKNLSPKRLSTGILMYT----LASAMCEE
Permag_9        LKVELAWPGN-IMHDVNKYWRTKNLSPKRLSTGILMYT----LASAMCDE
Bolpec_9        LKVELAWPGN-IMHDVNKYWRTKNLSPKRLSTGILMYT----LASAMCDE
Poefor9         LKVELAWPGN-IMHDVNKYWKTKNLSPKRLSTGILMYT----LASAMCDE
Masarm_9        LKVELAWPGN-IMHDVNKYWKTKNLSPKRLSTGILMYT----LASAMCDE
Angjap_9        LKVQLAWPGN-IMQYVNRYWKTRQLSPKRLSTGILMYT----LASAMCEE
Poelat_9        LKVELAWPGN-IMHDVNKYWKTKNLSPKRLSTGILMYT----LASAMCDE
Poeret_9        LKVELAWPGN-IMHDVNKYWKTKNLSPKRLSTGILMYT----LASAMCDE
Xipmac_9        LKIELAWPGN-IMHDVNKYWKTKNLSPKRLSTGILMYT----LASAMCDE
Hapbur_9        LKIELAWPGN-IMHDVNKYWKTKNLSPKRLSTGILMYT----LASAICDE
Orylat_9        LKVELAWPGN-IMHNVNKYWKTKNLSPKRLSTGILMYT----LAFSMCDE
Gamaff_9        LKVELAWPGN-IMHDVNKYWKTKNLSPKRLSTGILMYT----LASAMCDE
Neobri_9        LKIELAWPGN-IMHDVNKYWKTKNLSPKRLSTGILMYT----LASAICDE
Funhet_9        LKVELAWPGN-IMHDVNKYWKTKNLSPKRLSTGILMYT----LAYAMCDE
Punnye_9        LKIELAWPGN-IMHDVNKYWKTKNLSPKRLSTGILMYT----LASAICDE
Cypvar_9        LKVELAWPGN-IMHDVNKYWKTKNLSPKRLSTGILMYT----LAYAMCDE
Krymar_9        LKVELAWPGN-IMHDVNKYWKTKNLSPKRLSTGILMYT----LASAMCDE
Notfur_9        LKVELAWPGN-IMHDVNKYWKTKNLSPKRLSTGILMYT----LASAMCDE
Notpie_9        LKVELAWPGN-IMHDVNKYWKTKNLSPKRLSTGILMYT----LASAMCDE
Orenil_9        LKIELAWPGN-IMHDVNKYWKTKNLSPKRLSTGILMYT----LASAICDE
Auslim_9        LKVELAWPGN-IMQDVNKYWKTKNLSPKRLSTGILMYT----LASAMCDE
Anates_9        LKVELAWPGN-IMHDVNKYWKTKNLSPKRLSTGILMYT----LAYAMCDE
Astcal_9        LKIELAWPGN-IMHDVNKYWKTKNLSPKRLSTGILMYT----LASAICDE
Mayzeb_9        LKIELAWPGN-IMHDVNKYWKTKNLSPKRLSTGILMYT----LASAICDE
Orymel_9        LKVELAWPGN-IMHNVNKYWKTKNLSPKRLSTGILMYT----LAFAMCDE
Ampcit9         LKIELAWPGN-IMHDVNKYWKTKNLSPKRLSTGILMYT----LASAICDE
Macfas_2        VNVRTAYPSLRLLHAVRGYWLTNKVHIKRPTTGLLMYT----LATRFCNQ
Macmul_2        VNVRTAYPSLRLLHAVRGYWLTNKVHIKRPTTGLLMYT----LATRFCNQ
Macnem_2        VNVRTAYPSLRLLHAVRGYWLTNKVHIKRPTTGLLMYT----LATRFCNQ
Musmus_2        VNVRTAYPSLRLLHAVRGYWLTNKVHIKRPTTGLLMYT----LATRFCNQ
Ponabe_2        VNVRTAYPSLRLLHAVRGYWLTNKVHIKRPTTGLLMYT----LATRFCNE
Papanu_2        VNVRTAYPSLRLLHAVRGYWLTNKVHIKRPTTGLLMYT----LATRFCNQ
Nomleu_2        VNVRTAYPSLRLLHAVRGYWLTNKVHIKRPTTGLLMYT----LATRFCNQ
Pantro_2        VNVRTAYPSLPLLHAVRGYWLTNKVHIKRPTTGLLMYT----LATRFCNQ
Latcha_2        INVQTAYPSLRLLHAVRGYWLTNKVLIKRPSTGLLMYT----LATRFCSE
Gnapet2         LNVRPAFPSLRLLDAVRGYWLTNKVQIKRPTTGLLMYT----MATRFCDE
Panbuc_2b       VHVRPAFPSLRLLHAVRGYWLTNNVHIKRPTTGLLMYT----LATRFCDQ
Ostbic_2        INVHTAFPSLRLLHAVRGYWLTNKVQIKRPTTGLLMYT----MATRFCEQ
Sinrhi2         VNVRTAFPSLRLLHAVRGYWLTNHVQIKRPTTGLLMYT----MATRFCDE
Hetzeb_2        IRVQTAYPSLRLLHAVRGYWLTNRILIKRPTTGLLMYT----LATRFCDE
Hipcom_2        ADVRTAFPSLRLLHAVRGYWLTNKVHIKRPTTGLLMYT----MATRFCEE
Prigla_2        IHVQTAYPSLRFLHAVRGYWLTNRVLIKRPTTGLLMYT----LATRFCDE
Scytor_2        IHVQTAYPSLRFLHAVRGYWLTNRVLIKRPTTGLLMYT----LATRFCDE
Tetcal_2        IHVQTAYPSLRLLHAVRGYWLTNRVFIKRPTTGLLMYT----LATRFCDE
```

```
Rhityp_2        LHVQTAYPSLRLLHAVRGYWLTNKVLIKRPTTGLLMYT----LATRFCDE
Parkin_2b       LAVRPAFPSLRLLDAVRGYWLTNKVHIKRPTTGLLMYT----MATRFCEE
Angang_2        LNVRTAFPSLRLLHAVRGYWLTNKVLIKRPTTGLLMYT----MATRFCDE
Pygnat_2        VNVHTAFPSLRLLHAVRGYWLTNNVQIKRPTTGLLMYT----MATRFCDE
Parkin_2        LDVRPAFPSLRLLDAVRGYWLTNKVQIKRPTTGLLMYT----MATRFCDE
Gorgor_2        VNVRTAYPSLRLLHAVRGYWLTNKVHIKRPTTGLLMYT----LATRFCNQ
Thegel_2        VNVRTAYPSLRLLHAVRGYWLTNKVHIKRPTTGLLMYT----LATRFCNQ
Tupchi_2        VNVRTAYPSLRLLHAVRGYWLTNKVHIKRPTTGLLMYT----LATRFCNQ
Galgal_2        INVRTAYPSLRLLHAVRGYWLTNKVHIKRPTTGLLMYT----LATRFCNR
Eleele_2        GNVHAAFPSLRLLHAVRGYWLTNNIQIKRPTTGLLMYT----MATRFCDE
Danrer_2        VNVRTAFPSLRLLHAVRGYWLTNHVQIKRPTTGLLMYT----MATRFCDE
Canlupfam_2     VNVRTAYPSLRLLHAVRGYWLTNKVHIKRPTTGLLMYT----LATRFCNQ
Aloalo_2        VNVRTAFPSLRLLHAVRGYWLTNQVQIKRPTTGLLMYT----MATRFCDE
Astmex_2        VSVHAAFPSLRLLHAVRGYWLTNNIQIKRPTTGLLMYT----MATRFCEE
Sinans_2        VNVRTAFPSLRLLHAVRGYWLTNHVQIKRPTTGLLMYT----MATRFCDE
Sclfor_2        INVHTAFPSLRLLHAVRGYWLTNNVQIKRPTTGLLMYT----MATRFCEQ
Ceraty_2        VNVRTAYPSLRLLHAVRGYWLTNKVHIKRPTTGLLMYT----LATRFCNQ
Anocar_2        INVRTAYPSLRLLHAVRGYWLTNKVYIKRPTTGLLMYT----LATRFCNR
Anates_2        VDVRTAFPSLRLLHAVRGYWLTNNVHIKRPTTGLLMYT----MATRFCEE
Ampbic_2        VDVRTAFPSLRLLHAVRGYWLTNNVHIKRPTTGLLMYT----MATRFCEE
Sarpil_2        VNVRTAFPSLRLLHAVRGYWLTNQVQIKRPTTGLLMYT----MATRFCDE
Xenlae_2        INVHTAYPSLRLLHAVRGYWLTNKVHIKRPTTGILMYT----LATRFCNR
Cluhar_2        VNVRTAFPSLRLLHAVRGYWLTNQVQIKRPTTGLLMYT----MATRFCDE
Cypcar_2        VNVRTAFPSLRLLHAVRGYWLTNHVQIKRPTTGLLMYT----MATRFCDE
Denclu_2        VNVRTAFPSLRLLHAVRGYWLTNQVLIKRPTTGLLMYT----MATRFCEE
Notpie_2        VDVRMAFPSLRLLHAVRGYWLTNNVHIKRPTTGLLMYT----MATRFCEE
Erpcal2         INVQTAYPSLRLLYAVRGYWLTNKVPIKRPSTGLLMYT----LATRFCDE
Amical_2        INVRTAFPSLRLLHAVRGYWLTNKVPIKRPTTGMLMYT----LATRFCEE
Lepocu_2        INVRTAFPSLRLLHAVRGYWLTNKVPIKRPTTGLLMYT----LATRFCEE
Homsap_2        VNVRTAYPSLRLLHAVRGYWLTNKVHIKRPTTGLLMYT----LATRFCKQ
Bostau_2        VNVRTAYPSLRLLHAVRGYWLTNKVHIKRPTTGLLMYT----LATRFCNQ
Caraur_2        VNVRTAFPSLRLLHAVRGYWLTNQVQIKRPTTGLLMYT----MATRFCDE
Thythy_2B       VDVHTAFPSLRLLHAVRGYWLTNNVQIKRPTTGLLMYT----MATRFCEE
Onctsh_2B       VDVHTAFPSLRLLHAVRGYWLTNNVQIKRPTTGLLMYT----MATRFCEE
Salsal_2B       VDVHTAFPSLRLLHAVRGYWLTNNVQIKRPTTGLLMYT----MATRFCEE
Oncmyk_2B       VDVHTAFPSLRLLHAVRGYWLTNNVQIKRPTTGLLMYT----MATRFCEE
Onckis_2B       VDVHTAFPSLRLLHAVRGYWLTNNVQIKRPTTGLLMYT----MATRFCEE
Salsal_2A       VDVHTAFPSLRLLHAVRGYWLTNNVQIKRPTTGLLMYT----MATRFCEE
Thythy_2A       VDVHTAFPSLRLLHAVRGYWLTNNVQIKRPTTGLLMYT----MATRFCEE
Salalp_2A       VDVHTAFPSLRLLHAVRGYWLTNNVQIKRPTTGLLMYT----MATRFCEE
Corlav_2A       VDVHTAFPSLRLLHAVRGYWLTNNVQIKRPTTGLLMYT----MATRFCEE
Cormar_2A       VDVHTAFPSLRLLHAVRGYWLTNNVQIKRPTTGLLMYT----MATRFCEE
Onctsh_2A       VDVHTAFPSLRLLHAVRGYWLTNNVQIKRPTTGLLMYT----MATRFCEE
Oncmyk_2A       VDVHTAFPSLRLLHAVRGYWLTNNVQIKRPTTGLLMYT----MATRFCEE
Neobri_2        VDIRTAFPSLRLLHAVRGYWLTNNVHIKRPTTGLLMYT----MATRFCEE
Takrub_2        VDVRTAFPSLRLLHAVRGYWLTNNVHIKRPTTGLLMYT----MATRFCEE
Tetnig_2        VDVRTAFPSLRLLHAVRGYWLTNNVHIKRPTTGLLMYT----MATRFCEE
Orenil_2        VDIRTAFPSLRLLHAVRGYWLTNNVHIKRPTTGLLMYT----MATRFCEE
Orylat_2        VDVRTAFPSLRLLHAVRGYWLTNNVHIKRPTTGLLMYT----MATRFCEE
Krymar_2        VDVRMAFPSLRLLHAVRGYWLTNNVHIKRPTTGLLMYT----MATRFCEE
Notfur_2        VDVRMAFPSLRLLHAVRGYWLTNNVHIKRPTTGLLMYT----MATRFCEE
Poelat_2        VDVRTAFPSLRLLHAVRGYWLTNNVHIKRPTTGLLMYT----MATRFCEE
Gadmor_2        VGIRTAFPSLRLLHAVRGYWLTNNVQIKRPTTGLLMYT----MATRFCEE
Xipmac_2        VDVRTAFPSLRLLHAVRGYWLTNNVHIKRPTTGLLMYT----MATRFCEE
Poeret_2        VDVRTAFPSLRLLHAVRGYWLTNNVHIKRPTTGLLMYT----MATRFCEE
Astcal_2        VDIRTAFPSLRLLHAVRGYWLTNNVHIKRPTTGLLMYT----MATRFCEE
Hapbur_2        VDIRTAFPSLRLLHAVRGYWLTNNVHIKRPTTGLLMYT----MATRFCEE
Acapol_2B       VDVRTAFPSLRLLHAVRGYWLTNNVHIKRPTTGLLMYT----MATRFCEE
Ampoce_2A       VDVRTAFPSLRLLHAVRGYWLTNNVHIKRPTTGLLMYT----MATRFCEE
Acapol_2A       VDVRTAFPSLRLLHAVRGYWLTNNVHIKRPTTGLLMYT----MATRFCEE
Mayzeb_2        VDIRTAFPSLRLLHAVRGYWLTNNVHIKRPTTGLLMYT----MATRFCEE
Punnye_2        VDIRTAFPSLRLLHAVRGYWLTNNVHIKRPTTGLLMYT----MATRFCEE
Latcal_2        VDVRTAFPSLRLLHAVRGYWLTNNVHIKRPTTGLLMYT----MATRFCEE
Singra_2        VNVRTAFPSLRLLHAVRGYWLTNHVQIKRPTTGLLMYT----MATRFCDE
Auslim_2        VDVRMAFPSLRLLHAVRGYWLTNNVHIKRPTTGLLMYT----MATRFCEE
Monalb2         VDVRTAFPSLRLLHAVRGYWLTNNVHIKRPTTGLLMYT----LATRFCEE
Ampoce_2B       VDVRTAFPSLRLLHAVRGYWLTNNVHIKRPTTGLLMYT----MATRFCEE
Chiham_2        VDVRTAFPSLRLLHAVRGYWLTNHVHIKRPTTGLLMYT----MATRFCDE
Gasacu_2        VDVRTAVPSLRLLHAVRGYWLTNNVHIKRPTTGLLMYT----MATRFCEE
Gymacu_2        VDVRTAFPSLRLLHAVRGYWLTNHVHIKRPTTGLLMYT----MATRFCDE
Labber_2        VDVRTAFPSLRLLHAVRGYWLTNNVHIKRPTTGLLMYT----MATRFCEE
Cypvar_2        VDVRTAFPSLRLLHAVRGYWLTNNVHIKRPTTGLLMYT----MATRFCEE
Funhet_2        VDVRTAFPSLRLLHAVRGYWLTNNVHIKRPTTGLLMYT----MATRFCEE
Larcro_2        VDVRTAFPSLRLLHAVRGYWLTNNVHIKRPTTGLLMYT----MATRFCEE
Paroli_2        VDVRTAFPSLRLLHAVRGYWLTNNVHIKRPTTGLLMYT----MATRFCEE
Perflu_2        VDVRTAFPSLRLLHAVRGYWLTNNVHIKRPTTGLLMYT----MATRFCEE
Serdum_2        VDVRTAFPSLRLLHAVRGYWLTNNVHIKRPTTGLLMYT----MATRFCEE
```

```
Stepar_2        VDVRTAFPSLRLLHAVRGYWLTNNVHIKRPTTGLLMYT----MATRFCEE
Notcor_2        VDVRTAFPSLRLLHAVRGYWLTNHVHIKRPTTGLLMYT----MATRFCDE
Treber_2        VDVRTAFPSLRLLHAVRGYWLTNHVHIKRPTTGLLMYT----MATRFCDE
Serlaldor_2     VDVRTAFPSLRLLHAVRGYWLTNNVHIKRPTTGLLMYT----MATRFCEE
Plaste_2        VDVRTAFPSLRLLHAVRGYWLTNNVHIK-PTTGLLMYT----MATRFCDE
Clabat_4        LPVRMAYPSLRLVHAVRGYWLTNKVNIKRPSTGLLMYT----MATRFCDE
Ictpun_4A       LPVHMAYPSLRLVHAVRGYWLTNKVNIKRPSTGLLMYT----MATRFCDE
Panhyp_4        LPVRMAYPSLRLVHAVRGYWLTNKVNIKRPSTGLLMYT----MATRFCDE
Umbpyg_4        LSVRTAYPSLRLIHAVRGYWLTNKINIKRPSTGLLMYT----LATRFCDE
Esoluc_4        LRVRTAYPSLRLIHAVRGYWLTNKINIKRPSTGLLMYT----LATRFCDE
Plealt_4        LRVKTAYPSLRLVHAVRGYWLTNKINIKRPSTGLLMYT----LATRFCNE
Onckis_4B       LRVRTAYPSLRLIHVVRGYWLTNKINIKRPSTGLLMYT----LATRFCDE
Onckis_4A       LRVRTAYPSLRLIHVVRGYWLTNKINIKRPSTGLLMYT----LATRFCDE
Onctsh_4        LRVRTAYPSLRLIHVVRGYWLTNKINIKRPSTGLLMYT----LATRFCDE
Oncmyk_4        LRVRTAYPSLRLIHVVRGYWLTNKINIKRPSTGLLMYT----LATRFCDE
Corlav_4        LRVRTAYPSLRLIHAVRGYWLTNKINIKRPSTGLLMYT----LATRFCDE
Astmex_4A       LPLRTAYPSLRLIHAVRGYWLTNKVNIKRPSTGLLMYT----MATRFCDE
Pygnat_4        LPVHMAYPSLRLIHAVRGYWLTNKVNIKRPSTGLLMYT----MATRFCDE
Aloalo_4        LRVRTAYPSLRLIHAVRGYWLTNRIHIKRPSTGLLMYT----LATRFCDE
Cluhar_4        LSVKPAYPSLRLIHAVRGYWLTNKIHIKRPSTGLLMYT----LATRFCDE
Denclu_4        LHLRMAYPSLRLFHAVRGYWLTNKVNIKRPSTGLLMYT----LATRFCDE
Konpun4         LRVRTAYPSLRLIHAVRGYWLTNKIHIKRPSTGLLMYT----LATRFCDE
Sarpil_4        LRVRTAYPSLRLIHAVRGYWLTNRIHIKRPSTGLLMYT----LATRFCDE
Caraur_4        LKVRTAYPSLRLIHAVRGYWLTNKINIKRPSTGLLMYT----MATRFCDE
Cteide_4        LKVRMAYPSLRLIHAVRRYWLTNKINIKRPTTGLLMYT----MATRFCDE
Petmar_4        LPLRAAFPSLRLMHAVRGYWLTNKVYIKRPTTGLLMYT----LATRFCRE
Cypcar_4        LRLRTAYPSLRLIHAVRGFWLTNKINIKRPSTGLLMYT----LATRFCDE
Danrer_4        LKVRTAYPSLRLIHAVRGFWLTNKINIKRPTTGLLMYT----MATRFCDE
Misang_4        LKVRTAYPSLRLIHAVRGYWLTNKIKIKRPSTGLLMYT----MFTRFCDE
Angjap_4        LRVRTAYPSLRLIHAVRGYWLTNKVNIKRPSTGLLMYT----LATRFCDE
Cypcar_4A       LRLRTAYPSLRLIHAVRGFWLTNKINIKRPSTGLLMYT----LATRFCDE
Angang_4        LRVRPPTPRSGLIHAVRGYWLTNKVNIKRPSTGLLMYT----LATRFCDE
Cypcar_4B       LKVRTAYPSLRLIHAVRGYWLTNKINIKRPSTGLLMYT----LATRFCDE
Sinans_4A       LKVRTAYPSLRLIHAVRGYWLTNKINIKRPSTGLLMYT----MATRFCDE
Sinans_4B       LKVRTAYPSLRLIHAVRGYWLTNKINIKRPSTGLLMYT----MATRFCDE
Singra_4A       LKVRTAYPSLRLIHAVRGYWLTNKINIKRPSTGLLMYT----MATRFCDE
Aptalb_4        LPVRMAYPSLRLVHAVRGYWLTNKVNIKRPSTGLLMYT----MATRFCDE
Eigvir_4        LPVHMAYPSLRLIHAVRGYWLTNKVNIKRPSTGLLMYT----IATRFCDE
Eleele_4        LPVRMAYPSLRLIHAVRGYWLTNKVNIKRPSTGLLMYT----MATRFCDE
Parhas_4        LPVRMAYPSLRLVHAVRGYWLTNKVNIKRPSTGLLMYT----MATRFCDE
Sclfor_4        LKLRTAYPSLRLIHAVRGYWLTNKINIKRPSTGLLMYT----LATRFCDE
Gnapet4         LLLRTAYPSLRLIHAVRGYWLTNKVNIKRPSTGLLMYT----LATRFCNE
Ostbic_4        LRVRTAYPSLRLNHAVRGYWLTNKIKIKRPSTGLLMYT----LATRFCDE
Parkin_4        LLLRTAYPSLRLIHAVRGYWLTNKVNIKRPSTGLLMYT----LATRFCNE
Panbuc_4        LKVRTAYPSLRLIHAVRGYWLTNKVNIKRPSTGLLMYT----LATRFCDE
Erpcal4         LKVRTAYPSLRLIHAVRGYWLTNKINIKRPSTGLLMYT----LATRFCDE
Salsal_4        LRVRTAYPSLRLIHVVRGYWLTNKINIKRPSTGLLMYT----LATRFCDE
Saltru_4        LRVRTAYPSLRLIHVVRGYWLTNKINIKRPSTGLLMYT----LATRFCDE
Salalp_4        LRVRTAYPSLRLIHVVRGYWLTNKINIKRPSTGLLMYT----LATRFCDE
Salfon_4        LRVRTAYPSLRLIHVVRGYWLTNKINIKRPSTGLLMYT----LATRFCDE
Thythy_4        LSVRTAYPSLRLIHAVRGYWLTNKINIKRPSTGLLMYT----LATRFCDE
Calmil_4        LSIQTAYPSLRLIHAVRGYWLTNKVYIKRPSTGLLMYT----LATRFCDE
Hetzeb_4        LSIQTAYPSLRLIHAVRGYWLTNKIYIKRPSTGLLMYT----LATRFCDE
Rhityp_4        LNIQTAYPSLRLIHAVRGYWLTNKIYIKRPSTGLLMYT----LATRFCDE
Scytor_4        LNIQTAYPSLRLIHAVRGYWLTNKIYIKRPSTGLLMYT----LSTRFCDE
Squaca_4        LSIQTAYPSLRLIHAVRGYWLTNKVYIKRPSTGLLMYT----LATRFCDE
Amical_4        LKVRTAYPSLRLIHAVRGYWLTNKINIKRPSTGLLMYT----LATRFCDE
Lepocu_4        LKVRTAYPSLRLIHAVRGYWLTNKINIKRPSTGLLMYT----LATRFCDE
Agema_4         LPVRMAYPSLRLVHAVRGYWLTNKVNIKRPSTGLLMYT----MATRFCDE
Anocar_4        LKVRTAYPSLRLIHAVRGYWLTNKVFIKRPSTGLLMYT----LATRFCDE
Canlupfam_4     LKVRTAYPSLRLIHAVRGYWLTNKVPIKRPSTGLLMYT----LATRFCDE
Crigri_4        LKVRTAYPSLRLIHAVRGYWLTNKVPIKRPSTGLLMYT----LATRFCDE
Galgal_4        LKVRTAYPSLRLIHAVRGYWLTNKVHIKRPSTGLLMYT----LATRFCDE
Mesaur_4        LKVRTAYPSLRLIHAVRGYWLTNKVPIKRPSTGLLMYT----LATRFCDE
Homsap_4        LKVRTAYPSLRLIHAVRGYWLTNKVPIKRPSTGLLMYT----LATRFCDE
Notscuscu_4     LKVQTAYPSLRLIHAVRGYWLTNKVYIKRPSTGLLMYT----LATRFCDE
Psetextex_4     LKVRTAYPSLRLIHAVRGYWLTNKVYIKRPSTGLLMYT----LATRFCDE
Musmus_4        LQVRTAYPSLRLIHAVRGYWLTNKVPIKRPSTGLLMYT----LATRFCDE
Pogvit_4        LKVRTAYPSLRLIHAVRGYWLTNKVFIKRPSTGLLMYT----LATRFCDE
Promuc_4        LKVRTAYPSLRLIHAVRGYWLTNKVYIKRPSTGLLMYT----LATRFCDE
Pantro_4        LKVRTAYPSLRLIHAVRGYWLTNKVPIKRPSTGLLMYT----LATRFCDE
Pytbiv_4        LKVRTAYPSLRLIHAVRGYWLTNKVYIKRPSTGLLMYT----LATRFCDE
Ratnor_2        VNVRTAYPSLRLLHAVRGYWLTNKVHIKRPTTGLLMYT----LATRFCNQ
Ratnor_4        LKVRTAYPSLRLIHAVRGYWLTNKVPIKRPITGLLMYT----LATRFCDE
Siltro_2        INVHTAYPSLRLLHAVRGYWLTNKVHIKRPTTGILMYT----LATRFCNR
Siltro_4        LKVRTAYPSLRLIHAVRGYWLTNKVQIKRPSTGLLMYT----LATRFCDE
Susscr_4        LKVRTAYPSLRLIHAVRGYWLTNKVPIKRPSTGLLMYT----LATRFCDE
```

```
Taegut_4        LKVRTAYPSLRLIHAVRGYWLTNKVHIKRPSTGLLMYT----LATRFCDE
Termextri_4M    LKVRTAYPSLRLIHAVRGYWLTNKVHIKRPSTGLLMYT----LTTRFCDE
Thasirsir_4     LKVRTAYPSLRLIHAVRGYWLTNKVYIKRPSTGLLMYT----LATRFCDE
Xenlae_4B       LKVRTAYPSLRLIHAVRGYWLTNKVQIKRPSTGLLMYT----LATRFCDE
Xenlae_4A       LKVRTAYPSLRLIHAVRGYWLTNKVQIKRPSTGLLMYT----LATRFCDE
                201
Latcal_3        IHLYG----FWPFGWDPNTG---KELPYHYYDKKGTKFTTKWQESHQLPA
Perflu_3        IHLYG----FWPFGWDPNTG---KELPYHYYDKKGTKFTTKWQESHQLPA
Serdum_3        IHLYG----FWPFGWDPNTG---KELPYHYYDKKGTKFTTKWQESHQLPA
Stepar_3        IHLYG----FWPFGWDPNTG---KELPYHYYDKKGTKFTTKWQESHQLPA
Tetcal_3        IHLYG----FWPFAWDPNTG---KELPYHYYDKKGTKFTTKWQESHQLPA
Monalb_3        IHLYG----FWPFGWDPNTG---KELPYHYYDKKGTKFTTKWQESHQLPA
Molmol_3        IHLYG----FWPFGWDPNTG---KELPYHYYDKKGTKFTTKWQESHQLPA
Takrub_3        IHLYG----FWPFGWDPNTG---KELPYHYYDKKGTKFTTKWQESHQLPT
Canlupfam_3     IHLYG----FWPFGFDPNTR---EDLPYHYYDKKGTKFTTKWQESHQLPA
Bostau_3        IHLYG----FWPFGFDPNTR---EDLPYHYYDKKGTKFTTKWQESHQLPA
Homsap_3        IHLYG----FWPFGFDPNTR---EDLPYHYYDKKGTKFTTKWQESHQLPA
Siltro_3        IHLYG----FWPFGWDPNTG---KDLPYHYYDKKGTKFTTKWQESHQLPA
Anocar_3        IHLYG----FWPFGFNPNNR---EDLPYHYFDKKGTKFTTKWQESHQLPA
Musmus_3        IHLYG----FWPFGFDPNTR---EDLPYHYYDKKGTKFTTKWQESHQLPA
Pantro_3        IHLYG----FWPFGFDPNTR---EDLPYHYYDKKGTKFTTKWQESHQLPA
Ratnor_3        IHLYG----FWPFGFDPNTR---EDLPYHYYDKKGTKFTTKWQESHQLPA
Galgal_3        IHLYG----FWPFGFDPNTR---EDLPYHYYDKKGTKFTTKWQESHQLPA
Lepocu_3        IHLYG----FWPFGWDPNTG---KELPYHYYDKKGTKFTTKWQESHQLPT
Tetnig_3        IHLYG----FWPFGWDPNTG---RELPYHYYDKKGTKFTTKWQESHQLPT
Squaca_3        IHLYG----FWPFAWDPNTG---KELPYHYYDKKGTKFTTKWQESHQLPA
Perfla_3        IHLYG----FWPFGWDPNTG---KELPYHYYDKKGTKFTTKWQESHQLPA
Salsal_3        IHLYG----FWPFGWDPNTG---KELPYHYYDKKGTKFTTKWQESHQLPA
Serlaldor_3     IHLYG----FWPFGWDPNTG---KELPYHYYDKKGTKFTTKWQESHQLPA
Treber_3        IHLYG----FWPFGWDPNTG---NDLPYHYYDKKGTKFTTKWQETHQLPT
Cynsem_3        IHLYG----FWPFGWDPNTG---KELPYHYYDKKGTKFTTKWQESHQLPA
Paroli_3        IHLYG----FWPFGWDPNTG---KDLPYHYYDKKGTKFTTKWQETHQLPS
Paroli_3b       IHLYG----FWPFGWDPNTG---KELPYHYYDKKGTKFTTKWQESHQLPA
Plaste_3        IHLYG----FWPFGWDPNTG---NDLPYHYYDKKGTKFTTKWQETHQLPS
Plaste_3b       IHLYG----FWPFGWDPNTG---KELPYHYYDKKGTKFTTKWQESHQLPA
Erpcal3         IHLYG----FWPFGWDPNTG---KELPYHYYDKKGTKFTTKWQESHQLPT
Calmil_3b       IHLYG----FWPFAWDPNTG---RELPYHYYDKKGTKFNTNWQETHQLPA
Hetzeb_3        IHLYG----FWPFAWDPNTG---KELPYHYYDKKGTKFTTKWQESHQLPA
Latcha_3        IHLYG----FWPFGWDPNSG---KDLPYHYYDKKGTKFTTKWQESHQLPA
Anates_3        IHLYG----FWPFGWDPNTG---KELPYHYYDKKGTKFTTKWQESHQLPA
Ampcit3         IHLYG----FWPFGWDPNTG---KELPYHYYDKKGTKFTTKWQESHQLPA
Auslim_3        VHLYG----FWPFGWDPNTG---KELPYHYYDRKGTKFTTKWQESHQLPA
Cypvar_3        IHLYG----FWPFGWDPNTG---KELPYHYYDKKGTKFTTKWQESHQLPA
Krymar_3        VHLYG----FWPFGWDPNTG---KELPYHYYDRKGTKFTTKWQESHQLPA
Funhet_3        IHLYG----FWPFGWDPNTG---KELPYHYYDKKGTKFTTKWQESHQLPA
Gamaff3         IHLYG----FWPFGWDPNTG---KELPYHYYDKKGTKFTTKWQESHQLPA
Orylat_3        IHLYG----FWPFGWDPNTG---KELPYHYYDRKGTKFTTKWQESHQLPA
Orenil_3        IHLYG----FWPFGWDPNTG---KELPYHYYDKKGTKFTTKWQESHQLPA
Mayzeb_3        IHLYG----FWPFGWDPNTG---KELPYHYYDKKGTKFTTKWQESHQLPA
Punnye_3        IHLYG----FWPFGWDPNTG---KELPYHYYDKKGTKFTTKWQESHQLPA
Punnye_3a       IHLYG----FWPFGWDPNTG---KELPYHYYDKKGTKFTTKWQESHQLPA
Neobri_3        IHLYG----FWPFGWDPNTG---KELPYHYYDKKGTKFTTKWQESHQLPA
Misang_3        VHLYG----FWPFGWDPNTG---KELPYHYYDKKGTKFTTKWQESHQLPT
Sinrhi3         VHLYG----FWPFGWDPNTG---KELPYHYYDKKGTKFTTKWQESHQLPT
Singra_3        VHLYG----FWPFGWDPNTG---KELPYHYYDKKGTKFTTKWQESHQLPT
Hapbur_3        IHLYG----FWPFGWDPNTG---KELPYHYYDKKGTKFTTKWQESHQLPA
Astcal_3        IHLYG----FWPFGWDPNTG---KELPYHYYDKKGTKFTTKWQESHQLPA
Notfur_3        IHLYG----FWPFGWDPNTG---KELPYHYYDRKGTKFTTKWQESHQLPA
Cypcar_3        --------------------------------------------------
Poefor3         IHLYG----FWPFGWDPNTG---KELPYHYYDKKGTKFTTKWQESHQLPA
Poelat_3        IHLYG----FWPFGWDPNTG---KELPYHYYDKKGTKFTTKWQESHQLPA
Poeret_3        VHLYG----FWPFGWDPNTG---KELPYHYYDKKGTKFTTKWQESHQLPA
Angang_3        IHLYG----FWPFGWDPNTG---KELPYHYYDKKGTKFTTKWQESHQLPS
Xipmac_3        IHLYG----FWPFGWDPNTG---KELPYHYYDKKGTKFTTKWQESHQLPA
Masarm_3        IHLYG----FWPFGWDPNTG---KELPYHYYDKKGTKFTTKWQESHQLPA
Permag_3        --------------------------LPYHYYDKKGTKFTTKWQESHQLPA
Gnapet3         VHLYG----FWPFGWDPSTG---RELPYHYYDRKGTKFTTKWQESHQLPA
Ostbic_3        VHLYG----FWPFGWDPNTG---KELPYHYYDRKGTKFTTKWQETHELPT
Gadmor_3        IHLYG----FWPFGWDPNTG---KELPYHYYDKKGTKFTTKWQESHQLPA
Sclfor_3        VHLYG----FWPFGWDPNTG---KELPYHYYDRKGTKFTTKWRESHELPS
Acapol3         IHLYG----FWPFGWDPNTG---KELPYHYYDKKGTKFTTKWQESHQLPA
Ampoce_3        IHLYG----FWPFGWDPNTG---KELPYHYYDKKGTKFTTKWQESHQLPA
Sclfor_3b       IHLYG----FWPFAWDPISG---KGLPYHYYDKKGTKFTTKWQESHQLPA
Ostbic_3b       IHLYG----FWPFGWDPISG---KGLPYHYYDKKGTKFTTKWQESHQLPA
Parkin_3        VHLYG----FWPFGWDPSTG---RELPYHYYDRKGTKFTTKWQESHQLPA
Ampper3         IHLYG----FWPFGWDPNTG---KELPYHYYDKKGTKFTTKWQESHQLPA
Oncmyk_3        IHLYG----FWPFGWDPNTG---KELPYHYYDKKGTKFTTKWQESHQLPA
```

```
Gasacu_3       IHLYG----FWPFGWDPNTG---KELPYHYYDKKGTKFTTKWQESHQLPA
Labber_3       IHLYG----FWPFGWDPNTG---KELPYHYYDKKGTKFTTKWQESHQLPA
Panbuc_3       VHLYG----FWPFGWDPASG---RELPYHYYDKKGTKFTTKWQESHQLPA
Parhas3        VHLYG----FWPFGWDPNTG---KDLPYHYYDKKGTKFTTKWQESHQLPT
Cluhar_3       IHLYG----FWPFGWDPNTG---KELPYHYYDKKGTKFTTKWQESHQLPT
Aloalo_3       IHLYG----FWPFGWDPNTG---KELPYHYYDKKGTKFTTKWQESHQLPT
Panhyp_3       VHLYG----FWPFGWDPNTG---KELPYHYYDKKGTKFTTKWQESHQLPT
Eleele_3       VHLYG----FWPFGWDPNTG---KELPYHYYDKKGTKFTTKWQESHQLPT
Denclu_3       IHLYG----FWPFGWDPNTG---KELPYHYYDKKGTKFTTKWQESHQLPT
Sarpil_3       IHLYG----FWPFGWDPNTG---KELPYHYYDKKGTKFTTKWQESHQLPT
Eigvir_3       VHLYG----FWPFGWDPNTG---KELPYHYYDKKGTKFTTKWQESHQLPT
Aptalb_3       VHLYG----FWPFGWDPNTG---KDLPYHYYDKKGTKFTTKWQESHQLPT
Sinans_3       VHLYG----FWPFGWDPNTG---KELPYHYYDKKGTKFTTKWQESHQLPT
Pygnat_3       IHLYG----FWPFGWDPNTG---KELPYHYYDKKGTKFTTKWQESHQLPT
Astmex_3       IHLYG----FWPFGWDPNTG---KELPYHYYDKKGTKFTTKWQESHQLPT
Caraur_3       VHLYG----FWPFGWDPNTG---KELPYHYYDKKGTKFTTKWQESHQLPT
Danrer_3       VHLYG----FWPFGWDPNTG---KELPYHYYDKKGTKFTTKWQESHQLPT
Ictpun_3       IHLYG----FWPFGWDPNTG---KELPYHYYDKKGTKFTTKWQESHQLPT
Plealt_3       IHLYG----FWPFGWDPNTG---KELPYHYYDKKGTKFTTKWQESHQLPA
Plealt_9       IHLYG----FWPFGWDPNTG---KELPYHYYDKKGTKFTTKWQETHQLPS
Gasacu_9       IHLYG----FWPFGWDPNTG---NDLPYHYYDKKGTKFTTKWQETHQLPS
Parkin_9       VHLYG----FWPFGWDPISG---KGLPYHYFDKKGTKFTTKWQESHQLPA
Acapol_9       IHLYG----FWPFGWDPNTG---KELPYHYYDKKGTKFTTKWQETHQLPS
Larcro_9       IHLYG----FWPFGWDPNTG---KDLPYHYYDKKGTKFTTKWQETHQLPS
Notcor_9       IHLYG----FWPFGWDPNTG---NDLPYHYYDKKGTKFTTKWQETHQLPT
Latcal_9       IHLYG----FWPFGWDPNTG---KELPYHYYDKKGTKFTTKWQETHQLPS
Serlaldor_9    IHLYG----FWPFGWDPNTG---KELPYHYYDKKGTKFTTKWQETHQLPS
Monalb_9       VHLYG----FWPFGWDPNTG---KELPYHYYDKKGTKFTTKWQEAHQLPS
Serdum_9       IHLYG----FWPFGWDPNTG---KELPYHYYDKKGTKFTTKWQETHQLPS
Perflu_9       IHLYG----FWPFGWDPNSG---KDLPYHYYDKKGTKFTTKWQETHQLPS
Perfla_9       IHLYG----FWPFGWDPNSG---KDLPYHYYDKKGTKFTTKWQETHQLPS
Stepar_9       IHLYG----FWPFGWDPNTG---KELPYHYYDKKGTKFTTKWQETHQLPS
Molmol_9       IHLYG----FWPFGWDPNTG---NDLPYHYYDKKGTKFTTKWQETHQLPS
Hipcom_9       IHLYG----FWPFGWDPNTG---KDLPYHYYDKKGTKFTTKWQETHQLPS
Takrub_9 B     IHLYG----FWPFGWDPNTG---KELPYHYYDKKGTKFTTKWQETHQLPT
Takrub_9       IHLYG----FWPFGWDPNTG---KELPYHYYDKKGTKFTTKWQETHQLPT
Cynsem_9       IHLYG----FWPFGWDPNTG---KDLPYHYYDKKGTKFTTKWQETHQLPS
Scomax_9       IHLYG----FWPFGWDPNTG---KDLPYHYYDKKGTKFTTKWQETHQLPS
Corlav_9       IHLYG----FWPFGWDPNTG---KELPYHYYDKKGTKFTTKWQETHQLPS
Oncmyk_9       IHLYG----FWPFGWDPNTG---KELPYHYYDKKGTKFTTKWQETHQLPS
Salsal_9       IHLYG----FWPFGWDPNTG---KELPYHYYDKKGTKFTTKWQESHQLPA
Onctsh_9       IHLYG----FWPFGWDPNTG---KELPYHYYDKKGTKFTTKWQETHQLPS
Salalp_9       IHLYG----FWPFGWDPNTG---KELPYHYYDKKGTKFTTKWQETHQLPS
Ampoce_9       IHLYG----FWPFGWDPNTG---KELPYHYYDKKGTKFTTKWQETHQLPS
Ampper9        IHLYG----FWPFGWDPNTG---KELPYHYYDKKGTKFTTKWQETHQLPS
Labber_9       IHLYG----FWPFGWDPNTG---KDLPYHYYDKKGTKFTTKWQETHQLPT
Umbpyg_3B      IHLYG----FWPFGWDPNTG---KELPYHYYDKKGTKFTTKWQESHQLPA
Esoluc_3b      IHLYG----FWPFGWDPNTG---KELPYHYYDRKGTKFTTKWQESHQLPA
Gadmor_9       IHLYG----FWPFGWDPNTG---KDLPYHYYDKKGTKFTTKWQETHQLPS
Umbpyg_9       IHLYG----FWPFGWDPNTG---KELPYHYYDKKGTKFTTKWQETHQLPS
Esoluc_9       IHLYG----FWPFGWDPNTG---KELPYHYYDKKGTKFTTKWQETHQLPS
Permag_9       IHLYG----FWPFGWDPNTG---KDLPYHYYDRKGTKFTTKWQETHQLPN
Bolpec_9       IHLYG----FWPFGWDPNTG---KDLPYHYYDRKGTKFTTKWQETHQLPN
Poefor9        IHLYG----FWPFGWDPNTG---KELPYHYYDKKGTKFTTKWQETHQLPS
Masarm_9       IHLYG----FWPFGWDPNTG---KELPYHYYDKKGTKFTTKWQETHQLPS
Angjap_9       IHLYG----FWPFGWDPNTG---KELPYHYYDKKGTKFTTKWQESHQLPS
Poelat_9       IHLYG----FWPFGWDPNTG---KELPYHYYDKKGTKFTTKWQETHQLPS
Poeret_9       IHLYG----FWPFGWDPNTG---KELPYHYYDKKGTKFTTKWQETHQLPS
Xipmac_9       IHLYG----FWPFGWDPNTG---KDLPYHYYDKKGTKFTTKWQETHQLPS
Hapbur_9       IHLYG----FWPFGWDPNTG---KDLPYHYYDKKGTKFTTKWQETHQLPS
Orylat_9       IHLYG----FWPFGWDPNTG---KDLPYHYYDKKGTKFTTKWQETHQLPS
Gamaff_9       IHLYG----FWPFGWDPNTG---KDLPYHYYDKKGTKFTTKWQETHQLPS
Neobri_9       IHLYG----FWPFGWDPNTG---KDLPYHYYDKKGTKFTTKWQETHQLPS
Funhet_9       IHLYG----FWPFGWDPNTG---KELPYHYYDKKGTKFTTKWQETHQLPS
Punnye_9       IHLYG----FWPFGWDPNTG---KDLPYHYYDKKGTKFTTKWQETHQLPS
Cypvar_9       IHLYG----FWPFGWDPNTG---KELPYHYYDKKGTKFTTKWQETHQLPS
Krymar_9       IHLYG----FWPFGWDPNTG---KELPYHYYDKKGTKFTTKWQETHQLPS
Notfur_9       IHLYG----FWPFGWDPNTG---KELPYHYYDKKGTKFTTKWQETHQLPS
Notpie_9       IHLYG----FWPFGWDPNTG---KELPYHYYDKKGTKFTTKWQETHQLPS
Orenil_9       IHLYG----FWPFGWDPNTG---KDLPYHYYDKKGTKFTTKWQETHQLPS
Auslim_9       IHLYG----FWPFGWDPNTG---KELPYHYYDKKGTKFTTKWQETHQLPS
Anates_9       IHLYG----FWPFGWDPNTG---KDLPYHYYDKKGTKFTTKWQETHQLPS
Astcal_9       IHLYG----FWPFGWDPNTG---KDLPYHYYDKKGTKFTTKWQETHQLPS
Mayzeb_9       IHLYG----FWPFGWDPNTG---KDLPYHYYDKKGTKFTTKWQETHQLPS
Orymel_9       IHLYG----FWPFGWDPNTG---KELPYHYYDKKGTKFTTKWQETHQLPS
Ampcit9        IHLYG----FWPFGWDPNTG---KDLPYHYYDKKGTKFTTKWQETHQLPS
Macfas_2       IYLYG----FWPFPLDQN-Q---NPVKYHYYDSLKYGYTSQASP-HTMPL
```

```
Macmul_2       IYLYG----FWPFPLDQN-Q---NPVKYHYYDSLKYGYTSQASP-HTMPL
Macnem_2       IYLYG----FWPFPLDQN-Q---NPVKYHYYDSLKYGYTSQASP-HTMPL
Musmus_2       IYLYG----FWPFPLDQN-Q---NPVKYHYYDSLKYGYTSQASP-HTMPL
Ponabe_2       IYLYG----FWPFPLDQN-Q---NPVKYHYYDSLKYGYTSQASP-HTMPL
Papanu_2       IYLYG----FWPFPLDQN-Q---NPVKYHYYDSLKYGYTSQASP-HTMPL
Nomleu_2       IYLYG----FWPFPLDQN-Q---NPVKYHYYDSLKYGYTSQASP-HTMPL
Pantro_2       IYLYG----FWPFPLDQN-Q---NPVKYHYYDSLKYGYTSQASP-HTMPL
Latcha_2       IHLYG----FWPFPRDHS-E---RPVKYHYYDSLTYEYTSQASP-HTMPL
Gnapet2        IHLYG----FWPFALGPQ-G---NPVKYHYYDSLTYQYTSQASP-HTMPL
Panbuc_2b      IHLYG----FWPFPRDVG-G---RPVKYHYYDALTYGYTSQAGP-HAMPL
Ostbic_2       IHLYG----FWPFPLGPH-G---NPVKYHYYDALTYEYTSQVGP-HTMPL
Sinrhi2        IHLYG----FWPFAHDPD-G---KSVKYHYYDTLTYHYTSGASP-HTMPL
Hetzeb_2       IHLYG----FWPFPKDQK-G---NVVKYHYYDSLTYEYNSRATP-HTMPL
Hipcom_2       IHLYG----FWPFSHDAA-G---KAVKYHYYDTLKYEYTSSSSP-HSMPL
Prigla_2       IHLYG----FWPFPKDQK-G---NVVKYHYYDSLTYEYNSRATP-HTMPL
Scytor_2       IHLYG----FWPFPKDQK-G---NAVKYHYYDSLTYEYNSRATP-HTMPL
Tetcal_2       IHLYG----FWPFSKDPK-G---NAVKYHYYDSLTYEYNSRATP-HTMPL
Rhityp_2       IHLYG----FWPFPKDHK-G---NAVKYHYYDSLTYEYNSRATP-HTMPL
Parkin_2b      IHLYG----FWPFSHDAQ-G---NAVKYHYYDSLTYGYTSRTSP-HTMPL
Angang_2       IHLYG----FWPFPQDPQ-G---NPMKYHYYDTLTYEYDSHSSP-HTMPL
Pygnat_2       IHLYG----FWPFPRDSE-G---KPVKYHYYDTLTYQYTSSSSP-HTMPL
Parkin_2       IHLYG----FWPFALGPQ-G---NPVKYHYYDSLTYEYTSQASP-HTMPL
Gorgor_2       IYLYG----FWPFPLDQN-Q---NPVKYHYYDSLKYGYTSQASP-HTMPL
Thegel_2       IYLYG----FWPFPLDQN-Q---NPVKYHYYDSLKYGYTSQASP-HTMPL
Tupchi_2       IYLYG----FWPFPLDQN-Q---NPVKYHYYDSLKYGYTSQASP-HTMPL
Galgal_2       IHLYG----FWPFPLDQN-Q---QPVKYHYYDSLKYGYTSQASP-HTMPL
Eleele_2       IHLYG----FWPFSHGPE-G---KPVKYHYYDALTYQYTSSSSP-HTMPL
Danrer_2       IHLYG----FWPFAHDPD-G---KPVKYHYYDTLTYHYTSSASP-HTMPL
Canlupfam_2    IYLYG----FWPFPLDQN-Q---NPVKYHYYDSLKYGYTSQASP-HTMPL
Aloalo_2       IHLYG----FWPFHRDHQ-G---KRVKYHYYDTLTYEFTSRASP-HTMPL
Astmex_2       IHLYG----FWPFPRDSE-G---KPVKYHYYDTLKYQYTSSSSP-HTMPL
Sinans_2       IHLYG----FWPFAHDPD-G---KPVKYHYYDTLTYHYTSSASP-HTMPL
Sclfor_2       IHLYG----FWPFLLGPH-G---NPVKYHYYDALTYEYTSQVGP-HTMPL
Ceraty_2       IYLYG----FWPFPLDQN-Q---NPVKYHYYDSLKYGYTSQASP-HTMPL
Anocar_2       IYLYG----FWPFPRDQD-Q---NPVKYHYYDTLKYGYMSQTSP-HTMPL
Anates_2       IHLYG----FWPFPLDPQ-G---KPVKYHYYDALKYEYTSSSSP-HTMPL
Ampbic_2       IHLYG----FWPFPLDPH-G---KPVKYHYYDTLKYEYTSSSSP-HTMPL
Sarpil_2       IHLYG----FWPFHRDHQ-G---KRVKYHYYDTLTYQFTSRASP-HTMPL
Xenlae_2       IYLYG----FWPFPRDLH-Q---NPVKYHYYDSLKYGYTSQAGP-HAMPL
Cluhar_2       IHLYG----FWPFHRDHQ-G---NVVKYHYYDTLTYEFTSRASP-HTMPL
Cypcar_2       IHLYG----FWPFAHDPD-G---KPVKYHYYDMLTYHYTSSASP-HTMPL
Denclu_2       IHLYG----FWPFHRDAQ-G---KPVKYHYYDALTYEYTSHSSP-HTMPL
Notpie_2       IHLYG----FWPFPLDPK-G---KTVKYHYYDTLKYEYTSSSSP-HTMPL
Erpcal2        IHLYG----FWPFLQDQK-G---NSVKYHYYDSLRYEFDSQSSP-HAMPL
Amical_2       IHLYG----FWPFSRGPA-G---NPVKYHYYDSLTYEYTAQSSP-HTMPL
Lepocu_2       IHLYG----FWPFSLGPN-G---LPVKYHYYDSLTYEYRAQSSP-HSMPV
Homsap_2       IYLYG----FWPFPLDQN-Q---NPVKYHYYDSLKYGYTSQASP-HTMPL
Bostau_2       IYLYG----FWPFPLDQN-Q---NPVKYHYYDSLKYGYTSQASP-HTMPL
Caraur_2       IHLYG----FWPFAHDPD-G---KPVKYHYYDTLTYHYTSSASP-HTMPL
Thythy_2B      IHLYG----FWPFPKDSQ-G---KPVKYHYYDTLTYEYTSHASP-HTMPL
Onctsh_2B      IHLYG----FWPFPQDSQ-G---KPVKYHYYDTLTYEYTSHASP-HTMPL
Salsal_2B      IHLYG----FWPFPQDSQ-G---KSVKYHYYDTLTYEYTSHASP-HTMPL
Oncmyk_2B      IHLYG----FWPFPQDSQ-G---KPVKYHYYDTLTYEYTSHASP-HTMPL
Onckis_2B      IHLYG----FWPFPQDSQ-G---KPVKYHYYDTLTYEYTSHASP-HTMPL
Salsal_2A      IHLYG----FWPFPRDSQ-G---KPVKYHYYDTLTYEYTSHASP-HAMPL
Thythy_2A      IHLYG----FWPFPRNSQ-G---KPVKYHYYDTLTYEYTSHASP-HTMPL
Salalp_2A      IHLYG----FWPFPRDSQ-G---KPVKYHYYDTLTYEYTSHASP-HTMPL
Corlav_2A      IHLYG----FWPFPRDSQ-G---IPVKYHYYDTLTYEYTSHASP-HTMPL
Cormar_2A      IHLYG----FWPFPRDSQ-G---IPVKYHYYDTLTYEYTSHASP-HTMPL
Onctsh_2A      IHLYG----FWPFPRDSQ-G---KPVKYHYYDTLTYEYTSHASP-HTMPL
Oncmyk_2A      IHLYG----FWPFPQDSQ-G---KPVKYHYYDTLTYEYTSHASP-HTMPL
Neobri_2       IHLYG----FWPFPLDPQ-G---KPVKYHYYDTLKYEYTSSSSP-HTMPL
Takrub_2       IHLYG----FWPFPSDPH-G---RPVKYHYYDTLKYEYTSSSSP-HTMPL
Tetnig_2       IHLYG----FWPFPLDPR-G---RPVKYHYYDTLKYEYTSSSSP-HTMPL
Orenil_2       IHLYG----FWPFPLDPQ-G---KPVKYHYYDTLKYEYTSSSSP-HTMPL
Orylat_2       IHLYG----FWPFPVDSH-G---KAVKYHYYDSLKYEYTSSSSP-HTMPL
Krymar_2       IHLYG----FWPFALDPR-G---RPVKYHYYDALKYEYTSSSSP-HAMPL
Notfur_2       IHLYG----FWPFPLDPK-G---KTVKYHYYDTLKYEYTSSSSP-HTMPL
Poelat_2       IHLYG----FWPFPLGPQ-G---RPVKYHYYDTLKYEYTSSSSP-HTMPL
Gadmor_2       IHLYG----FWPFPLDPR-G---RPVKYHYYDTLKYDYTSHSSP-HTMPL
Xipmac_2       IHLYG----FWPFPLGPQ-G---RPVKYHYYDTLKYEYTSSSSP-HTMPL
Poeret_2       IHLYG----FWPFPLGPQ-G---RPVKYHYYDTLKYEYTSSSSP-HTMPL
Astcal_2       IHLYG----FWPFSLDPQ-G---KPVKYHYYDTLKYEYTSSSSP-HTMPL
Hapbur_2       IHLYG----FWPFSLDPQ-G---KPVKYHYYDTLKYEYTSSSSP-HTMPL
Acapol_2B      IHLYG----FWPFPLDPH-G---KPVKYHYYDTLKYEYTSSSSP-HTMPL
Ampoce_2A      IHLYG----FWPFPLDPH-G---NPVKYHYYDTLKYEYTSSSSP-HTMPL
Acapol_2A      IHLYG----FWPFPLDPH-G---KPVKYHYYDTLKYEYTSSSSP-HTMPL
```

```
Mayzeb_2        IHLYG----FWPFSLDPQ-G---KPVKYHYYDTLKYEYTSSSSP-HTMPL
Punnye_2        IHLYG----FWPFSLDPQ-G---KPVKYHYYDTLKYEYTSSSSP-HTMPL
Latcal_2        IHLYG----FWPFPLDPQ-G---KPVKYHYYDTLKYEYTSSSSP-HTMPL
Singra_2        IHLYG----FWPFAHDPD-G---KPVKYHYYDTLTYHYTSSASP-HTMPL
Auslim_2        IHLYG----FWPFPLDPR-G---RPVKYHYYDTLKYEYTSSSSP-HTMPL
Monalb2         IHLYG----FWPFPLDLQ-G---KPVKYHYYDTLKYEYTSSSSP-HTMPL
Ampoce_2B       IHLYG----FWPFPLDPH-G---NPVKYHYYDTLKYEYTSSSSP-HTMPL
Chiham_2        IHLYG----FWPFSHDPQ-G---KPVKYHYYDTLKYEYTSSSSP-HTMPL
Gasacu_2        IHLYG----FWPFRHDPQ-G---RPVKYHYYDALKYEYTSSSSP-HTMPL
Gymacu_2        IHLYG----FWPFSHDPQ-G---KPVKYHYYDTLKYEYTSSSSP-HTMPL
Labber_2        IHLYG----FWPFPLDPQ-G---RPVKYHYYDTLKYEYTSSASP-HTMPL
Cypvar_2        IHLYG----FWPFPLDPQ-G---RPVKYHYYDTLKYEYTSSSSP-HTMPL
Funhet_2        IHLYG----FWPFPLGPH-G---RPVKYHYYDTLKYQYTSSSSP-HTMPL
Larcro_2        IHLYG----FWPFPLDPQ-G---KPVKYHYYDTLKYEYTSSSSP-HTMPL
Paroli_2        IHLYG----FWPFALDPQ-G---KPVKYHYYDTLKYEYTSRSSP-HSMPL
Perflu_2        IHLYG----FWPFPLDPQ-G---RSVKYHYYDTLKYEYTSSSSP-HTMPL
Serdum_2        IHLYG----FWPFALDPQ-G---KPVKYHYYDTLKYEYTSSSSP-HTMPL
Stepar_2        IHLYG----FWPFPLDPQ-G---KPVKYHYYDTLKYEYTSSSSP-HTMPL
Notcor_2        IHLYG----FWPFSHDPQ-G---KPVKYHYYDTLKYEYTSSSSP-HTMPL
Treber_2        IHLYG----FWPFSHDPQ-G---KPVKYHYYDTLKYEYTSSSSP-HTMPL
Serlaldor_2     IHLYG----FWPFALDPQ-G---KPVKYHYYDTLKYEYTSSSSP-HTMPL
Plaste_2        IHLYG----FWPFALDPQ-G---KPVKYHYYDTLKYEYTSRSSP-HTMPL
Clabat_4        IHLYG----FWPFPKDNS-G---NPVKYHYYDMLKYRYFSNAGP-HRMPL
Ictpun_4A       IHLYG----FWPFPKDSS-G---NPVKYHYYDMLKYRYFSNASP-HRMPL
Panhyp_4        IHLYG----FWPFPKDAS-G---NPVKYHYYDMLKYRYFSNASP-HRMPL
Umbpyg_4        IHLYG----FWPFPRDAK-G---NAVKYHYYDMLKYRYFSNAGP-HRMPL
Esoluc_4        IHLYG----FWPFPRDAN-G---NVVKYHYYDMLKYSYFSNAGP-HRMPL
Plealt_4        IHLYG----FWPFPRDAN-G---NLVKYHYYDMLKYRYFSNAGP-HRMPL
Onckis_4B       IHLYG----FWPFPRDAN-G---NMVKYHYYDMLKYRYFSNASP-HRMPL
Onckis_4A       IHLYG----FWPFPRDAN-G---NMVKYHYYDMLKYRYFSNASP-HRMPL
Onctsh_4        IHLYG----FWPFPRDAN-G---NMVKYHYYDMLKYRYFSNASP-HRMPL
Oncmyk_4        IHLYG----FWPFPRDAN-G---NMVKYHYYDMLKYRYFSNAGP-HRMPL
Corlav_4        IHLYG----FWPFPRDSN-G---NVVKYHYYDLLKYRYFSNAGP-HRMPL
Astmex_4A       IHLYG----FWPFPKDAN-G---IPVKYHYYDLLKYRYFSSAGP-HRMPL
Pygnat_4        IHLYG----FWPFPKDAA-G---NPVKYHYYDTLKYRYFSRAGP-HRMPL
Aloalo_4        IHLYG----FWPFPKDTH-G---NPVKYHYYDTLKYRYFSNAGP-HRMPL
Cluhar_4        IHLYG----FWPFPKDSQ-G---NPVKYHYYDTIKYRYFSNAGP-HRMPL
Denclu_4        IHLYG----FWPFPRDTQ-G---NPVKYHYYDMLKYRYLSNAAP-HRMPL
Konpun4         IHLYG----FWPFPKDSQ-G---NTVKYHYYDTLKYRYFSNAGP-HRMPL
Sarpil_4        IHLYG----FWPFPKDTH-G---NPVKYHYYDTLKYRYFSNAGP-HRMPL
Caraur_4        IHLYG----FWPFPRDGS-G---NPVKYHYYDGLRYRYFSNAGP-HRMPL
Cteide_4        IYLYG----FWPFPKDAN-G---NPVKYHYYDTLKYRYFSNVGP-HRMPL
Petmar_4        IHLYG----FWPFQYNIA-G---KPVRYHYYEGLRYRYRSRAGP-HTMPL
Cypcar_4        IHLYG----FWPFPKDGN-G---NPVKYHYYDGLKYRYFSNAGP-HRMPL
Danrer_4        IYLYG----FWPFPKDAS-G---NPVQYHYFDGLKYRYFSNAGP-HRMPL
Misang_4        IHLYG----FWPFPKDGN-G---NPVNYHYYDTLKYRYFSNAGP-HRMPL
Angjap_4        IHLYG---------------------------------------------
Cypcar_4A       IHLYG----FWPFPKDGN-G---NPVKYHYYDGLKYRYFSNAGP-HRMPL
Angang_4        IHLYG----FWPFPKDLG-G---NPVKYHYYDALKYRYFSKASP-HRMPL
Cypcar_4B       IHLYG----FWPFPKDGN-G---NPVKYHYYDGLRYRYFSNASP-HRMPL
Sinans_4A       IHLYG----FWPFPKDGN-R---NTVKYHYYDGLKYRYFSSAGP-HRMPL
Sinans_4B       IYLYG----FWPFPKDGN-G---NPVKYHYYDGLRYRYFSNAGP-HRMPL
Singra_4A       IHLYG----FWPFPKDGN-R---NPVKYHYYDGLKYRYFSSAGP-HRMPL
Aptalb_4        IHLYG----FWPFPKDAE-G---NPVKYHYYDSLKYRYFSSAGP-HRMPL
Eigvir_4        IHLYG----FWPFPKDAE-G---NPVKYHYYDTLKYRYFSSAGP-HRMPL
Eleele_4        IHLYG----FWPFPKDAK-G---NLVKYHYYDTIKYRYFSNAGP-HRMPL
Parhas_4        IHLYG----FWPFPKDAE-G---NPVKYHYYDSLKYRYFSSAGP-HRMPL
Sclfor_4        IHLYG----FWPFPKDSK-G---NAVKYHYYDALKYRYFSNASP-HKMPL
Gnapet4         IHLYG----FWPFPKDSS-G---NPVKYHYYDKLKYRYFSNAGP-HRMPL
Ostbic_4        IHLYG----FWPFPKDSK-G---NMVKYHYYDTLKYRYSSKAGP-HRMPL
Parkin_4        IHLYG----FWPFPKDSS-G---NPVKYHYYDKLKYRYFSNAGP-HRMPL
Panbuc_4        IHLYG----FWPFPKDLQ-G---NPVKYHYYDRLKYRYFSKAGP-HRMPL
Erpcal4         IHLYG----FWPFPKDSI-G---KPVKYHYYDELKYRYFSNASP-HRMPL
Salsal_4        IHLYG----FWPFPRDAN-G---NMVKYHYYDMLKYRYFSNAGP-HRMPL
Saltru_4        IHLYG----FWPFPRDAN-G---NMVKYHYYDMLKYRYFSNAGP-HRMPL
Salalp_4        IHLYG----FWPFPRDAN-G---KIVKYHYYDMLKYRYFSNAGP-HRMPL
Salfon_4        IHLYG----FWPFPRDAN-G---KIVKYHYYDMLKYRYFSNAGP-HRMPL
Thythy_4        IHLYG----FWPFPRDAN-G---NVVKYHYYDMLKYRYFSNAGP-HRMPL
Calmil_4        IHLYG----FWPFPKDAN-G---TPVKYHYYDNLKYRYFSNAGP-HRMPL
Hetzeb_4        IHLYG----YWPFPKDAK-G---TPVKYHYYDNLKYRYFSNVGP-HRMPL
Rhityp_4        IHLYG----YWPFPKDSK-G---VPVKYHYYDNLKYRYFSNAGP-HRMPL
Scytor_4        IHLYG----YWPFPKDSK-G---TPVKYHYYDNLKYRYFSNAGP-HRMPL
Squaca_4        IHLYG----YWPFPKDAK-G---APVKYHYYDNLKYRYFSNAGP-HRMPL
Amical_4        IHLYG----FWPFPKDTK-G---RLVKYHYYDELKYRYFSNASP-HRMPL
Lepocu_4        IHLYG----FWPFPKDSQ-G---RPVKYHYYDELKYRYFSNASP-HRMPL
Agema_4         IHLYG----FWPFPKDAS-G---NPVKYHYYDMLKYRYFSNASP-HRMPL
Anocar_4        IHLYG----FWPFPRDIN-G---KTVKYHYYDDLKYRYFSNASP-HRMPL
```

```
Canlupfam_4    IHLYG----FWPFPKDLN-G---KAVKYHYYDDLKYRYFSNASP-HRMPL
Crigri_4       IHLYG----FWPFPKDLN-G---KAVKYHYYDDLKYRYFSNASP-HRMPL
Galgal_4       IHLYG----FWPFPKDLH-G---KPVKYHYYDDLKYRYFSNASP-HRMPL
Mesaur_4       IHLYG----FWPFPKDLN-G---KAVKYHYYDDLKYRYFSNASP-HRMPL
Homsap_4       IHLYG----FWPFPKDLN-G---KAVKYHYYDDLKYRYFSNASP-HRMPL
Notscuscu_4    IHLYG----FWPFPKDIN-G---KPVKYHYYDDLKYRYFSNASP-HRMPL
Psetextex_4    IHLYG----FWPFPKDIN-G---KPVKYHYYDDLKYRYFSNASP-HRMPL
Musmus_4       IHLYG----FWPFPKDLN-G---KAVKYHYYDDLKYRYFSNASP-HRMPL
Pogvit_4       IHLYG----FWPFPKDMN-G---KPVKYHYYDDLKYRYFSNASP-HRMPL
Promuc_4       IHLYG----FWPFPKDIN-G---KPVKYHYYDDLKYRYFSNASP-HRMPL
Pantro_4       IHLYG----FWPFPKDLN-G---KAVKYHYYDDLKYRYFSNASP-HRMPL
Pytbiv_4       IHLYG----FWPFPKDIN-G---KPVKYHYYDDLKYRYFSNASP-HRMPL
Ratnor_2       IYLYG----FWPFPLDQN-Q---NPVKYHYYDSLKYGYTSQASP-HTMPL
Ratnor_4       IHLYG----FWPFPKDLN-G---KAVKYHYYDDLKYRYFSNASP-HRMPL
Siltro_2       IYLYG----FWPFPRDLH-Q---NPVKYHYYDSLKYGYTSQAGP-HAMPL
Siltro_4       IHLYG----FWPFPKDVY-G---NQVKYHYYDELKYKYFSNAGP-HRMPL
Susscr_4       IHLYG----FWPFPKDLN-G---KAVKYHYYDDLKYRYFSNASP-HRMPL
Taegut_4       IHLYG----FWPFPKDLH-G---KPVKYHYYDDLKYRYFSNASP-HRMPL
Termextri_4M   IHLYG----FWPFPKDFY-G---KPVKYHYYDDLKYRYFSNASP-HRMPL
Thasirsir_4    IHLYG----FWPFPKDIN-G---KPVKYHYYDDLKYRYFSNASP-HRMPL
Xenlae_4B      IYLYG----FWPFTKDVY-G---NQVKYHYYDELKYKYFSNAGP-HRMPL
Xenlae_4A      IHLYG----FWPFPKDVH-G---NQVKYHYYDELKYKYFSNAGP-HRMPL
               251
Latcal_3       EFKLLYKMHTEGLLKLTLSHCA---
Perflu_3       EFKLLYKMHTEGLLKLSLSHCA---
Serdum_3       EFKLLYKMHMEGLLKLSLSHCA---
Stepar_3       EFKLLYKMHTEGLLKLSLSHCA---
Tetcal_3       EFKLLFKLHTEGLTKLTLSHCA---
Monalb_3       EFKLLYKMHMEGLLKLTLSRCA---
Molmol_3       EFKLLYRMHTEGLLKLTLSHCA---
Takrub_3       EFKLLYKMHREGLLKLSLSHCA---
Canlupfam_3    EFQLLYRMHGEGLTKLTLSHCA---
Bostau_3       EFQLLYRMHGEGLTKLTLSHCA---
Homsap_3       EFQLLYRMHGEGLTKLTLSHCA---
Siltro_3       EFKLLYKMHREGLTKLTLSQCA---
Anocar_3       EFQQLFRMHSEGLAKLTLSHCA---
Musmus_3       EFQLLYRMHGEGLTKLTLSHCA---
Pantro_3       EFQLLYRMHGEGLTKLTLSHCA---
Ratnor_3       EFQLLYRMHGEGLTKLTLSHCA---
Galgal_3       EFQLLYRMHGEGLAKLTLSRCA---
Lepocu_3       EFKLLYKMHTEGLTKLSLSHCA---
Tetnig_3       EFKLLYKMHREGVLKLSLSHCG---
Squaca_3       EFKLLFKLHTDGLTKLTLSHCA---
Perfla_3       EFKLLYKMHTEGLLKLSLSHCA---
Salsal_3       EFKLLYRMHTEGLIKLTLSHCA---
Serlaldor_3    EFRLLYKMHMEGLLKLSLSHCA---
Treber_3       EFKLLYKLHREGVIKLSVTHCS---
Cynsem_3       EFKLLYKMHTEGLLKLSLSHCA---
Paroli_3       EFKLLYKLHREGVTKLSLTHCT---
Paroli_3b      EFKLLYKMHTEGXLKLSLSHCA---
Plaste_3       EFKLLYQLHREGVTKLSLTHCA---
Plaste_3b      EFKLLYKMHTEGLLKLRLSHCG---
Erpcal3        EFKLLYKMHEEGLTKLTLSHCA---
Calmil_3b      EFKILFKMHTAGLTKLSLSPCV---
Hetzeb_3       EFKLLFKLHTDGLTKLTLSHCA---
Latcha_3       EFKLLYKMHGEGLTKLTLSHCA---
Anates_3       EFKLLYKMHMEGLLKLTLSHCA---
Ampcit3        EFKLLYKMHTEGLLKLSVSHCA---
Auslim_3       EFKLLYKMHMDGLLKLTLSHCA---
Cypvar_3       EFKLLYKMHTEGLLKLSLSHCA---
Krymar_3       EFKLLYRMHTDGLLKLTLSHCA---
Funhet_3       EFKLLYQMHTEGLLKLSLSHCA---
Gamaff3        EFKLLYKMHTEGLLKLSLSHCA---
Orylat_3       EFKLLHKMHTEGLLKLTLSHCA---
Orenil_3       EFKLLYKMHTEGLLKLSLSHCA---
Mayzeb_3       EFKLLYKMHTEGLLKLSLSHCA---
Punnye_3       EFKLLYKMHTEGLLKLSLSHCA---
Punnye_3a      EFKLLYKMHTEGLLKLSLSHCA---
Neobri_3       EFKLLYKMHTEGLLKLSLSHCA---
Misang_3       EFKLLFKMHTEGILKLSLSHCT---
Sinrhi3        EFKLLFKMHADGVLKLSLSHCT---
Singra_3       EFKLLFKMHADGVLKLSLSHCA---
Hapbur_3       EFKLLYKMHTEGLLKLSLSHCA---
Astcal_3       EFKLLYKMHTEGLLKLSLSHCA---
Notfur_3       EFKLLYKMHTDGVLKLSLSHCA---
Cypcar_3       -------------------------
Poefor3        EFKLLYKMHTEGLLKLSLSHCA---
```

Poelat_3	EFKLLYKMHTEGLLKLSLSHCA---
Poeret_3	EFKLLHRMHSEGLLKLSLSHCA---
Angang_3	EFKLLYKMHTDGVIKLSLSHCA---
Xipmac_3	EFKLLYKMHTEGLLKLSLSHCA---
Masarm_3	EFKLLYKMHMEGLLKLTLSHCA---
Permag_3	EFKLLYRMHTEGLLKLSLSHCA---
Gnapet3	EFKQLYKMHAEGLTKLSLSHCI---
Ostbic_3	EFKLLYKMHEEGLTKLSLARCA---
Gadmor_3	EFKLLYKMHTKGLLKLNLSHCA---
Sclfor_3	EFKLLYKMHEQGLAKLSLARCA---
Acapol3	EFKLLYKMHTEGLLKLSLSHCA---
Ampoce_3	EFKLLYKMHMEGLLKLSLSHCA---
Sclfor_3b	EFKLLHSLHMEGVVQLNLSPCA---
Ostbic_3b	EFKLLHSLHMEX------------
Parkin_3	EFKQLYKMHAEGLTKLSLSHCI---
Ampper3	EFKLLYKMHMEGLLKLSLSHCA---
Oncmyk_3	EFKLLYKMHTEGLIKLTLSHCA---
Gasacu_3	EFKLLYKMHTQGLLKLSLSHCA---
Labber_3	EFKLLYKMHTEGLLKLSLSHCA---
Panbuc_3	EFKFLYKMHTEGLIRLTLTPCI---
Parhas3	EFKLLFKMHTEGVLKLSLSHCT---
Cluhar_3	EFKLLFKMHTEGLLKLTLSHCA---
Aloalo_3	EFKLLFKMHTEGLLKLTLSHCA---
Panhyp_3	EFKLLFKMHTEGILKLSLSHCA---
Eleele_3	EFKLLFRMHTEGVLKLSLSHCT---
Denclu_3	EFKLLFKMHTEGLLKLSLSHCA---
Sarpil_3	EFKLLFKMHTEGLLKLTLTHCA---
Eigvir_3	EFKLLFKMHTEGVLKLSLSHCT---
Aptalb_3	EFKLLFKMHTEGVLKLSLSHCT---
Sinans_3	EFKLLFKMHADGVLKLSLSHCA---
Pygnat_3	EFKLLFKMHTEGVLKLSLSHCT---
Astmex_3	EFKLLFKMHTEGVLKLSLSHCA---
Caraur_3	EFKLLFKMHADGVLKLSLSHCA---
Danrer_3	EFKLLFKMHADGVLKLSLSHCA---
Ictpun_3	EFKLLFKMHTDGILKLSLSHCA---
Plealt_3	EFKLLYKMHTEGLLKLTLSHCA---
Plealt_9	EFKLLYKMHGEGVTRMSLSHCT---
Gasacu_9	EFKLLYKLHRDGVIKLSLTHCS---
Parkin_9	EFKLLHTLHTEGMVRLSVAPCA---
Acapol_9	EFKLLYKLHREGVIKLGLTHCS---
Larcro_9	EFKLLYKLHREGVIKLSLTHCT---
Notcor_9	EFKLLYKLHREGVIKLSVTHCS---
Latcal_9	EFKLLYKLHREGVTKLSLTHCT---
Serlaldor_9	EFKLLYKLHREGVTKLSLTHCT---
Monalb_9	EFKLLYKLYKEGVTKLSLTHCP---
Serdum_9	EFKLLYKLHREGVTKLSLTHCT---
Perflu_9	EFKLLYKLHREGVIKLSLTHCS---
Perfla_9	EFKLLYKLHREGVIKLSLTHCS---
Stepar_9	EFKLLYKLHRDGVIKLSLTHCS---
Molmol_9	EFKLLYKLHREGVIKLSLTHCA---
Hipcom_9	EFKLLYRLHRDGVTKLSLTHCT---
Takrub_9 B	EFKLLYKLHREGVIRLSLTHCT---
Takrub_9	EFKLLYKLHREGVIRLSLTHCT---
Cynsem_9	EFKLLYKLHREGVTKLSLTHCT---
Scomax_9	EFKLLYKLHREGVTKLSLTHCA---
Corlav_9	EFKLLYKMHGEGVTKLSLSHCS---
Oncmyk_9	EFKLLYKMHGEGVTKLSLSHCS---
Salsal_9	EFKLLYRMHTEGLIKLTLSHCA---
Onctsh_9	EFKLLYKMHGEGVTKLSLSHCS---
Salalp_9	EFKLLYKMHGEGVTKLSLSHCS---
Ampoce_9	EFKLLYKLHREGVIKLGLTHCS---
Ampper9	EFKLLYKLHREGVIKLGLTHCS---
Labber_9	EFKLLYKLHREGVIRLSLTHCS---
Umbpyg_3B	EFKLLYKMHTEGLIKLTLSHCA---
Esoluc_3b	EFKLLYKMHTEGLIKLTLSHCA---
Gadmor_9	EFRLLYKMHGEGVTKLSLSHCA---
Umbpyg_9	EFKLLYKMHAEGVTKLSLSHCS---
Esoluc_9	EFKLLYKMHGEGVTKLSLSHCS---
Permag_9	EFKLLYKLHREGAIKLSLTHCS---
Bolpec_9	EFKLLYKLHREGAIKLSLTHCS---
Poefor9	EFKLLYKLHREGVIKLSLTHCT---
Masarm_9	EFKLLYKLHREGVIKLSLTHCI---
Angjap_9	EFKLLYKMHTDGVIKLSLSHCA---
Poelat_9	EFKLLYKLHREGVIKLSLTHCT---
Poeret_9	EFKLLYKLHREGVIKLSLTHCT---
Xipmac_9	EFKLLYKLHREGVIKLSLTHCA---
Hapbur_9	EFKLLYKLHREGVIKLSLTHCS---

```
Orylat_9        EFKLLYKLHREGVTKLGLSHCT---
Gamaff_9        EFKLLYKLHREGVIKLSLTHCA---
Neobri_9        EFKLLYKLHREGVIKLSLTHCS---
Funhet_9        EFKLLYKLHREGVTKLSLTHCT---
Punnye_9        EFKLLYKLHREGVIKLSLTHCS---
Cypvar_9        EFKLLYKLHREGVTKLSLTHCT---
Krymar_9        EFKLLYKLHREGVIKLSLTHCI---
Notfur_9        EFKLLYKLHREGVTKLSLTHCS---
Notpie_9        EFKLLYKLHREGVTKLSLTRCS---
Orenil_9        EFKLLYKLHREGVIKLSLTHCS---
Auslim_9        EFKLLYKLHREGVTKLSLTHCT---
Anates_9        EFKLLYKLHREGVTKLSLTHCT---
Astcal_9        EFKLLYKLHREGVIKLSLTHCS---
Mayzeb_9        EFKLLYKLHREGVIKLSLTHCS---
Orymel_9        EFKLLYKLHREGVTKLGLSHCT---
Ampcit9         EFKLLYKLHREGVIKLSLTHCT---
Macfas_2        EFKALKSLHEQGALKLTVGQCD---
Macmul_2        EFKALKSLHEQGALKLTVGQCD---
Macnem_2        EFKALKSLHEQGALKLTVGQCD---
Musmus_2        EFKALKSLHEQGALKLTVGQCD---
Ponabe_2        EFKALKSLHEQGALKLTVGQCD---
Papanu_2        EFKALKSLHEQGALKLTVGQCD---
Nomleu_2        EFKALKSLHEQGALKLTVGQCD---
Pantro_2        EFKALKSLHEQGALKLTVGQCD---
Latcha_2        EFRTLYGLHQQGALKLTVGECA---
Gnapet2         EFKTLRALHRQGALCLHTEACS---
Panbuc_2b       EFQALKVLHERGALQLHTGLCH---
Ostbic_2        EFETLKALHRQGALRLHTGTCN---
Sinrhi2         EFRTLSALHRQGALRLHTGPCQ---
Hetzeb_2        EFKTLRNMHLQGALKLNVGECN---
Hipcom_2        EFRTLRALHHQGALRLHTGNCH---
Prigla_2        EFKTLRNMHLQGALKLNVGECK---
Scytor_2        EFKTLRNMHLQGALKLNVGECK---
Tetcal_2        EFKTLKNMHLRGALRLNVGECN---
Rhityp_2        EFKTLRNMHLQGALKLNIGACR---
Parkin_2b       EFRTLSRLHALGVLQLHTGSCR---
Angang_2        EFSTLRDLHSQGALRLHIGRCG---
Pygnat_2        EFRTLSMLHRKGALRLHTGTCR---
Parkin_2        EFKTLRALHRQGALCLHTEACS---
Gorgor_2        EFKALKSLHEQGALKLTVGQCD---
Thegel_2        EFKALKSLHEQGALKLTVGQCD---
Tupchi_2        EFKALKSLHEQGALKLTVGQCD---
Galgal_2        EFKALKTLHQQGALKLTVGECE---
Eleele_2        EFRTLSTLHSQGALQLHTGTCE---
Danrer_2        EFRTLSALHRQGALRLHTGPCK---
Canlupfam_2     EFKALKSLHEQGALKLTVGQCD---
Aloalo_2        EFRTLSSLHRQGALRLHTSACS---
Astmex_2        EFRTLSTLHRQGALRLHTETCR---
Sinans_2        EFRTLSALHRQGALQLHTGPCK---
Sclfor_2        EFETLRALHRQGALRLHTGACN---
Ceraty_2        EFKALKSLHEQGALKLTVGQCD---
Anocar_2        EFKALKALHQQGALKLTVGACD---
Anates_2        EFRTLSALHRQGALRLHTGQCD---
Ampbic_2        EFRTLSTLHRQGALRLHTGTCD---
Sarpil_2        EFRTLSSLHRQGALRLHTSACS---
Xenlae_2        EFKALKNLHLQGALKLNVGECE---
Cluhar_2        EFRTLSTLHKQGALRLHTSSCS---
Cypcar_2        EFRTLSALHRQGALRLHTGPCK---
Denclu_2        EFRTLRALHRQGALRLHTGTCS---
Notpie_2        EFRTLSSLHRQGALRLHTGTCD---
Erpcal2         EFQALQRLHQQGALKLNIGDCV---
Amical_2        EFKTLKNLHSQGALQLHTGPCN---
Lepocu_2        EFRALKRLHSQGALKLHTGECN---
Homsap_2        EFKALKSLHEQGALKLTVGQCD---
Bostau_2        EFKALKSLHEQGALKLTVGQCD---
Caraur_2        EFRTLSALHRQGALRLHTGPCK---
Thythy_2B       EFRTLSSLHRQGALRLHTGSCD---
Onctsh_2B       EFRTLSSLHRQGALRLHTGSCD---
Salsal_2B       EFRTLSSLHRQGALRLHTGSCD---
Oncmyk_2B       EFRTLSSLHRQGALRLHTGSCD---
Onckis_2B       EFRTLSSLHRQGALRLHTGSCD---
Salsal_2A       EFRTLSSLHRQGALRLNTGSCD---
Thythy_2A       EFRTLSSLHRQGALQLNTGSCD---
Salalp_2A       EFRTLSSLHRQGALRLNTGSCD---
Corlav_2A       EFRTLSSLHRQGALRLNTGSCD---
Cormar_2A       EFRTLSSLHRQGALRLNTGSCD---
Onctsh_2A       EFRTLSSLHRQGALRLNTGSCD---
```

```
Oncmyk_2A      EFRTLSSLHRQGALRLNTGSCD---
Neobri_2       EFRTLSTLHRQGALRLHTGVCG---
Takrub_2       EFRTLSSLHRQGALQLHTGPCD---
Tetnig_2       EFRTLSTLHRQGALRLHSGPCA---
Orenil_2       EFRTLSTLHRQGALRLHTGVCG---
Orylat_2       EFRTLSTLHRQGALRLHTGSCE---
Krymar_2       EFRTLSSLHRQGALRLHTGICH---
Notfur_2       EFRTLSSLHRQGALRLHTGTCD---
Poelat_2       EFRTLSTLHRQGALRLHTGSCD---
Gadmor_2       EFRTLNTLHRKGALRLHTGTCP---
Xipmac_2       EFRTLSTLHRQGALRLHTGSCD---
Poeret_2       EFRTLSTLHRQGALRLHTGSCD---
Astcal_2       EFRTLSTLHRQGALRLHTGVCG---
Hapbur_2       EFRTLSTLHRQGALRLHTGVCG---
Acapol_2B      EFRTLSALHRQGALRLHTGTCD---
Ampoce_2A      EFRTLSTLHRQGALRLHTGTCD---
Acapol_2A      EFRTLSALHRQGALRLHTGTCD---
Mayzeb_2       EFRTLSTLHRQGALRLHTGVCG---
Punnye_2       EFRTLSTLHRQGALRLHTGVCG---
Latcal_2       EFRTLSTLHRQGALRLHTGTCD---
Singra_2       EFRTLSALHRQGALQLHTGPCK---
Auslim_2       EFRTLSSLHRQGALRLHTGTCD---
Monalb2        EFRTLSTLHRQGALRLHTGPCD---
Ampoce_2B      EFRTLSTLHRQGALRLHTGTCD---
Chiham_2       EFRTLSALHRQGALRLHTGTCD---
Gasacu_2       EFRTLSALHRQGALRLHTGTCG---
Gymacu_2       EFRTLSALHRQGALRLHTGTCD---
Labber_2       EFRTLSALHRQGALRLHTGSCD---
Cypvar_2       EFRTLSTLHRQGALRLHTGSCD---
Funhet_2       EFRTLSTLHRQGALRLHTGHCD---
Larcro_2       EFRTLSALHRQGALQLHTGSCD---
Paroli_2       EFRTLNTLHRQGALRLHTGTCE---
Perflu_2       EFRTLSALHRQGALRLHTGTCD---
Serdum_2       EFRTLSTLHRQGALRLHTGTCD---
Stepar_2       EFRTLSTLHRQGALRLHTGTCD---
Notcor_2       EFRTLSALHRQGALRLHTGTCD---
Treber_2       EFRTLSALHRQGALRLHTGTCD---
Serlaldor_2    EFRTLSTLHRQGALRLHTGTCD---
Plaste_2       EFRTLNALHRQGALRLHTGTCD---
Clabat_4       EFKTLKMLHSKGALKLTTSKCR---
Ictpun_4A      EFKTLKMLHSKGALKLMTSKCT---
Panhyp_4       EFKTLKMLHSKGALKLTTSKCR---
Umbpyg_4       EFKTLKILHSKGALKLTTSKCT---
Esoluc_4       EFKTLKMLHSKGALKLTTSKCA---
Plealt_4       EFKTLKMLHSKGALKLTTSKCI---
Onckis_4B      EFKTLKMLHSKGALKLTTSKCE---
Onckis_4A      EFKTLKMLHSKGALKLTTSKCE---
Onctsh_4       EFKTLKMLHSKGALKLTTSKCE---
Oncmyk_4       EFKTLKMLHSKGALKLTTSKCE---
Corlav_4       EFKTLKMLHSKGALKLTTSKCE---
Astmex_4A      EFKTLKTLHSKGALKLTTSKCR---
Pygnat_4       EFKTLKMLHSKGALKLMTSTCR---
Aloalo_4       EFKTLKTLHSKGALKLTTSKCV---
Cluhar_4       EFKTLKVLHSKGALKLTTSKCM---
Denclu_4       EFKTLKALHYKGALKLTTSKCT---
Konpun4        EFKTLKTLHSKGALKLTTSKCT---
Sarpil_4       EFKTLKTLHSKGALKLTTSKCV---
Caraur_4       EFKTLKRLHSKGALKLTTSKCT---
Cteide_4       EFNTLKSLHNKGAMKLTTSKCT---
Petmar_4       EYRTLHRLHSLGALKLTTGKCT---
Cypcar_4       EFKTLKTLHSKGALKLTTSECT---
Danrer_4       EFQTLQRLHSKGALKLTTSKCT---
Misang_4       EFRTLRTLHNKGALKLTTTKCE---
Angjap_4       -------------------------
Cypcar_4A      EFKTLKTLHSKGALKLTTSECT---
Angang_4       EFKTLKTLHSRGALKLTTSKCA---
Cypcar_4B      EFKTLKTLHSKGALKLTTSKCT---
Sinans_4A      EFKTLKTLHSKGALKLTTSECT---
Sinans_4B      EFKTLKRLHSKGALKLTTSKCT---
Singra_4A      EFKTLKTLHSKGALKLTTSECT---
Aptalb_4       EFKTLKMLHGKGALKLTTSKC----
Eigvir_4       EFKTLKMLHSKGALKLTTSKCP---
Eleele_4       EFKTLKMLHSKGALKLTTSKCP---
Parhas_4       EFKTLKMLHGKGALKLTTSKCR---
Sclfor_4       EFNTLKMLHHKGALKLTTSKCT---
Gnapet4        EFKTLKFLHDRGALKLTTSKCT---
Ostbic_4       EFKTLKMLHNKGALKLTTLKCT---
```

```
Parkin_4        EFKTLKFLHERGALKLTTSKCT---
Panbuc_4        EFQTLKALHSKGALKLTTSTCT---
Erpcal4         EFKTLKMLHSKGALKLTTSKCL---
Salsal_4        EFKTLKMLHSKGALKLTTSKCE---
Saltru_4        EFKTLKMLHSKGALKLTTSKCE---
Salalp_4        EFKTLKMLHSKGALKLTTSKCE---
Salfon_4        EFKTLKMLHSKGALKLTTSKCE---
Thythy_4        EFKTLKMLHSKGALKLTTSKCE---
Calmil_4        EFKTLKSLHDRGALKLTTGKCG---
Hetzeb_4        EFKTLKSLHDRGALKLTTGKCV---
Rhityp_4        EFKTLKSLHDKGALKLTTGKCV---
Scytor_4        EFKTLKSLHDKGALKLTTGKCV---
Squaca_4        EFKTLKSLHDKGALKLTTGKCV---
Amical_4        EFKTLKTLHNKGALKLTTSTCA---
Lepocu_4        EFKTLKMLHNKGALKLTTTKCT---
Agema_4         EFKTLKMLHSKGALKLTTSKCR---
Anocar_4        EFKTLNMLHNRGALKLTTGKCA---
Canlupfam_4     EFKTLNVLHNRGALKLTTGKCV---
Crigri_4        EFKTLNVLHNRGALKLTTGKCM---
Galgal_4        EFKTLYVLHNRGALKLTTGKCV---
Mesaur_4        EFKTLNVLHNRGALKLTTGKCM---
Homsap_4        EFKTLNVLHNRGALKLTTGKCV---
Notscuscu_4     EFKTLNMLHNKGALKLTTGKCI---
Psetextex_4     EFKTLNMLHNKGALKLTTGKCI---
Musmus_4        EFKTLNVLHNRGALKLTTGKCM---
Pogvit_4        EFKTLNVLHNRGALKLTTGKCV---
Promuc_4        EFKTLNMLHNRGALKLTTGKCI---
Pantro_4        EFKTLNVLHNRGALKLTTGKCV---
Pytbiv_4        EFKTLNMLHNRGALKLTTGKCI---
Ratnor_2        EFKALKSLHEQGALKLTVGQCD---
Ratnor_4        EFKTLNVLHNRGALKLTTGKCM---
Siltro_2        EFKALKNLHLQGALKLNVGECR---
Siltro_4        EFKTLNLLHNKGALKLTTGKCI---
Susscr_4        EFKTLNVLHNRGALKLTTGKCV---
Taegut_4        EFKTLYVLHNRGALKLTTGKCI---
Termextri_4M    EFKTLHMLHNQGALKLTTGKCV---
Thasirsir_4     EFKTLNMLHNRGALKLTTGKCI---
Xenlae_4B       EFKTLNLLHNKGALKLTTGKCI---
Xenlae_4A       EFKTLNLLHNKGALKLTTGTCI---
```

B) Multiple sequence alignments (ClustalX) of 89 chordate ST8Sia sequences used in figure 2: Minimum Evolution phylogenetic tree of 89 chordates ST8Sia.

```
CLUSTAL X (1.81) multiple sequence alignment



Angang_3       LQEKSAKWKFNRTAFIQQRK----EISNYIDVAGNFTLTKSSVRVGQLMH
Lepocu_3       LQEKTSKWKLNKTAFIQQRK----EILRYIDVANNFSLTKNSVRVGQLMH
Latcha_3       LQEKPSKWIFNRTAFLKQRE----EILQNVDVVKNFSLTKSSVRVGQLMH
Squaca_3       AHAKPAKWTFNTTSFLQQRK----DISRYVDVFKNFTLVKKNVRVGQLLH
Astmex_3       LQENSSKWKFNRTAYLEQSK----EIAQYIDIPHNFTLTKNSVRVGQLMH
Orylat_3       -LKNSSKWTYNSTAFIQLRT----EISHHIDMPHNFTLTRDAVKVGQLMH
Hetzeb_3       VRGKPVKWTFNTTSFLKQRK----DISRYVDIYKNFTLVKKNVRVGQLLH
Anates_3       -MQNNSKWTYNSTAFTQLRK----EISQHIDIPHNFTLTRDSVRIGQLMH
Acisin_3       LQDKPSKWKFNRTAFLQQRK----EVLHYIDVAKNFTLTKDGIRVGQLMH
Masarm_3       DLQNSSKWRYNSTAFIQQRK----EISQYIDIPHNFTLTRRSVRIGQLMH
Calmil_3       FPGKPSKWAFNKTAYLKQRE----DIRHYIDVIQNFTLIKKNVRVGQLMH
Orymel_9       LQGKPYKWKFNRTAFYQQRK----DIFSYIDIPHNFSLTKNSVRVGQLMH
Anates_9       LQGKPSKWKFNKTAFYQQRK----DIFSYIDIPNNFSLTKNSVRVGQLMH
Masarm_9       LQGKPPKWKFNKTAFYQQRK----DIFSYIDIPSNFSLTKGSVRVGQLMH
Orylat_9       LQGKPYKWKFNRTAFYQQRK----DIFSYIDIPNNFSLTKNSVRVGQLMH
Eptbur_3/9     NLQELKHWSLNPAAVHHQRT----ELASFVDVSAELSLTRSTLLPGQLLH
Letcam_3/9     --------------------------------------------------
Anates_2       ARHSPNSWTFNKTLSNLIRK----NILRFFDPERDISILKGTLKPGDIIH
Latcha_2       -KPQLVEWKHNRTLSLKIRK----QILKFLDAERDISVLKGTLKPGDVIH
Amical_2       RTISPSEWRFNSSLSRSIRK----NILKFLDAEKDISVLKGNLKPGDTIH
Acisin_2       IVPAASEWRFNKDLSISIRK----NILKFLDAERDISVLKGNVQPGDIIH
Angang_2       LATPSSEWSFNRTLSNSIRK----NILKFLDAERDISMLRGSLRPGEVIH
Astmex_2       FTSPASGWIFNRTLSSIIRK----KVLRFLDAERDISILKSTFKPGDIIH
Orylat_2       NNHSLKTWTFNKTLSNLIRK----NILQFLDPERDISILKGTLKPGDIIH
Hetzeb_2       RLIDIPEWKFNATLTARIRK----DILKFLDAEKDISVLKGKLKAGDIIH
Lepocu_2       IESPSSDWRFNRSLSNIIRK----SILKFLDAEKDISVLKGNLKPGDTIH
Latcha_4       -QHSAEGWKINSSLVLEIRK----DILRFLDAERDVSAVKSNFKPGDVIH
Astmex_4-A     --LQSEDWKVNATLVAMIRK----DILHFLDAEKDVSVIKSNFKPGDTIH
Angang_4       LQHTAQGWHLNSSLVLMIRK----DVLRFLDAERDVSVVKSSLKPGDIIH
Lepocu_4       FQHSVEGWKVNSSLVIEIRK----DILRFLDAERDVSVVKSSFKPGDVIH
Amical_4       FQHSVEGWKVNSSLVIEIRK----DILRFLDAERDVSVVKSSFKPGDVIH
Calmil_4       FKHSVEGWKSNLSLVIKIRK----DILRFLDAERDISVVKSSLKPGDIIH
Squaca_4       FEHSVKGWKNNASLVVKIRK----DILRFLDAERDISVVKSSLKPGDIIH
Acisin_4       FQHSVEGWKINSSLVLEIRK----DILRFLDAEKDVSVVKSSFKPGDVIH
Hetzeb_4       FEHTVEGWKNNASLVVKIRK----DILRFLDAERDISVVKSSLKPGDIIH
Petmar_4       -------------------K----DILKFLDADRDVSILQAQVQPGDLVH
Letcam_4       DVHLSIGSRCVHLPCCASSK----DVLKFLDADRDVSILRAQVQPGDLVH
Eptbur_4       VNDVSSSWHFNATLAESIRK----DVLRFLDPERDMFLLQREVKPGVRIP
Latcha_1       VLQQGSEWRRNQTAVALFRC----FRER-----------------GKDL-
Angang_1       VLRQGQVWKKNQTRIRLFRH----LLKVCCNPRKMFAVTKENSPPGKVIW
Angang_1b      VLQLGQTWKKNETTVHLYRE----LLMDCCDPREKFAVTRGNTPLGTVLW
Astmex_1       -LRQGQTWSKNQTGIDLYRK----LLTECCDPKKMFAVTKENSPIGKTLW
Orylat_1       -LRQGDVWRKNQTGIDLYRD----LLTKCCNPKKMFAVTKENSPIGKVLW
Acisin_1       -LRQRKLWKKNQTGISIYRK----LVEECCDPRNLFAVTKQNTPLGKNLW
Lepocu_1       -LRQGKAWNKNFTGISMFRK----LLRDCCDPRNLFSVTKQNAPPGKILW
Hetzeb_1       EILHQAKWKRNQTAITAFRR----LLEQCCDAQRQFAVTKLNSPMGKSLW
Squaca_1       EILQQAKWKRNQTAIAAFRR----LLEQCCDAQRLFAVTKLNSPMGKSLW
Calmil_1       DVLHQPVWRRNQTATTAFRR----LLENCCETKRMFAVTQHNSPMGKNLW
Petmar_1       ------------------RQ----KIEKCCDPLKSFALTQTNSPVGKKIW
Letcam_1       --------------------------------------------------
Eptbur_1       AKIKDSLWTHNETALQNFKK----LLAQSCSADQ-LVVTQRTHPSGSMLW
Latcha_5       RRMQVCRWEINETEAKAFKS----YLSRCCNAPSFLFTTQKNTPLGTKLK
Letcam_5       --------------------------------------------------
Lepocu_5       RSLQVCKWELNKTEANAFKT----SLSRCCNAPSFLFTTKRNTPSGTKLR
Squaca_5       KTLQLCKWERNKTAANIFKS----HLTRCCNAPAFLFTTQKNTPKGIKLK
Angang_5       RNLQICKWEQSKAETNAFKS----SLSHCCNAPSFLFTTKRNTPSGTKLR
Acisin_5       KSLQACKWQMNKAEANAFKA----SLSRCCNAPAFMFTTQRNTPFGTKLR
Orylat_5       RNLQVCKWEQSKEETSNFKL----SLSRCCNAPSFLFTTKRNTPAGSKLR
Astmex_5       RNLQACMWGQNKEETANFKM----SLSRCCNAPSFLFTTRRNTPSGTKLR
Angang_5b      KSLQMCRWEQNKVEANAFRN----LLSRCCNAPSFLFTTKKNTPSGTKLR
Anates_5       RNLQVCRWEQNKEETSNFKM----SLSRCCNAPSFLFTTKRNTPAGTKLR
Calmil_5       RTLQLCKWELNKTEANTAR------LTNCCNAIQNFTVTQVNTALGANLT
Hetzeb_5       RTLQLCKWERNETAANIFKS----HLTRCCNAPVFLFTTQKNTPKGIKLK
Eptbur_5       ------------------------MLQRCCNAPSLLFVTQSNTPISSTVH
Latcha_6       NELQKCSWVIRPEEHEKFRS----ELSVCCNAVRNFIVSQNNTPLGTNMS
Latmen_6       NELQKCSWVIRPEEHEKFRS----ELSVCCNAVRNFIVSQNNTPLGTNMS
```

```
Squaca_6       AQLQQCQWHKQELEAEKFRS----ELKRCCYAAYDGLVTQRNTPVGTELT
Calmil_6       AKIHQCKWGKQKRAVENFRW----ELRKCCRTLSGSFVTQRNTPVGTELF
Hetzeb_6       AKLQKCQWHKQEVAAEELRS----ELKRCCHAAYNYLVTQKNMPVGTELI
Lepocu_8       AQVHSQTWRKQESRLQTFRA----QLNMKCQGFSRAIITQANTLLGSKVT
Acisin_8       LQLEAQGWERWEESYQKLRS----ALSRDCHGVSDAVITQKNTPLGSKVF
Angang_8       VELYSYAWKKQNANFNKFRS----QLSSRCRGVSTAIVTQNNTPLDSKIY
Orylat_8       LELYSEPWKKQEDNYHQFRS----QLNSKCHGLEKAIITQANTPQGTKLV
Astmex_8       LKSYSPRWKRREANFSKFRS----LLSSSCHAVSKAVVTQSNTPVGSKVV
Amical_8       LQTHIVDWEKQEHKFQSFRA----QLNSRCDGLSKAIISQTNTPLGSKVV
Anates_8       IEQYSHTWKRQEDNYREFRS----QLNSRCHGYDKAIITQANTPLGSRLL
Lepocu_8b      LQTHIVDWEKQEHKFQSFRA----QLNSRCDGLSKAIISQTNTPLGSKVV
Lepocu_7       TKLMSCPWTANPTLQERLRS----VLRSCCDAQQNLSLTQKNTRLGQRLH
Lepocu_7b      TQLMRCPWTLNLTQLEQQRT----ELRLSCNATGRLVVTQDNTPLGGTIT
Angang_b       KKIMSCPWTSNLTQQIIHKM----DLHTECNASGTLFVTRENTHLGQTLT
Latcha_7       NRKRCGELRENLTSQELPKET---DLNKCSNLTQRLVLTRENAPIGHEIE
Angang_7       RALMSCPHESNITQRERHRV----ELRSCCNATGSLFLTRQNTREGQRIK
Eptbur_7       ------------------RA----MLRSCCDARTGLVLTRGDAAERPGLA
Petmar_7       ------------------RA----MLRSCCDARTGLVLTRGDAAERPGLA
Letcam_7       ------------------------MLRSCCDARAGLVLTRGDAAERPGLA
Petmar_7b      --------------------------------------------------
Letcam_7r      ISVNKNKWKANASKKEAYRSVCVCYMKCFTDGSSNIVVTQNNSPLGSNIS
Petmar_7r      ISVNKNRWKANASKKETYKT----YMKCFTDGSSNIVVTQNNSPLGSNIS
Eptbur_7r      ISVNKNRWKANASKKETYKT----YMKCFTDGSSNIVVTQNNSPLGSNIS

Angang_3       YDYSSHKYVFSIGEN----LMSLLPES--------SPIVPE---RRFNVC
Lepocu_3       YDYSSHKYVFSISNN----FRSLLPDT--------SPIL-N---KQYNVC
Latcha_3       YDYSSHKYVFSISNH----FKSLLPDV--------SPIL-N---KCYNIC
Squaca_3       YDYSNHKYVFSVSNS----FRSLLPEV--------PPIL-K---MHYNTC
Astmex_3       YDYSSHKYVFSIGEN----FRSLLPDS--------SPVL-N---KRYNTC
Orylat_3       FDYSSHKYVYSIGEN----FRSLLPEV--------SPIL-N---RHYNVC
Hetzeb_3       YDYSNHKYVFSVSNS----FRSLLPDV--------PPIL-K---MHYNTC
Anates_3       YDYSSHKYVFSIGEN----FRSLLPDA--------SPIL-N---KHYNVC
Acisin_3       YDYSSHKYVFSISNN----FKSLLPDA--------SPVM-N---KHYNVC
Masarm_3       YDYSSHKYVFSIGEN----FYSLVPEV--------SPIL-N---KHYNIC
Calmil_3       FDYSSHKYVFSISNN----FRMLLPEV--------SPLL-K---MHYNTC
Orymel_9       FDYSSHKYVFSISNN----FKSLLPDI--------SPIL-N---KHYSVC
Anates_9       FDYSSHKYVFSISNN----FKSLLPDT--------SPIH-N---KHYSMC
Masarm_9       FDYSSHKYVFSISNN----FKSLLPDT--------SPIL-N---KHYSMC
Orylat_9       FDYSSHKYVFSISNN----FKSLLPEI--------SPIL-N---KHYSVC
Eptbur_3/9     FDYSPKHYVFVVSAA----LHSLLPIL--------PPFH-G---RHFNSC
Letcam_3/9     ------------------------LPS-----------------------
Anates_2       YVFDRH-STTNISEN----LYRLLPTV--------SPMK-N---QHHRRC
Latcha_2       YVFDRD-STMNISQN----LYKLLPRV--------SPLK-N---RHFQNC
Amical_2       YIFDRD-STMNISET----LYQLIPKV--------SPMK-Q---QHYRKC
Acisin_2       YVFDRE-RTMNISEN----LYRLLPSS--------PPLK-K---QHFKRC
Angang_2       YIFDRQ-STVNVSES----LYRLLPTV--------SPMK-D---RHYQRC
Astmex_2       YVFDRQ-STTNISEN----LYRLLPTV--------SPMK-N---QHYERC
Orylat_2       YVFDRH-STTNISEN----LYRLLPTV--------SPMK-N---QHHRRC
Hetzeb_2       YIFDRD-RTLNISHS----LYELLPRT--------SPMK-N---KHFKQC
Lepocu_2       YIFDRE-STMNVSET----LYQLIPKV--------SPMK-K---QHYRQC
Latcha_4       YVLDRR-RTLNISHN----LYSLLPEV--------SPMK-N---RRFKTC
Astmex_4-A     YILDRR-RTLNVSHT----LHSLLPEV--------SPLK-N---KRFKTC
Angang_4       YVLERR-RTLNVSRA----LHSLLPAV--------SPMK-N---RRFRTC
Lepocu_4       YVLDRR-RTLNISHN----LHSLLPEV--------SPMK-N---RRFKTC
Amical_4       YVLDRR-RTLNISHN----LHSLLPEV--------SPMK-N---RRFKTC
Calmil_4       YVLDRR-RTLNISRN----LHSLLPEV--------SPMK-N---KRFNAC
Squaca_4       YVLDRR-RTLNISQN----LHSLLPEV--------SPMK-N---KRFSTC
Acisin_4       YVLDRR-RTLNISHN----LHSLLPEV--------SPMK-N---RRFKTC
Hetzeb_4       YVLDRR-RTLNISHN----LHSLLPEV--------SPMK-N---KRFSTC
Petmar_4       YIFDRG-RTTNVSHS----LHALLPTA--------PPLR-G---GRYGSC
Letcam_4       YIFDRG-RTTNVSHS----LHALLPTA--------PPLR-G---GRYGSC
Eptbur_4       YILNRG-HFATVTRT----VHSLLPSA--------PLLH-G---RRFATC
Latcha_1       ------------------------TETD--------TPFQ-----LPLKKC
Angang_1       YDGELY-HSRTVKND----TYALFVQE--------MPLQ-----APLKRC
Angang_1b      YDGELF-YFHTVSNH----TYPLFVQE--------SPIK-----VPLKKC
Astmex_1       YDGEFY-HYHTVSNE----TYPFFVQD--------TPLQ-----LPLKKC
Orylat_1       YDGEIY-HSHTVNSE----TYQLFIKR--------RGAA-----SSCRRC
Acisin_1       YDGELF-HSLTVNND----TYSLFIKE--------NPLQ-----LPLKRC
Lepocu_1       YDGEFY-YSHTVNND----SYSLFIEE--------TPFQ-----QPLKKC
Hetzeb_1       FDGEFL-YSLTVNNE----IFSMFPQD--------TPLQ-----LPLKKC
Squaca_1       FDGEFF-YSLAVNNE----IFSMFPQD--------TPFQ-----LPLKKC
Calmil_1       FDGEFL-HSLSVNNE----MFAMFPQD--------TPFQ-----IPLKKC
Petmar_1       FDGELF-HWITVDNDTFGLFFCLYLQH--------QPLG-Q---KLLKRC
Letcam_1       --------------------------------------------------
Eptbur_1       YDGEIF-HWLHVDLP----LSQTFPKE--------NPLKAD------GSC
Latcha_5       YEVDSS-GTYNINSE----IFKMFPKD--------MPYS-R---TQFKKC
Letcam_5       --------------------------------------------------
```

```
Lepocu_5       YEVDTS-GILHISSE----IFKMFPND--------MPYT-R---SQFKRC
Squaca_5       YEVDSS-GFLFINAE----VFELFPKE--------MPYS-R---SQFKKC
Angang_5       YEVDTS-GILHISSE----IFKMFPDD--------MPYT-K---SQFKKC
Acisin_5       YEVDTS-GILHINPA----IFKMFPKD--------MPYS-R---SQFKKC
Orylat_5       YEVDTS-GILPITNE----VFKMFPHD--------MPYS-K---SQFKKC
Astmex_5       YEVDTS-GILHISPE----IYKMLPED--------MPYS-K---SQFKKC
Angang_5b      YEVDTS-STLPITSE----IFKMFPDD--------MPYS-K---SQYRKC
Anates_5       YEVDTS-GILPITTE----VFKMFPDD--------MPYS-K---SQYKKC
Calmil_5       YDAQPK-KQITITED----IFNLLPQE--------MPYS-R---SQFKKC
Hetzeb_5       YEVDSS-GFLFIDAE----VFQLFPKE--------MPYS-R---SQFKKC
Eptbur_5       YEQEPV-KNVPIESS----IYYMLPKS--------MPYQ-R---QQLARC
Latcha_6       YEVENG-RTFLITQR----IFKMFPQS--------QPFA-G---YPYNQC
Latmen_6       YEVENG-RTFLITQR----IFKMFPQS--------QPFA-G---YPYNQC
Squaca_6       YDAEPK-KKIKITHP----IFTIFPKD--------SAFS-S---RQYRRC
Calmil_6       YEAEPK-KKIKITPS----IFAIFPKD--------SPFR-G---RSIQRC
Hetzeb_6       YDAESK-NKINITPQ----IFSIFPKN--------SFFS-G---RQYRRC
Lepocu_8       YDGERR-KPVEVTPK----LYSTFPKE--------HPFG-N---VSFQSC
Acisin_8       YDGEKR-RHIKINQK----IFSTFPSE--------TPFV-N---VTFASC
Angang_8       YDGEKR-KPLQVTPK----LYSTFAKE--------QPFE-N---VTWKTC
Orylat_8       YDAERK-RTLVVNAE----VFNTFIKE--------NPFP-N---KTWDTC
Astmex_8       YDGEKT-KPLQVTKA----LFSTFAKE--------QPFG-N---ASWDTC
Amical_8       YDGEKR-KPIQVTPK----LFSTFPKE--------HPFG-N---RTFTSC
Anates_8       YDGDKR-KSLQVSPE----IFSTFAKE--------DPFS-N---KTLDTC
Lepocu_8b      YDGEKR-KPIQVTPK----LFSTFPKE--------HPFG-N---RTFTSC
Lepocu_7       YEVNRT-IWRQVDQE----LFEMLPQS--------SLFR-----AGLSRC
Lepocu_7b      YDAEK--WTRQVDTE----IFNMLPQS--------PPWA-GGPGARLRRC
Angang_b       YEVQRK-STKVVDKM----LYNMLPRA--------APWGIS---RVLGRC
Latcha_7       YEVAE--QRVRVEEP----LFKLLPRF-PPPFYQESPFK-K---APYKRC
Angang_7       YETNRK-KNILVDKS----IFKMLPKS--------TPWRND---SRFQRC
Eptbur_7       FEAAT--KTLHVTPQ----LLSVLPES--------SPLA-G---ELHGRC
Petmar_7       FEAAT--KTLHVTPQ----LLSVLPES--------SPLA-G---ELHGRC
Letcam_7       FEAAA--KTLHVTPQ----LLSVLPEVWPTQVCQSSPLA-G---ELHGRC
Petmar_7b      ------------------------------------PLA-G---ELHGRC
Letcam_7r      YLIGN--AQLTVGTE----LTNIIPMT--------FPGP-E---MKMKTC
Petmar_7r      YVIGN--AQLTVGTE----LTNIIPMT--------FPGP-E---MKMKTC
Eptbur_7r      YVIGN--AQLTVGTE----LTNIIPMT--------FPGP-E---MKMKTC

Angang_3       ALVGNSGVLTGSRCGPEIDRSDFVFR--------CNFAPTE-IFHRDVGR
Lepocu_3       AVVGNSGILTGSQCGPEIDKSDFIFR--------CNFAPTE-IFHKDVGK
Latcha_3       AVVGNSGILTDSQCGAEIDQSDFVFR--------CNFAPTE-IFHKDVGR
Squaca_3       AVVGNSGILTGSGCGAEIDKSDFVFR--------CNFAPTE-TFEKDVGK
Astmex_3       AVVGNSGILTGSRCGPTIDKYDFVFR--------CNFAPTE-VFRRDVGR
Orylat_3       AVVGNSGILTGSLCGAQIEKYDFVFR--------CNFAPTE-IFKKDVGR
Hetzeb_3       AVVGNSGILIGSRCGADIDKSDFVFR--------CNFAPTE-SFEKDVGK
Anates_3       AVVGNSGILTGSRCGPQIEKFDFVFR--------CNFAPTE-IFKKDVGR
Acisin_3       AVVGNSGILTGSKCGSEIDKADFVFR--------CNFAPTE-VFQKDVGR
Masarm_3       AIIGNSGILSGSRCGSQIEKFDFVFR--------CNFAPTE-IFKKDVGR
Calmil_3       AVVGNSGILRGSRCGDSIDKSDFVFR--------CNFAPTE-FFEKDVGR
Orymel_9       AVVGNSGILTGSHCGPEIDQADFVFR--------CNFAPTD-VYSKDVGR
Anates_9       AVVGNSGILTGSHCGPEIDQADFVFR--------CNFAPTD-VYSKDVGK
Masarm_9       AVVGNSGILTGSHCGPEIDQADFVFR--------CNFAPTE-VYSKDVGK
Orylat_9       AVVGNSGILTGSHCGPEIDQADFVFR--------CNFAPTD-VYSKDVGR
Eptbur_3/9     AVVGNGGVLTGSGCGQRIDTADFVIR--------CNFAPTT-GYVHDVGN
Letcam_3/9     ----------------------------------C---------------
Anates_2       AIVGNSGILLNSSCGHEIDSHDFIIR--------CNLAPVE-EYSRDVGW
Latcha_2       AIVGNSGILLNSNCGREIDDHQFVIR--------CNLAPVQ-EYASDVGT
Amical_2       AIVGNSGILLNSRCGEEIDSHDFVIR--------CNLAPVE-EYADDVGL
Acisin_2       AIVGNSGILLNSGCGAEIDSHDFVIR--------CNLAPVE-EYSRDVGL
Angang_2       AIVGNSGILLNSSCGRQIDSHDFVIR--------CNLAPVE-EYAADVGQ
Astmex_2       AIVGNSGILLNSSCGPEIDSHDFVIR--------CNLAPVE-EYAEDVGL
Orylat_2       AIVGNSGILLNSSCGSEIDAHDFVIR--------CNLAPVD-EYSQDVGR
Hetzeb_2       AIVGNSGILLNSGCGQEIDAHEFVIR--------CNLAPVE-EYAHDVGL
Lepocu_2       AIVGNSGILLKSRCGAEIDSHDFVIR--------CNLAPVE-EYRADVGR
Latcha_4       AVVGNSGILLGSSCGKEIDSHEFVIR--------CNLAPLV-EFAEDVGI
Astmex_4-A     AVVGNSGVLLKSGCGKEIDGHDFVIR--------CNLAPLA-EFAEDVGL
Angang_4       AVVGNSGVLLNSGCGREIDGHDFVIR--------CNLAPLE-EFAEDVGL
Lepocu_4       AVVGNSGILINSGCGKEIDNHDFVIR--------CNLAPLA-EFSEDVGL
Amical_4       AVVGNSGILINSGCGREIDSHDFVIR--------CNLAPLA-EFSEDVGL
Calmil_4       AVVGNSGILLGSGCGKEIDSHEFVIR--------CNLAPLV-EYTDDVGS
Squaca_4       AVVGNSGILLGSGCGKEIDSHEFVIR--------CNLAPLV-EYADDVGL
Acisin_4       AVVGNSGILLDSGCGNEIDSHDFVIR--------CNLAPLS-EFAEDVGM
Hetzeb_4       AVVGNSGILLGSGCGKEIDSHEFVIR--------CNLAPLM-EYTDDVGL
Petmar_4       AVVGNSGILEGSACGEEIDQHDFVIR--------CNLAPVE-GYERDVGS
Letcam_4       AVVGNSGILEGSACGEEIDQHDFVIR--------CNLAPVE-GYERDVGS
Eptbur_4       ALVGNSGILRGSHCGSEIDRHDYIVR--------CNMAPVT-PFKRDVGI
Latcha_1       SVVGNGGILKKSGCGRQIDNADFVMR--------CNLPPLSNEYRNDAGS
Angang_1       AVVGNGGVLKRSGCGRDIDGADFVLR--------CNLPPLSKEYTEDVGT
```

```
Angang_1b      AVVGNGGILKGSGCGGHIDRADFVMR--------CNLPPLSEDYTQDVGS
Astmex_1       SVVGNGGVLKNSGCGKDIDQADFVMR--------CNLPPLSKKYTDDVGT
Orylat_1       AVVGNGGILKGSKKGKEIDHHHYIFR--------CNLPPLSREYVEDVGT
Acisin_1       SVVGNGGILKNSGCGKHIDQTDFIMR--------CNLPPLSKQYTEDVGT
Lepocu_1       SVVGNGGILKHSGCGKEIDRADFIMR--------CNLPPLSEDYREDVGT
Hetzeb_1       SVVGNGGILKESGCGSQIDKVDFVMR--------CNLPPLSNEYTKDAGY
Squaca_1       SVVGNGGILKKSNCGSQIDKADFVMR--------CNLPPLSNEYTKDAGY
Calmil_1       SVVGNGGILKRSACGDKIDKADFVMR--------CNLPPLSNEYARDVGH
Petmar_1       AIVGNSAIMLGHGCGKDIDEADVIFRVICFCPLRCNLPPITKQYEPDIGT
Letcam_1       ---------------------DLIVS------------------------
Eptbur_1       ALVGNGGILKDSGCGNEIDSVDFIIR--------CNLPPLGEKYSTDVGT
Latcha_5       AVIGNGGILKSSGCGREIDSMDFVFR--------CNLPPISEKYVLDVGF
Letcam_5       --------------------------------------------------
Lepocu_5       AVIGNGGIIKNSKCGKEIDSADFVFR--------CNIPPISEKYAVDVGY
Squaca_5       AVVGNGGILKNSKCGNEIDATDFVFR--------CNLPPISEKYTMDVGL
Angang_5       AVIGNGGIIKNSKCGKEIDSADFVFR--------CNIPPISDKYAEDVGY
Acisin_5       AVIGNGGILKNSKCGKAIDSADFVFR--------CNLPPISEEYAADVGY
Orylat_5       AVIGNGGIIKNSKCGKEIDSADFVFR--------CNIPPIKEKYSTDVGS
Astmex_5       AVIGNGGIIKNSKCGKEIDSADFVFR--------CNIPPISDKYAADVGT
Angang_5b      AVIGNGGIIKNSKCGKEIDSADFVFR--------CNIPPISEKYAVDVGY
Anates_5       AVIGNGGIIKNSKCGKEIDSADFVFR--------CNIPPINEKYSADVGT
Calmil_5       AVVGNGGILKKSECGKEINSADFVFS--------CNLPPLSNEYARDVGH
Hetzeb_5       AVVGNGGILTNSRCGNEIDSADFVFR--------CNLPPISEKYTMDVGL
Eptbur_5       AVVGNGGILHNSRCGSEIDAMEFVFR--------CNLPPVSAEFAKDVGS
Latcha_6       AVVGNGGILKNSACGTEIDQADFVFR--------CNLPPTLGNISIDVGS
Latmen_6       AVVGNGGILKNSACGTEIDQADFVFR--------CNLPPTLGNISIDVGS
Squaca_6       AVIGNGGILANSSCGAEIDQADFVFR--------CNLPPVGGDFAQDVGT
Calmil_6       AVVGNAGILHNSSCGAEIDQADFVFR--------CNLPPMGGNFTKDVGS
Hetzeb_6       AVIGNGGILANSSCGAEIDQADFVFR--------CNLPPMGGEFTRDIGT
Lepocu_8       AVVGNGGILANSSCGEEIDGAQFVIR--------CNLPPVDRKYQDDVGN
Acisin_8       AVVGNGGILLDSDCGEEIDRADFIIR--------CNLPPVKGRYHSDVGN
Angang_8       AVVGNGGILVNSSCGEAIDSAHFVFR--------CNLPPLDKAYQKDVGN
Orylat_8       AVVGNGGILANSSCGKTIDSAQFVIR--------CNLPPLSNGFEKDVGI
Astmex_8       AVVGNGGILVNSSCAEKINSANLVIR--------CNLPPLGNGYEKDVGN
Amical_8       AVVGNGGILMNSSCGGEIDEAQFVIR--------CNLPPVDHGYQRDVGN
Anates_8       AVIGNGGILTNSSCGNMIDSAQFVIR--------CNLPPLDNGYEKHVGI
Lepocu_8b      AVVGNGGILMNSSCGGEIDEAQFVIR--------CNLPPVDHGYQRDVGN
Lepocu_7       AVVGNGGILKNSNCGAEIDRADYIIR--------INLPPLA-QSAADVGL
Lepocu_7b      AVVGNGGILRNSSCGAHIDRADFVIR--------FNLPPL--NYSRDVGV
Angang_b       AVVGNGGILKNSSCGEKINSADFVIR--------LNLPPM--NYSSDVGV
Latcha_7       AVVGNAGILLNSGCGRRIDQADFVFR--------CNLPPL--NYSKDVGS
Angang_7       AVVGNGGILRNSSCGAEIDSADIVFR--------LNMAPI--NNSRDVGV
Eptbur_7       AVVGNSGALLGSRCGRSIDNADFVIS---------VQPPSGVGKLH-AGR
Petmar_7       AVVGNSGALLGSRCGRSIDNADFVIR---------VQPPSGVGKLH-AGR
Letcam_7       AVVGNSGALLGSRCGRSIDNADFVIR-------RCNLPPVSGNFTRDVGN
Petmar_7b      AVVGNSGALLGSRCGRSIDNADFVIR-------RCNLPPVSGNFTRDVGN
Letcam_7r      AVVGNSGFLLNSKCGKEIDKSDFIMR--------CNLPNISKKYSMDVGE
Petmar_7r      AVVGNSGFLLNSKCGKEIDKSDFIMR--------CNLPNISKKYSMDVGE
Eptbur_7r      AVVGNSGFLLNSKCGKEIDKSDFIMR--------CNLPNISKKYSMDVGE

Angang_3       RTN--LTTFNPSILEK--YYNNLLTIQDRNNFFLSLKKLD-GVVLWIPAF
Lepocu_3       KTN--LTTFNPSILEK--YYNNLLTIQDRNNFFLSLKKLD-GAILWIPAF
Latcha_3       KTN--LTTFNPSILEK--YYNNLLTIQDRNNFFLSLKKLD-GAILWIPAF
Squaca_3       KTN--LTTFNPSILEK--YYNNLLTIQDRNKFFLNLKKLD-GAILWIPAF
Astmex_3       RTN--LTTFNPSILEK--YYNNLLTIQDRNNFFLSLKKLD-RAVLWIPAF
Orylat_3       RTN--MTTFNPSILEK--YYNNLLTVQDRNNFFLSLKKLD-GAILWIPAF
Hetzeb_3       KTN--LTTFNPSILEK--YYNNLLTIQDRNKFFLNLKKLD-GAILWIPAF
Anates_3       RTN--LTTFNPSILEK--YYNNLLTVQDRNNFFLSLKKLD-GAILWIPAF
Acisin_3       KTN--LTTFNPSILEK--YYNNLLTIQDRNNFFLSLKKLD-GAILWIPAF
Masarm_3       RTN--MTTFNPSILEK--YYNNLLTVQDRNNFFLSLKKLD-GAILWIPAF
Calmil_3       KTN--LTTFNPSILEK--YYNNLMAIQDRNKFFLNLKKLD-GAILWIPAF
Orymel_9       KTN--MTTFNPSILER--YYNNLLTIQDRNNFFLHLKKLE-GAILWIPAF
Anates_9       KTN--LTTFNPSILER--YYNNLLTIQDRNNFFLNLKKLE-GAILWIPAF
Masarm_9       KTN--LTTFNPSILER--YYNNLLTIQDRNNFFLSLKKLE-GAILWIPAF
Orylat_9       KTN--MTTFNPSILER--YYNNLLTIQDRNNFFLHLKKLE-GAILWIPAF
Eptbur_3/9     RTS--LTTFNPSIIDG--RYGGLLTANDRERFLNRLQELD-GAVLWIPAF
Letcam_3/9     --------------------------------------------------
Anates_2       QTN--LVTMNPSVVQR--AFQDLVSEEWRARFLRRLQSLS-GSVLWIPAF
Latcha_2       KTD--FVTMNPSVIQR--AFEDLVNETWKEKFLQRLQSLN-GSIFWIPAF
Amical_2       RTS--LVTMNPSVVQR--AFQDLVSESWRERFLQRLQSLN-GSVLWIPAF
Acisin_2       RTG--LVTMNPSVVQR--AFEDLVSEAWREKLLQRLRGLS-GSVLWIPAF
Angang_2       RTS--LVTMNPSVVER--AFRDLSSEAWRERFVRRLRALE-GSVLWIPAF
Astmex_2       RSS--LVTMNPSVVQR--AFQDLSSKEWRQRFVRRLQALS-GSVLWIPAF
Orylat_2       QTN--LVTMNPSVVQR--AFQDLVSEEWKQRFLQRLQSLG-GSVLWIPAF
Hetzeb_2       KTD--LVTMNPSVVQR--AFEDLKNETWKEKLLHRLKMLD-GGILWIPAF
Lepocu_2       RTS--LVTMNPSVVQR--AFQDLASSHWRERFLERLSSLN-GSVLWIPSF
Latcha_4       ESD--FITMNPSVIQR--AFGGFHNETDREKFVYRLAMLN-DSVLWIPAF
```

```
Astmex_4-A     KSD--FTTMNPSVIQR--VYGGLRNETQRERFVERLSMLN-DSVLWIPAF
Angang_4       RSD--FTTMNPSVIQR--AYGGLRNDSDRERFVRRLGALN-DSVLWIPAF
Lepocu_4       RSD--FITMNPSVIQR--AFGGFKNETDRERFVQRLTLLN-DSVLWIPAF
Amical_4       RSD--FITMNPSVIQR--AFGGFKNETDREKFVRRLTMLN-DSVLWIPAF
Calmil_4       KSD--FVTMNPSVVQR--AFGSLQNETDRENFVRRLAVLN-DSVLWIPAF
Squaca_4       KSD--FVTMNPSVVQR--AFGSLQSETDREKFVDRLAVLN-DSVLWIPAF
Acisin_4       SSD--FITMNPSVIQR--VYGGFKNESDREKFVQRLAMLN-GSVLWIPAF
Hetzeb_4       KSD--FVTMNPSVVQR--AFGSLRSETDREKFVDRLAVLN-DSVLWIPAF
Petmar_4       RVD--FVTMNPSVVER--TYGGLRTKADHDRFGRRLRALN-SSILWIPAF
Letcam_4       RVD--FVTMNPSVVER--TYGGLRTQADHDRFGRRLRALN-GSVLWIPAF
Eptbur_4       RTD--FLTMNPSILPR--YYGGLRNATLRDRFVARLRALG-KGIFWIPAF
Latcha_1       KSH--LVTANPSIIEK--RFQNLL--WSRKAFVDSVQVYN-HSYIYMPAF
Angang_1       RTH--LVTANPSIIEK--RFHNLL--WSRKAFVDSMKAYG-PSYVYMPAF
Angang_1b      RTH--LVTANPSIIVE--RFQGLM--WSRKAFVESMRAYG-RSFVYMPAF
Astmex_1       RTQ--LVTANPSIIEK--SFQNLL--WSRKAFVESMKAYG-SSYIYMPAF
Orylat_1       KTH--LVTANPSIIEK--RFQNLV--WSRKTFVDSMKVYG-SSYIYMPAF
Acisin_1       KAH--LVTANPSIIEK--RFQNLL--WSRRAFVEYVKAYD-SSFIYMPAF
Lepocu_1       KTH--LVTANPSIIEK--RFQNLL--WSRKKFVESVKVYG-SSYIYMPAF
Hetzeb_1       RTH--LVTANPSIIEK--RFQNLL--WSRKSFVESMKVYG-RSYIYMPAF
Squaca_1       RTH--LVTANPSIIEK--RFQNLL--WSRKSFVESMKVYG-RSYVYMPAF
Calmil_1       RTQ--LVTANPSIIKK--RYQNLM--WSRKAFVESMKVYG-RSYIYMPGF
Petmar_1       KTH--LVTANPSIIQM--RNQNLL--WSRRAFSESMLAYG-HSFVYMPAF
Letcam_1       -----------------RYQNLL--WSRRAFSESMRAYG-HSFVYMPAF
Eptbur_1       KTS--LVTANPSIIGN--RFSNFI--PSSHSFLDAISAYG-SAQILFPAF
Latcha_5       KTD--IVTINPSIIAD--RFHKLE--KWRRPFYTALQAYE-NASVVLPAF
Letcam_5       ------------------------------PFAEALEVYG-NASLVLPAF
Lepocu_5       KTG--IVTINPSIITE--RFQKLE--KWRRPFYEVLQAYE-NASIVLPAF
Squaca_5       KTD--IVTINPSIVTD--RFQKLE--KWRRPFYEVLQAYE-NASVVMPAF
Angang_5       KTD--IVTINPSIITE--RFQKLE--KWRKPFYEVLQNYE-NSSVVLPAF
Acisin_5       KTD--IVTINPSIITE--RFQKLE--KWRKPFYEVLQTYE-NSSVVLPAF
Orylat_5       KTD--LVTINPSIITE--RFQKLE--KWRRPFYDVLQNYE-NSSVVLPAF
Astmex_5       KTD--LVTINPSIITE--RFQKLE--KWRKPFYDVLLNYE-NSSVVLPAF
Angang_5b      KTN--MVTVNPSIIID--RFQKLE--KWRKPFFKALQTYN-DSSVVLPAF
Anates_5       KTD--LVTINPSIITE--RFQKLE--KWRRPFYEVLQNYE-NSSVVLPAF
Calmil_5       RTQ--LVTANPSIIKK--RFQKLD--KWRRPFVELLQVYE-NTSVVMPAF
Hetzeb_5       KTD--IVTINPSIITE--RFQKLE--KWRRPFYEVLQAYE-NASVVMPAF
Eptbur_5       KTD--IVTINPSIIID--RYDSLM--QRHGPFLARIRSYG-NASLLLPAF
Latcha_6       KTS--LVTLNPSIITH--RFGKLN--EKRKPFVETVSHYG-EAFLLLPAF
Latmen_6       KTS--LVTLNPSIITH--RFGKLN--EKRKPFVETVSHYG-EAFLLLPAF
Squaca_6       KTN--LVTANPSIIAE--RYAELH--DRRKPFFTNLTVYN-EALLLLPAF
Calmil_6       KTH--LVTANPKVIIE--RYAELH--KRRKPFANTLVIYN-DALLLFPAF
Hetzeb_6       KTN--LVTANPSIILE--RYAKLQ--DRRRSFFTNLAVYD-EALLLLPAF
Lepocu_8       KTD--LVTANPSILLE--RFEGLM--ELRRPFVESLGDYG-QPMLALPAF
Acisin_8       KTD--LVTANPSILLE--KFAALS--EWRRPFADCVSRYGAGAMLALPAF
Angang_8       KTN--LVTANPSILIE--KFEGLM--EYRRPFVESLSDYG-EALLLLPAF
Orylat_8       KSD--IVTANPSIITE--KYFSLM--RHRRPFAEAMRIYG-NSMVLLPAF
Astmex_8       KTS--LVTANPSILIE--KFSGLM--ERRRPFVESLRPYG-DSLLLLPAF
Amical_8       KTS--LVTANPSILLD--KFNGLM--ELRRPFVDSLGSYG-DPLLVLPAF
Anates_8       KTD--LVTANPSILIE--KYGALM--ARRRPFVESLRRYG-NSLLLLPAF
Lepocu_8b      KTS--LVTANPSILLD--KFNGLM--ELRRPFVDSLGSYG-DPLLVLPAF
Lepocu_7       KTS--LVSANPSQINN--VYRRLS--YERRPFVDTVSSFG-LAPLLLPAF
Lepocu_7b      KSS--LVTVNPSQIIN--SYRNLN--YARRPFVQRVSTYE-NAHLVLPAF
Angang_b       KSS--LVTINPSQITQ--SFMGLC--NARKPFVDKAAAHG-NAYLAMAPF
Latcha_7       KTD--LVTANPSIIIQ--KYHGLK--ESRRLFVERMKVYK-DALILMAAF
Angang_7       KTS--LVTINPSQIRV--GYPDLQ--KRPQPLVERVSAYG-DAPLLMPAF
Eptbur_7       RQPQPHGDAQPQHH-Q---YGGLHSSESRSRFVSDVTDYG-DALLLVPAL
Petmar_7       RQPQPHGDAQPQHHQD----GGLHSSESRSRFVSDVTDYG-DALLLVPAL
Letcam_7       RSH--MVTLNPSIIKE---YGGLHSSESRSRFVSDVTDYG-DALLLVPAL
Petmar_7b      RSH--MVTLNPSIIKERGRYGGLHSSESRSRFVSDVTDYG-DALLLVPAL
Letcam_7r      KTN--LVFVKPSLIVN--KYSGLR--DSRIKFVEDMQKYGSDFLF-TAFN
Petmar_7r      KTN--LVFVKPSLIVN--KYSGLR--DSRINFVEDMQNYGSDFLF-TAFN
Eptbur_7r      KTN--LVFVKPSLIVN--KYSGLR--DSRINFVEDMQNYGSDFLF-TAFN

Angang_3       FFHTSATVTRT-LVDFFVEHRG-QLKVQLAWPGNIMQYVNRYWKTRQLSP
Lepocu_3       FFHTSATVTRT-LVDFFVEHKG-QLKVQLAWPGNIMQYVNRYWKTKHLAP
Latcha_3       FFHTSATVTRT-LVDFFVEHKG-QLKLQLAWPGNIMQHVNRYWKLKHLSP
Squaca_3       FFHTSAPVTRT-LVDFFMEHRA-QLKIQLAWPGNIMQHINKYWKTKQLSP
Astmex_3       FFHTSATVTRT-LVDFFVEHQG-QLRVQLAWPGNIMQYVNRYWKTKQLSP
Orylat_3       FFHTSATVTRT-LVDFFVEHRG-QLKVQLAWPGNIMQYINNYWKTKQLSP
Hetzeb_3       FFHTSAPVTRT-LVDFFIEHRA-QLKVQLAWPGNIMQHINKYWKTKQLSP
Anates_3       FFHTSATVTRT-LVDFFVEHRG-QLNVQLAWPGNIMQYINNYWKTKQLSP
Acisin_3       FFHTSATVTRT-LVDFFVEHKG-QLKVKLAWPGNIMQHVNRYWKTKHLAP
Masarm_3       FFHTSATVTRT-LVDFFVEHRG-QLKVQLAWPGNIMQYINNYWKTKQLSP
Calmil_3       FFHSSALVTRT-LVDFFVEHRR-QLKVELAWPGNIMQYVNKYWKTKQLSP
Orymel_9       FLHTSATVTRT-LVDFFVEHKG-QLKVELAWPGNIMHNVNKYWKTKNLSP
Anates_9       FLHTSATVTRT-LVDFFVEHKG-QLKVELAWPGNIMHDVNKYWKTKNLSP
Masarm_9       FLHTSATVTRT-LVDFFVEHKG-QLKVELAWPGNIMHDVNKYWKTKNLSP
```

```
Orylat_9       FLHTSATVTRT-LVDFFVEHKG-QLKVELAWPGNIMHNVNKYWKTKNLSP
Eptbur_3/9     LFHTSAVITRT-LVDFFLENKS-RLGVQLAWPGPLVGHITRFWRTRALAP
Letcam_3/9     ------------------------------P--------RFWRTKGLSP
Anates_2       MAKGGEERVEW-ALRLILLHTV-DVRTAFPSL-RLLHAVRGYWLTNNVHI
Latcha_2       MAKGGEERVEW-VNDLIIKSGI-NVQTAYPSL-RLLHAVRGYWLTNKVLI
Amical_2       MAKGGEERVEW-ANDLILKHHI-NVRTAFPSL-RLLHAVRGYWLTNKVPI
Acisin_2       MSKGGEDRVEC-VNDLILKHHL-DVQTAFPSL-RLLHAVRGYWLTNKVLI
Angang_2       MAKGGEDRVEW-ATRLILRHAL-NVRTAFPSL-RLLHAVRGYWLTNKVLI
Astmex_2       MAKSGEDRVEW-AVHLILSHTV-SVHAAFPSL-RLLHAVRGYWLTNNIQI
Orylat_2       MAKGGEERVEW-ALRLILRHTV-DVRTAFPSL-RLLHAVRGYWLTNNVHI
Hetzeb_2       MAKGGEERVEW-VNNLIIKHKI-RVQTAYPSL-RLLHAVRGYWLTNRILI
Lepocu_2       MAKGGEERVQW-ASELILRHGI-NVRTAFPSL-RLLHAVRGYWLTNKVPI
Latcha_4       MAKGGEKHVEW-VNELILKNKL-KVRTAYPSL-RLIHAVRG---------
Astmex_4-A     MVKGGEKHVEG-VNELILKRKL-PLRTAYPSL-RLIHAVRGYWLTNKVNI
Angang_4       MVKGGERHVEC-VNDLILRHRL-RVRPPTPRS-GLIHAVRGYWLTNKVNI
Lepocu_4       MVKGGEKHVEC-VNELILKNKL-KVRTAYPSL-RLIHAVRGYWLTNKINI
Amical_4       MVKGGEKHVEC-VNELILKNKL-KVRTAYPSL-RLIHAVRGYWLTNKINI
Calmil_4       MVKGGERHVEW-VNELILKNKL-SIQTAYPSL-RLIHAVRGYWLTNKVYI
Squaca_4       MVKGGEKHVEY-VNELILKNRL-SIQTAYPSL-RLIHAVRGYWLTNKVYI
Acisin_4       MVKGGEKHVEW-VNELILKNKL-KVRTAYPSL-RLIHAVRGYWLTNKINI
Hetzeb_4       MVKGGEKHVEY-VNELILKNRL-SIQTAYPSL-RLIHAVRGYWLTNKIYI
Petmar_4       MAKGGEQHVEI-VNNLLLTLGL-PLRAAFPSL-RLMHAVRGYWLTNKVYI
Letcam_4       MAKGGERHVES-VNNLLLTLGL-PLRAAFPSL-RLMHAVRGYWLTNKVYI
Eptbur_4       MVPPERRWVDA-VTDLIAHEHL-SLHPAMPSL-RLMHAVRGYWLSNHVDI
Latcha_1       SMKTGTELSLR-AYYTLSDFSA-NQMVLFANP-DFLRHAAKFWKNKGVHA
Angang_1       TMKPGTAPSLR-AYHALADAGS-NQTVLFANP-DFLRSVGQFWKGRGIHA
Angang_1b      SSRPGTDPSLR-AGHALADSGS-EQTVLFASP-DFLRSVERFWKGRGVRA
Astmex_1       SMKPGTEPSLR-AYHTLADTAS-NQTVLFANP-DFLKNVGRFWKSRGVHG
Orylat_1       SMAPGTDPSLR-AYFALTDVPS-NLTMLFANP-DFLLSVSKFWKAHGVHA
Acisin_1       SMKPGTDPSIR-VYHTLSDVSA-NQTVLFANP-DFLRNVGKFWKSRGIHA
Lepocu_1       SMKPGTDPSFR-AYHALSDIGA-NQTVVFANP-DFLKNVGKFWKSRGIHA
Hetzeb_1       SMKTGTDPSFR-TYYTLSDFSA-NQTILFANP-DFLRNVEKFWKSKGIHA
Squaca_1       SMKTGTDPSFR-TYYTLSDFSA-NQTILFANP-DFLRNVEKFWKSKGIHA
Calmil_1       SMKAGTEPSFR-TYYTLSDVNA-NQTIIFANP-DFLRNVQKFWKGKGVHA
Petmar_1       SMRAATESSLR-VYHTLHDCHQ-NQTTVFANP-GFLHSAGTFWMDNGVRA
Letcam_1       SMRAATESSLR-VYHTLHDCHQ-NQTTVFANP-SFLHSAGTFWTENGVRA
Eptbur_1       STSLGTDPSLK-AHAVIVRANS-AQSAIFMNP-DFLHNASFFWRARGLHA
Latcha_5       YNTRNTDVSFR-VKYVLDDFES-QQSVYFFHP-QYLLNASRFWSNQGVRA
Letcam_5       YVGRNTELALR-VRYTVRRQGL-PRPVLFFHP-AYLASVGAFWRARGVRA
Lepocu_5       YNTRNTDVSFR-VKYVLDDFDS-QKSVFYFHP-QYLINVQRFWMMQGVRA
Squaca_5       YNTRNTDVSFR-VKYVLDDFES-QQSMFYFHP-RYLENVSRFWMRQGLRA
Angang_5       YNTRNTDVSFR-VKYMLDDFDS-QKGVYFFHP-QYLLNVQRFWSLQGVRA
Acisin_5       YNTRNTDVSFR-VKYVLDDFDS-PQSVFYFHP-QYLLNVQRFWMMQGVMA
Orylat_5       YNTRNTDVSFR-VKYMLDDFDS-QRSVFFFHP-QYLLNVQRFWAVQGVRA
Astmex_5       YNMRNTDVSFR-VKYMLDDFDS-QRGVFFFHP-QYLLNVQRFWAVQGVRA
Angang_5b      YNTHNTEVSFR-VKYTMDDFGS-RRPVYFFNP-QYLLSVQRFWAIQGVRA
Anates_5       YNTRNTDVSFR-VKYMLDDFDS-QRGVFFFHP-QYLLNVQRFWAVQGVRA
Calmil_5       YNTRNTDVSLR-VRYALDDFSA-PQDLFYFHP-RYLENVARFWGRQGVRA
Hetzeb_5       YNTRNTDVSFR-VKYVLDDFES-QQSMFYFHP-RYLENVSRFWMRQGLRA
Eptbur_5       FGGRSMELALR-ARYVLDEAGS-SRPLYFFHP-IYLAHVGAFWRRHGVQE
Latcha_6       SFRSNTALSFK-VYHTLEAFRG-KQKTIFFHP-KYLKSLALFWRSKGIKV
Latmen_6       SFRSNTALSFK-VYHTLEAFRG-KQKTIFFHP-KYLKSLALFWRSKGIKV
Squaca_6       SYTKNTVLCFR-ALYTLQDFQA-KQKVVFFNP-VYLKHLTEFWLSKGIQV
Calmil_6       YHSKNTALSFR-AHYTLQDFKS-KQRVIFFNP-IYLKHLAHFWLSKGMQV
Hetzeb_6       SYSKNTDLCFR-ALYTLQDFQA-KQKVVFFNP-VYLKNLANFWLSKGIQV
Lepocu_8       SYGHNTPVSLR-AVYTLQDFNS-PVRAVFLNP-EYLQNLARFWKAHGLRT
Acisin_8       SYGRNTPLSLR-ALYTLQDFRS-PVRPIFLNP-RYLGSLARFWRARGLRS
Angang_8       SYAHNTPVSLR-AFYTLRDFGG-RARPAFLSP-AYLQSLAHFWRAQGLRT
Orylat_8       SFGHNTALSMR-AFYTLEDFES-SARAVYFNP-EYLKNLANFWRSEGLKS
Astmex_8       SYSHNTPVSLR-ALYALEDFNAVGPRPVFFNP-EYLSSLARFWRGRGLRT
Amical_8       SYSRNTPVSLR-ALYTLQDFDS-PVRPVFLNP-EYLHNLARFWKAQGLQA
Anates_8       SYGHNTPVSLR-AFYTIEDFKS-PTRPVFFNP-EYLQSLALFWRSQGLRA
Lepocu_8b      SYSRNTPVSLR-ALYTLQDFDS-PVRPVFLNP-EYLHNLARFWKAQGLQA
Lepocu_7       VFPHCTEPCVR-AFYSLLELRP-QQRALFFSP-QYLRQLALYWQRQGLHP
Lepocu_7b      AFSFCTDPCFR-VYYTLQDNRP-HQRALFYHP-DYLRQLATYWQEKGLRE
Angang_b       SYVVGTELSFR-VFHTMKDMRP-QQEVLYFHP-DYLFRLARYWQKQGLTG
Latcha_7       SQSFATEISLA-VAYALEDFGS-KQKAIFFHP-AYLRQLGSLWRSWGVRA
Angang_7       AYTTCTDISFK-VHKVVQKMRP-NQKVVFFNP-EYLLELFQYWKHRGLEE
Eptbur_7       AYVHNLLPALR-AHRALEAAGA-RQKAVFFHP-DYLRRLARLWAARGLRP
Petmar_7       AYVHNLLPALR-AHRALEAAGA-RQKAVFFHP-DYLRRLARLWAARGLRP
Letcam_7       AYVHNLLPALR-AHRALEAAGA-RQKAVFFHP-DYLRRLARLWAARGLHP
Petmar_7b      AYVHNLLPALR-AHRALEAAGA-RQKAVFFHP-DYLRRLARLWAARGLRP
Letcam_7r      HLTHTNLA-YKQAFYTMDDFAT-GQSILFLNP-NYSRGLRALWNHQGLAS
Petmar_7r      HLTHTNLA-YK-AFYTMDDFAT-GQSILFLNP-NYSRGLRALWNHQGLAS
Eptbur_7r      HLTHTNLA-YK-AFYTMDDFAT-GQSILFLNP-NYSRGLRALWNHQGLAS

Angang_3       KRLSTGILMYTLASAMCEEIHLYGFWPFGWDPN---TGK-----------
```

```
Lepocu_3       KRLSTGILMYTLASAMCEEIHLYGFWPFGWDPN---TGK-----------
Latcha_3       KRLSTGILMYTLASAMCQEIHLYGFWPFGWDPN---SGK-----------
Squaca_3       KRLSTGILMYTLASAICEEIHLYGFWPFAWDPN---TGK-----------
Astmex_3       KRLSTGILMFTLASSLCEQIHLYGFWPFGWDPN---TGK-----------
Orylat_3       KRLSTGILMYTLASSMCDQIHLYGFWPFGWDPN---TGK-----------
Hetzeb_3       KRLSTGILMYTLASAICEEIHLYGFWPFAWDPN---TGK-----------
Anates_3       KRLSTGILMYTLASSMCDQIHLYGFWPFGWDPN---TGK-----------
Acisin_3       KRLSTGILMYTLASSMCEEIHLYGFWPFGWDPN---TGK-----------
Masarm_3       KRLSTGILMYTLASSMCDQIHLYGFWPFGWDPN---TGK-----------
Calmil_3       KRLSTGILMYTLASSVCEEIHLYGFWPFAWDPN---TGR-----------
Orymel_9       KRLSTGILMYTLAFAMCDEIHLYGFWPFGWDPN---TGK-----------
Anates_9       KRLSTGILMYTLAYAMCDEIHLYGFWPFGWDPN---TGK-----------
Masarm_9       KRLSTGILMYTLASAMCDEIHLYGFWPFGWDPN---TGK-----------
Orylat_9       KRLSTGILMYTLAFSMCDEIHLYGFWPFGWDPN---TGK-----------
Eptbur_3/9     KRLSTGMLMYTIASAFCDEISLYGFWPFAWEPI---SGR-----------
Letcam_3/9     KRLSTGLLMASLCASLCDRTDLYGFWPFAWDPR---TGS-----------
Anates_2       KRPTTGLLMYTMATRFCEEIHLYGFWPFPLDPQ---G-K-----------
Latcha_2       KRPSTGLLMYTLATRFCSEIHLYGFWPFPRDHS---E-R-----------
Amical_2       KRPTTGMLMYTLATRFCEEIHLYGFWPFSRGPA---G-N-----------
Acisin_2       KRPTTGLLMYTLATRFCDEIHLYGFWPFPQDQD---G-N-----------
Angang_2       KRPTTGLLMYTMATRFCDEIHLYGFWPFPQDPQ---G-N-----------
Astmex_2       KRPTTGLLMYTMATRFCEEIHLYGFWPFPRDSE---G-K-----------
Orylat_2       KRPTTGLLMYTMATRFCEEIHLYGFWPFPVDSH---G-K-----------
Hetzeb_2       KRPTTGLLMYTLATRFCDEIHLYGFWPFPKDQK---G-N-----------
Lepocu_2       KRPTTGLLMYTLATRFCEEIHLYGFWPFSLGPN---G-L-----------
Latcha_4       --------------------------------------------------
Astmex_4-A     KRPSTGLLMYTMATRFCDEIHLYGFWPFPKDAN---G-I-----------
Angang_4       KRPSTGLLMYTLATRFCDEIHLYGFWPFPKDLG---G-N-----------
Lepocu_4       KRPSTGLLMYTLATRFCDEIHLYGFWPFPKDSQ---G-R-----------
Amical_4       KRPSTGLLMYTLATRFCDEIHLYGFWPFPKDTK---G-R-----------
Calmil_4       KRPSTGLLMYTLATRFCDEIHLYGFWPFPKDAN---G-T-----------
Squaca_4       KRPSTGLLMYTLATRFCDEIHLYGYWPFPKDAK---G-A-----------
Acisin_4       KRPSTGLLMYTMATRFCDEIHLYGFWPFPKDSH---G-K-----------
Hetzeb_4       KRPSTGLLMYTLATRFCDEIHLYGYWPFPKDAK---G-T-----------
Petmar_4       KRPTTGLLMYTLATRFCREIHLYGFWPFQYNIA---G-K-----------
Letcam_4       KRPTTGLLMYTLATRFCREIHLYGFWPFQYNIA---G-Q-----------
Eptbur_4       KRPTTGLLMYTLFTRFCRQIHLYGFWPFHSDTE---G-R-----------
Latcha_1       KRLSTGLFLVSAALGLCEEVTLYGFWPFSVDLH---G-V-----------
Angang_1       KRLSTGLFMASLALGLCEDVELYGFWPFSVDLE---E-R-----------
Angang_1b      RRLSTGLFLASLALGLCEEVELYGFWPFATDPR---G-R-----------
Astmex_1       KRLSTGLFLVSLALGLCEEVTAYGFWPFSVGLD---E-R-----------
Orylat_1       RRLSTGLFLVSLAMGLCDEVTTYGFWPFSVDLD---E-Q-----------
Acisin_1       KRLSTGMFLVSLALGLCDEVTLYGFWPFSLDLQ---E-R-----------
Lepocu_1       KRLSTGLFLVSLALGLCDEVTIYGFWPFSVDLN---E-R-----------
Hetzeb_1       KRLSTGLFLVSVALGLCEDVTLYGFWPFSMDLQ---G-T-----------
Squaca_1       KRLSTGLFLVSVALGLCEDVTLYGFWPFSMDLQ---G-R-----------
Calmil_1       KRLSTGLFLVSVALGLCEEVTLYGFWPFSTDLE---E-R-----------
Petmar_1       TRLSTGMFLVSAALGMCEDVHLYGFWPFSEDLS---G-R-----------
Letcam_1       MRLSTGMFLVSAALGMCEDVHLYGFWPFSEDLS---G-R-----------
Eptbur_1       GRPSTGLFLATAAIRLCRRLTVYGFWPLEIGPEFWKNGS-----------
Latcha_5       KRLSSGLMLVTAALELCHEVHLYGFWAFPMNPS---G-I-----------
Letcam_5       RRLSSGLMVASAALELCREVHAYGFWPFGVGAR---G-D-----------
Lepocu_5       KRLSSGLMLVTAALELCEEVHLYGFWAFPMNPS---G-I-----------
Squaca_5       KRLSSGLMLVTAAMELCEEVHLYGFWAFSMDPS---G-L-----------
Angang_5       KRLSSGLMLVTAAMELCEEVHLYGFWAFPMNPA---G-I-----------
Acisin_5       KRLSTGLMLVTAAMELCEEVHLYGFWGFPMDPN---G-I-----------
Orylat_5       KRLSSGLMLVTAALEICEEVHLYGFWAFPMNPS---G-V-----------
Astmex_5       KRLSSGLMLVTAAMELCEEVHLYGFWAFPMNPS---G-I-----------
Angang_5b      KRLTSGLMLATAAMELCEEVHLYGFWAFSMNPS---G-I-----------
Anates_5       KRLSSGLMLVTAALEMCEEVHLYGFWAFPMNPS---G-I-----------
Calmil_5       KRPSSGLMLVTAAMELCQEVHLYGFWGFPMDPS---G-I-----------
Hetzeb_5       KRLSSGLMLVTAAMELCEEVHLYGFWAFSMDPS---G-L-----------
Eptbur_5       RRSSSGLLVASAAALELCREVHLYGFWPFSQDVD---G-A-----------
Latcha_6       YRLSSGFMIANAAIELCKEVRLYGFWPFSKNNE---G-K-----------
Latmen_6       YRLSSGFMIANAARRLCKEVRLYGFWPFSKNNE---G-K-----------
Squaca_6       NRLSTGIMVVSVAIELCEEVWIYGFWPFGRNVE---G-R-----------
Calmil_6       QNLSTGITVASMAMELCSEVWIYGFWPFGKNTE---G-E-----------
Hetzeb_6       KRLSTGIMIVSVAVELCEEVWIYGFWPFGKNVE---G-K-----------
Lepocu_8       VRLSTGLIVASLALELCASVTLYGFWPFSLHPF---SKQ-----------
Acisin_8       SRLSTGLMMASLALELCSSVRLYGFWPFDHGPN---G-S-----------
Angang_8       VRLSTGLIVASLALELCANVHLYGFWPHAQHPH---DRR-----------
Orylat_8       PRLSTGIMMASIALEVCSEVHLYGFWPFDVHPY---SHQ-----------
Astmex_8       ARLSTGLIVTSLALELCTNVHLYGFWPFSLHPH---GRQ-----------
Amical_8       IRLSTGLIMASLALELCANVQLYGFWPFSKDPH---SKH-----------
Anates_8       VRLSSGLIMASLALELCTDVHLYGFWPFSNHPH---DLH-----------
Lepocu_8b      IRLSTGLIMASLALELCANVQLYGFWPFSKDPH---SKH-----------
Lepocu_7       VRLSTGMMLASVALELCDHVHLYGFWPFPFDLS---R-R-----------
```

```
Lepocu_7b      TRLSTGLMMVSAALELCDRVELYGFWPFSFDLS---E-R-----------
Angang_b       LRLSSGFMLVSVALELCEKVDIYGFWPFPTDLI---Q-K-----------
Latcha_7       RRLSSGLMLVSAALELCDSVALYGFWPFSTGLD---G-E-----------
Angang_7       LRLTTGLMLASVAMELCDSVHLYGFWPFELDLF---Q-C-----------
Eptbur_7       QRLSTGLMLASASLTLCRQVHLYGFWPLERGPD---G-A-----------
Petmar_7       QRLSTGLMLASASLTLCRQVHLYGFWPLERGPD---G-A-----------
Letcam_7       QRLSTGLMLASASLTLCSQVHLYGFWPFERGPD---G-A-----------
Petmar_7b      QRLSTGLMLASASLTLCRQVHLYGFWPLERGPD---G-A-----------
Letcam_7r      KYLSAGLHMTSAALEACEEVHLFGFWPMEIASVSRRGRRGRRGRRSRTSI
Petmar_7r      KYLSAGLHMTSAALEACEEVHLFGFWPMEIASVSRRGRRGRRSR---TSI
Eptbur_7r      KYLSAGLHMTSAALEACEEVHLFGFWPMEM--------------------

Angang_3       -ELPYHYYDKKGTKFTTKWQESHQLPSEFKLLYKMHTDG-------VIKLSLSHCA--
Lepocu_3       -ELPYHYYDKKGTKFTTKWQESHQLPTEFKLLYKMHTEG-------LTKLSLSHCA--
Latcha_3       -DLPYHYYDKKGTKFTTKWQESHQLPAEFKLLYKMHGEG-------LTKLTLSHCA--
Squaca_3       -ELPYHYYDKKGTKFTTKWQESHQLPAEFKLLFKLHTDG-------LTKLTLSHCAS-
Astmex_3       -ELPYHYYDKKGTKFTTKWQESHQLPTEFKLLFKMHTEG-------VLKLSLSHCA--
Orylat_3       -ELPYHYYDRKGTKFTTKWQESHQLPAEFKLLHKMHTEG-------LLKLTLSHCA--
Hetzeb_3       -ELPYHYYDKKGTKFTTKWQESHQLPAEFKLLFKLHTDG-------LTKLTLSHCAS-
Anates_3       -ELPYHYYDKKGTKFTTKWQESHQLPAEFKLLYKMHMEG-------LLKLTLSHCA--
Acisin_3       -DLPYHYYDKKGTKFTTKWQESHQLPTEFKLLYKMHGEG-------LTKLTLSHCA--
Masarm_3       -ELPYHYYDKKGTKFTTKWQESHQLPAEFKLLYKMHMEG-------LLKLTLSHCA--
Calmil_3       -ELPYHYYDKKGTKFNTNWQETHQLPAEFKILFKMHTAG-------LTKLSLSPCVS-
Orymel_9       -ELPYHYYDKKGTKFTTKWQETHQLPSEFKLLYKLHREG-------VTKLGLSHCT--
Anates_9       -DLPYHYYDKKGTKFTTKWQETHQLPSEFKLLYKLHREG-------VTKLSLTHCT--
Masarm_9       -ELPYHYYDKKGTKFTTKWQETHQLPSEFKLLYKLHREG-------VIKLSLTHCIA-
Orylat_9       -DLPYHYYDKKGTKFTTKWQETHQLPSEFKLLYKLHREG-------VTKLGLSHCT--
Eptbur_3/9     -PLPYHYYARRGARFTTAWQEAHQLPAEFRLFQQLHGQG-------ALRLHLGSCT--
Letcam_3/9     -PIPYHYYDAPGAKFSTRWQESHQLPLEFQLLRSLHAHG-------ALRLRLEACA--
Anates_2       -PVKYHYYDALKYEYTSS-SSPHTMPLEFRTLSALHRQG-------ALRLHTGQCD--
Latcha_2       -PVKYHYYDSLTYEYTSQ-ASPHTMPLEFRTLYGLHQQG-------ALKLTVGECA--
Amical_2       -PVKYHYYDSLTYEYTAQ-SSPHTMPLEFKTLKNLHSQG-------ALQLHTGPCN--
Acisin_2       -PVKYHYYDSLTYEYTSQ-SSPHTMPL-------------------------------
Angang_2       -PMKYHYYDTLTYEYDSH-SSPHTMPLEFSTLRDLHSQG-------ALRLHIGRCGAD
Astmex_2       -PVKYHYYDTLKYQYTSS-SSPHTMPLEFRTLSTLHRQG-------ALRLHTETCR--
Orylat_2       -AVKYHYYDSLKYEYTSS-SSPHTMPLEFRTLSTLHRQG-------ALRLHTGSCE--
Hetzeb_2       -VVKYHYYDSLTYEYNSR-ATPHTMPLEFKTLRNMHLQG-------ALKLNVGECNLT
Lepocu_2       -PVKYHYYDSLTYEYRAQ-SSPHSMPVEFRALKRLHSQG-------ALKLHTGECN--
Latcha_4       ----------------------------------------------------------
Astmex_4-A     -PVKYHYYDLLKYRYFSS-AGPHRMPLEFKTLKTLHSKG-------ALKLTTSKCR--
Angang_4       -PVKYHYYDALKYRYFSK-ASPHRMPLEFKTLKTLHSRG-------ALKLTTSKCARP
Lepocu_4       -PVKYHYYDELKYRYFSN-ASPHRMPLEFKTLKMLHNKG-------ALKLTTTKCT--
Amical_4       -LVKYHYYDELKYRYFSN-ASPHRMPLEFKTLKTLHNKG-------ALKLTTSTCA--
Calmil_4       -PVKYHYYDNLKYRYFSN-AGPHRMPLEFKTLKSLHDRG-------ALKLTTGKCGQP
Squaca_4       -PVKYHYYDNLKYRYFSN-AGPHRMPLEFKTLKSLHDKG-------ALKLTTGKCV--
Acisin_4       -SVKYHYYDELKYRYFSN-ASPHRMPLEFKTLKMLHNKG-------ALKLTTSKCA--
Hetzeb_4       -PVKYHYYDNLKYRYFSN-VGPHRMPLEFKTLKSLHDRG-------ALKLTTGKCV--
Petmar_4       -PVRYHYYEGLRYRYRSR-AGPHTMPLEYRTLHRLHSLG-------ALKLTTGKCT--
Letcam_4       -PVRYHYYEGLRYRYRSR-AGPHAMPLEYRTLHRLHSLG-------ALKLTTGKCT--
Eptbur_4       -PVPYHYYEVMSYSYYSR-SSPHAMPLEFQALHRLHELG-------ALRMRTQACT--
Latcha_1       -SISHHYYDNVLPY-----TGFHAMPEEFLQLWLLHKIG-------ILRMQIGQCK--
Angang_1       -PISHHYYDNVLPY-----SGFHAMPEEFLQLWHLHKTG-------TLRMHIGQCPDA
Angang_1b      -PVGHHYYNDVPPL-----WGVHAMPEEFLRLWELHKGG-------ALRMRVDSCPRE
Astmex_1       -PVSHHYYDNILPS-----SGFHAMPEEFLQLWHLHKSG-------TLRLRVGSCV--
Orylat_1       -PISHHYYDNILPY-----KWFHAMPEEFVQLWHLHKSG-------ILRMKVGHCS--
Acisin_1       -FISHHYYDNVMPF-----SGFHAMPEEFLQLWHLHQSG-------TLRMQVGRCD--
Lepocu_1       -FISHHYYDNILPF-----SGFHAMPEEFLQLWVLHKTG-------TLRMRVGQCE--
Hetzeb_1       -IISHHYYDNVMPY-----SGYHAMPEEFLQLWLLHKSG-------ILKMQIGKCEEN
Squaca_1       -IISHHYYDNVMPY-----SGYHAMPEEFLQLWLLHKSG-------ILKMQIGKCEEN
Calmil_1       -TISHHYYDNVMPD-----TSYHAMPEEFLQLWLLHKSG-------ILKIHVGKCGQI
Petmar_1       -AISHHYYDDVPPL-----WYYHAMPEEFEQLWMLHQRG-------ILRMHLEQCD--
Letcam_1       -AISHHYYDDVPPL-----WYYHAMPEEFEQLWMLHQRG-------ILRMHLEQC---
Eptbur_1       -HLQHHYYDSHLPL-----PVYHSMAQEFSLLWELHEQGLQDRREKVHKRYRSLCRL-
Latcha_5       -FITHHYYDNVKPR-----PGFHAMPSEIFKFLHMHSKG-------VLRVHTGTCS--
Letcam_5       -SLAHHYYDDAKPR-----PGFHAMPAETVHLMHLHARG-------TLHLHTEPCP--
Lepocu_5       -FITHHYYDNVKPR-----PGFHSMPNEIFSFLHLHSRG-------ILQVHTSPCR--
Squaca_5       -YITHHYYDNVKPR-----PGFHKMTTEIFNFLHMHSKG-------ILQVHTGTCS--
Angang_5       -FITHHYYDNVKPR-----PGFHAMPHEIFNFLHLHSRG-------ILHVHTNPCR--
Acisin_5       -FMTHHYYDNVKPR-----PGFHAMPHEIFNFLHLHSRG-------ILQVHTRACS--
Orylat_5       -YITHHYYDNVKPR-----PGFHAMPHEIFNFIHMHTRG-------IINVHTDQCM--
Astmex_5       -FITHHYYDNVKPR-----PGFHAMPHEIFNFLHMHARG-------IVHVHTGVCR--
Angang_5b      -FIMHHYYDNIKPR-----PGFHAMPSEIFSFLHLHSRG-------ILHVHTNHCR--
Anates_5       -FITHHYYDNVKPR-----PGFHAMPHEIFNFIHMHSRG-------IINVHTGQCT--
Calmil_5       -HITHHYYDNVKPR-----PGFHSMPNEIFTFLHMHSRG-------VLQVHTTPCR--
Hetzeb_5       -YITHHYYDNVKPR-----PGFHKMTTEIFNFLHMHSRG-------ILQVHTGICS--
Eptbur_5       -HLSHHYYDDQRPR-----PGIHVMPIENFHFLQLHSKG-------ILRVHTQRCS--
Latcha_6       -NISHHYYDNQLPK-----PGIHSMPKEFYHFLKLHNKG-------IIKLQFGRCD--
```

```
Latmen_6        -NISHHYYDNQLPK-----PGIHSMPKEFYHFLKLHNKG-------IIKLQFGRCD--
Squaca_6        -VMSHHYFDNLLPK-----PDVHSMPSEFHQLLQMHSKG-------IIKLQTAQCQAD
Calmil_6        -LMSHHYFDSLLAE-----PDLHSTSNEFYQLLRMHSKG-------IVKLQMGQCEAE
Hetzeb_6        -VVSHHYFDNLLPK-----PDTHSMPLEFHHLLQMHSKG-------MIKLQMTQCQAD
Lepocu_8        -HLTNHYYDDQQSK-----KSFHAMPAEFEQLLRLHSQG-------VIRVHLGSC---
Acisin_8        -PLLHHYYDDRRSP-----SSIHAMPAEFSQLLGLHRQG-------VLRIQLGRCQ--
Angang_8        -PLTNHYYDDRQGK-----KKVHAMPAEFGHLLRLHRQG-------VVRVHLGQCEDR
Orylat_8        -GLTNHYYDDRKAK-----NKFHAMPTEFNLLLQLHRKG-------VLRLHLGDCT--
Astmex_8        -TITNHYYDNRQSK-----KNVHSMPSEFEHLLRLHVSG-------VIQLHLGECS--
Amical_8        -PLTNHYYDDQQAK-----KTIHAMSTEFSHLLMLHNQG-------IIRVQLGKCQ--
Anates_8        -VLTNHYYDDRKTK-----TKFHAMPAEFELLLRLHSQG-------VLRLHLGECH--
Lepocu_8b       -PLTNHYYDDQQAK-----KTIHAMSTEFSHLLMLHNQG-------IIRVQLGKCQ--
Lepocu_7        -PLSHHYFDDVGPK-----PGAHMMAEEFLKLLQLHSQG-------ALQLHLGPCP--
Lepocu_7b       -PLSNHYYDDVPPN-----RGMHAMPEEFLRLLRMHSQG-------VLRLRVGQCP--
Angang_b        -PVTHHYYDNVVAK-----QGVHAMPEEFLTLLHMHSQG-------VLQLHVGKCQ--
Latcha_7        -AVLHHYYDNVPPE-----PGVHAMSSEFVHYLQMHSKG-------VLQLHVGKC---
Angang_7        -PVTHHYYDNVGPS-----RRMHAMPKEFLQLLKMHCQG-------SIHLQLTRCH--
Eptbur_7        -SLPHHYFDDRPPR-----PGAHDMPQEFSHYLLLHSQG-------AIRLHMGACT--
Petmar_7        -SLPHHYFDDRPPR-----PGAHDMPQEFSHYLLLHSQG-------AIRLHMGACT--
Letcam_7        -SLPHHYFDDRPPR-----PGAHDMPQEFSHYLLLHSQG-------AIRLHMGACT--
Petmar_7b       -SLPHHYFDDRPPR-----PGAHDMPQEFSHYLLLHSQG-------AIRLHMGACT--
Letcam_7r       VTVRHSYFEQSDQE--SKGKEVFDLKMEFAHLLRLHTNG-------IIRLQLGACAV-
Petmar_7r       VTVQQSYFEQSSQE--SKVKEVFDLKMEFAHLLRLHTNG-------IIRLQLGACAV-
Eptbur_7r       -TVQQSYFEQSSQE--SKVKEVFDLKMEFAHLLRLHTNG-------IIRLQLGACAV-
```

C) Multiple sequence alignments (ClustalX) of ST8Sia sequences used in figure 5: Substitution rate analysis of the impact of *st8sia* gene duplications and losses

Figure 5A

```
CLUSTAL X (1.81) multiple sequence alignment
             1
Cluhar2      MSFEFRALLFGIVTMLVIFLIIADISVVE-EEIANIGESQKYYLHSSAPK
Denclu_2     MPFEFRALMFGIVTVLVAFLILADISEVE-EEIANTGIYQKFHSQNFIPK
Danrer_2     MSFEFRILMFGIGTALVIFVIIADISEVE-EEIANIEDSRKFHLKSVALQ
Hipcom_2     MTPTSRTLLFLFVTLLVILLIADDISQVENEEEQEALKSSQSTMKRLFPK
Cormar_2A    MQLEFRTVMFGIVTLLVIFLIIADIAEIE-EEIANIGGSRTLYLHSLIPK
Oncmyk_2A    MQLELRTLMFGIVTLLVIFLIIADIAEVQ-EEIGNIGGSRTLYLHSLIPK
Onctsh_2A    MQLEFRTLMFGIVTLLVIFLIIADIAEVQ-EEIGNIGGSRTLYLHSLIPK
Thythy_2A    MQLEFRTVMFAVVTLLVIFMIIADIAEVE-EEIANIGGSRTFYFLNLNLK
Salsal_2A    MHLEFRTLMFGIVTLLVVFLIIADIAEVQ-EEIGNIGGSRTLYLHSLIPK
Salalp_2A    MQLEFRTLIFGIVTLLVIFLIIADIAEVQ-EEIG----------------
Oncmyk_2B    MQLELRTLMFGIVTVLVLFLIIADIAEVE-KEIANLGGSRQLYLQTLIPK
Onckis_2B    MQLELRTLMFGIVTVLVLFLIIADIAEVE-KEIANLGGSRQLYLHSLIPK
Salalp_2B    MQLELRTLMFGIVTVLVLFLIIADIAEVE-KEIANLGGSRELYLHSLIPK
Salsal_2B    MQLELRTLMFGIVTVLVLFLIIADIAEVE-KEIANFGGSRQLCLHSLIPK
Thythy_2B    MQLEFRTLMFGIVTVLVIFLIIADIAEVE-EEVANVGGSRK--LHSLIPK
Onctsh_2B    MQLELRTLMFGIVTVLVLFLIIADIAEVE-KEIA----------------
Cormar_2B    -------------SLLILLKSKKKLR------------------------
Corclu_4     MRLSRKRWTICTISILVIFYKTKEITRSE---------------------
Corlav_4     MRLSRKRWTICTISILVIFYKTKEITRSE---------------------
Onckis_4     MRLSRKRWTICTISILVIFYKTKEITRSE---------------------
Oncmyk_4     MRLSRKRWTICTISILVIFYKTKEITRSE---------------------
Salsal_4     MRLSRKRWTICTISILVIFYKTKEITRSE---------------------
Salalp_4     MRLSRKRWTICTISILVIFYKTKEITRSE---------------------
Salfon_4     MRLSRKRWTICTISILVIFYKTKEITRSE---------------------
Thythy_4     MRLSRKRWTICTISILVIFYKTKEITRSE---------------------

             51
Cluhar2      LNRSTVLVPSSAPVVKS-KGDNSSLAQ---FARNGTSASPAEWTFNRTLS
Denclu_2     SNRNMPLKTSPSLDLSSKDLGSKAIKNCSSSHASKNKSVVSDWSFNRTLS
Danrer_2     SNRSSDLNAAPTSLVTYRKSKVSSLASPSDIKRKTSNSSSSEWTFNRTLS
Hipcom_2     PRRKTSRRVGSTSLPRP--AAINHLEPG---DNSSGKLASSGWTFNKTLS
Cormar_2A    PNRNVAVKANPTPLISE--GEDKSPASP-SGLNNTTRLSSDNWTFNRTLS
Oncmyk_2A    PNRNVAVKANPTPLGNE--GEEKSPASP-SGLKNATRLSSDNWTFNRTLS
Onctsh_2A    PNRNVAVKANPTPLGNE--GEEKSPASP-SGLKNATRLSSDNWTFNRTLS
Thythy_2A    PNRNTAVKANPTSLVSE--GEDKSPPS--SDSNNTTRLSSDNWTFNRTLS
Salsal_2A    PNRNVAVKANPTPLVNE--GEDKSPASP-SGSKNSTRLSSDNWTFNRTLS
Salalp_2A    ---NVAVKANPTPLVNE--GEDKSPASP-SGSKNATRLSSDNWTFNRTLS
Oncmyk_2B    PNRNVAVKANPKPLVSE--GEDKSPASP-SYSNNTTKLSTDNWMFNRTLS
Onckis_2B    PNRNVAVKANPKPLVSE--GEDKSPASP-SYSNNTIKLSTDNWMFNRTLS
Salalp_2B    PNRNVAVKVNPKPLVSE--GEDKRPASP-SYLNNTTKLSTDNWTFNRTLS
Salsal_2B    PNRNVAVKANPKPLVSE--GEDKSPAST-SYSNNTTKLSTDNWTFNRTLS
Thythy_2B    PNRNVAVKANPKPLVSE--GEDKSPASP-SGSNNTTKLSSDNWTFNRTLS
Onctsh_2B    ---NVAVKANPKPLVSE--GEDKSPDSP-SYSNNTTKLSTDNWMFNRTLS
Cormar_2B    ---NVAVKANPKPLVSE--GEDKSPASP-SYSNNTTKLSSDNWTFNRSLS
Corclu_4     -EHQEAQVTGDSELDTSRLMVNSSEKSSRSG-PSFFQHSVEGWRLNSSLV
Corlav_4     -EHQEAQVTGDSELDTSRLMVNSSEKSSRSG-PSFFQHSVEGWRLNSSLV
Onckis_4     -EHQEAQVTG--ELGTSRLMVNSSEKSSRSGVPSFFQHSVEGWRVNSSLV
Oncmyk_4     -EHQEAQVTG--ELGTSRLMVNSSEKSSRSGVSSFLQHSVEGWRVNSSLV
Salsal_4     -EHQEAQVTGDSELGTSRLMVNSSEKSSRSGVPSFFQHSVEGWRVNSSLV
Salalp_4     -EHQEAQVTGDSELGTSRLMVNSSEKSSRSGVPSFFQHSVEGWRVNSSLV
Salfon_4     -EHQEAQVTGDSELGTSRLMVNSSEKSSRSGVPSFFQHSVEGWRVNSSLV
Thythy_4     -EHQEAQVTGDSELGTSRLMVNSSDKSSRSGVPSFFQHSVKGWRVNSSLV
             101
Cluhar2      NMIRKNILKFLDPERDISILKGTLKPGDIIHYVFDRQSTMNVSENLYRLL
Denclu_2     SLIRKNILRFLDAERDISILKGTLKPGDVVHYIFDRQSTMNISENLYRLL
Danrer_2     NLIRKNILKFLDAERDISILKSTFKPGDVIHYIFDRQSTTNISENRYHLL
Hipcom_2     KLIRKNVLRFLDPERDISILKGSLRPGDVVHYVFDRHSTTNISENLHRLL
Cormar_2A    SLIRKNILRFFDPERDISILKGTLKPGDVIHYIFDRQSTTNISENLYRLL
Oncmyk_2A    SLIRKNILGFFDPERDISILKGTLKPGDVIHYVFDRQSTTNISENLYQLL
Onctsh_2A    SLIRKNILGFFDPERDISILKGTLKPGDVIHYVFDRQSTTNISENLYRLL
Thythy_2A    SLIRKNILRFFDPERDISILKGTLKPGDVIHYIFDRQSTTNISENLYRLL
Salsal_2A    SLIRKNILGFFDPERDISILKGTLKPGDVIHYIFDRQSTTNISENLYRLL
Salalp_2A    SLIRKNILRFFDPERDISILKGTLKPGDVIHYVFDRQSTTNISENLYRLL
Oncmyk_2B    NLIRKNILRFFDPERDISILKGTLKPGDVIHYIFDRQSTTNISENLYQLL
```

```
Onckis_2B     NLIRKNILRFFDPERDISILKGTLKPGDVIHYIFDRQSTTNISENLYQLL
Salalp_2B     NLIRKNILRFFDPERDISILKGTLKPGDIIHYIFDRQSTTNISENLYQLL
Salsal_2B     NLIRKNILRFFDPERDISILKGTLKPGDVIHYIFDRQSTTNISENLYQLL
Thythy_2B     NLIRKNILRFFDPERDISILKGTLKPGDVIHYIFDRQSTTNISENLYRLL
Onctsh_2B     NLIRKNILRFFDPERDISILKGTLKPGDVIHYIFDRQSTTNISENLYQLL
Cormar_2B     NFYRKNILRFFDPERDISILKGTLKPGDVIHYIFDRQSTTNISENLYRLL
Corclu_4      LMIRKDVLRFLDAERDVSVVKSSFKPGDTIHYVLDRRRTLNISHTLHSLL
Corlav_4      LMIRKDVLRFLDAERDVSVVKSSFKPGDTIHYVLDRRRTLNISHTLHSLL
Onckis_4      LMIRKDVLRFLDAERDVSVVKSSFKPGDTIHYVLDRRRTLNVSHTLHSLL
Oncmyk_4      LMIRKDVLRFLDAERDVSVVKSSFKPGDTIHYVLDRRRTLNVSHTLHSLL
Salsal_4      LMIRKDVLRFLDAERDVSVVKSSFKPGDTIHYVLDRRRTLNVSHTLHSLL
Salalp_4      LMIRKDVLRFLDAERDVSVVKSSFKPGDTIHYVLDRRRTLNVSHTLHSLL
Salfon_4      LMIRKDVLRFLDAERDVSVVKSSFKPGDTIHYVLDRRRTLNVSHTLHSLL
Thythy_4      LMIRKDVLRFLDAERDVSVVKSSFKPGDTIHYVLDRRRTLNVSHTLHSLL
              151
Cluhar2       PTASPMKNQHHKSCAIVGNSGILLNSSCGPEIDAHDFVIRCNLAPVNEFS
Denclu_2      PTASPMKNQHHRLCAIVGNSGILLNSSCGPEIDSHDFVIRCNLAPVEEYA
Danrer_2      PTVSPMKNQHYRKCAIVGNSGILLNSSCGREIDSHDFVIRCNLAPVEEYA
Hipcom_2      PSVSPLKNRHYRLCAIVGNSGILLNSSCGSQIDSHDFVIRCNLAPVEEYA
Cormar_2A     PTASPMKNQHHRRCAIVGNSGILLNSSCGPEIDSHDFVIRCNLAPVEEYA
Oncmyk_2A     PTASPMKNQHHRSCAIVGNSGIQLNSSCGPEIDSHDFVIRCNLAPVEEYA
Onctsh_2A     PTASPMKNQHHRSCAIVGNSGILLNSSCGPEIDSHDFVIRCNLAPVEEYA
Thythy_2A     PTASPMKNQHHRRCAIVGNSGILLNSSCGPEIDSHDFVIRCNLAPVEEYA
Salsal_2A     PTASPMKNQHHRRCAIVGNSGILLNSSCGPEIDSHDFVIRCNLAPVEEYA
Salalp_2A     PTASPMKNQHHRRCAIVGNSGILLNSSCGPEIDSHDFVIRCNLAPVEEYA
Oncmyk_2B     PTASPMKNQHHRHCAIIGNSGILLNSSCGPEIDSYDFVIRCNLAPVEEYA
Onckis_2B     PTASPMKNQHHRHCAIIGNSGILLNSSCGPEIDSYDFVIRCNLAPVEEYA
Salalp_2B     PTASPMKNQHHRRCAIIGNSGILLNSSCGPEIDSYDFVIRCNLAPVEEYA
Salsal_2B     PTVSPMKNQYHRRCGIIGNSGILLNSSCGPEIDSYDFVIRCNLAPVEEYA
Thythy_2B     PTTSPMKNQYHRRCAIVGNSGVLLNSSCGPEIDSHDFVIRCNLAPVEEYA
Onctsh_2B     PTASPMKNQHHRHCAIIGNSGILLNSSCGPEIDSYDFVIRCNLAPVEEYA
Cormar_2B     PTVSPMKNQHHRRCAIVGNSGILLNSSCGPEIDSYDFVIRGNQAPVEEYA
Corclu_4      PDVSPLKNKRFKTCAVVGNSGVLLNSGCGKEIDRHDFVIRCNLAPLAEFA
Corlav_4      PDVSPLKNKRFKTCAVVGNSGVLLNSGCGKEIDRHDFVIRCNLAPLAEFA
Onckis_4      PDVSPLKNKRFRTCAVVGNSGVLLNSGCGKEIDSHDFVIRCNLPPLSEFA
Oncmyk_4      PDVSPLKNKRFRTCAVVGNSGVLLNSGCGKEIDSHDFVIRCNLPPLSEFA
Salsal_4      PDVSPLKNKRFRTCAVVGNSGVLLNSGCGKEIDSHDFVIRCNLPPLSEFA
Salalp_4      PDVSPLKNKRFRTCAVVGNSGVLLNSGCGKEIDSHDFVIRCNLPPLSEFA
Salfon_4      PDVSPLKNKRFRTCAVVGNSGVLLNSGCGKEIDSHDFVIRCNLPPLSEFA
Thythy_4      PDVSPLKNKRFKTCAVVGNSGVLLHSGCGKEIDSHDFVIRCNLAPLADFA
              201
Cluhar2       ADVGHRTNLVTMNPSVVQRAFQDLSSEEWRQRFVRRLQDLSGSVLWIPAF
Denclu_2      ADVGHRTDLVTMNPSVVQRAFQDLANEEWRERFVQRLRALSGSVLWIPAF
Danrer_2      ADVGLRTSLVTMNPSVVQRAFQDLNSEEWVQRFVQRLQSLSGSVLWIPAF
Hipcom_2      DDVGHRTDLVTMNPSVVQRAFRDLVDDEWRRRFARRLASLSGSVLWIPAF
Cormar_2A     GDVGRRTNLVTMNPSVVQRAFHDLASEQWRERFLQRLRGLSGSVLWIPAF
Oncmyk_2A     GDVGRRTNLVTMNPSVVQRAFHDLASEQWRERFLQRLRGLSGSVLWIPAF
Onctsh_2A     GDVGRRTNLVTMNPSVVQRAFHDLASEQWRERFLQRLRGLSGSVLWIPAF
Thythy_2A     GDVGRRTNLVTMNPSVVQRAFHDLASEQWRERFLQRLRGLSGSVLWIPAF
Salsal_2A     GDVGQRTNLVTMNPSVVQRAFHDLASEQWRERFLQRLRGLSGSVLWIPAF
Salalp_2A     GDVGRRTNLVTMNPSVVQRAFHDLASEQWRERFLQRLRGLSGSVLWIPAF
Oncmyk_2B     GDVGRRTNLVTMNPSVVQRAFQDLASEEWRERFLQRLRSLSGSVLWIPAF
Onckis_2B     GDVGRRTNLVTMNPSVVQRAFQDLASEEWRERFLQRLQSLSGSVLWIPAF
Salalp_2B     GDVGQRTNLVTMNPSVVQRAFQDLASEEWRERFLQRLRSLSGSVLWIPAF
Salsal_2B     GDVGRRTNLVTMNPSVVQRAFQDLASEEWRERFLQRLRGLSGSVLWIPAF
Thythy_2B     GDVGRHTNLVTMNPSVVQRAFQDLASEEWRERFLQRLRGLSGSVLWIPAF
Onctsh_2B     GDVGRRTNLVTMNPSVVQRAFQDLASEEWRERFLQRLRSLSGSVLWIPAF
Cormar_2B     GDVGRRTNLVTMNPSVVQRAFQDLASEEWRERFLQRLRGLSGSVLWIPAF
Corclu_4      EDVGLRSDFTTMNPSVIQRVYGSLKNATDTERFVQRLRMLNDSVLWIPAF
Corlav_4      EDVGLRSDFTTMNPSVIQRVYGGLKNATDTERFVQRLRMLNDSVLWIPAF
Onckis_4      EDVGLRSDFTTMNPSVIQRAYGGLKNATDTERFVQRLRGLNDSVLWIPAF
Oncmyk_4      EDVGLRSDFTTMNPSVIQRAYGGLKNATDTERFVQRLRGLNDSVLWIPAF
Salsal_4      EDVGLRSDFTTMNPSVIQRAYGGLKNATDTERFVQRLRGLNDSVLWIPAF
Salalp_4      EDVGLRSDFTTMNPSVIQRVYGGLKNATDTERFVERLRGLNDSVLWIPAF
Salfon_4      EDVGLRSDFTTMNPSVIQRAYGGLKNATDTERFVERLRGLNDSVLWIPAF
Thythy_4      EDVGLRSDFTTMNPSVIQRAYGGLKNATDTERFVHRLQVLNDSVLWIPAF
              251
Cluhar2       MAKGGEERVEWAIRLIIMHTVNVRTAFPSLRLLHAVRGYWLTNQVQIKRP
Denclu_2      MAKGGEERVEWAIRLILLHTVNVRTAFPSLRLLHAVRGYWLTNQVLIKRP
Danrer_2      MAKGGEERVEWAIRLILLHTVNVRTAFPSLRLLHAVRGYWLTNHVQIKRP
Hipcom_2      MAKGGEERVEWALRLILRHAADVRTAFPSLRLLHAVRGYWLTNKVHIKRP
Cormar_2A     MAKGGEERVEWAIRLILLHTVDVHTAFPSLRLLHAVRGYWLTNNVQIKRP
Oncmyk_2A     MAKGGEERVEWAIRLILLHTVDVHTAFPSLRLLHAVRGYWLTNNVQIKRP
Onctsh_2A     MAKGGEERVEWAIRLILLHTVDVHTAFPSLRLLHAVRGYWLTNNVQIKRP
Thythy_2A     MAKGGEERVEWAIRLILLHTVDVHTAFPSLRLLHAVRGYWLTNNVQIKRP
Salsal_2A     MAKGGEERVEWAIRLILLHTVDVHTAFPSLRLLHAVRGYWLTNNVQIKRP
Salalp_2A     MAKGGEERVEWAIRLILLHTVDVHTAFPSLRLLHAVRGYWLTNNVQIKRP
```

```
Oncmyk_2B     MAKGGEERVEWAIRLILLHTVDVHTAFPSLRLLHAVRGYWLTNNVQIKRP
Onckis_2B     MAKGGEERVEWAIRLILLHTVDVHTAFPSLRLLHAVRGYWLTNNVQIKRP
Salalp_2B     MAKGGEERVEWAIRLILLHTVDVHTAFPSLRLLHAVRGYWLTNNIQIKRP
Salsal_2B     MAKGGEERVEWAIRLILLHTVDVHTAFPSLRLLHAVRGYWLTNNVQIKRP
Thythy_2B     MAKGGEERVEWAIRLILLHTVDVHTAFPSLRLLHAVRGYWLTNNVQIKRP
Onctsh_2B     MAKGGEERVEWAIRLILLHTVDVHTAFPSLRLLHAVRGYWLTNNVQIKRP
Cormar_2B     MAKGGEERVEWAIRLILLHTVDVHTAFPSLRLLHAVRRYWLTNNVQIKRP
Corclu_4      MVKGGERHVESVNELIVKRKLRVRTAYPSLRLIHAVRGYWLTNKINIKRP
Corlav_4      MVKGGERHVESVNELIVKRKLRVRTAYPSLRLIHAVRGYWLTNKINIKRP
Onckis_4      MVKGGERHVESVNELIVKRKLRVRTAYPSLRLIHVVRGYWLTNKINIKRP
Oncmyk_4      MVKGGERHVESVNELIVKRKLRVRTAYPSLRLIHVVRGYWLTNKINIKRP
Salsal_4      MVKGGERHVESVNELIVKRKLRVRTAYPSLRLIHVVRGYWLTNKINIKRP
Salalp_4      MVKGGERHVESVNELIVKRKLRVRTAYPSLRLIHVVRGYWLTNKINIKRP
Salfon_4      MVKGGERHVESVNELIVKRKLRVRTAYPSLRLIHVVRGYWLTNKINIKRP
Thythy_4      MVKGGERHVESVNELIVKRKLSVRTAYPSLRLIHAVRGYWLTNKINIKRP
              301
Cluhar2       TTGLLMYTMATRFCDEIHLYGFWPFHRDHQGNVVKYHYYDTLTYEFTSRA
Denclu_2      TTGLLMYTMATRFCEEIHLYGFWPFHRDAQGKPVKYHYYDALTYEYTSHS
Danrer_2      TTGLLMYTMATRFCDEIHLYGFWPFAHDPDGKPVKYHYYDTLTYHYTSSA
Hipcom_2      TTGLLMYTMATRFCEEIHLYGFWPFSHDAAGKAVKYHYYDTLKYEYTSSS
Cormar_2A     TTGLLMYTMATRFCEEIHLYGFWPFPRDSQGIPVKYHYYDTLTYEYTSHA
Oncmyk_2A     TTGLLMYTMATRFCEEIHLYGFWPFPQDSQGKPVKYHYYDTLTYEYTSHA
Onctsh_2A     TTGLLMYTMATRFCEEIHLYGFWPFPRDSQGKPVKYHYYDTLTYEYTSHA
Thythy_2A     TTGLLMYTMATRFCEEIHLYGFWPFPRNSQGKPVKYHYYDTLTYEYTSHA
Salsal_2A     TTGLLMYTMATRFCEEIHLYGFWPFPRDSQGKPVKYHYYDTLTYEYTSHA
Salalp_2A     TTGLLMYTMATRFCEEIHLYGFWPFPRDSQGKPVKYHYYDTLTYEYTSHA
Oncmyk_2B     TTGLLMYTMATRFCEEIHLYGFWPFPQDSQGKPVKYHYYDTLTYEYTSHA
Onckis_2B     TTGLLMYTMATRFCEEIHLYGFWPFPQDSQGKPVKYHYYDTLTYEYTSHA
Salalp_2B     TTGLLMYTMATRFCEEIHLYGFWPFPQDSQGKPVKYHYYDTLTYEYTSHA
Salsal_2B     TTGLLMYTMATRFCEEIHLYGFWPFPQDSQGKSVKYHYYDTLTYEYTSHA
Thythy_2B     TTGLLMYTMATRFCEEIHLYGFWPFPKDSQGKPVKYHYYDTLTYEYTSHA
Onctsh_2B     TTGLLMYTMATRFCEEIHLYGFWPFPQDSQGKPVKYHYYDTLTYEYTSHA
Cormar_2B     TTGLLMYTMATRFCEEIHLYGFWPFPQDSQGKPVKYHYYDTLTYTYTSHA
Corclu_4      STGLLMYTLATRFCDEIHLYGFWPFPRDSNGNVVKYHYYDLLKYRYFSNA
Corlav_4      STGLLMYTLATRFCDEIHLYGFWPFPRDSNGNVVKYHYYDLLKYRYFSNA
Onckis_4      STGLLMYTLATRFCDEIHLYGFWPFPRDANGNMVKYHYYDMLKYRYFSNA
Oncmyk_4      STGLLMYTLATRFCDEIHLYGFWPFPRDANGNMVKYHYYDMLKYRYFSNA
Salsal_4      STGLLMYTLATRFCDEIHLYGFWPFPRDANGNMVKYHYYDMLKYRYFSNA
Salalp_4      STGLLMYTLATRFCDEIHLYGFWPFPRDANGKIVKYHYYDMLKYRYFSNA
Salfon_4      STGLLMYTLATRFCDEIHLYGFWPFPRDANGKIVKYHYYDMLKYRYFSNA
Thythy_4      STGLLMYTLATRFCDEIHLYGFWPFPRDANGNVVKYHYYDMLKYRYFSNA
              351
Cluhar2       SPHTMPLEFRTLSTLHKQGALRLHTSSCSSPAA
Denclu_2      SPHTMPLEFRTLRALHRQGALRLHTGTCSPAA-
Danrer_2      SPHTMPLEFRTLSALHRQGALRLHTGPCKPPT-
Hipcom_2      SPHSMPLEFRTLRALHHQGALRLHTGNCHAQPE
Cormar_2A     SPHTMPLEFRTLSSLHRQGALRLNTGSCDAGM-
Oncmyk_2A     SPHTMPLEFRTLSSLHRQGALRLNTGSCDVGT-
Onctsh_2A     SPHTMPLEFRTLSSLHRQGALRLNTGSCDTGM-
Thythy_2A     SPHTMPLEFRTLSSLHRQGALQLNTGSCDARM-
Salsal_2A     SPHAMPLEFRTLSSLHRQGALRLNTGSCDAGM-
Salalp_2A     SPHTMPLEFRTLSSLHRQGALRLNTGSCDAGM-
Oncmyk_2B     SPHTMPLEFRTLSSLHRQGALRLHTGSCDVGT-
Onckis_2B     SPHTMPLEFRTLSSLHRQGALRLHTGSCDVGT-
Salalp_2B     SPHTMPLEFRTLSSLHRQGALRLHTGSCGVGT-
Salsal_2B     SPHTMPLEFRTLSSLHRQGALRLHTGSCDVGT-
Thythy_2B     SPHTMPLEFRTLSSLHRQGALRLHTGSCDAGI-
Onctsh_2B     SPHTMPLEFRTLSSLHRQGALRLHTGSCDVGT-
Cormar_2B     SPHTMPLEFRTLSSLHRQGALRLHTGSCDAGT-
Corclu_4      GPHRMPLEFKTLKMLHSKGALKLTTSKCES---
Corlav_4      GPHRMPLEFKTLKMLHSKGALKLTTSKCES---
Onckis_4      SPHRMPLEFKTLKMLHSKGALKLTTSKCES---
Oncmyk_4      GPHRMPLEFKTLKMLHSKGALKLTTSKCES---
Salsal_4      GPHRMPLEFKTLKMLHSKGALKLTTSKCES---
Salalp_4      GPHRMPLEFKTLKMLHSKGALKLTTSKCES---
Salfon_4      GPHRMPLEFKTLKMLHSKGALKLTTSKCES---
Thythy_4      GPHRMPLEFKTLKMLHSKGALKLTTSKCES---
```

Figure 5B and C

```
CLUSTAL X (1.81) multiple sequence alignment
                1
Latcal_3        IVFSIGENFRSLLPE----VSPILN---KHYNVCAVVGNSGILTGSRCGP
Perflu_3        IVFSIGENFHSLLPA----VSPILN---KHYNVCAVVGNSGILTGSHCGA
Serdum_3        IVFSIGENFRSLLPE----VSPILN---KHYNVCAVVGNSGILTGSRCGP
Stepar_3        IVFSIGENFRSLLPE----ASPILN---KHYNVCAVVGNSGILTGSRCGP
Tetcal_3        VVFSVSNSFRSLLPE----VPPILK---MHYNTCAVVGNSGILTGSSCGA
Monalb_3        IVFSISENFRSLLPK----VSPVLN---KHYNVCAVVGNSGILTGSRCGP
Molmol_3        IVFSIGENFRSLLPE----TSPILN---KHFNVCAVVGNSGILTGSRCGA
Takrub_3        VVFSIGENFRSLLPE----TSPILQ---KHYNVCAVVGNSGILTGSRCGQ
Canlupfam_3     VVFSISNNFRSLLPD----VSPIVN---KHFNICAVVGNSGILTGSRCGQ
Bostau_3        VVFSISNNFRSLLPD----VSPIVN---KRYNICAVVGNSGILTGSRCGP
Homsap_3        VVFSISNNFRSLLPD----VSPIMN---KHYNICAVVGNSGILTFIQCGR
Siltro_3        VVFSISNNFRSLLPD----TSPVMN---KRYNVCAVIGNSGILTESQCGA
Anocar_3        VVFSISNSFRMLLPD----VSPIQN---KHYNICAVVGNSGILVGSQCGQ
Musmus_3        VVFSISNNFRSLLPD----VSPIMN---KRYNVCAVVGNSGILTGSQCGQ
Pantro_3        VVFSISNNFRSLLPD----VSPIMN---KHYNICAVVGNSGILTGSQCGQ
Ratnor_3        VVFSISNNFRSLLPD----VSPILN---KRYNICAVVGNSGILTGSQCGQ
Galgal_3        VVFSISNNFRSLLPD----VSPILN---KHYNVCAVVGNSGILTGSQCGQ
Lepocu_3        VVFSISNNFRSLLPD----TSPILN---KQYNVCAVVGNSGILTGSQCGP
Tetnig_3        IVFSIGENFRSLLPE----VSPIRQ---KHYNVCAVVGNSGILTGSRCGQ
Squaca_3        VVFSVSNSFRSLLPE----VPPILK---MHYNTCAVVGNSGILTGSGCGA
Perfla_3        IVFSIGENFHSLLPA----VSPILN---KHYNVCAVVGNSGILTGSRCGA
Salsal_3        IVFSIGENFKSLLPE----ASPIVN---KHYNVCAVVGNSGILTGSRCGP
Serlaldor_3     IVFSIGENFRSLLPE----ASPILN---KHYNVCAVVGNSGILTGSRCGP
Treber_3        IVFSISNNLKSLLPD----TSPIRN---KHYSMCAVVGNSGILTGSHCGP
Cynsem_3        IVFSIGENFRSLLPE----VSPIVN---KHYNVCAVVGNSGILTGSRCGP
Paroli_3        IVFSISNNFKSLLPD----ASPVLN---KHYSVCAVVGNSGILTGSHCGP
Paroli_3b       IVFSIGENFRSLLPD----ASPILN---KHYNVCAVVGNSGILTGSRCGP
Plaste_3        IVFSISNNFKSLLPD----ASPLLN---KHYSVCAVVGNSGILTGSHCGP
Plaste_3b       IVFSIGENFRSLLPE----ASPILN---KHYNVCAVVGNSGILTGSRCGP
Erpcal3         VVFSISNNFKSLLPD----ASPVLN---KHYNVCAVVGNSGILTGSQCGP
Calmil_3b       VVFSISNNFRMLLPE----VSPLLK---MHYNTCAVVGNSGILRGSRCGD
Hetzeb_3        IVFSVSNSFRSLLPD----VPPILK---MHYNTCAVVGNSGILIGSRCGA
Latcha_3        VVFSISNHFKSLLPD----VSPILN---KCYNICAVVGNSGILTDSQCGA
Anates_3        IVFSIGENFRSLLPD----ASPILN---KHYNVCAVVGNSGILTGSRCGP
Ampcit3         IVFSISENFRSLLPE----VSPILN---KHYNVCAVVGNSGILTGSRCGP
Auslim_3        IVYSIGENFRSLLPE----ASPILN---KRYNVCAVVGNSGILTGSRCGA
Cypvar_3        IVYSIGENFRSLLPE----VSPILN---KQYNVCAVVGNSGILTGSRCGA
Krymar_3        IVYSIGENFRSLLPE----VSPILD---KHYNVCAVVGNSGILTGSRCGA
Funhet_3        IVYSIGENFRSLLPE----ASPVLN---KRYNVCAVVGNSGILTGSRCGA
Gamaff3         IVYSIGDNFRSLLPD----VSPILN---MRYNVCAVIGNSGILTGSRCGA
Orylat_3        MVYSIGENFRSLLPE----VSPILN---RHYNVCAVVGNSGILTGSLCGA
Orenil_3        IVFSISENFRSLLPE----VSPILN---KHYNVCAVIGNSGILTGSRCGP
Mayzeb_3        IVFSISDNFRSLLPE----VSPILN---KHYNVCAVIGNSGILTGSRCGP
Punnye_3        IVFSISDNFRSLLPE----VSPILN---KHYNVCAVIGNSGILTGSRCGP
Punnye_3a       IVFSISDNFRSLLPE----VSPILN---KHYNVCAVIGNSGILTGSRCGP
Neobri_3        IVFSISDNFRSLLPE----VSPILN---KHYNVCAVIGNSGILTGSRCGP
Misang_3        VVFSIGENLRSLLPD----SSPIIN---KRYNTCAVIGNSGILTGSRCGP
Sinrhi3         IVFSIGENLRSLLPD----SSPVLN---KHFNTCAVVGNSGILTGSRCGA
Singra_3        VVFSIGENLRSLLPD----SSPVLN---KRFNSCAVVGNSGILTGSRCGT
Hapbur_3        IVFSISDNFRSLLPE----VSPILN---KHYNVCAVIGNSGILTGSRCGP
Astcal_3        IVFSISDNFRSLLPE----VSPILN---KHYNVCAVIGNSGILTGSRCGP
Notfur_3        IVYSIGENFRSLLPN----VSPILN---KHYNVCAVVGNSGILTGSRCGT
Cypcar_3        YVFSIGENLRSLLPD----SSPVLN---KRFNSCAVVGNSGILTGSRCGA
Poefor3         IVYSIGENFRSLLPE----VSPVLN---KRYNICAVIGNSGILTGSRCGA
Poelat_3        IVYSIGENFRSLLPE----VSPVLN---KRYNICAVIGNSGILTGSRCGA
Poeret_3        IVYSIGENFRSLLPE----VSPVLN---KRYNVCAVIGNSGILTGSRCGA
Angang_3        VVFSIGENLMSLLPE----SSPIVPE--RRFNVCALVGNSGVLTGSRCGP
Xipmac_3        IVYSIGENFRSLLPE----VSPVLN---KRYNVCAVIGNSGILTGSRCGA
Masarm_3        IVFSIGENFYSLVPE----VSPILN---KHYNICAIIGNSGILSGSRCGS
Permag_3        VVYSIGENFYSLLPE----ASPILH---RHYKRCAVVGNSGILTGSKCGT
Gnapet3         VVFSISSNFKSLLPE----SSPIQN---KHYNVCAVVGNSGILTGSSCGP
Ostbic_3        VVFSVSSSFRSLLPD----SSPIEK---KHYNVCAVVGNSGILTGSSCGP
Gadmor_3        IVFSIGQNFHSLLPE----ASPILN---KHYNVCAVVGNSGILTGSKCGP
Sclfor_3        VVFSVSGSFRSLLPD----SSPVEK---KHYNVCAVVGNGGILTGSSCGP
Acapol3         IVFSIGENFRSLLPD----VSPILN---KHYNVCAVIGNSGILTGSRCGP
Ampoce_3        IVFSIGENFRSLLPD----VSPILN---KHYNVCAVVGNSGILTGSRCGP
Sclfor_3b       VVFAISNNFKSLLPV----TSPIQN---KHYNTCAVVGNSGILTSSHCGH
Ostbic_3b       VVFAISNNFKSLLPE----TSPIQN---KHYNTCAVVGNSGILTSSHCGH
Parkin_3        VVFSISSNFKSLLPE----SSPIQN---KHYNVCAVVGNSGILTGSSCGH
Ampper3         IVFSIGENFRSLLPD----VSPILN---KHYNVCAVVGNSGILTGSRCGP
Oncmyk_3        IVFSIGENFKSLLPE----ASPIVN---KHYNVCAVVGNSGVLTGSRCGP
Gasacu_3        IVFSIGENFRSLLPE----VSPILN---KHYNVCAVVGNSGILTGSRCGP
```

```
Labber_3       IVFSIGENFRSLLPE----VSPILN---KHYNVCAVIGNSGILTGSRCGP
Panbuc_3       VVFSISSSFRSLLPD----HSPILN---KHYNTCAVVGNSGILIGSRCGP
Parhas3        VVFSIGENFRSLLPD----SSPVLN---KRYNTCAVVGNSGILTGSRCGA
Cluhar_3       VVFSIGENFRSLLPE----LSPILN---KRYNTCAVVGNSGILTGSRCGP
Aloalo_3       VVFSIGENFRSLLPE----ISPILN---KRYNTCAVVGNSGILTGSRCGP
Panhyp_3       VVFSIGENFRSLLPD----SSPILN---KRYNTCAVVGNSGILTGSRCGA
Eleele_3       VVFSIGENFRSLLPD----SSPILN---KLYNTCAVVGNSGILTGSRCGA
Denclu_3       IVFSIGENFKSLLPE----TSPVMN---KLYNTCAVVGNSGILMGSRCGP
Sarpil_3       VVFSIGENLRSLLPE----ISPILN---KRYNTCAVVGNSGILTGSRCGP
Eigvir_3       VVFSIGENFRSLLPD----SSPILN---KRYNTCAVVGNSGILTDSRCGS
Aptalb_3       VVFSIGENFRSLLPD----SSPVLN---KRYNTCAVVGNSGILTGSRCGA
Sinans_3       VVFSIGENLRSLLPD----SSPVLN---RRFNTCAVVGNSGILTGSRCGA
Pygnat_3       VVFSIGENFRSLLPD----SSPILN---KRYNTCAVIGNSGILTGSRCGP
Astmex_3       IVFSIGENFRSLLPD----SSPVLN---KRYNTCAVVGNSGILTGSRCGP
Caraur_3       VVFSIGENLRSLLPD----SSPVLN---KRFNSCAVVGNSGILTGSRCGA
Danrer_3       VVFSIGENLRSLLPD----ASPVLN---KRYNTCAVVGNSGILTGSRCGP
Ictpun_3       YVFSIGENFRSLLPD----SSPILN---KRYNTCAVVGNSGILTGSRCGA
Plealt_3       YVFSIGENFKSLLPE----ASPVVN---KHYNVCAVVGNSGILTGSRCGA
Plealt_9       YVFSISNNFKSLLPE----TSPILN---KHYNVCAVVGNSGILTGSHCGS
Gasacu_9       IVFSISNNLKSLLPD----ASPIRN---KHYSVCAVVGNSGILTGSHCGP
Parkin_9       VVFAISGNFKSLLPD----SSPILN---KHYNMCAVVGNSGILTGSRCGP
Acapol_9       IVFSISNNFKSLLPE----TSPIHN---KHYSMCAVVGNSGILTGSHCGP
Larcro_9       IVFSISNNFKSLLPE----TSPIHN---KHYSICSVVGNSGILTGSHCGP
Notcor_9       IVFSISNNLKSLLPD----TSPIRN---KHYSMCAVVGNSGILTGSHCGP
Latcal_9       IVFSISNNFKSLLPD----TSPILN---KHYSMCAVVGNSGILTGSHCGP
Serlaldor_9    IVFSISNNFKSLLPD----TSPILN---KHYSMCAVVGNSGILTGSHCGP
Monalb_9       IVFSISNSFKSLLPD----TSPILN---KHYSMCAVVGNSGILTGSHCGP
Serdum_9       IVFSISNNFKSLLPD----TSPILN---KHYSMCAVVGNSGILTGSHCGP
Perflu_9       IVFSISNNLKSLLPD----TSPIHN---KHYSMCAVVGNSGILTGSHCGP
Perfla_9       IVFSISNNLKSLLPD----TSPIHN---KHYSMCAVVGNSGILTGSHCGP
Stepar_9       IVFSISNNFKSLLPE----TSPIHN---KHYSVCAVVGNSGILTGSHCGP
Molmol_9       IVFSISNNFKSLLPD----TSPIRN---KHYSICSVVGNSGILTGSHCGP
Hipcom_9       IVFSISNNFKSLLPD----ASPILD---KHYGTCAVVGNSGILTGSHCGS
Takrub_9 B     IVFSISNNFKSLLPD----TSPIHN---KHYSLCSVVGNSGILTGSHCGD
Takrub_9       IVFSISNNFKSLLPD----TSPIHN---KHYSLCSVVGNSGILTGSHCGA
Cynsem_9       IVFSISNNFKSLLPD----ISPILN---KHYSMCAVVGNSGILTGSHCGP
Scomax_9       IVFSISNNLKSLLPD----ISPILN---KHYSVCAVVGNSGILTGSHCGP
Corlav_9       VVFSISNNLKSLLPD----ASPIQN---KHYNVCAVVGNSGILTGSHCGP
Oncmyk_9       VVFSISNNLKSLLPD----ASPIQN---KHYNVCAVVGNSGILTGSHCGP
Salsal_9       VVFSISNNLKSLLPD----ASPIQN---KHYNVCAVVGNSGILTGSHCGP
Onctsh_9       VVFSISNNLKSLLPD----ASPIQN---KHYNVCAVVGNSGILTGSHCGP
Salalp_9       VVFSISNNLKSLLPD----ASPIQN---KHYNVCAVVGNSGILTGSHCGP
Ampoce_9       IVFSISNNFKSLLPE----TSPIHN---KHYSMCAVVGNSGILTGSHCGP
Ampper9        IVFSISNNFKSLLPE----TSPIHN---KHYSMCAVVGNSGILTGSHCGP
Labber_9       IVFSISNNFKSLLPD----TSPIRN---KHYSICSVVGNSGILTGSHCGP
Umbpyg_3B      IVFSIGENFQSLLPE----VSPIVD---KHYNVCAIVGNSGILTGSRCGP
Esoluc_3b      VVFSIGENFKSLLPE----ASPIAN---KHYNVCAVVGNSGILTGSRCGP
Gadmor_9       IVFSISNNFKSLLPD----MSPIYN---KHYNVCAVVGNSGILTGSRCGN
Umbpyg_9       VVFSISNNLKSLLPD----ASPIQN---KHYNVCAVVGNSGILTGSHCGP
Esoluc_9       VVFSISNNLKSLLPD----ASPIQN---KHYNVCAVVGNSGILTGSHCGP
Permag_9       IVFSISNNFKSLLPD----TSPILN---KHYSVCSVVGNSGILTDSHCGP
Bolpec_9       IVFSISNNFKSLLPD----TSPILN---KHYSVCSVVGNSGILTDSHCGP
Poefor9        IVFSISNNFKSLLPE----ASPILN---KHYSTCAVVGNSGILTGSHCGP
Masarm_9       IVFSISNNFKSLLPD----TSPILN---KHYSMCAVVGNSGILTGSHCGP
Angjap_9       VVFSIGENFMSLLPE----SSPIVPE--RRFNVCALVGNSGVLTGSRCGP
Poelat_9       IVFSISNNFKSLLPE----ASPILN---KHYSTCAVVGNSGILTGSHCGP
Poeret_9       IVFSISNNFKSLLPE----ASPILN---KHYSTCAVVGNSGILTGSHCGP
Xipmac_9       IVFSISNNFKSLLPD----ASPIRN---KHYSTCAVVGNSGILTGSHCGP
Hapbur_9       IVFSISNNFKSLLPD----TSPILN---KHYSMCAVVGNSGILTGSHCGP
Orylat_9       IVFSISNNFKSLLPE----ISPILN---KHYSVCAVVGNSGILTGSHCGP
Gamaff_9       IVFSISNNFKSLLPD----ASPIRN---KHYSTCAVVGNSGILTGSHCGP
Neobri_9       IVFSISNNFKSLLPD----TSPILN---KHYSMCAVVGNSGILTGSHCGP
Funhet_9       IVFSISNNFKSLLPE----TSPILN---KHYSTCAVVGNSGILTGSHCGP
Punnye_9       IVFSISNNFKSLLPD----TSPILN---KHYSMCAVVGNSGILTGSHCGP
Cypvar_9       IVFSISNNFKSLLPE----TSPILN---KHYSTCAVVGNSGILTGSHCGP
Krymar_9       IVFSISNNFKSLLPD----ISPIFN---KHYSMCAVVGNSGILTGSHCGP
Notfur_9       IVFSISNNFKSLLPD----TSPILN---KHYSMCAVVGNSGILTGSHCGP
Notpie_9       IVFSISNNFKSLLPD----TSPILN---KHYSMCAVVGNSGILTGSHCGP
Orenil_9       IVFSISNNFKSLLPD----TSPILN---KHYSMCAVVGNSGILTGSHCGP
Auslim_9       IVFSISNNFKSLLPE----TSPITN---RHYSVCAVVGNSGILTGSHCGP
Anates_9       IVFSISNNFKSLLPD----TSPIHN---KHYSMCAVVGNSGILTGSHCGP
Astcal_9       IVFSISNNFKSLLPD----TSPILN---KHYSMCAVVGNSGILTGSHCGP
Mayzeb_9       IVFSISNNFKSLLPD----TSPILN---KHYSMCAVVGNSGILTGSHCGP
Orymel_9       IVFSISNNFKSLLPD----ISPILN---KHYSVCAVVGNSGILTGSHCGP
Ampcit9        IVFSISNNFKSLLPD----TSPILN---KHYSMCAVVGNSGILTGSHCGP
Macfas_2       ATMNVSQNLYELLPR----TSPLKN---KHFGTCAIVGNSGVLLNSGCGQ
Macmul_2       ATMNVSQNLYELLPR----TSPLKN---KHFGTCAIVGNSGVLLNSGCGQ
```

```
Macnem_2       ATMNVSQNLYELLPR----TSPLKN---KHFGTCAIVGNSGVLLNSGCGQ
Musmus_2       ATMNVSQNLYELLPR----TSPLKN---KHFQTCAIVGNSGVLLNSGCGQ
Ponabe_2       ATMNVSQNLYELLPR----TSPLKN---KHFGTCAIVGNSGVLLNSGCGQ
Papanu_2       ATMNVSQNLYELLPR----TSPLKN---KHFGTCAIVGNSGVLLNSGCGQ
Nomleu_2       ATMNVSQNLYELLPR----TSPLKN---KHFRTCAIVGNSGVLLNSGCGQ
Pantro_2       ATMNVSQNLYELLPR----TSPLKN---KHFGTCAIVGNSGVLLNSGCGQ
Latcha_2       ATMNISQNLYKLLPR----VSPLKN---RHFQNCAIVGNSGILLNSNCGR
Gnapet2        ATMNISDSLYQLLPT----VSPMKN---QHHRRCAIVGNSGILLNSSCGP
Panbuc_2b      ATTNVSENLYQLLPS----VSPLKH---QHYGRCAIVGNSGILLNSSCGQ
Ostbic_2       ATMNVSNELYELLPK----VSPLKN---QFHKRCAIVGNSGILLNSSCGK
Sinrhi2        ATTNISENLYHLLPT----VSPMKN---QHYKQCAIVGNSGILLNSSCGR
Hetzeb_2       ATLNISHSLYELLPR----TSPMKN---KHFKQCAIVGNSGILLNSGCGQ
Hipcom_2       PTTNISENLHRLLPS----VSPLKN---RHYRLCAIVGNSGILLNSSCGS
Prigla_2       ATLNVSHSLYELLPR----ISPMKN---KHYKQCAIVGNSGILLNSGCGQ
Scytor_2       ATLNISHSLYELLPR----TSPMKN---KHFKQCAIVGNSGILLNSGCGQ
Tetcal_2       ATLNISYSLYELLPR----TSPMKN---KHFKQCAIVGNSGILLNSGCGQ
Rhityp_2       ATLNISHSLYELLPR----TSPMKN---KHFKECAIVGNSGILLKSGCGQ
Parkin_2b      ATTNVSENLHALLPT----TSPMKN---RHYQRCAIVGNSGILLNSSCGK
Angang_2       ATVNVSESLYRLLPT----VSPMKD---RHYQRCAIVGNSGILLNSSCGR
Pygnat_2       ATTNISENLYRLLPT----ASPMKN---QHYEQCAIVGNSGILLNSSCGA
Parkin_2       ATMNVSASLYQLLPT----VSPMKN---QHHKRCAIVGNSGILLNSSCGP
Gorgor_2       ATMNVSQNLYELLPR----TSPLKN---KHFGTCAIVGNSGVLLNSGCGQ
Thegel_2       ATMNVSQNLYELLPR----TSPLKN---KHFGTCAIVGNSGVLLNSGCGQ
Tupchi_2       ATMNVSQNLYELLPR----TSPLKN---KHFRTCAIVGNSGVLLNSGCGQ
Galgal_2       ATMNVSQNLYELLPR----TSPLKG---KQFPSCAIVGNSGVLLGSGCGP
Eleele_2       ATTNISENLYRLLPT----VSPMKN---QHYKRCAIVGNSGILLNSSCGP
Danrer_2       ATTNISENRYHLLPT----VSPMKN---QHYRKCAIVGNSGILLNSSCGR
Canlupfam_2    ATMNVSQNLYDLLPR----TSPLKN---KHFRTCAIVGNSGVLLNSGCGQ
Aloalo_2       PTMNVSENLYRLLPT----ASPMKN---QRHKRCAIVGNSGILLNSSCGS
Astmex_2       ATTNISENLYRLLPT----VSPMKN---QHYERCAIVGNSGILLNSSCGP
Sinans_2       ATTNISENLYHLLPT----VSPMKN---QHYKQCAIVGNSGILLNSSCGR
Sclfor_2       ATLNVPNELYELLPR----VSPLKN---QFYKRCAIVGNSGILLNSSCGQ
Ceraty_2       ATMNVSQNLYELLPR----TSPLKN---KHFGTCAIVGNSGVLLNSGCGQ
Anocar_2       ATMNVSQNLYALLPR----TSPLKG---KHFRTCAIVGNSGILLNSGCGE
Anates_2       PTTNISENLYRLLPT----VSPMKN---QHHRRCAIVGNSGILLNSSCGH
Ampbic_2       PTTNISENLYRLLPT----VSPMKN---QHHRRCAIVGNSGILLNSSCGL
Sarpil_2       PTMNVSENLYSLLPT----ASPMKN---QHHKRCAIVGNSGILLNSSCGS
Xenlae_2       ATMNISKNLYELLPR----TSPLKN---KHFKTCAIVGNSGILLNSGCGK
Cluhar_2       PTMNVSENLYRLLPT----ASPMKN---QHHKSCAIVGNSGILLNSSCGP
Cypcar_2       ATTNISENLYNLLPT----VSPMKN---QHYKQCAIVGNSGILLNSSCGR
Denclu_2       ATMNISENLYRLLPT----ASPMKN---QHHRLCAIVGNSGILLNSSCGP
Notpie_2       PTTNISENLYRLLPT----ASPMKN---QHHRKCAIVGNSGILLNSSCGP
Erpcal2        ATMNISKSLYQLIPK----QSPLKG---QHFQKCAIVGNSGVLLNSGCGK
Amical_2       ATMNISETLYQLIPK----VSPMKQ---QHYRKCAIVGNSGILLNSRCGE
Lepocu_2       ATMNVSETLYQLIPK----VSPMKK---QHYRQCAIVGNSGILLKSRCGA
Homsap_2       ATMNVSQNLYELLPR----TSPLKN---KHFGTCAIVGNSGVLLNSGCGQ
Bostau_2       ATMNVSQNLYELLPR----TSPLKN---KHFGTCAIVGNSGVLLNSGCGQ
Caraur_2       ATTNISENLYNLLPT----VSPMKN---RHYKQCAIVGNSGILLNSSCGR
Thythy_2B      PTTNISENLYRLLPT----TSPMKN---QYHRRCAIVGNSGVLLNSSCGP
Onctsh_2B      PTTNISENLYQLLPT----ASPMKN---QHHRHCAIIGNSGILLNSSCGP
Salsal_2B      PTTNISENLYQLLPT----VSPMKN---QYHRRCGIIGNSGILLNSSCGP
Oncmyk_2B      PTTNISENLYQLLPT----ASPMKN---QHHRHCAIIGNSGILLNSSCGP
Onckis_2B      PTTNISENLYQLLPT----ASPMKN---QHHRHCAIIGNSGILLNSSCGP
Salsal_2A      PTTNISENLYRLLPT----ASPMKN---QHHRRCAIVGNSGILLNSSCGP
Thythy_2A      PTTNISENLYRLLPT----ASPMKN---QHHRRCAIVGNSGILLNSSCGP
Salalp_2A      PTTNISENLYRLLPT----ASPMKN---QHHRRCAIVGNSGILLNSSCGP
Corlav_2A      PTTNISENLYRLLPT----ASPMKN---QHHRRCAIVGNSGILLNSSCGP
Cormar_2A      PTTNISENLYRLLPT----ASPMKN---QHHRRCAIVGNSGILLNSSCGP
Onctsh_2A      PTTNISENLYRLLPT----ASPMKN---QHHRSCAIVGNSGILLNSSCGP
Oncmyk_2A      PTTNISENLYQLLPT----ASPMKN---QHHRSCAIVGNSGIQLNSSCGP
Neobri_2       PTTNISENLYRLLPT----VSPMKN---QHYRRCAIVGNSGILLNSSCGP
Takrub_2       PTTNVSETLYHFLPT----VSPMKN---QHHRRCAIVGNSGILLNSSCGP
Tetnig_2       PTTNISETLYHFLPS----VSPMKN---QHHRRCAIVGNSGVLLNSSCGP
Orenil_2       PTTNISENLYRLLPT----ISPMKN---QHYRRCAIVGNSGILLNSSCGP
Orylat_2       PTTNISENLYRLLPT----VSPMKN---QHHRRCAIVGNSGILLNSSCGS
Krymar_2       PTTNISENLYRLLPT----ASPMRR---QHHRRCAIVGNSGVLLNSSCGP
Notfur_2       PTTNISENLYRLLPT----ASPMKN---QHHRKCAIVGNSGILLNSSCGP
Poelat_2       PTTNISENLYRLLPT----ASPMKN---QYHRRCAIVGNSGILLNSSCGP
Gadmor_2       PTTNISENLYRLLPT----VSPMRN---QHHRKCAIVGNSGILLNSSCGQ
Xipmac_2       PTTNISENLYRLLPT----ASPMKN---QYHRRCAIVGNSGILLNSSCGP
Poeret_2       PTTNISENLYRLLPT----ASPMKN---QYHRRCAIVGNSGILLNSSCGS
Astcal_2       PTTNISENLYRLLPT----VSPMKN---QHYRRCAIVGNSGILLNSSCGP
Hapbur_2       PTTNISENLYRLLPT----VSPMKN---QHYRRCAIVGNSGILLNSSCGP
Acapol_2B      PTTNISENLYRLLPT----VSPMKN---QHHRRCAIVGNSGILLNSSCGL
Ampoce_2A      PTTNISENLYRLLPT----VSPMKN---QHHRRCAIVGNSGILLNSSCGL
Acapol_2A      PTTNISENLYRLLPT----VSPMKN---QHHRRCAIVGNSGILLNSSCGL
Mayzeb_2       PTTNISENLYRLLPT----VSPMKN---QHYRRCAIVGNSGILLNSSCGP
```

```
Punnye_2        PTTNISENLYRLLPT----VSPMKN---QHYRRCAIVGNSGILLNSSCGP
Latcal_2        PTTNISENLYRLLPT----VSPMKN---QHHRRCAIVGNSGILLNSSCGP
Singra_2        ATTNISENLYHLLPT----VSPMKN---QHYKQCAIVGNSGILLNSSCGR
Auslim_2        PTTNVSENLYRLLPT----VSPLKH---QHHRSCAIVGNSGVLLNSSCGP
Monalb2         PTTNISENLYRLLPT----VSPMKN---QHX-----TGKK----NSSCGP
Ampoce_2B       PTTNISENLYRLLPT----VSPMKN---QHHRRCAIVGNSGILLNSSCGL
Chiham_2        PTTNISENLYRLLPT----VSPMKN---QHHKRCAIVGNSGILLNSSCGP
Gasacu_2        PTTNVSENLYRLLPT----ASPMKN---QHHRRCAIVGNSGILLNSSCGP
Gymacu_2        PTTNISENLYRLLPT----VSPMKN---QHHKRCAIVGNSGILLNSSCGP
Labber_2        PTTNISENLYRLLPT----ASPLKN---QHHRRCAIVGNSGILLNSSCGP
Cypvar_2        PTTNISENLYRLLPT----ASPLKN---QYHKRCAIVGNSGILLNSSCGP
Funhet_2        PTTNISEDLYRLLPT----ASPMKN---QYHRRCAIVGNSGILLNSSCGP
Larcro_2        PTTNISQNLYRLLPT----VSPMKN---QHHRRCAIVGNSGILLNSSCGS
Paroli_2        PTTNISENLYRLLPT----VSPMKN---QHHRRCAIVGNSGILLNSSCGH
Perflu_2        PTINISENLYRLLPT----VSPMKN---QHHRRCAIVGNSGILLNSSCGP
Serdum_2        PTTNISENLYRLLPT----VSPMKN---QHHRRCAIVGNSGILLNSSCGP
Stepar_2        PTTNISENLYRLLPT----VSPMKN---QHHRRCAIVGNSGILLNSSCGL
Notcor_2        PTTNISENLYRLLPT----VSPMKN---QHHKRCAIVGNSGILLNSSCGP
Treber_2        PTTNISENLYRLLPT----VSPMKN---QHHKRCAIVGNSGILLNSSCGP
Serlaldor_2     PTTNISENLYRLLPT----ISPMKN---QHHRRCAIVGNSGILLNSSCGP
Plaste_2        PTTNISENLYRLLPT----VSPMKN---QHHRRCAIVGNSGVLLNSSCGQ
Clabat_4        ATLNVSHTLHSLLPE----VSPLKN---KRFKTCAVVGNSGVLLKSGCGK
Ictpun_4A       ATLNVSHTLHSLLPD----VSPLKN---KRFKTCAVVGNSGVLLKSGCGK
Panhyp_4        ATLNVSHTLHSLLPE----VSPLKN---KRFKTCAVVGNSGVLLKSGCGK
Umbpyg_4        ATLNISHTLHSLLPD----VSPLKN---KRFRTCALVGNSGVLLRSGCGR
Esoluc_4        ATLNVSRTLHSLLPD----VSPLKN---KRFRTCAVVGNSGVLLNSGCGR
Plealt_4        ATLNVSQTLHSLLPE----VSPLKN---KRFNTCAVVGNSGVLLNSSCGK
Onckis_4B       ATLNVSHTLHSLLPD----VSPLKN---KRFRTCAVVGNSGVLLNSGCGK
Onckis_4A       ATLNVSHTLHSLLPD----VSPLKN---KRFRTCAVVGNSGVLLNSGCGK
Onctsh_4        ATLNVSHTLHSLLPD----VSPLKN---KRFRTCAVVGNSGVLLNSGCGK
Oncmyk_4        ATLNVSHTLHSLLPD----VSPLKN---KRFRTCAVVGNSGVLLNSGCGK
Corlav_4        ATLNISHTLHSLLPD----VSPLKN---KRFKTCAVVGNSGVLLNSGCGK
Astmex_4A       ATLNVSHTLHSLLPE----VSPLKN---KRFKTCAVVGNSGVLLKSGCGK
Pygnat_4        ATLNVSHTLHSLLPE----VSPLKN---KRFRTCAVVGNSGILLKSGCGK
Aloalo_4        ATLNVSQTLHKLLPE----VSPLKN---RRFKTCAVVGNSGVLLSSGCGQ
Cluhar_4        ATLNVSQTLHKLLPE----VSPLKN---RRFKTCAVVGNSGVLLSSGCGH
Denclu_4        ATLNISRTLHNLLPE----VSPLKN---RRFKTCAVVGNSGILLNSGCGR
Konpun4         ATLNVSQTLHKLLPE----VSPLKN---RRFKTCAVVGNSGVLLNSGCGQ
Sarpil_4        ATLNVSQTLHKLLPE----VSPLKN---RRFKTCAVVGNSGVLLSSGCGQ
Caraur_4        ATFNISQTLHSLLPE----VSPMKN---KTFKTCAVVGNSGVLLKSGCGK
Cteide_4        ATFNVSHTLHSLLPE----VSPLKN---KMFKTCAVVGNSGILLKSGCGK
Petmar_4        ATTNVSHSLHALLPT----APPLRG---GRYGSCAVVGNSGILEGSACGE
Cypcar_4        ATFNISQTLHSLLPE----VSPLKN---KTFKTCAVVGNSGVLLKSRCGK
Danrer_4        ATFSVSQTLHSLLPE----VSPLKN---KTFKTCAVVGNSGILLKSGCGK
Misang_4        ATFNVTHTLHSLLPE----VSPLKN---KKFKTCAVVGNSGILLKSRCGK
Angjap_4        ATLNVSRALHSLLPA----VSPMKN---RRFRTCAVVGNSGVLLNSGCGP
Cypcar_4A       ATFNISQTLHSLLPE----VSPLKN---KTFKTCAVVGNSGVLLKSRCGK
Angang_4        ATLNVSRALHSLLPA----VSPMKN---RRFRTCAVVGNSGVLLNSGCGR
Cypcar_4B       ATFNVSQTLHSLLPE----VSPLKN---KTFKTCAVVGNSGVLLKSGCGK
Sinans_4A       VTFNISQTLHSLLPE----VSPLKN---KTFKTCAIVGNSGILLKSRCGK
Sinans_4B       ATFNVSQTLHSLLPE----VSPLKN---KTFKTCSVVGNSGVLLKSGCGK
Singra_4A       ATFNISQTLHSLLPE----VSPLKN---KTFKTCAVVGNSGILLKSRCGK
Aptalb_4        AKLSVSHTLHSLLPE----VSPLKN---RRFKTCAVVGNSGVLLKSGCGK
Eigvir_4        ATLNISHTLHSLLPE----VSPLKN---KRFGTCAVVGNSGVLLKSGCGK
Eleele_4        ATLNVSHTLHSLLPE----VSPLKN---KRFRTCAVVGNSGVLLKSGCGK
Parhas_4        AKLNVSHTLHSLLPE----VSPLKN---RRFKTCAVVGNSGVLLKSGCGK
Sclfor_4        ATLSISQSLHSLLPD----VSPMKN---RRFKTCAVVGNSGILLKSGCGK
Gnapet4         ATLNVSHTLHSLLPD----TSPMKN---RRFNTCAVVGNSGVLLNSGCGK
Ostbic_4        ATLTISQTLHSLLPD----VSPLKN---KRFKTCAVVGNSGILLNSGCGK
Parkin_4        ATLNISHTLHSLLPD----ASPMKN---RRFNTCAVVGNSGVLLNSGCGK
Panbuc_4        ATFNVSHTLHSLLPD----VSPMKN---KRFKTCAVVGNSGVLLNSGCGR
Erpcal4         ATLNISHNLHSLLPE----VSPMKN---RRFKTCAVVGNSGILLGSGCGR
Salsal_4        ATLNVSHTLHSLLPD----VSPLKN---KRFRTCAVVGNSGVLLNSGCGK
Saltru_4        ATLNVSHTLHSLLPD----VSPLKN---KRFRTCAVVGNSGVLLNSGCGK
Salalp_4        ATLNVSHTLHSLLPD----VSPLKN---KRFRTCAVVGNSGVLLNSGCGK
Salfon_4        ATLNVSHTLHSLLPD----VSPLKN---KRFRTCAVVGNSGVLLNSGCGK
Thythy_4        ATLNVSHTLHSLLPD----VSPLKN---KRFKTCAVVGNSGVLLHSGCGK
Calmil_4        ATLNISRNLHSLLPE----VSPMKN---KRFNACAVVGNSGILLGSGCGK
Hetzeb_4        ATLNISHNLHSLLPE----VSPMKN---KRFSTCAVVGNSGILLGSGCGK
Rhityp_4        ATLNISHNLHRLLPE----VSPMKN---KRFSSCAVVGNSGILLGSGCGK
Scytor_4        ATLNISRNLHSLLPD----VSPMKN---KRFSTCAVVGNSGILLGSGCGK
Squaca_4        ATLNISQNLHSLLPE----VSPMKN---KRFSTCAVVGNSGILLGSGCGK
Amical_4        ATLNISHNLHSLLPE----VSPMKN---RRFKTCAVVGNSGILINSGCGR
Lepocu_4        ATLNISHNLHSLLPE----VSPMKN---RRFKTCAVVGNSGILINSGCGK
Agema_4         ATLNVSHTLHSLLPE----VSPLKN---KRFKTCAVVGNSGVLLKSGCGK
Anocar_4        ATLNISQDLHSLLPE----VSPMKN---RRFKMCAVVGNSGILLDSGCGK
Canlupfam_4     ATLNISQDLHSLLPE----VSPMKN---RRFKTCAVVGNSGILLDSECGK
```

```
Crigri_4        ATLNISHDLHSLLPE----VSPMKN---RRFKTCAVVGNSGILLDSGCGK
Galgal_4        ATLNISQDLHSLLPE----VSPMKN---RRFKTCAVVGNSGILLDSGCGK
Mesaur_4        ATLNISHDLHSLLPE----VSPMKN---RRFKTCAVVGNSGILLDSECGK
Homsap_4        ATLNISHDLHSLLPE----VSPMKN---RRFKTCAVVGNSGILLDSECGK
Notscuscu_4     ATLNISQDLHSLLPE----VSPMKN---RRFKTCAVVGNSGILLDSGCGK
Psetextex_4     ATLNISQDLHSLLPE----VSPMKN---RRFKTCAVVGNSGILLDSGCGK
Musmus_4        ATLNISHNLHSLLPE----VSPMKN---RRFKTCAVVGNSGILLDSGCGK
Pogvit_4        ATLNISQDLHRLLPE----VSPMKN---RRFKTCAVVGNSGILLDSGCGK
Promuc_4        ATLNISQDLHSLLPE----VSPMKN---RRFKTCSVVGNSGILLDSGCGK
Pantro_4        ATLNISHDLHSLLPE----VSPMKN---RRFKTCAVVGNSGILLDSECGK
Pytbiv_4        ATLNISQDLHSLLPE----VSPMKN---RRFKTCAVVGNSGILLDSGCGK
Ratnor_2        ATMNVSQNLYELLPR----TSPLKN---KHFQTCAIVGNSGVLLNSGCGQ
Ratnor_4        ATLNISHDLHSLLPE----VSPMKN---RRFKTCAVVGNSGILLDSGCGK
Siltro_2        ATMNISKNLYELLPR----TSPLKN---KHFKTCAIVGNSGILLNSGCGK
Siltro_4        ATLNISQNLHSLLPE----VSPMKS---RRFRTCAVVGNSGILLNSGCGK
Susscr_4        ATLNISQDLHSLLPE----VSPMKN---RRFKTCAVVGNSGILLDSECGK
Taegut_4        ATLNISQDLHSLLPE----VSPMKN---RRFKTCAVVGNSGILLNSGCGK
Termextri_4M    ATLNISQDLHSLLPE----VSPMKN---RRFKTCAVVGNSGILLDSACGK
Thasirsir_4     ATLNISQDLHRLLPE----VSPMKN---RRFKACAVVGNSGILLDSGCGK
Xenlae_4B       ATLNISQNLHSLLPE----VSPMKS---RRFRTCAVVGNSGILLNSGCGN
Xenlae_4A       ATLNISQNLHSLLPE----VSPMKS---RRFRTCAVVGNSGILLSSGCGK
                51
Latcal_3        QIEKFDYVFR--CNFAP-TEIFKKDVGRRTNMTTFN-PSILEKYYNNLLT
Perflu_3        QIEKFDFVFR--CNFAP-TEIFKKDVGRRTNMTTFN-PSILEKYYNNLLT
Serdum_3        QIEKFDFVFR--CNFAP-TEIFKKDVGRRTNMTTFN-PSILEKYYNNLLT
Stepar_3        QIEKFDFVFR--CNFAP-TEIFKKDVGRRTNMTTFN-PSILEKYYNNLLT
Tetcal_3        DIDKSDFVFR--CNFAP-TETFENDVGRKTNLTTFN-PSILEKYYNNLLT
Monalb_3        QIEKFDFVFR--CNFAP-TEVFKKDVGRRTNMTTFN-PSILEKYYNNLLT
Molmol_3        QIEKFDFVLR--CNFAP-TEIFKKDVGRRTNMTTFN-PSILEKYYNNLLT
Takrub_3        QIDRFDFVFR--CNFAP-TEIFKKDVGRRTNMTTFN-PSILEKYYNNLLT
Canlupfam_3     EIDKSDFVFR--CNFAP-TEAFQRDVGRKTNLTTFN-PSILEKYYNNLLT
Bostau_3        QIDKSDFVFR--CNFAP-TEAFQRDVGRKTNLTTFN-PSILEKYYNNLLT
Homsap_3        EIDKSDFVFR--CNFAP-SEAFQRDVGRKTNLTTFN-PSILEKYYNNLLT
Siltro_3        EIDKADFVFR--CNFAP-TEGFQKDVGRKTNLTTFN-PSILEKYYNNLLT
Anocar_3        EIDKYDFVFR--CNFAP-TEAFHKDVGRKTNITTFN-PSILEKYYNNLLT
Musmus_3        EIDKSDFVSR--CNFAP-TEAFHKDVGRKTNLTTFN-PSILEKYYNNLLT
Pantro_3        EIDKSDFVFR--CNFAP-TEAFQRDVGRKTNLTTFN-PSILEKYYNNLLT
Ratnor_3        EIDKSDFVFR--CNFAP-TEAFHKDVGKKTNLTTFN-PSILEKYYNNLLT
Galgal_3        EIDKSDFVFR--CNFAP-TEAFQKDVGRKTNLTTFN-PSILEKYYNNLLT
Lepocu_3        EIDKSDFIFR--CNFAP-TEIFHKDVGKKTNLTTFN-PSILEKYYNNLLT
Tetnig_3        EIDSLDFVFR--CNFAP-TELFKKDVGRRTNMTTFN-PSILEKYYNNLLT
Squaca_3        EIDKSDFVFR--CNFAP-TETFEKDVGKKTNLTTFN-PSILEKYYNNLLT
Perfla_3        QIEKFDFVFR--CNFAP-TEIFKKDVGRRTNMTTFN-PSILEKYYNNLLT
Salsal_3        EIEKFDFVFR--CNFAP-TEIFRRDVGRRTNLTTFN-PSILEKYYNNLLT
Serlaldor_3     QIEKFDFVFR--CNFAP-TEIFKKDVGRRTNMTTFN-PSILEKYYNNLLT
Treber_3        EIDQADFVFR--CNFAP-TEIYSKDVGKKTNLTTFN-PSILERYYNNLLT
Cynsem_3        QIEKFDFVFR--CNFAP-TEVFKKDVGRRTNMTTFN-PSILEKYYNNLLT
Paroli_3        EIDQADFVFR--CNFAP-TEVYSKDVGRKTNLTTFN-PSILERYYNNLLT
Paroli_3b       QIEKFDFVFR--CNFAP-TEIFKKDVGRRTNMTTFN-PSILEKYYNNLLT
Plaste_3        EIDQADFVFR--CNFAP-TEVYSKDVGRKTNLTTFN-PSILERYYNNLLT
Plaste_3b       QIEKFDFVFR--CNFAP-TELFKKDVGRQTNMTTFN-PSILEKYYNNLLT
Erpcal3         EIDKSDFIFR--CNFAP-TEIFQKDVGRKTNLTTFN-PSILEKYYNNLLT
Calmil_3b       SIDKSDFVFR--CNFAP-TEFFEKDVGRKTNLTTFN-PSILEKYYNNLMA
Hetzeb_3        DIDKSDFVFR--CNFAP-TESFEKDVGKKTNLTTFN-PSILEKYYNNLLT
Latcha_3        EIDQSDFVFR--CNFAP-TEIFHKDVGRKTNLTTFN-PSILEKYYNNLLT
Anates_3        QIEKFDFVFR--CNFAP-TEIFKKDVGRRTNLTTFN-PSILEKYYNNLLT
Ampcit3         QIEKYDFVFR--CNFAP-TEIFKKDVGRRTNMTTFN-PSILEKYYNNLLT
Auslim_3        HIERYDYVFR--CNFAP-TEIFRKDVGRRTNMTTFN-PSILEKYYNNLLT
Cypvar_3        QIEKYDFVFR--CNFAP-TEIFKKDVGRRTNMTTFN-PSILEKYYNNLLT
Krymar_3        QIERYDYVFR--CNFAP-TEIFKKDVGRRTNMTTFN-PSILEKYYNNLLT
Funhet_3        QIEKYDFVFR--CNFAP-TEIFKKDVGRRTNMTTFN-PSILEKYYNNLLT
Gamaff3         QIEKYDFVLR--CNFAP-TEIFKKDVGRRTNMTTFN-PSILEKYYNNLLT
Orylat_3        QIEKYDFVFR--CNFAP-TEIFKKDVGRRTNMTTFN-PSILEKYYNNLLT
Orenil_3        QIEKYDFVFR--CNFAP-TEIFKKDVGRRTNMTTFN-PSILEKYYNNLLT
Mayzeb_3        QIEKYDFVFR--CNFAP-TEIFKKDVGRRTNMTTFN-PSILEKYYNNLLT
Punnye_3        QIEKYDFVFR--CNFAP-TEIFKKDVGRRTNMTTFN-PSILEKYYNNLLT
Punnye_3a       QIEKYDFVFR--CNFAP-TEIFKKDVGRRTNMTTFN-PSILEKYYNNLLT
Neobri_3        QIEKYDFVFR--CNFAP-TEIFKKDVGRRTNMTTFN-PSILEKYYNNLLT
Misang_3        EIDKYDFVFR--CNFAP-TEVFRKDVGRRTNLTTFN-PSILEKYYNNLLT
Sinrhi3         QIDSYDFVFR--CNFAP-TEVFRRDVGRRTNLTTFN-PSILEKYYNNLLT
Singra_3        QIDSYDFVFR--CNFAP-TEAFRRDVGRRTNLTTFN-PSILEKYYNNLLT
Hapbur_3        QIEKYDFVFR--CNFAP-TEIFKKDVGRRTNMTTFN-PSILEKYYNNLLT
Astcal_3        QIEKYDFVFR--CNFAP-TEIFKKDVGRRTNMTTFN-PSILEKYYNNLLT
Notfur_3        QIEKYDFVFR--CNFAP-TEIFKKDVGRRTNMTTFN-PSILEKYYNNLLT
Cypcar_3        QIDSYDYVFR--CNFAP-TEVFRRDVGRRTNLTTFN-PSILEKYYNNLLT
Poefor3         QIEKYDFVLR--CNFAP-TEIFKKDVGRRTNMTTFN-PSILEKYYNNLLT
Poelat_3        QIEKYDFVLR--CNFAP-TEIFKKDVGRRTNMTTFN-PSILEKYYNNLLT
```

```
Poeret_3       QIEKYDFVLR--CNFAP-TEIFKKDVGRRTNMTTFN-PSILEKYYNNLLT
Angang_3       EIDRSDFVFR--CNFAP-TEIFHRDVGRRTNLTTFN-PSILEKYYNNLLT
Xipmac_3       QIEKYDFVLR--CNFAP-TEIFKKDVGRRTNMTTFN-PSILEKYYNNLLT
Masarm_3       QIEKFDFVFR--CNFAP-TEIFKKDVGRRTNMTTFN-PSILEKYYNNLLT
Permag_3       QIDSFDFVFR--CNFAP-TEVFKKDVGRRTNMTTFN-PSILEKYYNNLLT
Gnapet3        QIDKSDFVFR--CNFAP-TEVFHKDVGHKTNLTTFN-PSILEKYYNNLLT
Ostbic_3       RIDSSDFVFR--CNFAP-TELFRKDVGRKTNLTTFN-PSILEKYYNNLLT
Gadmor_3       QIEKFDYVFR--CNFAP-TEIFKKDVGRHTNMTTFN-PSILEKYYNNLLT
Sclfor_3       RIDGSDFVFR--CNFAP-TELFHRDVGRKTNLSTFN-PSILEKYYNNLLT
Acapol3        QIEKFDFVFR--CNFAP-TEIFKKDVGRRTNMTTFN-PSILEKYYNNLLT
Ampoce_3       QIEKFDFVFR--CNFAP-TEIFKKDVGRRTNMTTFN-PSILEKYYNNLLT
Sclfor_3b      EIDRFDFVFR--CNFAP-TELFHKDVGHKTNITTFN-PSILEKYYNNLLT
Ostbic_3b      EIDRFDFVFR--CNFAP-TELFHKDVGHKTNITTFN-PSILEKYYNNLLT
Parkin_3       QIDESDFVFR--CNFAP-TEVFHKDVGHKTNLTTFN-PSILEKYYNNLLT
Ampper3        QIEKFDFVFR--CNFAP-TEIFKKDVGRRTNMTTFN-PSILEKYYNNLLT
Oncmyk_3       EIEKFDFVFR--CNFAP-TEIFHRDVGRRTNLTTFN-PSILEKYYNNLLT
Gasacu_3       QIEKFDFVFR--CNFAP-TEIFKKDVGRRTNMTTFN-PSILEKYYNNLLT
Labber_3       QIEKFDYVFR--CNFAP-TEVFKKDVGRQTNMTTFN-PSILEKYYNNLLT
Panbuc_3       EIDRYDFVFR--CNFAP-TEAFRKDVGRKTNLTTFN-PSILEKYYNNLLT
Parhas3        AIDKYDFVFR--CNFAP-TEAFRKDVGRRTNLTTFN-PSILEKYYNNLLT
Cluhar_3       EIDKFDFVFR--CNFAP-TEVFRKDVGRHTNLTTFN-PSILEKYYNNLLT
Aloalo_3       EIDKFDFVFR--CNFAP-TEVFRKDVGRHTNLTTFN-PSILEKYYNNLLT
Panhyp_3       AIDAYDFVFR--CNFAP-TEVFRKDVGRRTNLTTFN-PSILEKYYNNLLT
Eleele_3       TIDQYDFVFR--CNFAP-TEVFRKDVGRRTNLTTFN-PSILEKYYNNLLT
Denclu_3       DIDKYDFIFR--CNFAP-TELFRKDVGRRTNLTTFN-PSILEKYYNNLLT
Sarpil_3       EIDKFDYVFR--CNFAP-TEVFRKDVGRRTNLTTFN-PSILEKYYNNLLT
Eigvir_3       AIDKYDFVFR--CNFAP-TEVFRKDVGRRTNLTTFN-PSILEKYYNNLLT
Aptalb_3       AIDRYDFVFR--CNFAP-TEAFRKDVGRRTNLTTFN-PSILEKYYNNLLT
Sinans_3       QIDSYDFVFR--CNFAP-TEVFRRDVGRRTNLTTFN-PSILEKYYNNLLT
Pygnat_3       TIDKYDFVFR--CNFAP-TEVFRRDVGRRTNLTTFN-PSILEKYYNNLLT
Astmex_3       TIDKYDFVFR--CNFAP-TEVFRRDVGRRTNLTTFN-PSILEKYYNNLLT
Caraur_3       QIDSYDFVFR--CNFAP-TEIFRRDVGRRTNLTTFN-PSILEKYYNNLLT
Danrer_3       EIDKYDFVFR--CNFAP-TEVFRRDVGRRTNLTTFN-PSILEKYYNNLLT
Ictpun_3       AIDAYDFVFR--CNFAP-TEVFRRDVGRRTNLTTFN-PSILEKYYNNLLT
Plealt_3       EIEKFDFVFR--CNFAP-TEIFRRDVGRRTNMTTFN-PSILEKYYNNLLT
Plealt_9       EIDKADFIFR--CNFAP-TEVFYKDVGRKTNMTTFN-PSILEHYYNNLLT
Gasacu_9       EIDQADFVFR--CNFAP-TEVYSKDVGKKTNLTTFN-PSILERYYNNLLT
Parkin_9       EIDKFNFIFR--CNFAP-TEIYHRDVGRKTNMTTFN-PSILERYYNNLLT
Acapol_9       EIDEADFVFR--CNFAP-TEVYSKDVGKKTNLTTFN-PSILERYYNNLLT
Larcro_9       EIDQADFVFR--CNFAP-TEVYSKDVGRKTNLTTFN-PSILERYYNNLLT
Notcor_9       EIDQADFVFR--CNFAP-TEIYSKDVGKKTNLTTFN-PSILERYYNNLLT
Latcal_9       EIDQADFVFR--CNFAP-TEVYSKDVGRKTNLTTFN-PSILERYYNNLLT
Serlaldor_9    EIDQADFVFR--CNFAP-TEVYSKDVGRKTNLTTFN-PSILERYYNNLLT
Monalb_9       EIDQADFVFR--CNFAP-TEVYFKDVGRKTNLTTFN-PSILERYYNNLLT
Serdum_9       EIDQADFVFR--CNFAP-TEVYSKDVGRKTNLTTFN-PSILERYYNNLLT
Perflu_9       EIDQADFVFR--CNFAP-TEFYSKDVGKKTNLTTFN-PSILERYYNNLLT
Perfla_9       EIDQADFVFR--CNFAP-TEFYSKDVGKKTNLTTFN-PSILERYYNNLLT
Stepar_9       EIDQADFVFR--CNFAP-TEVYSKDVGKKTNLTTFN-PSILERYYNNLLT
Molmol_9       EIDQADFVFR--CNFAP-TDIYSKDVGRKTNLTTFN-PSILERYYNNLLT
Hipcom_9       EIDRADFVFR--CNFAP-TEVYAKDVGRKTNLTTFN-PSILERYYNNLLT
Takrub_9 B     NIDQADFVFR--CNFAP-TEVYSKDVGRKTNMTTFN-PSILERYYNNLLT
Takrub_9       NIDQADFVFR--CNFAP-TEVYSKDVGRKTNMTTFN-PSILERYYNNLLT
Cynsem_9       EIDQADFVFR--CNFAP-TEVYSKDVGKKTNLTTFN-PSILERYYNNLLT
Scomax_9       EIDQADFVFR--CNFAP-TEVYSKDVGRKTNLTTFN-PSILERYYNNLLT
Corlav_9       EIDSADFIFR--CNFAP-TDSYYKDVGRKTNLTTFN-PSILERYYNNLLT
Oncmyk_9       EIDSADFIFR--CNFAP-TDSYYKDVGRKTNLTTFN-PSILERYYNNLLT
Salsal_9       EIDSSDFIFR--CNFAP-TDSYYKDVGRKTNLTTFN-PSILERYYNNLLT
Onctsh_9       EIDSADFIFR--CNFAP-TDSYYKDVGRKTNLTTFN-PSILERYYNNLLT
Salalp_9       EIDSADFIFR--CNFAP-TDSYYKDVGRKTNLTTFN-PSILERYYNNLLT
Ampoce_9       EIDEADFVFR--CNFAP-TEVYSKDVGKKTNLTTFN-PSILERYYNNLLT
Ampper9        EIDEADFVFR--CNFAP-TEVYSKDVGKKTNLTTFN-PSILERYYNNLLT
Labber_9       EIDQADFVFR--CNFAP-TDVYSKDVGKKTNLTTFN-PSILERYYNNLLT
Umbpyg_3B      EIEKFDFVFR--CNFAP-TEIFRRDVGRRTNLTTFN-PSILEKYYNNLLT
Esoluc_3b      EIEKFDFVFR--CNFAP-TEVFRRDVGRRTNLTTFN-PSILEKYYNNLLT
Gadmor_9       EIDQADFVFR--CNFAP-TEVYYKDVGRKTNMTTFN-PSILERYYNNLLT
Umbpyg_9       EIDSADFIFR--CNFAP-IDSYYKDVGRKTNLTTFN-PSILERYYNNLLT
Esoluc_9       EIDSADFIFR--CNFAP-IESYYKDVGRKTNLTTFN-PSILERYYNNLLT
Permag_9       EIDQADFVFR--CNFAP-TEVYSKDVGKKTNLTTFN-PSILERYYNNLLT
Bolpec_9       EIDQADFVFR--CNFAP-TEVYSKDVGKKTNLTTFN-PSILERYYNNLLT
Poefor9        EIDQADFVFR--CNFAP-TEVYFKDVGKKTNLTTFN-PSILERYYNNLLT
Masarm_9       EIDQADFVFR--CNFAP-TEVYSKDVGKKTNLTTFN-PSILERYYNNLLT
Angjap_9       EIDRSDFVFR--CNFAP-TEVFHRDVGRRTNLTTFN-PSILEKYYNNLLT
Poelat_9       EIDQADFVFR--CNFAP-TEVYFKDVGKKTNLTTFN-PSILERYYNNLLT
Poeret_9       VIDQADFVFR--CNFAP-TEFYFKDVGKKTNLTTFN-PSILERYYNNLLT
Xipmac_9       EIDQADFVFR--CNFAP-TEIYFKDVGKKTNLTTFN-PSILERYYNNLLT
Hapbur_9       EIDQADFVFR--CNFAP-TDIYSKDVGKKTNLTTFN-PSILERYYNNLLT
Orylat_9       EIDQADFVFR--CNFAP-TDVYSKDVGRKTNMTTFN-PSILERYYNNLLT
```

```
Gamaff_9       EIDQADFVFR--CNFAP-TEVYFKDVGKKTNLTTFN-PSILERYYNNLLT
Neobri_9       EIDQADFVFR--CNFAP-TDIYSKDVGKKTNLTTFN-PSILERYYNNLLT
Funhet_9       EIDQADFVFR--CNFAP-TEVYSKDVGKKTNLTTFN-PSILERYYNNLLT
Punnye_9       EIDQADFVFR--CNFAP-TDIYSKDVGKKTNLTTFN-PSILERYYNNLLT
Cypvar_9       EIDQADFVFR--CNFAP-TDVYSKDVGKKTNLTTFN-PSILERYYNNLLT
Krymar_9       EIDQADFVFR--CNFAP-TEVYSKDVGKKTNLTTFN-PSILERYYNNLLT
Notfur_9       EIDQADFVFR--CNFAP-TEIYSKDVGKKTNMTTFN-PSILERYYNNLLT
Notpie_9       EIDQADFVFR--CNFAP-TEIYSKDVGKKTNMTTFN-PSILERYYNNLLT
Orenil_9       EIDQADFVFR--CNFAP-TDIYSKDVGKKTNLTTFN-PSILERYYNNLLT
Auslim_9       EIDQADFVFR--CNFAP-TEIYSKDVGRKTNLTTFN-PSILERYYNNLLT
Anates_9       EIDQADFVFR--CNFAP-TDVYSKDVGKKTNLTTFN-PSILERYYNNLLT
Astcal_9       EIDQADFVFR--CNFAP-TDIYSKDVGKKTNLTTFN-PSILERYYNNLLT
Mayzeb_9       EIDQADFVFR--CNFAP-TDIYSKDVGKKTNLTTFN-PSILERYYNNLLT
Orymel_9       EIDQADFVFR--CNFAP-TDVYSKDVGRKTNMTTFN-PSILERYYNNLLT
Ampcit9        EIDQADFVFR--CNFAP-TDIYSKDVGKKTNLTTFN-PSILERYYNNLLT
Macfas_2       EIDAHSFVIR--CNLAP-VQEYARDVGLKTDLVTMN-PSVIQRAFEDLVN
Macmul_2       EIDAHSFVIR--CNLAP-VQEYARDVGLKTDLVTMN-PSVIQRAFEDLVN
Macnem_2       EIDAHSFVIR--CNLAP-VQEYARDVGLKTDLVTMN-PSVIQRAFEDLVN
Musmus_2       EIDTHSFVIR--CNRAP-VQEYARDVGLKTDLVTMN-PSVIQRAFEDLVN
Ponabe_2       EIDAHSFVIR--CNLAP-VQEYARDVGLKTDLVTMN-PSVIQRAFEDLVN
Papanu_2       EIDAHSFVIR--CNLAP-VQEYARDVGLKTDLVTMN-PSVIQRAFEDLVN
Nomleu_2       EIDAHSFVIR--CNLAP-VQEYARDVGLKTDLVTMN-PSVIQRAFEDLVN
Pantro_2       EIDAHSFVIR--CNLAP-VQEYARDVGLKTDLVTMN-PSVIQRAFEDLVN
Latcha_2       EIDDHQFVIR--CNLAP-VQEYASDVGTKTDFVTMN-PSVIQRAFEDLVN
Gnapet2        EIDSHDFVIR--CNLAP-VEEYERDVGLRSSLVTMN-PSVVQRAFRDLAS
Panbuc_2b      EIDACDYVIR--CNLAP-VQEYVTDVGQRTSMVTMN-PSVVQRAFQDLAS
Ostbic_2       EIDSNDFVIR--CNLAP-VDEYEQDVGRRTSLVTMN-PSVVQRAFQDLAS
Sinrhi2        EIDSHDFVIR--CNLAP-VEEYATDVGLRTSLVTMN-PSVVQRAFQDLNS
Hetzeb_2       EIDAHEFVIR--CNLAP-VEEYAHDVGLKTDLVTMN-PSVVQRAFEDLKN
Hipcom_2       QIDSHDFVIR--CNLAP-VEEYADDVGHRTDLVTMN-PSVVQRAFRDLVD
Prigla_2       EIDTHEFVIR--CNLAP-VEEYAQDVGLKTDLVTMN-PSVVQRAFEDLKN
Scytor_2       EIDTHEFVIR--CNLAP-VEEYAQDVGLKTDLVTMN-PSVVQRAFEDLKN
Tetcal_2       EIDAHEFVIR--CNLAP-VEEYVQDVGMKTDLVTMN-PSVVQRAFEDLKN
Rhityp_2       EIDDHQFVIR--CNLAP-VEEYAQDVGLKTDLVTMN-PSVVQRAFEDLKN
Parkin_2b      EIDSHDFVIR--CNLAP-VQEFELDVGLRTGLVTMN-PSVVQRAFQDLGS
Angang_2       QIDSHDFVIR--CNLAP-VEEYAADVGQRTSLVTMN-PSVVERAFRDLSS
Pygnat_2       EIDSHDFVIR--CNLAP-VEEYTNDVGLRTSLVTMN-PSVVQRAFQDLNS
Parkin_2       EIDSHDFVIR--CNLAP-VEEYEHDVGLRSSLVTMN-PSVVQRAFRDLAS
Gorgor_2       EIDAHSFVIR--CNLAP-VQEYARDVGPKTDLVTMN-PSVIQRAFEDLVN
Thegel_2       EIDAHSFVIR--CNLAP-VQEYARDVGLKTDLVTMN-PSVIQRAFEDLVN
Tupchi_2       EIDTHSFVIR--CNLAP-VQEYAQDVGLKTDLVTMN-PSVIQRAFEDLVN
Galgal_2       EIDTHSFVIR--CNLAP-VQEYSQDVGTKTDLVTMN-PSVIQRAFEDLMN
Eleele_2       EIDSHDFIIR--CNLAP-IEHYVKDVGVRTGLVTMN-PSVVERVFQDLSS
Danrer_2       EIDSHDFVIR--CNLAP-VEEYAADVGLRTSLVTMN-PSVVQRAFQDLNS
Canlupfam_2    EIDTHSFVIR--CNLAP-VQEYGRDVGLKTDLVTMN-PSVIQRAFEDLVN
Aloalo_2       EIDSYDFVIR--CNLAP-VDEFTADVGHRTNLVTMN-PSVVQRAFQDLSS
Astmex_2       EIDSHDFVIR--CNLAP-VEEYAEDVGLRSSLVTMN-PSVVQRAFQDLSS
Sinans_2       EIDSHDFVIR--CNLAP-VEEYATDVGLRTSLVTMN-PSVVQRAFQDLNS
Sclfor_2       EIDSNDFVIR--CNLAP-VEEYEQDVGHRTSLVTMN-PSVVQRAFQDLAS
Ceraty_2       EIDAHSFVIR--CNLAP-VQEYARDVGLKTDLVTMN-PSVIQRAFEDLVN
Anocar_2       EIDAHSFVIR--CNLAP-VQEYSRDVGTKMDLVTMN-PSVIQRAFEDLVN
Anates_2       EIDSHDFIIR--CNLAP-VEEYSRDVGWQTNLVTMN-PSVVQRAFQDLVS
Ampbic_2       EIDSHDFVIR--CNLAP-VEDYSRDVGRQTDLVTMN-PSVVQRAFQDLVS
Sarpil_2       EIDSYDFVIR--CNLAP-VDEFSADVGHRTSLVTMN-PSVVQRAFQDLSS
Xenlae_2       EIDSHDFVIR--CNLAP-VEEYAKDVGTKTNLVTMN-PSVVQRAFEDLVN
Cluhar_2       EIDAHDFVIR--CNLAP-VNEFSADVGHRTNLVTMN-PSVVQRAFQDLSS
Cypcar_2       EIDSHDFVIR--CNLAP-VEEYATDVGLRTSLVTMN-PSVVQRAFQDLNS
Denclu_2       EIDSHDFVIR--CNLAP-VEEYAADVGHRTDLVTMN-PSVVQRAFQDLAN
Notpie_2       EIDSHDFVIR--CNLAP-VEEYSQDVGWRTNLVTMN-PSVVQRAFQDLVT
Erpcal2        EIDTHEFVIR--CNLAP-VQEFASDVGIRTNLVTMN-PSVVQRAFEDLAN
Amical_2       EIDSHDFVIR--CNLAP-VEEYADDVGLRTSLVTMN-PSVVQRAFQDLVS
Lepocu_2       EIDSHDFVIR--CNLAP-VEEYRADVGRRTSLVTMN-PSVVQRAFQDLAS
Homsap_2       EIDAHSFVIR--CNLAP-VQEYARDVGLKTDLVTMN-PSVIQRAFEDLVN
Bostau_2       EIDTHSFVIR--CNLAP-VQEYARDVGLKTDLVTMN-PSVVQRAFEDLVN
Caraur_2       EIDSHDFVIR--CNLAP-VEEYATDVGLRTSLVTMN-PSVVQRAFQDLNS
Thythy_2B      EIDSHDFVIR--CNLAP-VEEYAGDVGRHTNLVTMN-PSVVQRAFQDLAS
Onctsh_2B      EIDSYDFVIR--CNLAP-VEEYAGDVGRRTNLVTMN-PSVVQRAFQDLAS
Salsal_2B      EIDSYDFVIR--CNLAP-VEEYAGDVGRRTNLVTMN-PSVVQRAFQDLAS
Oncmyk_2B      EIDSYDFVIR--CNLAP-VEEYAGDVGRRTNLVTMN-PSVVQRAFQDLAS
Onckis_2B      EIDSYDFVIR--CNLAP-VEEYAGDVGRRTNLVTMN-PSVVQRAFQDLAS
Salsal_2A      EIDSHDFVIR--CNLAP-VEEYAGDVGQRTNLVTMN-PSVVQRAFHDLAS
Thythy_2A      EIDSHDFVIR--CNLAP-VEEYAGDVGRRTNLVTMN-PSVVQRAFHDLAS
Salalp_2A      EIDSHDFVIR--CNLAP-VEEYAGDVGRRTNLVTMN-PSVVQRAFHDLAS
Corlav_2A      EIDSHDFVIR--CNLAP-VEEYAGDVGRRTNLVTMN-PSVVQRAFHDLAS
Cormar_2A      EIDSHDFVIR--CNLAP-VEEYAGDVGRRTNLVTMN-PSVVQRAFHDLAS
Onctsh_2A      EIDSHDFVIR--CNLAP-VEEYAGDVGRRTNLVTMN-PSVVQRAFHDLAS
Oncmyk_2A      EIDSHDFVIR--CNLAP-VEEYAGDVGRRTNLVTMN-PSVVQRAFHDLAS
```

```
Neobri_2       EIDSHDFVIR--CNLAP-VEEYFQDVGWRTNLVTMN-PSVVQRAFQDLVT
Takrub_2       EIDSHDFVIR--CNLAP-VEDYYKDVGWRTNLVTMN-PSVVQRAFRDLAS
Tetnig_2       EIDSHDFVIR--CNLAP-VEDFHNDVGWRTNLVTMN-PSVVQRAFRDLAS
Orenil_2       EIDSHDFVIR--CNLAP-VEEYFQDVGWRTNLVTMN-PSVVQRAFQDLVT
Orylat_2       EIDAHDFVIR--CNLAP-VDEYSQDVGRQTNLVTMN-PSVVQRAFQDLVS
Krymar_2       EIDSHDFVIR--CNLAP-VEEYSRDVGRRTDLVTMN-PSVVQRAFQDLLT
Notfur_2       EIDSHDFVIR--CNLAP-VEEYSQDVGWRTNLVTMN-PSVVQRAFQDLVT
Poelat_2       EIDSHDFVIR--CNLAP-VEEYSQDVGRRTNLVTMN-PSVVQRAFQDLVS
Gadmor_2       EIDSYDFVIR--CNLAP-VKEYSVDVGRRTNLVTMN-PSVVQRAFQDLAS
Xipmac_2       EIDSHDFVIR--CNLAP-VEEYSQDVGRRTNLVTMN-PSVVQRAFQDLVS
Poeret_2       EIDSHDFVIR--CNLAP-VEEYSQDVGRRTNLVTMN-PSVVQRAFQDLVS
Astcal_2       EIDSHDFVIR--CNLAP-VEEYFQDVGWRTNLVTMN-PSVVQRAFQDLVT
Hapbur_2       EIDSHDFVIR--CNLAP-VEEYFQDVGWRTNLVTMN-PSVVQRAFQDLVT
Acapol_2B      EIDSHDFVIR--CNLAP-VDDYSRDVGRRTNLVTMN-PSVVQRAFQDLVS
Ampoce_2A      EIDSHDFVIR--CNLAP-VEDYSRDVGRQTNLVTMN-PSVVQRAFQDLVN
Acapol_2A      EIDSHDFVIR--CNLAP-VDDYSRDVGRRTNLVTMN-PSVVQRAFQDLVS
Mayzeb_2       EIDSHDFVIR--CNLAP-VEEYFQDVGWRTNLVTMN-PSVVQRAFQDLVT
Punnye_2       EIDSHDFVIR--CNLAP-VEEYFQDVGWRTNLVTMN-PSVVQRAFQDLVT
Latcal_2       EIDSHDFVIR--CNLAP-VEEYSRDVGWRTNLVTMN-PSVVQRAFQDLVS
Singra_2       EIDSHDFVIR--CNLAP-VEEYATDVGLRTSLVTMN-PSVVQRAFQDLNS
Auslim_2       EIDSHDFVIR--CNLAP-VEEYSEDVGRRTNLVTMN-PSVVQRAFQDLVT
Monalb2        EIDSHDFVIR--CNLAP-VEEYSRDVGRRTNLVTMN-PSVVQRAFQDLVT
Ampoce_2B      EIDSHDFVIR--CNLAP-VEDYSRDVGRQTNLVTMN-PSVVQRAFQDLVN
Chiham_2       DIDSHDFVIR--CNLAP-VDEFSQDVGRRTNLVTMN-PSVVQRAFQDLVS
Gasacu_2       DIDSHDFVIR--CNLAP-VEDYSLDVGRRTNLVTMN-PSVVQRAFQDLVS
Gymacu_2       DIDSHDFVIR--CNLAP-VDEFSQDVGRRTNLVTMN-PSVVQRAFQDLVS
Labber_2       EIDSHDFVIR--CNLAP-VADYYQDVGWRTNLVTMN-PSVVQRAFQDLVS
Cypvar_2       EIDSHDFVIR--CNLAP-VEEYSQDVGLRTNLVTMN-PSVVQRAFQDLIS
Funhet_2       EIDSHDFVIR--CNLAP-VEEYSQDVGRRTNLVTMN-PSVVQRAFQDLVS
Larcro_2       EIDSHDFVIR--CNLAP-VEEYYRDVGWRTNLVTMN-PSVVQRAFQDLVS
Paroli_2       EIDSHDFVIR--CNLAP-VEEFSWDVGRRTNLVTMN-PSVVQRAFQDLVS
Perflu_2       DIDSHDFVIR--CNLAP-VEEYFQDVGWRTNLVTMN-PSVVQRAFQDLVS
Serdum_2       EIDSHDFVIR--CNLAP-VEEYSRDVGRRTNLVTMN-PSVVQRAFMDLVS
Stepar_2       EIDSHDFVIR--CNLAP-VEDYSRDVGRRTNLVTMN-PSVVQRAFQDLVS
Notcor_2       DIDSHDFVIR--CNLAP-VDEFSQDVGRRTNLVTMN-PSVVQRAFQDLVS
Treber_2       DIDSHDFVIR--CNLAP-VDEFSQDVGRRTNLVTMN-PSVVQRAFQDLVS
Serlaldor_2    EIDSHDFVIR--CNLAP-VEEYSRDVGRRTNLVTMN-PSVVQRAFMDLVS
Plaste_2       EIDSHDFVIR--CNLAP-VEEYSWDVGTRTSLVTMN-PSVVQRAFQDLVS
Clabat_4       EIDIHDFVIR--CNLAP-LAEFAEDVGLRSDFTTMN-PSVIQRVYGGLSN
Ictpun_4A      EIDSHDFVIR--CNLAP-LAEFADDVGLRSDFTTMN-PSVIQRVYGGLLN
Panhyp_4       EIDSHDFVIR--CNLAP-LAEFAEDVGLRSDFTTMN-PSVIQRVYGGLLN
Umbpyg_4       EIDSHDFVIR--CNLAP-LAEFSEDVGLRSDFTTMN-PSVIQRAYGGLKN
Esoluc_4       EIDSHDFVIR--CNLAP-LAQFAEDVGLRSDFTTMN-PSVIQRAYGGLRN
Plealt_4       EIDSHDFVIR--CNLAP-LSEYADDVGVRSDFTTMN-PSVIQRAYGGLKN
Onckis_4B      EIDSHDFVIR--CNLPP-LSEFAEDVGLRSDFTTMN-PSVIQRAYGGLKN
Onckis_4A      EIDSHDFVIR--CNLPP-LSEFAEDVGLRSDFTTMN-PSVIQRAYGGLKN
Onctsh_4       EIDSHDFVIR--CNLPP-LSEFAEDVGLRSDFTTMN-PSVIQRAYGGLKN
Oncmyk_4       EIDSHDFVIR--CNLPP-LSEFAEDVGLRSDFTTMN-PSVIQRAYGGLKN
Corlav_4       EIDRHDFVIR--CNLAP-LAEFAEDVGLRSDFTTMN-PSVIQRVYGGLKN
Astmex_4A      EIDGHDFVIR--CNLAP-LAEFAEDVGLKSDFTTMN-PSVIQRVYGGLRN
Pygnat_4       EIDSHDFVIR--CNLAP-LVEFAEDVGLKSDFTTMN-PSVIQRVYGGLRN
Aloalo_4       EIDSHDFVIR--CNLAP-LAEFAADVGLRSDFTTMN-PSVIQRAYGGLRN
Cluhar_4       EIDSHDFVIR--CNLAP-QAEFAADVGLRSDFTTMN-PSVIQRAYGGLRN
Denclu_4       EIDSHDFIIR--CNLAP-LKEFAEDVGLRSDFTTMN-PSVIHRVYGGLQK
Konpun4        EIDSHDFVIR--CNLAP-LVEFAADVGLRSDFTTMN-PSVIQRAYSGLRN
Sarpil_4       EIDSHDFVIR--CNLAP-LAEFADDVGLRSDFTTMN-PSVIQRAYGGLRN
Caraur_4       EIDSHGFVIR--CNLAP-LVEFADDVGLRSDFTTMN-PSVIPRVYGALRD
Cteide_4       EIDNHDFVIR--CNLAP-LKDFADDVGLRSDFTTMN-PSVIQRVYKGLRD
Petmar_4       EIDQHDFVIR--CNLAP-VEGYERDVGSRVDFVTMN-PSVVERTYGGLRT
Cypcar_4       EIDSHGFVIR--CNLAP-LEEFADDVGLRSDFTTMN-PSVIPRVYGGLQD
Danrer_4       EIDNHSFVIR--CNLAP-LEGFADDVGLRSDFTTMN-PSVIQRVYGGLRE
Misang_4       EIDNHDFVIR--CNLAP-LEEFSDDVGLKSDFTTMN-PSVIQRVYGGLHN
Angjap_4       EIDGHDFVIR--CNLAP-LEEFAEDVGLRSDFTTMN-PSVIQRAYGGLRN
Cypcar_4A      EIDSHGFVIR--CNLAP-LEEFADDVGLRSDFTTMN-PSVIPRVYGGLQD
Angang_4       EIDGHDFVIR--CNLAP-LEEFAEDVGLRSDFTTMN-PSVIQRAYGGLRN
Cypcar_4B      EIDSHGFVIR--CNLAP-LEDFADDVGLRSDFTTMN-PSVIPRVYGGLRD
Sinans_4A      EIDSHGFVIR--CNLAP-LEEFADDVGLRSDFTTMN-PSVIPRVFGGLRD
Sinans_4B      EIDSHGFVIR--CNLAP-LEEFADDVGSRSDFTTMN-PSVIPRVFGGLRD
Singra_4A      EIDSHGFVIR--CNLAP-LEEFADDVGLRSDFTTMN-PSVIPRVFGGLQD
Aptalb_4       EIDSHDFVIR--CNLAP-LAEFIKDVGLKSDFTTMN-PSVIQRMYGGLRN
Eigvir_4       EIDDHDFVIR--CNLAP-LAEFIKDVGWKSDFTTMN-PSVIQRVYHGLRN
Eleele_4       EIDSHDFVIR--CNLAP-LAKFAEDVGLKSDFTTMN-PSVIQRMYGGLRN
Parhas_4       EIDSHDFVIR--CNLAP-LAEFIKDVGLKSDFTTMN-PSVIQRMYGGLRN
Sclfor_4       EIDNHNFVIR--CNLAP-LVEFSEDVGLQSDFTTMN-PSVIQRAFGGLKN
Gnapet4        EIDSNDFVIR--CNLAP-LANFSEDVGLRSDFTTMN-PSVIQRAFGGLRN
Ostbic_4       EIDSHDFVIR--CNLAP-LVEFSEDVGLRSDFTTMN-PSVIQRVFGSLKN
Parkin_4       EIDSNDFVIR--CNLAP-LANFSEDVGLRSDFTTMN-PSVIQRAFGGLRN
```

```
Panbuc_4       EIDSHDFIIR--CNLAP-VTEFAVDVGQRSHFITMN-PSVIQRAYGSLRN
Erpcal4        EIDNHDFVIR--CNLAP-VVEFAEDVGNRSDFITMN-PSVIQRAFGGFQN
Salsal_4       EIDSHDFVIR--CNLPP-LSEFAEDVGLRSDFTTMN-PSVIQRAYGGLKN
Saltru_4       EIDSHDFVIR--CNLPP-LSEFAEDVGLRSDFTTMN-PSVIQRAYGGLKN
Salalp_4       EIDSHDFVIR--CNLPP-LSEFAEDVGLRSDFTTMN-PSVIQRVYGGLKN
Salfon_4       EIDSHDFVIR--CNLPP-LSEFAEDVGLRSDFTTMN-PSVIQRAYGGLKN
Thythy_4       EIDSHDFVIR--CNLAP-LADFAEDVGLRSDFTTMN-PSVIQRAYGGLKN
Calmil_4       EIDSHEFVIR--CNLAP-LVEYTDDVGSKSDFVTMN-PSVVQRAFGSLQN
Hetzeb_4       EIDSHEFVIR--CNLAP-LMEYTDDVGLKSDFVTMN-PSVVQRAFGSLRS
Rhityp_4       EIDSHEFVIR--CNLAP-LVEYADDVGLKSDFVTMN-PSVVQRAFGSLRS
Scytor_4       EIDSHEFVIR--CNLAP-LVEYADDVGLKSDFVTMN-PSVVQRAFGSLRS
Squaca_4       EIDSHEFVIR--CNLAP-LVEYADDVGLKSDFVTMN-PSVVQRAFGSLQS
Amical_4       EIDSHDFVIR--CNLAP-LAEFSEDVGLRSDFITMN-PSVIQRAFGGFKN
Lepocu_4       EIDNHDFVIR--CNLAP-LAEFSEDVGLRSDFITMN-PSVIQRAFGGFKN
Agema_4        EIDSHDFVIRR-CNLAP-LSEFAEDVGLRSDFTTMN-PSVIQRVYSGLLN
Anocar_4       EIDSHEFVIR--CNLAP-VVEFAADVGTKSDFITMN-PSVVQRAFGGFRN
Canlupfam_4    EIDSHNFVIR--CNLAP-VVEFAADVGTKSDFITMN-PSVVQRAFGGFRN
Crigri_4       EIDSHNFVIR--CNLAP-VVEFAADVGTKSDFITMN-PSVVQRAFGGFRN
Galgal_4       EIDTHDFVIR--CNLAP-VVEFAADVGNKSDFITMN-PSVVQRAFGGFRN
Mesaur_4       EIDSHNFVIR--CNLAP-VVEFAADVGTKSDFITMN-PSVVQRAFGGFRN
Homsap_4       EIDSHNFVIR--CNLAP-VVEFAADVGTKSDFITMN-PSVVQRAFGGFRN
Notscuscu_4    EIDSHEFVIR--CNLAP-VVEFAADVGTKSDFITMN-PSVVQRAFGGFRN
Psetextex_4    EIDSHEFVIR--CNLAP-VVEFAADVGTKSDFITMN-PSVVQRAFGGFRN
Musmus_4       EIDSHNFVIR--CNLAP-VVEFAADVGTKSDFITMN-PSVVQRAFGGFRN
Pogvit_4       EIDNHEFVIR--CNLAP-VVEFAADVGTKSDFITMN-PSVVQRAFGGFRN
Promuc_4       EIDNHEFVIR--CNLAP-VVEFAADVGTKSDFITMN-PSVVQKAFGGFRN
Pantro_4       EIDSHNFVIR--CNLAP-VVEFAADVGTKSDFITMN-PSVVQRAFGGFRN
Pytbiv_4       EIDNHEFVIR--CNLAP-VVEFAADVGTKSDFITMN-PSVVQRAFGGFRN
Ratnor_2       EIDTHSFVIR--CNLAP-VQEYARDVGLKTDLVTMN-PSVIQRAFEDLVN
Ratnor_4       EIDSHNFVIR--CNLAP-VVEFAADVGTKSDFITMN-PSVVQRAFGGFRN
Siltro_2       EIDSHDFVIR--CNLAP-VEEYATDVGTKTNLVTMN-PSVVQRAFEDLVN
Siltro_4       EIDSHDFVIR--CNLAP-VVEFAAHVGTKSDFITMN-PSVVQRAFGGFRN
Susscr_4       EIDSHNFVIR--CNLAP-VVEFAADVGTKSDFITMN-PSVVQRAFGGFRN
Taegut_4       EIDSHDFVIR--CNLAP-VVEYAADVGTKSDFITMN-PSVVQRAFGGFRN
Termextri_4M   EIDNHDFVIR--CNLAP-VVEFAADVGTKSDFITMN-PSVVQRAFGGFRN
Thasirsir_4    EIDSHEFVIR--CNLAP-VVEFAADVGTKSDFITMN-PSVVQRAFGGFRN
Xenlae_4B      EIDSHDFVIR--CNLAP-VVEFAADVGTKSDFITMN-PSVVQRAFGGFRN
Xenlae_4A      EIDSHDFVIR--CNLAP-VVEFAADVGTKSDFITMN-PSVVQRAFGGFRN
               101
Latcal_3       VQDR-NNFFLSLKKLDGAILWIPAFFFHTSATVTRTLVDFFVEHRGQ---
Perflu_3       VQDR-NNFFLSLKKLDGAILWIPAFFFHTSATVTRTLVDFFVEHRGQ---
Serdum_3       VQDR-NNFFLSLKKLDGAILWIPAFFFHTSATVTRTLVDFFVEHRGQ---
Stepar_3       VQDR-NNFFLSLKKLDGAILWIPAFFFHTSATVTRTLVDFFVEHRGQ---
Tetcal_3       IQDR-NKFFLNLKRLDGAILWIPAFFFHTSAPVTRTLVDFFIEHRAQ---
Monalb_3       VQDR-NNFFLSLKKLDEAILWIPAFFFHTSATVTRTLVDFFVEHQGQ---
Molmol_3       VQDR-NNFFLSLKKLDGVILWIPAFFFHTSATVTRTLVDFFVEHRGQ---
Takrub_3       VQDR-NNFFLSLKKLDTTILWIPAFFFHTSATVTRTLVDFFVEHRGQ---
Canlupfam_3    IQDR-NNFFLSLKKLDGAILWIPAFFFHTSATVTRTLVDFFVEHRGQ---
Bostau_3       IQDR-NNFFLSLKKLDGAILWIPAFFFHTSATVTRTLVDFFVEHRGQ---
Homsap_3       IQDR-NNFFLSLKKLDGAILWIPAFFFHTSATVTRTLVDFFVEHRGQ---
Siltro_3       IQDR-NNFFLSLKKLDGAILWIPAFFFHTSASVTRTLVDFFVEHRDQ---
Anocar_3       IQDR-NNFFLSLKKLDGAILWIPAFFFHTSATITRTLVDFFIEHRAQ---
Musmus_3       IQDR-NNFFLSLKKLDGAILWIPAFFFHTSATVTRTLVDFFVEHRGQ---
Pantro_3       IQDR-NNFFLSLKKLDGAILWIPAFFFHTSATVTRTLVDFFVEHRGQ---
Ratnor_3       IQDR-NNFFLSLKKLDGAILWIPAFFFHTSATVTRTLVDFFVEHRGQ---
Galgal_3       IQDR-NNFFLSLKKLDGAILWIPAFFFHTSATVTRTLVDFFVEHRGQ---
Lepocu_3       IQDR-NNFFLSLKKLDGAILWIPAFFFHTSATVTRTLVDFFVEHKGQ---
Tetnig_3       VQDR-NNFFLSLKRLDPAILWIPAFFFHTSATVTRTLVDFFVEPRGQ---
Squaca_3       IQDR-NKFFLNLKKLDGAILWIPAFFFHTSAPVTRTLVDFFMEHRAQ---
Perfla_3       VQDR-NNFFLSLKKLDGAILWIPAFFFHTSATVTRTLVDFFVEHRGQ---
Salsal_3       IQDR-NNFFLSLKKLDRAILWIPAFFFHTSATVTRTLVDFFVEHRGQ---
Serlaldor_3    VQDR-NNFFLSLKKLDGAILWIPAFFFHTSATVTRTLVDFFVEHRGQ---
Treber_3       IQDR-NNFFLNLKKLEGAILWIPAFFLHTSATVTRTLVDFFVEHKGQ---
Cynsem_3       VQDR-NNFFLSLKKLDNAILWIPAFFFHTSATVTRTLVDFFVEHRGQ---
Paroli_3       IQDR-NNFFLNLKKLEGAILWIPAFFLHTSATVTRTLVDFFVEHKGQ---
Paroli_3b      VQDR-NNFFLSLKKLDNAILWIPAFFFHTSATVTRTLVDFFVEHRGQ---
Plaste_3       IQDR-NNFFLNLKKLESAILWIPAFFLHTSATVTRTLVDFFVEHKGQ---
Plaste_3b      VQDR-NNFFLSLKKLDSAILWIPAFFFHTSATVTRTLVDFFVEHRGQ---
Erpcal3        IQDR-NNFFLSLKKLDGAILWIPAFFFHTSATVTRTLVDFFVEHKGQ---
Calmil_3b      IQDR-NKFFLNLKKLDGAILWIPAFFFHSSALVTRTLVDFFVEHRRQ---
Hetzeb_3       IQDR-NKFFLNLKKLDGAILWIPAFFFHTSAPVTRTLVDFFIEHRAQ---
Latcha_3       IQDR-NNFFLSLKKLDGAILWIPAFFFHTSATVTRTLVDFFVEHKGQ---
Anates_3       VQDR-NNFFLSLKKLDGAILWIPAFFFHTSATVTRTLVDFFVEHRGQ---
Ampcit3        VQDR-NNFFLSLKKLDGVILWIPAFFFHTSATVTRTLVDFFVEHRDQ---
Auslim_3       VQDR-NNFFLSLKKLDGAILWIPAFFFHTSATVTRTLVDFFVEHRGQ---
Cypvar_3       VQDR-NNFFLSLKKLDRAILWIPAFFFHTSATVTRTLVDFFVEHRGQ---
Krymar_3       VQDR-NNFFLSLKKLDSAILWIPAFFFHTSATVTRTLVDFFVEHRGQ---
```

```
Funhet_3       VQDR-NNFFLSLKKLDRAILWIPAFFFHTSATVTRTLVDFFVEHRGQ---
Gamaff3        VQDR-NNFFLSLKKLDRAILWIPAFFFHTSATVTRTLVDFFVEHRGQ---
Orylat_3       VQDR-NNFFLSLKKLDGAILWIPAFFFHTSATVTRTLVDFFVEHRGQ---
Orenil_3       VQDR-NNFFLSLKKLDGAILWIPAFFFHTSATVTRTLVDFFVEHRGQ---
Mayzeb_3       VQDR-NNFFLSLKKLDGAILWIPAFFFHTSATVTRTLVDFFVEHKGQ---
Punnye_3       VQDR-NNFFLSLKKLDGAILWIPAFFFHTSATVTRTLVDFFVEHKGQ---
Punnye_3a      VQDR-NNFFLSLKKLDGAILWIPAFFFHTSATVTRTLVDFFVEHKGQ---
Neobri_3       VQDR-NNFFLSLKKLDGAILWIPAFFFHTSATVTRTLVDFFVEHKGQ---
Misang_3       IQDR-NNFFLSLKKLDGAILWIPAFFFHTSATVTRTLVDFFVEHKGE---
Sinrhi3        IQDR-NNFYLSLKKLDGAILWIPAFFFHTSATVTRTLVDFFVEHKGQ---
Singra_3       IQDR-NNFFLSLKKLDGAILWIPAFFFHTSATVTRTLVDFFVEHKGQ---
Hapbur_3       VQDR-NNFFLSLKKLDGAILWIPAFFFHTSATVTRTLVDFFVEHKGQ---
Astcal_3       VQDR-NNFFLSLKKLDGAILWIPAFFFHTSATVTRTLVDFFVEHKGQ---
Notfur_3       VQDR-NNFFLSLKKLDGAVLWIPAFFFHTSATVTRTLVDFFVEHRGQ---
Cypcar_3       IQDR-NNFYLSLKKLDGAILWIPAFFFHTSATVTRTLVDFFVEHKGQ---
Poefor3        VQDR-NNFFLSLKKLDRAILWIPAFFFHTSATVTRTLVDFFVEHRGQ---
Poelat_3       VQDR-NNFFLSLKKLDRAILWIPAFFFHTSATVTRTLVDFFVEHRGQ---
Poeret_3       VQDR-NNFFLSLKKLDTAILWIPAFFFHTSATVTRTLVDFFVEHRGQ---
Angang_3       IQDR-NNFFLSLKKLDGVVLWIPAFFFHTSATVTRTLVDFFVEHRGQ---
Xipmac_3       VQDR-NNFFLSLKKLDRAILWIPAFFFHTSATVTRTLVDFFVEHRGQ---
Masarm_3       VQDR-NNFFLSLKKLDGAILWIPAFFFHTSATVTRTLVDFFVEHRGQ---
Permag_3       VQDR-NNFFLSLKRLDGAILWIPAFFFHTSATVTRTLVDFFVEHRGK---
Gnapet3        IQDR-NNFFLSLKKLDSAILWIPAFFFHTSATVTRTLVDFFVEHKGQ---
Ostbic_3       IQDR-NNFFLSLKKLDDAILWIPAFFFHTSATVTRTLVDFFVEHKDQ---
Gadmor_3       VQDR-NNFFLSLKRLDGAILWIPAFFFHTSATVTRTLVDFFVEHRGQ---
Sclfor_3       IQDR-NNFFLSLKKLDDAILWIPAFFFHTSATVTRTLVDFFVEHKGQ---
Acapol3        VQDR-NNFFLSLKKLDGAILWIPAFFFHTSATVTRTLVDFFVEHRGQ---
Ampoce_3       VQDR-NNFFLSLKKLDGAILWIPAFFFHTSATVTRTLVDFFVEHRGQ---
Sclfor_3b      IQDR-NNFFLSLKKLDGAILWIPAFFFHTSATVTRTLVDFFVEHKGQ---
Ostbic_3b      IQDR-NNFFLSLKKLDGAILWIPAFFFHTSATVTRTLVDFFVEHKGQ---
Parkin_3       IQDR-NNFFLSLKKLDSAILWIPAFFFHTSATVTRTLVDFFVEHKGQ---
Ampper3        VQDR-NNFFLSLKKLDGAILWIPAFFFHTSATVTRTLVDFFVEHRGQ---
Oncmyk_3       LQDR-NNFFLSLKKLDGAILWIPAFFFHTSATVTRTLVDFFVEHRGQ---
Gasacu_3       VQDR-NNFFLSLKKLDGVILWIPAFFFHTSATVTRTLVDFFVEHRGQ---
Labber_3       VQDR-NNFFLSLKRLDGAILWIPAFFFHTSATVTRTLVDFFVEHRGQ---
Panbuc_3       IQDR-NNFFLSLKKLDGAVLWIPAFFFHTSATVTRTLVDFFVEHQGQ---
Parhas3        IQDR-NNFFLSLKKLDGAILWIPAFFFHTSATVTRTLVDFFVEHRGQ---
Cluhar_3       IQDR-NNFFLSLKKLDGAILWIPAFFFHTSATVTRTLVDFFVEHKGQ---
Aloalo_3       IQDR-NNFFLSLKKLDGAILWIPAFFFHTSATVTRTLVDFFVEHKGQ---
Panhyp_3       IQDR-NNFFLSLKKLDGAILWIPAFFFHTSATVTRTLVDFFVEHRGQ---
Eleele_3       IQDR-NNFFLSLKKLDGAILWIPAFFFHTSATVTRTLVDFFVEHRGQ---
Denclu_3       IQDR-NNFFLSLKKLDGAVLWIPAFFFHTSATVTRTLVDFFVEHKGQ---
Sarpil_3       IQDR-NNFFLSLKKLDGAILWIPAFFFHTSATVTRTLVDFFVEHKGQ---
Eigvir_3       IQDR-NNFFLSLKKLDGAILWIPAFFFHTSATVTRTLVDFFVEHQGQ---
Aptalb_3       IQDR-NNFFLSLKKLDGAILWIPAFFFHTSATVTRTLVDFFVEHRGQ---
Sinans_3       IQDR-NNFYLSLKKLDGAILWIPAFFFHTSATVTRTLVDFFVEHKGQ---
Pygnat_3       IQDR-NNFFLSLKKLDRAILWIPAFFFHTSATVTRTLVDFFVEHRGQ---
Astmex_3       IQDR-NNFFLSLKKLDRAVLWIPAFFFHTSATVTRTLVDFFVEHQGQ---
Caraur_3       IQDR-NNFYLSLKKLDGAILWIPAFFFHTSATVTRTLVDFFVEHKGQ---
Danrer_3       IQDR-NNFFLSLKKLDGAILWIPAFFFHTSATVTRTLVDFFVEHKGQ---
Ictpun_3       IQDR-NNFFLSLKKLDGAILWIPAFFFHTSATVTRTLVDFFVEHRGQ---
Plealt_3       IQDR-NNFFLSLKKLDGAILWIPAFFFHTSATVTRTLVDFFVEHRGQ---
Plealt_9       IQDR-NNFFLNLKKLEGAILWIPAFFLHTSATVTRTLVDFFVEHKGQ---
Gasacu_9       IQDR-NNFFLNLKKLEGAILWIPAFFLHTSATVTRTLVDFFVEHKGQ---
Parkin_9       IQDR-NNFLLRLKKLDGAILWIPAFFFHTSAPVTRTLIDFFIEHKDQ---
Acapol_9       IQDR-NNFFLNLKKLEGAILWIPAFFLHTSATVTRTLVDFFVEHKGQ---
Larcro_9       IQDR-NNFFLNLKKLEGAILWIPAFFLHTSATVTRTLVDFFVEHKGQ---
Notcor_9       IQDR-NNFFLNLKKLEGAILWIPAFFLHTSATVTRTLVDFFVEHKGQ---
Latcal_9       IQDR-NNFFLNLKKLEGAILWIPAFFLHTSATVTRTLVDFFVEHKGQ---
Serlaldor_9    IQDR-NNFFLNLKKLEGAILWIPAFFLHTSATVTRTLVDFFVEHKGQ---
Monalb_9       IQDR-NNFFLSLKKLEGAILWIPAFFLHTSATVTRTLVDFFVEHKGQ---
Serdum_9       IQDR-NNFFLNLKKLEGAILWIPAFFLHTSATVTRTLVDFFVEHKGQ---
Perflu_9       IQDR-NNFFLNLKKLEGAILWIPAFFLHTSATVTRTLVDFFVEHKKQ---
Perfla_9       IQDR-NNFFLNLKKLEGAILWIPAFFLHTSATVTRTLVDFFVEHKKQ---
Stepar_9       IQDR-NNFFLNLKKLEGAILWIPAFFLHTSATVTRTLVDFFVEHKGQ---
Molmol_9       IQDR-NNFFLNLKKLEGAILWIPAFFLHTSATVTRTLVDFFVEHKGQ---
Hipcom_9       IQDR-NNFFLHLKKLEGAILWIPAFFLHTSATVTRTLVDFFVEHKGQ---
Takrub_9 B     IQDR-NNFFLNLKKLGGAILWIPAFFLHTSATVTRTLVDFFVEHKGQ---
Takrub_9       IQDR-NNFFLNLKKLGGAILWIPAFFLHTSATVTRTLVDFFVEHKGQ---
Cynsem_9       IQDR-NNFFLNLKKLEGAILWIPAFFLHTSATVTRTLVDFFVEHKSQ---
Scomax_9       IQDR-NNFFLSLKKLEEAILWIPAFFLHTSATVTRTLVDFFVEHKGQ---
Corlav_9       IQDR-NNFFLNLKKLEGAILWIPAFFLHTSATVTRTLVDFFVEHKGQ---
Oncmyk_9       IQDR-NNFFLNLKKLEGAILWIPAFFLHTSATVTRTLVDFFVEHKGQ---
Salsal_9       IQDR-NNFFLNLKKLEGAILWIPAFFLHTSATVTRTLVDFFVEHKGQ---
Onctsh_9       IQDR-NNFFLNLKKLEGAILWIPAFFLHTSATVTRTLVDFFVEHKGQ---
Salalp_9       IQDR-NNFFLNLKKLEGAILWIPAFFLHTSATVTRTLVDFFVEHKGQ---
Ampoce_9       IQDR-NNFFLNLKKLEGAILWIPAFFLHTSATVTRTLVDFFVEHKGQ---
```

```
Ampper9         IQDR-NNFFLNLKKLEGAILWIPAFFLHTSATVTRTLVDFFVEHKGQ---
Labber_9        IQDR-NNFFLNLKKLEGAILWIPAFFLHTSATVTRTLVDFFVEHKGQ---
Umbpyg_3B       IQDR-NNFFLSLKKLDGAILWI-AFFFHTSATVTRTLVDFFVEHRGQ---
Esoluc_3b       IQDR-NNFFLSLKKLDGAILWIPAFFFHTSATVTRTLVDFFVEHRGQ---
Gadmor_9        IQDR-NNFFLNVKKLEGAILWIPAFFLHTSATVTRTLVDFFVEHKGQ---
Umbpyg_9        IQDR-NNFFLNLKKLEGAILWIPAFFLHTSATVTRTLVDFFVEHKGQ---
Esoluc_9        IQDR-NNFFLNLKKLEGAILWIPAFFLHTSATVTRTLVDFFVEHKGQ---
Permag_9        IQDR-NNFFLNLKKLEGAILWIPAFFLHTSATVTRTLVDFFVEHKGQ---
Bolpec_9        IQDR-NNFFLNLKKLEGAILWIPAFFLHTSATVTRTLVDFFVEHKGQ---
Poefor9         IQDR-NNFFLNLKKLEGAILWIPAFFLHTSATVTRTLVDFFVEHKGQ---
Masarm_9        IQDR-NNFFLSLKKLEGAILWIPAFFLHTSATVTRTLVDFFVEHKGQ---
Angjap_9        IQDR-NNFFLSLKKLDGVVLWIPAFFFHTSATVTRTLVDFFVEHRGQ---
Poelat_9        IQDR-NNFFLNLKKLEGAILWIPAFFLHTSATVTRTLVDFFVEHKGQ---
Poeret_9        IQDR-NNFFLNLKKLEGAILWIPAFFLHTSATVTRTLVDFFVEHKGQ---
Xipmac_9        IQDR-NNFFLNLKKLEGAILWIPAFFLHTSATVTRTLVDFFVEHKGQ---
Hapbur_9        IQDR-NNFFLNLKKLEGAILWIPAFFLHTSATVTRTLVDFFVEHKGQ---
Orylat_9        IQDR-NNFFLHLKKLEGAILWIPAFFLHTSATVTRTLVDFFVEHKGQ---
Gamaff_9        IQDR-NNFFLNLKKLEGAILWIPAFFLHTSATVTRTLVDFFVEHKGQ---
Neobri_9        IQDR-NNFFLNLKKLEGAILWIPAFFLHTSATVTRTLVDFFVEHKGQ---
Funhet_9        IQDR-NNFFLNLKKLEGAILWIPAFFLHTSATVTRTLVDFFVEHKGQ---
Punnye_9        IQDR-NNFFLNLKKLEGAILWIPAFFLHTSATVTRTLVDFFVEHKGQ---
Cypvar_9        IQDR-NNFFLNLKKLEGAILWIPAFFLHTSATVTRTLVDFFVEHKGQ---
Krymar_9        IQDR-NNFFLNLKKLEGAILWIPAFFLHTSATVTRTLVDFFVEHKGQ---
Notfur_9        IQDR-NNFFLNLKKLEGAILWIPAFFLHTSATVTRTLVDFFVEHKGQ---
Notpie_9        IQDR-NNFFLNLKKLEGAILWIPAFFLHTSATVTRTLVDFFVEHKGQ---
Orenil_9        IQDR-NNFFLNLKKLEGAILWIPAFFLHTSATVTRTLVDFFVEHKGQ---
Auslim_9        IQDR-NNFFLNLKKLEGAILWIPAFFLHTSATVTRTLVDFFVEHKGQ---
Anates_9        IQDR-NNFFLNLKKLEGAILWIPAFFLHTSATVTRTLVDFFVEHKGQ---
Astcal_9        IQDR-NNFFLNLKKLEGAILWIPAFFLHTSATVTRTLVDFFVEHKGQ---
Mayzeb_9        IQDR-NNFFLNLKKLEGAILWIPAFFLHTSATVTRTLVDFFVEHKGQ---
Orymel_9        IQDR-NNFFLHLKKLEGAILWIPAFFLHTSATVTRTLVDFFVEHKGQ---
Ampcit9         IQDR-NNFFLNLKKLEGAILWIPAFFLHTSATVTRTLVDFFVEHKGQ---
Macfas_2        ATWR-EKLLQRLHSLNGSILWIPAFMARGGKERVEWVNELILKHH-----
Macmul_2        ATWR-EKLLQRLHSLNGSILWIPAFMARGGKERVEWVNELILKHH-----
Macnem_2        ATWR-EKLLQRLHSLNGSILWIPAFMARGGKERVEWVNELILKHH-----
Musmus_2        ATWR-EKLLQRLHGLNGSILWIPAFMARGGKERVEWVNALILKHH-----
Ponabe_2        ATWR-EKLLQRLHSLNGSILWIPAFMARGGKERVEWVNELILKHH-----
Papanu_2        ATWR-EKLLQRLHSLNGSILWIPAFMARGGKERVEWVNELILKHH-----
Nomleu_2        ATWR-EKLLQRLHSLNGSILWIPAFMARGGKERVEWVNELILKHH-----
Pantro_2        ATWR-EKLLQRLHSLNGSILWIPAFMARGGKERVEWVNELILKHH-----
Latcha_2        ETWK-EKFLQRLQSLNGSIFWIPAFMAKGGEERVEWVNDLIIKSG-----
Gnapet2         QQWR-DHFLQRLRSIAGGVLWIPAFMAKGGEERVELAARLILRHG-----
Panbuc_2b       ESWQ-ERFLQRLDQLGSSVLWIPAFMAKGGEERVEWALDLIRQHA-----
Ostbic_2        KQWK-GRFLQRLQGLNGSVLWIPAFMAKGGEERVAWAVRLILQHA-----
Sinrhi2         EEWV-QRFVHRLQSLSGSVLWIPAFMAKGGEERVEWAIRLILLHT-----
Hetzeb_2        ETWK-EKLLHRLKMLDGGILWIPAFMAKGGEERVEWVNNLIIKHK-----
Hipcom_2        DEWR-RRFARRLASLSGSVLWIPAFMAKGGEERVEWALRLILRHA-----
Prigla_2        ETWK-EKLLHRLKMLDDGILWIPAFMAKGGEERVEWVNKLIIEHK-----
Scytor_2        DTWK-EKLLHRLKMLDGGILWIPAFMAKGGEERVEWVNRLIIEHK-----
Tetcal_2        ETWK-EKLLHRLTMLDGAILWIPAFMAKGGEERVEWVNNLIIEHK-----
Rhityp_2        ETWK-EKLLHRLKMLDGGILWIPAFMAKGGEERVEWVNNLIIKHK-----
Parkin_2b       ARWR-EHFLQRLRGLGDAVLWIPAFMAKGGERRVELALDVIRRQR-----
Angang_2        EAWR-ERFVRRLRALEGSVLWIPAFMAKGGEDRVEWATRLILRHA-----
Pygnat_2        EEWR-QRFVQRLQALSGSVLWIPAFMAKGGEERVEWAVRLILSHT-----
Parkin_2        QLWR-DHFLQRLRSIAGGVLWIPAFMAKGGEERVELAARLILRHS-----
Gorgor_2        ATWR-EKLLQRLHSLNGSILWIPAFMARGGKERVEWVNELILKHH-----
Thegel_2        ATWR-EKLLQRLHSLNGSILWIPAFMARGGKERVEWVNELILKHH-----
Tupchi_2        ATWR-EKLLQRLHSLNGSILWIPAFMARGGKERVEWVNELILKHR-----
Galgal_2        ETWR-EKLLQRLHSLNGSILWIPAFMAKGGKERVEWVNELILKHR-----
Eleele_2        EEWR-QQFVQRLRELGSSVLWIPAFMAKGGEERVERAIRLILSHT-----
Danrer_2        EEWV-QRFVQRLQSLSGSVLWIPAFMAKGGEERVEWAIRLILLHT-----
Canlupfam_2     ATWR-EKLLQRLRSLNGSILWIPAFMARGGKERVEWVNELILKHH-----
Aloalo_2        EEWR-QRFIRRLQDLSGSVLWIPAFMAKGGEERVEWAIRLILTHT-----
Astmex_2        KEWR-QRFVRRLQALSGSVLWIPAFMAKSGEDRVEWAVHLILSHT-----
Sinans_2        EEWV-QRFVHRLQSLSGSVLWIPAFMAKGGEERVEWAIRLILLHT-----
Sclfor_2        EQWK-GRFLRRLQGLNGSVLWIPAFMAKGGEERVAWAVRLILQHA-----
Ceraty_2        ATWR-EKLLQRLHSLNGSILWIPAFMARGGKERVEWVNELILKHH-----
Anocar_2        DTWR-EKLLQRLHSLNGSILWIPAFMAKGGKERVEWVNELILKNH-----
Anates_2        EEWR-ARFLRRLQSLSGSVLWIPAFMAKGGEERVEWALRLILLHT-----
Ampbic_2        EEWT-ERFLQRLRSLSGSVLWIPAFMAKGGEERVEWALRLILLHT-----
Sarpil_2        EEWR-QRFIRRLQDLSGSVLWIPAFMAKGGEERVEWAIRLILTHT-----
Xenlae_2        DTWK-DKFLQRLKSLNESILWIPAFMAKGGEERVEWVNDLIIKHH-----
Cluhar_2        EEWR-QRFVRRLQDLSGSVLWIPAFMAKGGEERVEWAIRLIIMHT-----
Cypcar_2        EEWV-QRFVHRLQSLSGSVLWIPAFMAKGGEERVEWAIRLILLHT-----
Denclu_2        EEWR-ERFVQRLRALSGSVLWIPAFMAKGGEERVEWAIRLILLHT-----
Notpie_2        EEWK-ERFLQRLRNLSGSVLWIPAFMAKRGEDRVEWVLRLILLHT-----
Erpcal2         DTWK-DRLLQRLQNLNGSILWIPAFMAKGGEQRVELVNSLILKHQ-----
```

```
Amical_2       ESWR-ERFLQRLQSLNGSVLWIPAFMAKGGEERVEWANDLILKHH-----
Lepocu_2       SHWR-ERFLERLSSLNGSVLWIPSFMAKGGEERVQWASELILRHG-----
Homsap_2       ATWR-EKLLQRLHSLNGSILWIPAFMARGGKERVEWVNELILKHH-----
Bostau_2       ATWR-EKLLQRLHSLNGSILWIPAFMARGGKQRVEWVNELILKHH-----
Caraur_2       EEWV-QRFVHRLQSLSGSVLWIPAFMAKGGEERVEWAIRLILLHT-----
Thythy_2B      EEWR-ERFLQRLRGLSGSVLWIPAFMAKGGEERVEWAIRLILLHT-----
Onctsh_2B      EEWR-ERFLQRLRSLSGSVLWIPAFMAKGGEERVEWAIRLILLHT-----
Salsal_2B      EEWR-ERFLQRLRGLSGSVLWIPAFMAKGGEERVEWAIRLILLHT-----
Oncmyk_2B      EEWR-ERFLQRLRSLSGSVLWIPAFMAKGGEERVEWAIRLILLHT-----
Onckis_2B      EEWR-ERFLQRLQSLSGSVLWIPAFMAKGGEERVEWAIRLILLHT-----
Salsal_2A      EQWR-ERFLQRLRGLSGSVLWIPAFMAKGGEERVEWAIRLILLHT-----
Thythy_2A      EQWR-ERFLQRLRGLSGSVLWIPAFMAKGGEERVEWAIRLILLHT-----
Salalp_2A      EQWR-ERFLQRLRGLSGSVLWIPAFMAKGGEERVEWAIRLILLHT-----
Corlav_2A      EQWR-ERFLQRLRGLSGSVLWIPAFMAKGGEERVEWAIRLILLHT-----
Cormar_2A      EQWR-ERFLQRLRGLSGSVLWIPAFMAKGGEERVEWAIRLILLHT-----
Onctsh_2A      EQWR-ERFLQRLRGLSGSVLWIPAFMAKGGEERVEWAIRLILLHT-----
Oncmyk_2A      EQWR-ERFLQRLRGLSGSVLWIPAFMAKGGEERVEWAIRLILLHT-----
Neobri_2       NEWK-ERFLRRLRSLSGSVLWIPAFMAKGGEERVEWALRLILLHT-----
Takrub_2       EEWR-ARFLRRLQSLSGSVLWIPAFMAKGGEERVEWTLRLILLHT-----
Tetnig_2       EEWR-DRFLRRLQSLSGSILWIPAFMAKGGEERVDWTLRLILLHT-----
Orenil_2       DEWK-ERFLRRLRSLSGSVLWIPAFMAKGGEERVEWALRLILLHT-----
Orylat_2       EEWK-QRFLQRLQSLGGSVLWIPAFMAKGGEERVEWALRLILRHT-----
Krymar_2       EEWR-ERFLQRLRDLRGGVLWVPAFMAKGGEERVEWALRLILLHA-----
Notfur_2       EEWK-ERFLQRLRNLSGSVLWIPAFMAKRGEDRVEWVLRLILLHT-----
Poelat_2       DEWK-DRFLQRLQNLSGSVLWIPAFMAKGGEERVEWALRLILVHT-----
Gadmor_2       PEWR-ARFVARLQSLRGSVLWIPAFMAKGGEERVEWAARLILLHT-----
Xipmac_2       DEWK-DRFLQRLQNLSGSVLWIPAFMAKGGEERVEWALRLILVHT-----
Poeret_2       DEWK-DRFLRRLQNLSGSVLWIPAFMAKGGEERVEWALRLILVHT-----
Astcal_2       NEWK-ERFLRRLRSLSGSVLWIPAFMAKGGEERVEWALRLILLHT-----
Hapbur_2       NEWK-ERFLRRLRSLSGSVLWIPAFMAKGGEERVEWALRLILLHT-----
Acapol_2B      EEWT-ERFLQRLRSLSGSVLWIPAFMAKGGEERVEWALRLILLHT-----
Ampoce_2A      EEWT-ERFLQRLRSLSGSVLWIPAFMAKGGEERVEWALRLILLHT-----
Acapol_2A      EEWT-ERFLQRLRSLSGSVLWIPAFMAKGGEERVEWALRLILLHT-----
Mayzeb_2       NEWK-ERFLRRLRSLSGSVLWIPAFMAKGGEERVEWALRLILLHT-----
Punnye_2       NEWK-ERFLRRLRSLSGSVLWIPAFMAKGGEERVEWALRLILLHT-----
Latcal_2       EEWR-DRFLQRLRSLSGSVLWIPAFMAKGGEERVEWALRLILLHT-----
Singra_2       EEWV-QRFVHRLQSLSGSVLWIPAFMAKGGEERVEWAIRLILLHT-----
Auslim_2       EEWK-ERFLRRLRDLGGSVLWIPAFMAKGGEERVEWALRLILLHA-----
Monalb2        DEWR-ERFLQRLQSLSGSVLWIPAFMAKGGEERVEWALRLILLHT-----
Ampoce_2B      EEWT-ERFLQRLRSLSGSVLWIPAFMAKGGEERVEWALRLILLHT-----
Chiham_2       EEWR-DRFLQRLQSLSGSVLWIPAFMAKGGEERVEWALRLILSHT-----
Gasacu_2       DEWR-DRFLQRLQSLSGSVLWIPAFMAKGGEERVEWALRLILSHT-----
Gymacu_2       EEWR-DRFLQRLQSLSGSVLWIPAFMAKGGEERVEWALRLILSHT-----
Labber_2       EEWR-DRFLQRLQSLSGSVLWIPAFMAKGGEERVEWALRLILLHT-----
Cypvar_2       EEWK-DRFLQRLRDLSGSVLWIPAFMAKGGEERVEWALRLILVHT-----
Funhet_2       DEWK-ERFLQRLRNLSGSVLWIPAFMAKGGEERVEWALRLIRVHT-----
Larcro_2       EEWR-DRFLKRLQSLSGSVLWIPAFMAKGGEERVEWALRLILLHT-----
Paroli_2       DEWR-DRFLQRLQSLSGSVLWIPAFMAKGGEERVEWALRLILLHT-----
Perflu_2       EEWR-DRFLQRLQSLSGSVLWIPAFMAKGGEERVEWALRLILLHT-----
Serdum_2       EEWR-DRFLQRLQSLSGSVLWIPAFMAKGGEERVEWALRLILLHT-----
Stepar_2       EEWK-ERFLQRLRSLSGSVLWIPAFMAKGGEERVEWALRLILLHT-----
Notcor_2       EEWR-DRFLQRLQSLSGSVLWIPAFMAKGGEERVEWALRLILSHT-----
Treber_2       EEWR-DRFLQRLQSLSGSVLWIPAFMAKGGEERVEWALRLILSHT-----
Serlaldor_2    EEWR-DRFLQRLQSLSGSVLWIPAFMAKGGEERVEWALRLILLHT-----
Plaste_2       DEWR-NRFLQRLQSLSGSVLWIPAFMAKGGEERVEWALRLILLHT-----
Clabat_4       ETTR-ERFVQRLRLLNDSVLWIPAFMVKGGEKHVEGVNELILKKK-----
Ictpun_4A      ETVR-ENFVQRLRLLNDSVLWIPAFMVKGGEKHVEVVNELILKRK-----
Panhyp_4       ETVR-ELFVQRLSLLNDSVLWIPAFMVKGGEKHVEGVNELILKRK-----
Umbpyg_4       ATDT-ERFVQRLRMLKDSVLWIPAFMVKGGEQHVERVNELIVKRK-----
Esoluc_4       ASDM-ERFVQRLRMLNDSVLWIPAFMVKGGERHVESVNELIVKRK-----
Plealt_4       VSDA-ERFVRRLRMLNDSVLWIPAFMVKGGEKHVECVNELIVKRK-----
Onckis_4B      ATDT-ERFVQRLRGLNDSVLWIPAFMVKGGERHVESVNELIVKRK-----
Onckis_4A      ATDT-ERFVQRLRGLNDSVLWIPAFMVKGGERHVESVNELIVKRK-----
Onctsh_4       ATDT-ERFVQRLRGLNDSVLWIPAFMVKGGERHVESVNELIVKRK-----
Oncmyk_4       ATDT-ERFVQRLRGLNDSVLWIPAFMVKGGERHVESVNELIVKRK-----
Corlav_4       ATDT-ERFVQRLRMLNDSVLWIPAFMVKGGERHVESVNELIVKRK-----
Astmex_4A      ETQR-ERFVERLSMLNDSVLWIPAFMVKGGEKHVEGVNELILKRK-----
Pygnat_4       ETVR-EHFVERLGMLNDSVLWIPAFMVKGGEKHVEGVNELILKRK-----
Aloalo_4       ATDR-ERFVERLRALNDSVLWIPAFMVKGGEKHVEGVNELILQRS-----
Cluhar_4       ATDR-ERFVERLQALNDSVLWIPAFMVKGGEKHVEGVNELILQRG-----
Denclu_4       ESDR-EKFVHRLIALNDSVLWIPAFMVKGGEKHVEVVNNFILKQG-----
Konpun4        STDR-ERFVERLRALNDSVLWIPAFMVKGGEKHVEGVNELILQRG-----
Sarpil_4       ATDR-ERFVERLRALNDSVLWIPAFMVKGGEKHVEGVNELILQRG-----
Caraur_4       ERQQ-ERFIRRLQQLNNSVLWIPAFMVKGGERHVEIVNQLILKHK-----
Cteide_4       ETEQ-ENFILRLQQLNNSVLWIPAFTVKGSERHVEIVNELILKHK-----
Petmar_4       KADH-DRFGRRLRALNSSILWIPAFMAKGGEQHVEIVNNLLLTLG-----
Cypcar_4       ERQR-ERFIQRLQQLNNSVLWIPAFMVKGGERHVEIVNELILKHK-----
```

```
Danrer_4        ETQQ-ENLIQRLRQLNDSVLWIPAFMVKGGMKHVDTVNELILKHK-----
Misang_4        KTAQ-EHFIQRLRQLNDSVLWIPAFMVKGSEKHVEGVNELILKNK-----
Angjap_4        DSDR-ERFVRRLGALNDSVLWIPAFMVKGGERHVECVNDLILRHR-----
Cypcar_4A       ERQR-ERFIQRLQQLNNSVLWIPAFMVKGGERHVEIVNELILKHK-----
Angang_4        DSDR-ERFVRRLGALNDSVLWIPAFMVKGGERHVECVNDLILRHR-----
Cypcar_4B       ERQR-ERFIRRLQQLNNSVLWIPAFMVKGGERHVEIVNQLILKHK-----
Sinans_4A       ERQR-ESFILRLQQLNNSVLWIPAFMVKGGERHVEIVNELILKHK-----
Sinans_4B       ERQR-VQFIRRLQQLNNSVLWIPAFMVKGGERHVEIVNQLILKHR-----
Singra_4A       ERQQ-ESFIRRLQQLNNSVFWIPAFMVKGGERHVETVNELILKHK-----
Aptalb_4        ETVR-EGFLRRLAALNDSVLWIPAFMVKGGEKHVEGVNELILRRK-----
Eigvir_4        ETAR-ESFLRRLAALNDSILWIPAFMVKGGEKHVEGVNELILRRK-----
Eleele_4        ETAR-DCFLRRLAVLNDSVLWIPAFMVKGGEKHVEGVNELILQRK-----
Parhas_4        VTVR-EGFLRRLAALNDSVLWIPAFMVKGGEKHVEGVNELILRRK-----
Sclfor_4        ESDR-VRFVQRLTMLNESVLWIPAFMVKGGEQHVERVNELILKNK-----
Gnapet4         ESDR-ERFVQRLIMLNDSILWIPAFMVKGGEKHVEGVNELILNRK-----
Ostbic_4        ESDR-EKFVQRLIMLNDSILWIPAFMVKGGEQHVEHVSELILKNR-----
Parkin_4        ESDR-ERFVQRLIMLNDSILWIPAFMVKGGEKHVEGVNELILNRK-----
Panbuc_4        ATDR-QRFVQRLVALNDSVLWIPAFMVKGGEKHVESVNELILKNG-----
Erpcal4         ETDR-EKFVQRLAMLNDSVLWIPAFMVKGGEKHVEWVNELILKNK-----
Salsal_4        ATDT-ERFVQRLRGLNDSVLWIPAFMVKGGERHVESVNELIVKRK-----
Saltru_4        ATDT-ERFVQRLRGLNDSVLWIPAFMVKGGERHVESVNELIVKRK-----
Salalp_4        ATDT-ERFVERLRGLNDSVLWIPAFMVKGGERHVESVNELIVKRK-----
Salfon_4        ATDT-ERFVERLRGLNDSVLWIPAFMVKGGERHVESVNELIVKRK-----
Thythy_4        ATDT-ERFVHRLQVLNDSVLWIPAFMVKGGERHVESVNELIVKRK-----
Calmil_4        ETDR-ENFVRRLAVLNDSVLWIPAFMVKGGERHVEWVNELILKNK-----
Hetzeb_4        ETDR-EKFVDRLAVLNDSVLWIPAFMVKGGEKHVEYVNELILKNR-----
Rhityp_4        ETDR-EKFVDRLTVLNDSVLWIPAFMVKGGEKYVEYVNELILKNR-----
Scytor_4        ETDR-EKFVDRLAVLNDSVLWIPAFMVKGGEKHVEYVNELILKNR-----
Squaca_4        ETDR-EKFVDRLAVLNDSVLWIPAFMVKGGEKHVEYVNELILKNR-----
Amical_4        ETDR-EKFVRRLTMLNDSVLWIPAFMVKGGEKHVECVNELILKNK-----
Lepocu_4        ETDR-ERFVQRLTLLNDSVLWIPAFMVKGGEKHVECVNELILKNK-----
Agema_4         ETVR-ERFVQRLSLLNDSVLWIPAFMVKGGEKHVEGVNELILKRK-----
Anocar_4        ESDR-EKFVHRLSMLNDSVLWIPAFMVKGGEKHVEWVNALILKNK-----
Canlupfam_4     ESDR-EKFVHRLSMLNDSVLWIPAFMVKGGEKHVEWVNALILKNK-----
Crigri_4        ESDR-AKFVHRLSMLNDSVLWIPAFMVKGGEKHVEWVNALILKNK-----
Galgal_4        ESDR-EKFGHRLSMLNDSVLWIPAFMVKGGEKHLEWVNALILKNK-----
Mesaur_4        ESDR-EKFVHRLSMLNDSVLWIPAFMVKGGEKHVEWVNALILKNK-----
Homsap_4        ESDR-EKFVHRLSMLNDSVLWIPAFMVKGGEKHVEWVNALILKNK-----
Notscuscu_4     ESDR-EKFVHRLSMLNDSVLWIPAFMVKGGEKHVQWVNALILKNK-----
Psetextex_4     ESDR-EKFVHRLSMLNDSVLWIPAFMVKGGEKHVQWVNALILKNK-----
Musmus_4        ESDR-EKFVHRLSMLNDSVLWIPAFMVKGGEKHVEWVNALILKNK-----
Pogvit_4        ESDR-EKFVHRLSMLNDSVLWIPAFMVKGGEKHVEWVNALILKNK-----
Promuc_4        ESDR-EKFVHRLSMLNDSVLWIPAFMVKGGEKHVEWVNALILKNK-----
Pantro_4        ESDR-EKFVHRLSMLNDSVLWIPAFMVKGGEKHVEWVNALILKNK-----
Pytbiv_4        ESDR-EKFVHRLSMLNDSVLWIPAFMVKGGEKHVEWVNALILKNK-----
Ratnor_2        ATWR-EKLLQRLHGLNGSILWIPAFMARGGKERVEWVNALILKHH-----
Ratnor_4        ESDR-EKFVHRLSMLNDSVLWIPAFMVKGGEKHVEWVNALILKNK-----
Siltro_2        DTWK-DKFLQRLKSLNESILWIPAFMAKGGEERVEWVNDLIIKHH-----
Siltro_4        ESDR-EKFVHRLSMLNDSVLWIPAFMVKGGEKHVEWVNALILKNQ-----
Susscr_4        ESDR-EKFVHRLSMLNDSVLWIPAFMVKGGEKHVEWVNALILKNK-----
Taegut_4        ESDR-EKFVHRLSMLNDSVLWIPAFMVKGGEKHVEWVNALILKNK-----
Termextri_4M    ESDR-EKFVHRLSMLNDSVLWIPAFMVKGGEKHVEWVNALILKNK-----
Thasirsir_4     ESDR-EKFVHRLSMLNDSVLWIPAFMVKGGEKHVEWVNALILKNK-----
Xenlae_4B       ESDR-EKFVHRLSMLNDSVLWIPAFMVKGGEKHVEWVNALILKNQ-----
Xenlae_4A       ESDR-EKFVHRLSMLNDSVLWIPAFMVKGGEKHVEWVNALILKNQ-----
                151
Latcal_3        LKVQLAWPGN-IMQYINNYWKTKQLSPKRLSTGILMYT----LASSMCDQ
Perflu_3        LKVQLAWPGN-IMQYVNSYWKTKQLSPKRLSTGILMYT----LASSMCDQ
Serdum_3        LKVQLAWPGN-IMQYINNYWKTKQLSPKRLSTGILMYT----LASSMCDQ
Stepar_3        LKVQLAWPGN-IMQYINNYWKTKQLSPKRLSTGILMYT----LASSMCDQ
Tetcal_3        LKVQLAWPGN-IMQHINKYWKTKQLSPKRLSTGILMYT----LASAICEE
Monalb_3        LKVQLAWPGN-IMQYINNYWKTKQLSPKRLSTGILMYT----LASSMCDQ
Molmol_3        LKVQLAWPGN-IMQYINNYWKTKQLSPKRLSTGILMYT----LASSMCDQ
Takrub_3        LKVRLAWPGN-IMQYINNYWKTKQLSPKRLSTGILMYT----LASSMCDQ
Canlupfam_3     LKVQLAWPGN-IMQHVNRYWKNKHLSPKRLSTGILMYT----LASAICEE
Bostau_3        LKLQLAWPGN-IMQHVNRYWKNKHLSPKRLSTGILMYT----LASAVCEE
Homsap_3        LKVQLAWPGN-IMQHVNRYWKNKHLSPKRLSTGILMYT----LASAICEE
Siltro_3        LKVQLDWPGN-IMQHVNRYWKNKHLSPKRLSTGILMYT----LASSVCEE
Anocar_3        LKVQLAWPGN-IMQHVNRYWKNKHLAPKRLSTGILMYT----LASAICDE
Musmus_3        LKVQLAWPGN-IMQHVNRYWKNKHLSPKRLSTGILMYT----LASAICEE
Pantro_3        LKVQLAWPGN-IMQHVNRYWKNKHLSPKRLSTGILMYT----LASAICEE
Ratnor_3        LKVQLAWPGN-IMQHVNRYWKNKHLSPKRLSTGFLMYT----LASAICEE
Galgal_3        LKVQLAWPGN-IMQHVNRYWKNKHLSPKRLSTGILMYT----LASAICEE
Lepocu_3        LKVQLAWPGN-IMQYVNRYWKTKHLAPKRLSTGILMYT----LASAMCEE
Tetnig_3        LKVQLAWPGN-IMQYINNYWKTKQLSPKRLSTGILMYT----LASSMCER
Squaca_3        LKIQLAWPGN-IMQHINKYWKTKQLSPKRLSTGILMYT----LASAICEE
Perfla_3        LKVQLAWPGN-IMQYVNSYWKTKQLSPKRLSTGILMYT----LASSMCDQ
```

```
Salsal_3        LKVQLAWPGN-IMQYVNRYWKTKQLSPKRLSTGILMYT----LASSMCEE
Serlaldor_3     LKVQLAWPGN-IMQYINNYWKTKQLSPKRLSTGILMYT----LASSMCDQ
Treber_3        LKVELAWPGN-IMHDVNKYWKTKNLSPKRLSTGILMYT----LASAMCDE
Cynsem_3        LKVQLAWPGN-IMQYIN-------------STGILMYT----LASSMCDQ
Paroli_3        LKVKLAWPGN-IMHDVNKYWKTKNLSPKRLSTGILMYT----LAYAMCDE
Paroli_3b       LKVQLAWPGN-IMQYINNYWKTKQLSPKRLSTGILMYT----LASSMCDQ
Plaste_3        LKVKLAWPGN-IMHDVNKYWKTKNLSPKRLSTGILMYT----LAYAMCDE
Plaste_3b       LKVQLAWPGN-IMQYINSYWKTKQLSPKRLSTGILMYT----LASSMCDQ
Erpcal3         LKVQLAWPGN-IMQHINRYWKTKHLAPKRLSTGILMYT----LASAMCEE
Calmil_3b       LKVELAWPGN-IMQYVNKYWKTKQLSPKRLSTGILMYT----LASSVCEE
Hetzeb_3        LKVQLAWPGN-IMQHINKYWKTKQLSPKRLSTGILMYT----LASAICEE
Latcha_3        LKLQLAWPGN-IMQHVNRYWKLKHLSPKRLSTGILMYT----LASAMCQE
Anates_3        LNVQLAWPGN-IMQYINNYWKTKQLSPKRLSTGILMYT----LASSMCDQ
Ampcit3         LKVQLAWPGN-IMKYVNNYWKTKQLSPKRLSTGILMYT----LASSMCDQ
Auslim_3        LKVQLAWPGN-IMQYINNYWKTKRLSPKRLSTGILMYT----LASSMCDQ
Cypvar_3        LKVQLAWPGN-IMQYINNYWKTKQLSPKRLSTGILMYT----LASSMCDQ
Krymar_3        LKVQLAWPGN-IMQYINNYWKTKQLSPKRLSTGILMYT----LASSMCDQ
Funhet_3        LKVQLAWPGN-IMQYINNYWKTKQLSPKRLSTGILMYT----LASSMCDQ
Gamaff3         LKVQLAWPGN-IMQHINNYWKTKQLSPKRLSTGILMYT----LASSMCDQ
Orylat_3        LKVQLAWPGN-IMQYINNYWKTKQLSPKRLSTGILMYT----LASSMCDQ
Orenil_3        LKVQLAWPGN-IMKYVNNYWKTKQLSPKRLSTGILMYT----LASSMCDQ
Mayzeb_3        LKVQLAWPGN-IMKYVNNYWKTKQLSPKRLSTGILMYT----LASSMCDQ
Punnye_3        LKVQLAWPGN-IMKYVNNYWKTKQLSPKRLSTGILMYT----LASSMCDQ
Punnye_3a       LKVQLAWPGN-IMKYVNNYWKTKQLSPKRLSTGILMYT----LASSMCDQ
Neobri_3        LKVQLAWPGN-IMKYVNNYWKTKQLSPKRLSTGILMYT----LASSMCDQ
Misang_3        LKVKLAWPGN-IMQYVNRYWKTKHLSPKRLSTGILMFT----LASSMCEQ
Sinrhi3         LKVQLAWPGN-IMQYVNRYWKTKQLSPKRLSTGILMFT----LASSLCEQ
Singra_3        LKVQLAWPGN-IMQYVNRYWKTKQLSPKRLSTGILMFT----LASSLCEQ
Hapbur_3        LKVQLAWPGN-IMKYVNNYWKTKQLSPKRLSTGILMYT----LASSMCDQ
Astcal_3        LKVQLAWPGN-IMKYVNNYWKTKQLSPKRLSTGILMYT----LASSMCDQ
Notfur_3        LKVQLAWPGN-IMQYINNYWKTKQLSPKRLSTGILMYT----LASSMCDQ
Cypcar_3        LKVRLAWPGN-IMQYVNRKWTTASF-------------------------
Poefor3         LKVQLAWPGN-IMQYINNYWKTKQLSPKRLSTGILMYT----LASSMCDQ
Poelat_3        LKVQLAWPGN-IMQYINNYWKTKQLSPKRLSTGILMYT----LASSMCDQ
Poeret_3        LRVQLAWPGN-IMQYINNYWKTKQLSPKRLSTGILMYT----LASSMCDQ
Angang_3        LKVQLAWPGN-IMQYVNRYWKTRQLSPKRLSTGILMYT----LASAMCEE
Xipmac_3        LKVQLAWPGN-IMQHINNYWKTKHLSPKRLSTGILMYT----LASSMCDQ
Masarm_3        LKVQLAWPGN-IMQYINNYWKTKQLSPKRLSTGILMYT----LASSMCDQ
Permag_3        LNVQLAWPGN-IMQYINNYWKTKQLSPKRLSTGILMYT----LASS----
Gnapet3         LKVQLAWPGN-IMQYVNRYWKTKQLSPKRLSTGILVYT----LAAAMCEE
Ostbic_3        LKVQLAWPGN-IMQHVNRFWKTKQLSPKRLSTGILMYT----LASGMCDQ
Gadmor_3        LKVQLAWPGN-IMQYVNSYWKTKQLSPKRLSTGILMYT----LASSMCDQ
Sclfor_3        LKVQLAWPGN-IMQHIKRFWKTKHLSPKRLSTGILMYT----LASAMCDQ
Acapol3         LKVQLAWPGN-IMQYINNYWKTKQLSPKRLSTGILMYT----LASSMCDQ
Ampoce_3        LKVQLAWPGN-IMQYINNYWKTKQLSPKRLSTGILMYT----LASSMCDQ
Sclfor_3b       LKVQLAWPGN-IMKFFNRYWKTKQLSPKRLSTGILMYT----LASVMCEE
Ostbic_3b       LKVQLAWPGN-IMKFFNRYWKTKQLSPKRLSTGILMYT----LASAMCEE
Parkin_3        LKVQLAWPGN-IMQYVNRYWKTKQLSPKRLSTGILVYT----LAAAMCEE
Ampper3         LKVQLAWPGN-IMQYINNYWKTKQLSPKRLSTGILMYT----LASSMCDQ
Oncmyk_3        LKVQLAWPGN-IMQYINRYWKTKQLSPKRLSTGILMYT----LASSMCEE
Gasacu_3        LKVRLAWPGN-IMQYVNSYWKTKQLSPKRLSTGILMYT----LASTMCDQ
Labber_3        LKVQLAWPGN-IMQYINSYWKTKQLSPKRLSTGILMYT----LASSMCNQ
Panbuc_3        LKMRLAWPGN-IMQYFNSYWRTKQLSPKRLSTGILMYT----LASAMCEE
Parhas3         LRVQLAWPGN-IMKYINRYWKTRQLSPKRLSTGILMFT----LASSVCEQ
Cluhar_3        LKVQLAWPGN-IMQYVNRYWKNKQLSPKRLSTGILMYT----LASSLCDQ
Aloalo_3        LKVQLAWPGN-IMQYVNRYWKNKQLSPKRLSTGILMYT----LASSLCDQ
Panhyp_3        LRVQLAWPGN-IMQYVNRYWKTKQLSPKRLSTGILMFT----LASSLCEQ
Eleele_3        LRVQLAWPGN-IMTYINRYWKTRQLSPKRLSTGILMFT----LASSMCKQ
Denclu_3        LKVQLAWPGN-IMQYVNRYWKTKQLSPKRLSTGILMYT----LASSLCEQ
Sarpil_3        LKVQLAWPGN-IMQYVNRYWKNKQLSPKRLSTGILMYT----LASSLCDQ
Eigvir_3        LRVQLAWPGN-IMKYINRYWKTRQLSPKRLSTGILMFT----LASSVCEQ
Aptalb_3        LRVQLAWPGN-IMKYINRYWKTRQLSPKRLSTGILMFT----LASSVCEQ
Sinans_3        LKVQLAWPGN-IMQYVNRYWKTKQLSPKRLSTGILMFT----LASSLCEQ
Pygnat_3        LRVQLAWPGN-IMQYVNRYWKTKQLSPKRLSTGILMFT----LASSLCEQ
Astmex_3        LRVQLAWPGN-IMQYVNRYWKTKQLSPKRLSTGILMFT----LASSLCEQ
Caraur_3        LKVQLAWPGN-IMQYVNRYWKTKQLSPKRLSTGILMFT----LASSLCEQ
Danrer_3        LKVQLAWPGN-IMQYVNKYWKTKQLSPKRLSTGILMFT----LASSLCEQ
Ictpun_3        LRVQLAWPGN-IMQYVNRYWKTKQLAPKRLSTGILMFT----LASSLCDQ
Plealt_3        LKVQLAWPGN-IMQYVNKYWKTKQLSPKRLSTGILMYT----LASSMCDE
Plealt_9        LKIELAWPGN-IMQDVNKYWKTKNLSPKRLSTGILMYT----LASAMCEE
Gasacu_9        LKVELAWPGN-IMHDVNKYWKTKNLSPKRLSTGILMYT----LASAMCDE
Parkin_9        LNVQLAFPGN-IMHYINSYWKTKQLSPKRLSTGILMYT----LASAMCEE
Acapol_9        LKVELAWPGN-IMHDVNKYWKTKNLSPKRLSTGILMYT----LASAMCDE
Larcro_9        LKVELAWPGN-IMHDVNKYWKTKNLSPKRLSTGILMYT----LASAMCDE
Notcor_9        LKVELAWPGN-IMHDVNKYWKTKNLSPKRLSTGILMYT----LASAMCDE
Latcal_9        LKVELAWPGN-IMHDVNKYWKTKNLSPKRLSTGILMYT----LAYAMCDE
Serlaldor_9     LKVELAWPGN-IMHDVNKYWKTKNLSPKRLSTGILMYT----LAYAMCDE
```

```
Monalb_9       LKVDLAWPGN-IMHNINKYWKTKSLSPKRLSTGILMYT----LATAMCDE
Serdum_9       LKVELAWPGN-IMHDVNKYWKTKNLSPKRLSTGILMYT----LAYAMCDE
Perflu_9       LKIELAWPGN-IMHDVNKYWKTKNLSPKRLSTGILMYT----LASAMCDE
Perfla_9       LKIELAWPGN-IMHDVNKYWKTKNLSPKRLSTGILMYT----LASAMCDE
Stepar_9       LKVELAWPGN-IMHDVNKYWKTKNLSPKRLSTGILMYT----LASAMCDE
Molmol_9       LKVELAWPGN-IMHDVNKYWKTKNLSPKRLSTGILMYT----LASAMCDE
Hipcom_9       LKVELAWPGN-IMHDVNKYWKTKNLSPKRLSTGILMYT----LASAMCDE
Takrub_9 B     LKVELAWPGN-IMHDVNKYWKTKNLSPKRLSTGILMYT----LASAMCDE
Takrub_9       LKVELAWPGN-IMHDVNKYWKNKNLSPKRVSTGIFMYT----LASAMCDE
Cynsem_9       LKVKLAWPGN-IMHDVNKYWKTKNLSPKRLSTGILMYT----LAYAMCDE
Scomax_9       LKVKLAWPGN-IMHDVNKYWKTKNLSPKRLSTGILMYT----LAYAMCDE
Corlav_9       LKIELAWPGN-IMQDVNKYWKTKNLSPKRLSTGILMYT----LASAMCEE
Oncmyk_9       LKIELAWPGN-IMQDVNKYWKTKNLSPKRLSTGILMYT----LASAMCEE
Salsal_9       LKIELAWPGN-IMQDVNKYWKTKNLSPKRLSTGILMYT----LASAMCEE
Onctsh_9       LKIELAWPGN-IMQDVNKYWKTKNLSPKRLSTGILMYT----LASAMCEE
Salalp_9       LKIELAWPGN-IMQDVNKYWKTKNLSPKRLSTGILMYT----LASAMCEE
Ampoce_9       LKVELAWPGN-IMHDVNKYWKTKNLSPKRLSTGILMYT----LASAMCDE
Ampper9        LKVELAWPGN-IMHDVNKYWKTKNLSPKRLSTGILMYT----LASAMCDE
Labber_9       LKVELAWPGN-IMHDVNKYWKTKNLSPKRLSTGILMYT----LASAMCDE
Umbpyg_3B      LKVKLAWPGN-IMHYVNRYWKTKHLSPKRLSTGILMYT----LASAMCEE
Esoluc_3b      LKVQLAWPGN-IMHYINRYWKTKQLSPKRLSTGILMYT----LASAMCEE
Gadmor_9       LKIELAWPGN-IMQDVNKYWKTKNLSPKRLSTGILMYT----LASAMCEE
Umbpyg_9       LKVELAWPGN-IMQDVNKYWKTKNLSPKRLSTGILMYT----LASAMCEE
Esoluc_9       LKVELAWPGN-IMQDVNKYWKTKNLSPKRLSTGILMYT----LASAMCEE
Permag_9       LKVELAWPGN-IMHDVNKYWRTKNLSPKRLSTGILMYT----LASAMCDE
Bolpec_9       LKVELAWPGN-IMHDVNKYWRTKNLSPKRLSTGILMYT----LASAMCDE
Poefor9        LKVELAWPGN-IMHDVNKYWKTKNLSPKRLSTGILMYT----LASAMCDE
Masarm_9       LKVELAWPGN-IMHDVNKYWKTKNLSPKRLSTGILMYT----LASAMCDE
Angjap_9       LKVQLAWPGN-IMQYVNRYWKTRQLSPKRLSTGILMYT----LASAMCEE
Poelat_9       LKVELAWPGN-IMHDVNKYWKTKNLSPKRLSTGILMYT----LASAMCDE
Poeret_9       LKVELAWPGN-IMHDVNKYWKTKNLSPKRLSTGILMYT----LASAMCDE
Xipmac_9       LKIELAWPGN-IMHDVNKYWKTKNLSPKRLSTGILMYT----LASAMCDE
Hapbur_9       LKIELAWPGN-IMHDVNKYWKTKNLSPKRLSTGILMYT----LASAICDE
Orylat_9       LKVELAWPGN-IMHNVNKYWKTKNLSPKRLSTGILMYT----LAFSMCDE
Gamaff_9       LKVELAWPGN-IMHDVNKYWKTKNLSPKRLSTGILMYT----LASAMCDE
Neobri_9       LKIELAWPGN-IMHDVNKYWKTKNLSPKRLSTGILMYT----LASAICDE
Funhet_9       LKVELAWPGN-IMHDVNKYWKTKNLSPKRLSTGILMYT----LAYAMCDE
Punnye_9       LKIELAWPGN-IMHDVNKYWKTKNLSPKRLSTGILMYT----LASAICDE
Cypvar_9       LKVELAWPGN-IMHDVNKYWKTKNLSPKRLSTGILMYT----LAYAMCDE
Krymar_9       LKVELAWPGN-IMHDVNKYWKTKNLSPKRLSTGILMYT----LASAMCDE
Notfur_9       LKVELAWPGN-IMHDVNKYWKTKNLSPKRLSTGILMYT----LASAMCDE
Notpie_9       LKVELAWPGN-IMHDVNKYWKTKNLSPKRLSTGILMYT----LASAMCDE
Orenil_9       LKIELAWPGN-IMHDVNKYWKTKNLSPKRLSTGILMYT----LASAICDE
Auslim_9       LKVELAWPGN-IMQDVNKYWKTKNLSPKRLSTGILMYT----LASAMCDE
Anates_9       LKVELAWPGN-IMHDVNKYWKTKNLSPKRLSTGILMYT----LAYAMCDE
Astcal_9       LKIELAWPGN-IMHDVNKYWKTKNLSPKRLSTGILMYT----LASAICDE
Mayzeb_9       LKIELAWPGN-IMHDVNKYWKTKNLSPKRLSTGILMYT----LASAICDE
Orymel_9       LKVELAWPGN-IMHNVNKYWKTKNLSPKRLSTGILMYT----LAFAMCDE
Ampcit9        LKIELAWPGN-IMHDVNKYWKTKNLSPKRLSTGILMYT----LASAICDE
Macfas_2       VNVRTAYPSLRLLHAVRGYWLTNKVHIKRPTTGLLMYT----LATRFCNQ
Macmul_2       VNVRTAYPSLRLLHAVRGYWLTNKVHIKRPTTGLLMYT----LATRFCNQ
Macnem_2       VNVRTAYPSLRLLHAVRGYWLTNKVHIKRPTTGLLMYT----LATRFCNQ
Musmus_2       VNVRTAYPSLRLLHAVRGYWLTNKVHIKRPTTGLLMYT----LATRFCNQ
Ponabe_2       VNVRTAYPSLRLLHAVRGYWLTNKVHIKRPTTGLLMYT----LATRFCNE
Papanu_2       VNVRTAYPSLRLLHAVRGYWLTNKVHIKRPTTGLLMYT----LATRFCNQ
Nomleu_2       VNVRTAYPSLRLLHAVRGYWLTNKVHIKRPTTGLLMYT----LATRFCNQ
Pantro_2       VNVRTAYPSLPLLHAVRGYWLTNKVHIKRPTTGLLMYT----LATRFCNQ
Latcha_2       INVQTAYPSLRLLHAVRGYWLTNKVLIKRPSTGLLMYT----LATRFCSE
Gnapet2        LNVRPAFPSLRLLDAVRGYWLTNKVQIKRPTTGLLMYT----MATRFCDE
Panbuc_2b      VHVRPAFPSLRLLHAVRGYWLTNNVHIKRPTTGLLMYT----LATRFCDQ
Ostbic_2       INVHTAFPSLRLLHAVRGYWLTNKVQIKRPTTGLLMYT----MATRFCEQ
Sinrhi2        VNVRTAFPSLRLLHAVRGYWLTNHVQIKRPTTGLLMYT----MATRFCDE
Hetzeb_2       IRVQTAYPSLRLLHAVRGYWLTNRILIKRPTTGLLMYT----LATRFCDE
Hipcom_2       ADVRTAFPSLRLLHAVRGYWLTNKVHIKRPTTGLLMYT----MATRFCEE
Prigla_2       IHVQTAYPSLRFLHAVRGYWLTNRVLIKRPTTGLLMYT----LATRFCDE
Scytor_2       IHVQTAYPSLRFLHAVRGYWLTNRVLIKRPTTGLLMYT----LATRFCDE
Tetcal_2       IHVQTAYPSLRLLHAVRGYWLTNRVFIKRPTTGLLMYT----LATRFCDE
Rhityp_2       LHVQTAYPSLRLLHAVRGYWLTNKVLIKRPTTGLLMYT----LATRFCDE
Parkin_2b      LAVRPAFPSLRLLDAVRGYWLTNKVHIKRPTTGLLMYT----MATRFCEE
Angang_2       LNVRTAFPSLRLLHAVRGYWLTNKVLIKRPTTGLLMYT----MATRFCDE
Pygnat_2       VNVHTAFPSLRLLHAVRGYWLTNNVQIKRPTTGLLMYT----MATRFCDE
Parkin_2       LDVRPAFPSLRLLDAVRGYWLTNKVQIKRPTTGLLMYT----MATRFCDE
Gorgor_2       VNVRTAYPSLRLLHAVRGYWLTNKVHIKRPTTGLLMYT----LATRFCNQ
Thegel_2       VNVRTAYPSLRLLHAVRGYWLTNKVHIKRPTTGLLMYT----LATRFCNQ
Tupchi_2       VNVRTAYPSLRLLHAVRGYWLTNKVHIKRPTTGLLMYT----LATRFCNQ
Galgal_2       INVRTAYPSLRLLHAVRGYWLTNKVHIKRPTTGLLMYT----LATRFCNR
Eleele_2       GNVHAAFPSLRLLHAVRGYWLTNNIQIKRPTTGLLMYT----MATRFCDE
```

```
Danrer_2        VNVRTAFPSLRLLHAVRGYWLTNHVQIKRPTTGLLMYT----MATRFCDE
Canlupfam_2     VNVRTAYPSLRLLHAVRGYWLTNKVHIKRPTTGLLMYT----LATRFCNQ
Aloalo_2        VNVRTAFPSLRLLHAVRGYWLTNQVQIKRPTTGLLMYT----MATRFCDE
Astmex_2        VSVHAAFPSLRLLHAVRGYWLTNNIQIKRPTTGLLMYT----MATRFCEE
Sinans_2        VNVRTAFPSLRLLHAVRGYWLTNHVQIKRPTTGLLMYT----MATRFCDE
Sclfor_2        INVHTAFPSLRLLHAVRGYWLTNNVQIKRPTTGLLMYT----MATRFCEQ
Ceraty_2        VNVRTAYPSLRLLHAVRGYWLTNKVHIKRPTTGLLMYT----LATRFCNQ
Anocar_2        INVRTAYPSLRLLHAVRGYWLTNKVYIKRPTTGLLMYT----LATRFCNR
Anates_2        VDVRTAFPSLRLLHAVRGYWLTNNVHIKRPTTGLLMYT----MATRFCEE
Ampbic_2        VDVRTAFPSLRLLHAVRGYWLTNNVHIKRPTTGLLMYT----MATRFCEE
Sarpil_2        VNVRTAFPSLRLLHAVRGYWLTNQVQIKRPTTGLLMYT----MATRFCDE
Xenlae_2        INVHTAYPSLRLLHAVRGYWLTNKVHIKRPTTGILMYT----LATRFCNR
Cluhar_2        VNVRTAFPSLRLLHAVRGYWLTNQVQIKRPTTGLLMYT----MATRFCDE
Cypcar_2        VNVRTAFPSLRLLHAVRGYWLTNHVQIKRPTTGLLMYT----MATRFCDE
Denclu_2        VNVRTAFPSLRLLHAVRGYWLTNQVLIKRPTTGLLMYT----MATRFCEE
Notpie_2        VDVRMAFPSLRLLHAVRGYWLTNNVHIKRPTTGLLMYT----MATRFCEE
Erpcal2         INVQTAYPSLRLLYAVRGYWLTNKVPIKRPSTGLLMYT----LATRFCDE
Amical_2        INVRTAFPSLRLLHAVRGYWLTNKVPIKRPTTGMLMYT----LATRFCEE
Lepocu_2        INVRTAFPSLRLLHAVRGYWLTNKVPIKRPTTGLLMYT----LATRFCEE
Homsap_2        VNVRTAYPSLRLLHAVRGYWLTNKVHIKRPTTGLLMYT----LATRFCKQ
Bostau_2        VNVRTAYPSLRLLHAVRGYWLTNKVHIKRPTTGLLMYT----LATRFCNQ
Caraur_2        VNVRTAFPSLRLLHAVRGYWLTNQVQIKRPTTGLLMYT----MATRFCDE
Thythy_2B       VDVHTAFPSLRLLHAVRGYWLTNNVQIKRPTTGLLMYT----MATRFCEE
Onctsh_2B       VDVHTAFPSLRLLHAVRGYWLTNNVQIKRPTTGLLMYT----MATRFCEE
Salsal_2B       VDVHTAFPSLRLLHAVRGYWLTNNVQIKRPTTGLLMYT----MATRFCEE
Oncmyk_2B       VDVHTAFPSLRLLHAVRGYWLTNNVQIKRPTTGLLMYT----MATRFCEE
Onckis_2B       VDVHTAFPSLRLLHAVRGYWLTNNVQIKRPTTGLLMYT----MATRFCEE
Salsal_2A       VDVHTAFPSLRLLHAVRGYWLTNNVQIKRPTTGLLMYT----MATRFCEE
Thythy_2A       VDVHTAFPSLRLLHAVRGYWLTNNVQIKRPTTGLLMYT----MATRFCEE
Salalp_2A       VDVHTAFPSLRLLHAVRGYWLTNNVQIKRPTTGLLMYT----MATRFCEE
Corlav_2A       VDVHTAFPSLRLLHAVRGYWLTNNVQIKRPTTGLLMYT----MATRFCEE
Cormar_2A       VDVHTAFPSLRLLHAVRGYWLTNNVQIKRPTTGLLMYT----MATRFCEE
Onctsh_2A       VDVHTAFPSLRLLHAVRGYWLTNNVQIKRPTTGLLMYT----MATRFCEE
Oncmyk_2A       VDVHTAFPSLRLLHAVRGYWLTNNVQIKRPTTGLLMYT----MATRFCEE
Neobri_2        VDIRTAFPSLRLLHAVRGYWLTNNVHIKRPTTGLLMYT----MATRFCEE
Takrub_2        VDVRTAFPSLRLLHAVRGYWLTNNVHIKRPTTGLLMYT----MATRFCEE
Tetnig_2        VDVRTAFPSLRLLHAVRGYWLTNNVHIKRPTTGLLMYT----MATRFCEE
Orenil_2        VDIRTAFPSLRLLHAVRGYWLTNNVHIKRPTTGLLMYT----MATRFCEE
Orylat_2        VDVRTAFPSLRLLHAVRGYWLTNNVHIKRPTTGLLMYT----MATRFCEE
Krymar_2        VDVRMAFPSLRLLHAVRGYWLTNNVHIKRPTTGLLMYT----MATRFCEE
Notfur_2        VDVRMAFPSLRLLHAVRGYWLTNNVHIKRPTTGLLMYT----MATRFCEE
Poelat_2        VDVRTAFPSLRLLHAVRGYWLTNNVHIKRPTTGLLMYT----MATRFCEE
Gadmor_2        VGIRTAFPSLRLLHAVRGYWLTNNVQIKRPTTGLLMYT----MATRFCEE
Xipmac_2        VDVRTAFPSLRLLHAVRGYWLTNNVHIKRPTTGLLMYT----MATRFCEE
Poeret_2        VDVRTAFPSLRLLHAVRGYWLTNNVHIKRPTTGLLMYT----MATRFCEE
Astcal_2        VDIRTAFPSLRLLHAVRGYWLTNNVHIKRPTTGLLMYT----MATRFCEE
Hapbur_2        VDIRTAFPSLRLLHAVRGYWLTNNVHIKRPTTGLLMYT----MATRFCEE
Acapol_2B       VDVRTAFPSLRLLHAVRGYWLTNNVHIKRPTTGLLMYT----MATRFCEE
Ampoce_2A       VDVRTAFPSLRLLHAVRGYWLTNNVHIKRPTTGLLMYT----MATRFCEE
Acapol_2A       VDVRTAFPSLRLLHAVRGYWLTNNVHIKRPTTGLLMYT----MATRFCEE
Mayzeb_2        VDIRTAFPSLRLLHAVRGYWLTNNVHIKRPTTGLLMYT----MATRFCEE
Punnye_2        VDIRTAFPSLRLLHAVRGYWLTNNVHIKRPTTGLLMYT----MATRFCEE
Latcal_2        VDVRTAFPSLRLLHAVRGYWLTNNVHIKRPTTGLLMYT----MATRFCEE
Singra_2        VNVRTAFPSLRLLHAVRGYWLTNHVQIKRPTTGLLMYT----MATRFCDE
Auslim_2        VDVRMAFPSLRLLHAVRGYWLTNNVHIKRPTTGLLMYT----MATRFCEE
Monalb2         VDVRTAFPSLRLLHAVRGYWLTNNVHIKRPTTGLLMYT----LATRFCEE
Ampoce_2B       VDVRTAFPSLRLLHAVRGYWLTNNVHIKRPTTGLLMYT----MATRFCEE
Chiham_2        VDVRTAFPSLRLLHAVRGYWLTNHVHIKRPTTGLLMYT----MATRFCDE
Gasacu_2        VDVRTAVPSLRLLHAVRGYWLTNNVHIKRPTTGLLMYT----MATRFCEE
Gymacu_2        VDVRTAFPSLRLLHAVRGYWLTNHVHIKRPTTGLLMYT----MATRFCDE
Labber_2        VDVRTAFPSLRLLHAVRGYWLTNNVHIKRPTTGLLMYT----MATRFCEE
Cypvar_2        VDVRTAFPSLRLLHAVRGYWLTNNVHIKRPTTGLLMYT----MATRFCEE
Funhet_2        VDVRTAFPSLRLLHAVRGYWLTNNVHIKRPTTGLLMYT----MATRFCEE
Larcro_2        VDVRTAFPSLRLLHAVRGYWLTNNVHIKRPTTGLLMYT----MATRFCEE
Paroli_2        VDVRTAFPSLRLLHAVRGYWLTNNVHIKRPTTGLLMYT----MATRFCEE
Perflu_2        VDVRTAFPSLRLLHAVRGYWLTNNVHIKRPTTGLLMYT----MATRFCEE
Serdum_2        VDVRTAFPSLRLLHAVRGYWLTNNVHIKRPTTGLLMYT----MATRFCEE
Stepar_2        VDVRTAFPSLRLLHAVRGYWLTNNVHIKRPTTGLLMYT----MATRFCEE
Notcor_2        VDVRTAFPSLRLLHAVRGYWLTNHVHIKRPTTGLLMYT----MATRFCDE
Treber_2        VDVRTAFPSLRLLHAVRGYWLTNHVHIKRPTTGLLMYT----MATRFCDE
Serlaldor_2     VDVRTAFPSLRLLHAVRGYWLTNNVHIKRPTTGLLMYT----MATRFCEE
Plaste_2        VDVRTAFPSLRLLHAVRGYWLTNNVHIK-PTTGLLMYT----MATRFCDE
Clabat_4        LPVRMAYPSLRLVHAVRGYWLTNKVNIKRPSTGLLMYT----MATRFCDE
Ictpun_4A       LPVHMAYPSLRLVHAVRGYWLTNKVNIKRPSTGLLMYT----MATRFCDE
Panhyp_4        LPVRMAYPSLRLVHAVRGYWLTNKVNIKRPSTGLLMYT----MATRFCDE
Umbpyg_4        LSVRTAYPSLRLIHAVRGYWLTNKINIKRPSTGLLMYT----LATRFCDE
Esoluc_4        LRVRTAYPSLRLIHAVRGYWLTNKINIKRPSTGLLMYT----LATRFCDE
```

```
Plealt_4        LRVKTAYPSLRLVHAVRGYWLTNKINIKRPSTGLLMYT----LATRFCNE
Onckis_4B       LRVRTAYPSLRLIHVVRGYWLTNKINIKRPSTGLLMYT----LATRFCDE
Onckis_4A       LRVRTAYPSLRLIHVVRGYWLTNKINIKRPSTGLLMYT----LATRFCDE
Onctsh_4        LRVRTAYPSLRLIHVVRGYWLTNKINIKRPSTGLLMYT----LATRFCDE
Oncmyk_4        LRVRTAYPSLRLIHVVRGYWLTNKINIKRPSTGLLMYT----LATRFCDE
Corlav_4        LRVRTAYPSLRLIHAVRGYWLTNKINIKRPSTGLLMYT----LATRFCDE
Astmex_4A       LPLRTAYPSLRLIHAVRGYWLTNKVNIKRPSTGLLMYT----MATRFCDE
Pygnat_4        LPVHMAYPSLRLIHAVRGYWLTNKVNIKRPSTGLLMYT----MATRFCDE
Aloalo_4        LRVRTAYPSLRLIHAVRGYWLTNRIHIKRPSTGLLMYT----LATRFCDE
Cluhar_4        LSVKPAYPSLRLIHAVRGYWLTNKIHIKRPSTGLLMYT----LATRFCDE
Denclu_4        LHLRMAYPSLRLFHAVRGYWLTNKVNIKRPSTGLLMYT----LATRFCDE
Konpun4         LRVRTAYPSLRLIHAVRGYWLTNKIHIKRPSTGLLMYT----LATRFCDE
Sarpil_4        LRVRTAYPSLRLIHAVRGYWLTNRIHIKRPSTGLLMYT----LATRFCDE
Caraur_4        LKVRTAYPSLRLIHAVRGYWLTNKINIKRPSTGLLMYT----MATRFCDE
Cteide_4        LKVRMAYPSLRLIHAVRRYWLTNKINIKRPTTGLLMYT----MATRFCDE
Petmar_4        LPLRAAFPSLRLMHAVRGYWLTNKVYIKRPTTGLLMYT----LATRFCRE
Cypcar_4        LRLRTAYPSLRLIHAVRGFWLTNKINIKRPSTGLLMYT----LATRFCDE
Danrer_4        LKVRTAYPSLRLIHAVRGFWLTNKINIKRPTTGLLMYT----MATRFCDE
Misang_4        LKVRTAYPSLRLIHAVRGYWLTNKIKIKRPSTGLLMYT----MFTRFCDE
Angjap_4        LRVRTAYPSLRLIHAVRGYWLTNKVNIKRPSTGLLMYT----LATRFCDE
Cypcar_4A       LRLRTAYPSLRLIHAVRGFWLTNKINIKRPSTGLLMYT----LATRFCDE
Angang_4        LRVRPPTPRSGLIHAVRGYWLTNKVNIKRPSTGLLMYT----LATRFCDE
Cypcar_4B       LKVRTAYPSLRLIHAVRGYWLTNKINIKRPSTGLLMYT----LATRFCDE
Sinans_4A       LKVRTAYPSLRLIHAVRGYWLTNKINIKRPSTGLLMYT----MATRFCDE
Sinans_4B       LKVRTAYPSLRLIHAVRGYWLTNKINIKRPSTGLLMYT----MATRFCDE
Singra_4A       LKVRTAYPSLRLIHAVRGYWLTNKINIKRPSTGLLMYT----MATRFCDE
Aptalb_4        LPVRMAYPSLRLVHAVRGYWLTNKVNIKRPSTGLLMYT----MATRFCDE
Eigvir_4        LPVHMAYPSLRLIHAVRGYWLTNKVNIKRPSTGLLMYT----IATRFCDE
Eleele_4        LPVRMAYPSLRLIHAVRGYWLTNKVNIKRPSTGLLMYT----MATRFCDE
Parhas_4        LPVRMAYPSLRLVHAVRGYWLTNKVNIKRPSTGLLMYT----MATRFCDE
Sclfor_4        LKLRTAYPSLRLIHAVRGYWLTNKINIKRPSTGLLMYT----LATRFCDE
Gnapet4         LLLRTAYPSLRLIHAVRGYWLTNKVNIKRPSTGLLMYT----LATRFCNE
Ostbic_4        LRVRTAYPSLRLNHAVRGYWLTNKIKIKRPSTGLLMYT----LATRFCDE
Parkin_4        LLLRTAYPSLRLIHAVRGYWLTNKVNIKRPSTGLLMYT----LATRFCNE
Panbuc_4        LKVRTAYPSLRLIHAVRGYWLTNKVNIKRPSTGLLMYT----LATRFCDE
Erpcal4         LKVRTAYPSLRLIHAVRGYWLTNKINIKRPSTGLLMYT----LATRFCDE
Salsal_4        LRVRTAYPSLRLIHVVRGYWLTNKINIKRPSTGLLMYT----LATRFCDE
Saltru_4        LRVRTAYPSLRLIHVVRGYWLTNKINIKRPSTGLLMYT----LATRFCDE
Salalp_4        LRVRTAYPSLRLIHVVRGYWLTNKINIKRPSTGLLMYT----LATRFCDE
Salfon_4        LRVRTAYPSLRLIHVVRGYWLTNKINIKRPSTGLLMYT----LATRFCDE
Thythy_4        LSVRTAYPSLRLIHAVRGYWLTNKINIKRPSTGLLMYT----LATRFCDE
Calmil_4        LSIQTAYPSLRLIHAVRGYWLTNKVYIKRPSTGLLMYT----LATRFCDE
Hetzeb_4        LSIQTAYPSLRLIHAVRGYWLTNKIYIKRPSTGLLMYT----LATRFCDE
Rhityp_4        LNIQTAYPSLRLIHAVRGYWLTNKIYIKRPSTGLLMYT----LATRFCDE
Scytor_4        LNIQTAYPSLRLIHAVRGYWLTNKIYIKRPSTGLLMYT----LSTRFCDE
Squaca_4        LSIQTAYPSLRLIHAVRGYWLTNKVYIKRPSTGLLMYT----LATRFCDE
Amical_4        LKVRTAYPSLRLIHAVRGYWLTNKINIKRPSTGLLMYT----LATRFCDE
Lepocu_4        LKVRTAYPSLRLIHAVRGYWLTNKINIKRPSTGLLMYT----LATRFCDE
Agema_4         LPVRMAYPSLRLVHAVRGYWLTNKVNIKRPSTGLLMYT----MATRFCDE
Anocar_4        LKVRTAYPSLRLIHAVRGYWLTNKVFIKRPSTGLLMYT----LATRFCDE
Canlupfam_4     LKVRTAYPSLRLIHAVRGYWLTNKVPIKRPSTGLLMYT----LATRFCDE
Crigri_4        LKVRTAYPSLRLIHAVRGYWLTNKVPIKRPSTGLLMYT----LATRFCDE
Galgal_4        LKVRTAYPSLRLIHAVRGYWLTNKVHIKRPSTGLLMYT----LATRFCDE
Mesaur_4        LKVRTAYPSLRLIHAVRGYWLTNKVPIKRPSTGLLMYT----LATRFCDE
Homsap_4        LKVRTAYPSLRLIHAVRGYWLTNKVPIKRPSTGLLMYT----LATRFCDE
Notscuscu_4     LKVQTAYPSLRLIHAVRGYWLTNKVYIKRPSTGLLMYT----LATRFCDE
Psetextex_4     LKVRTAYPSLRLIHAVRGYWLTNKVYIKRPSTGLLMYT----LATRFCDE
Musmus_4        LQVRTAYPSLRLIHAVRGYWLTNKVPIKRPSTGLLMYT----LATRFCDE
Pogvit_4        LKVRTAYPSLRLIHAVRGYWLTNKVFIKRPSTGLLMYT----LATRFCDE
Promuc_4        LKVRTAYPSLRLIHAVRGYWLTNKVYIKRPSTGLLMYT----LATRFCDE
Pantro_4        LKVRTAYPSLRLIHAVRGYWLTNKVPIKRPSTGLLMYT----LATRFCDE
Pytbiv_4        LKVRTAYPSLRLIHAVRGYWLTNKVYIKRPSTGLLMYT----LATRFCDE
Ratnor_2        VNVRTAYPSLRLLHAVRGYWLTNKVHIKRPTTGLLMYT----LATRFCNQ
Ratnor_4        LKVRTAYPSLRLIHAVRGYWLTNKVPIKRPITGLLMYT----LATRFCDE
Siltro_2        INVHTAYPSLRLLHAVRGYWLTNKVHIKRPTTGILMYT----LATRFCNR
Siltro_4        LKVRTAYPSLRLIHAVRGYWLTNKVQIKRPSTGLLMYT----LATRFCDE
Susscr_4        LKVRTAYPSLRLIHAVRGYWLTNKVPIKRPSTGLLMYT----LATRFCDE
Taegut_4        LKVRTAYPSLRLIHAVRGYWLTNKVHIKRPSTGLLMYT----LATRFCDE
Termextri_4M    LKVRTAYPSLRLIHAVRGYWLTNKVHIKRPSTGLLMYT----LTTRFCDE
Thasirsir_4     LKVRTAYPSLRLIHAVRGYWLTNKVYIKRPSTGLLMYT----LATRFCDE
Xenlae_4B       LKVRTAYPSLRLIHAVRGYWLTNKVQIKRPSTGLLMYT----LATRFCDE
Xenlae_4A       LKVRTAYPSLRLIHAVRGYWLTNKVQIKRPSTGLLMYT----LATRFCDE
                201
Latcal_3        IHLYG----FWPFGWDPNTG---KELPYHYYDKKGTKFTTKWQESHQLPA
Perflu_3        IHLYG----FWPFGWDPNTG---KELPYHYYDKKGTKFTTKWQESHQLPA
Serdum_3        IHLYG----FWPFGWDPNTG---KELPYHYYDKKGTKFTTKWQESHQLPA
Stepar_3        IHLYG----FWPFGWDPNTG---KELPYHYYDKKGTKFTTKWQESHQLPA
```

```
Tetcal_3        IHLYG----FWPFAWDPNTG---KELPYHYYDKKGTKFTTKWQESHQLPA
Monalb_3        IHLYG----FWPFGWDPNTG---KELPYHYYDKKGTKFTTKWQESHQLPA
Molmol_3        IHLYG----FWPFGWDPNTG---KELPYHYYDKKGTKFTTKWQESHQLPA
Takrub_3        IHLYG----FWPFGWDPNTG---KELPYHYYDKKGTKFTTKWQESHQLPT
Canlupfam_3     IHLYG----FWPFGFDPNTR---EDLPYHYYDKKGTKFTTKWQESHQLPA
Bostau_3        IHLYG----FWPFGFDPNTR---EDLPYHYYDKKGTKFTTKWQESHQLPA
Homsap_3        IHLYG----FWPFGFDPNTR---EDLPYHYYDKKGTKFTTKWQESHQLPA
Siltro_3        IHLYG----FWPFGWDPNTG---KDLPYHYYDKKGTKFTTKWQESHQLPA
Anocar_3        IHLYG----FWPFGFNPNNR---EDLPYHYFDKKGTKFTTKWQESHQLPA
Musmus_3        IHLYG----FWPFGFDPNTR---EDLPYHYYDKKGTKFTTKWQESHQLPA
Pantro_3        IHLYG----FWPFGFDPNTR---EDLPYHYYDKKGTKFTTKWQESHQLPA
Ratnor_3        IHLYG----FWPFGFDPNTR---EDLPYHYYDKKGTKFTTKWQESHQLPA
Galgal_3        IHLYG----FWPFGFDPNTR---EDLPYHYYDKKGTKFTTKWQESHQLPA
Lepocu_3        IHLYG----FWPFGWDPNTG---KELPYHYYDKKGTKFTTKWQESHQLPT
Tetnig_3        IHLYG----FWPFGWDPNTG---RELPYHYYDKKGTKFTTKWQESHQLPT
Squaca_3        IHLYG----FWPFAWDPNTG---KELPYHYYDKKGTKFTTKWQESHQLPA
Perfla_3        IHLYG----FWPFGWDPNTG---KELPYHYYDKKGTKFTTKWQESHQLPA
Salsal_3        IHLYG----FWPFGWDPNTG---KELPYHYYDKKGTKFTTKWQESHQLPA
Serlaldor_3     IHLYG----FWPFGWDPNTG---KELPYHYYDKKGTKFTTKWQESHQLPA
Treber_3        IHLYG----FWPFGWDPNTG---NDLPYHYYDKKGTKFTTKWQETHQLPT
Cynsem_3        IHLYG----FWPFGWDPNTG---KELPYHYYDKKGTKFTTKWQESHQLPA
Paroli_3        IHLYG----FWPFGWDPNTG---KDLPYHYYDKKGTKFTTKWQETHQLPS
Paroli_3b       IHLYG----FWPFGWDPNTG---KELPYHYYDKKGTKFTTKWQESHQLPA
Plaste_3        IHLYG----FWPFGWDPNTG---NDLPYHYYDKKGTKFTTKWQETHQLPS
Plaste_3b       IHLYG----FWPFGWDPNTG---KELPYHYYDKKGTKFTTKWQESHQLPA
Erpcal3         IHLYG----FWPFGWDPNTG---KELPYHYYDKKGTKFTTKWQESHQLPT
Calmil_3b       IHLYG----FWPFAWDPNTG---RELPYHYYDKKGTKFNTNWQETHQLPA
Hetzeb_3        IHLYG----FWPFAWDPNTG---KELPYHYYDKKGTKFTTKWQESHQLPA
Latcha_3        IHLYG----FWPFGWDPNSG---KDLPYHYYDKKGTKFTTKWQESHQLPA
Anates_3        IHLYG----FWPFGWDPNTG---KELPYHYYDKKGTKFTTKWQESHQLPA
Ampcit3         IHLYG----FWPFGWDPNTG---KELPYHYYDKKGTKFTTKWQESHQLPA
Auslim_3        VHLYG----FWPFGWDPNTG---KELPYHYYDRKGTKFTTKWQESHQLPA
Cypvar_3        IHLYG----FWPFGWDPNTG---KELPYHYYDKKGTKFTTKWQESHQLPA
Krymar_3        VHLYG----FWPFGWDPNTG---KELPYHYYDRKGTKFTTKWQESHQLPA
Funhet_3        IHLYG----FWPFGWDPNTG---KELPYHYYDKKGTKFTTKWQESHQLPA
Gamaff3         IHLYG----FWPFGWDPNTG---KELPYHYYDKKGTKFTTKWQESHQLPA
Orylat_3        IHLYG----FWPFGWDPNTG---KELPYHYYDRKGTKFTTKWQESHQLPA
Orenil_3        IHLYG----FWPFGWDPNTG---KELPYHYYDKKGTKFTTKWQESHQLPA
Mayzeb_3        IHLYG----FWPFGWDPNTG---KELPYHYYDKKGTKFTTKWQESHQLPA
Punnye_3        IHLYG----FWPFGWDPNTG---KELPYHYYDKKGTKFTTKWQESHQLPA
Punnye_3a       IHLYG----FWPFGWDPNTG---KELPYHYYDKKGTKFTTKWQESHQLPA
Neobri_3        IHLYG----FWPFGWDPNTG---KELPYHYYDKKGTKFTTKWQESHQLPA
Misang_3        VHLYG----FWPFGWDPNTG---KELPYHYYDKKGTKFTTKWQESHQLPT
Sinrhi3         VHLYG----FWPFGWDPNTG---KELPYHYYDKKGTKFTTKWQESHQLPT
Singra_3        VHLYG----FWPFGWDPNTG---KELPYHYYDKKGTKFTTKWQESHQLPT
Hapbur_3        IHLYG----FWPFGWDPNTG---KELPYHYYDKKGTKFTTKWQESHQLPA
Astcal_3        IHLYG----FWPFGWDPNTG---KELPYHYYDKKGTKFTTKWQESHQLPA
Notfur_3        IHLYG----FWPFGWDPNTG---KELPYHYYDRKGTKFTTKWQESHQLPA
Cypcar_3        --------------------------------------------------
Poefor3         IHLYG----FWPFGWDPNTG---KELPYHYYDKKGTKFTTKWQESHQLPA
Poelat_3        IHLYG----FWPFGWDPNTG---KELPYHYYDKKGTKFTTKWQESHQLPA
Poeret_3        VHLYG----FWPFGWDPNTG---KELPYHYYDKKGTKFTTKWQESHQLPA
Angang_3        IHLYG----FWPFGWDPNTG---KELPYHYYDKKGTKFTTKWQESHQLPS
Xipmac_3        IHLYG----FWPFGWDPNTG---KELPYHYYDKKGTKFTTKWQESHQLPA
Masarm_3        IHLYG----FWPFGWDPNTG---KELPYHYYDKKGTKFTTKWQESHQLPA
Permag_3        -------------------------LPYHYYDKKGTKFTTKWQESHQLPA
Gnapet3         VHLYG----FWPFGWDPSTG---RELPYHYYDRKGTKFTTKWQESHQLPA
Ostbic_3        VHLYG----FWPFGWDPNTG---KELPYHYYDRKGTKFTTKWQETHELPT
Gadmor_3        IHLYG----FWPFGWDPNTG---KELPYHYYDKKGTKFTTKWQESHQLPA
Sclfor_3        VHLYG----FWPFGWDPNTG---KELPYHYYDRKGTKFTTKWRESHELPS
Acapol3         IHLYG----FWPFGWDPNTG---KELPYHYYDKKGTKFTTKWQESHQLPA
Ampoce_3        IHLYG----FWPFGWDPNTG---KELPYHYYDKKGTKFTTKWQESHQLPA
Sclfor_3b       IHLYG----FWPFAWDPISG---KGLPYHYYDKKGTKFTTKWQESHQLPA
Ostbic_3b       IHLYG----FWPFGWDPISG---KGLPYHYYDKKGTKFTTKWQESHQLPA
Parkin_3        VHLYG----FWPFGWDPSTG---RELPYHYYDRKGTKFTTKWQESHQLPA
Ampper3         IHLYG----FWPFGWDPNTG---KELPYHYYDKKGTKFTTKWQESHQLPA
Oncmyk_3        IHLYG----FWPFGWDPNTG---KELPYHYYDKKGTKFTTKWQESHQLPA
Gasacu_3        IHLYG----FWPFGWDPNTG---KELPYHYYDKKGTKFTTKWQESHQLPA
Labber_3        IHLYG----FWPFGWDPNTG---KELPYHYYDKKGTKFTTKWQESHQLPA
Panbuc_3        VHLYG----FWPFGWDPASG---RELPYHYYDKKGTKFTTKWQESHQLPA
Parhas3         VHLYG----FWPFGWDPNTG---KDLPYHYYDKKGTKFTTKWQESHQLPT
Cluhar_3        IHLYG----FWPFGWDPNTG---KELPYHYYDKKGTKFTTKWQESHQLPT
Aloalo_3        IHLYG----FWPFGWDPNTG---KELPYHYYDKKGTKFTTKWQESHQLPT
Panhyp_3        VHLYG----FWPFGWDPNTG---KELPYHYYDKKGTKFTTKWQESHQLPT
Eleele_3        VHLYG----FWPFGWDPNTG---KELPYHYYDKKGTKFTTKWQESHQLPT
Denclu_3        IHLYG----FWPFGWDPNTG---KELPYHYYDKKGTKFTTKWQESHQLPT
Sarpil_3        IHLYG----FWPFGWDPNTG---KELPYHYYDKKGTKFTTKWQESHQLPT
```

Eigvir_3	VHLYG----FWPFGWDPNTG---KELPYHYYDKKGTKFTTKWQESHQLPT
Aptalb_3	VHLYG----FWPFGWDPNTG---KDLPYHYYDKKGTKFTTKWQESHQLPT
Sinans_3	VHLYG----FWPFGWDPNTG---KELPYHYYDKKGTKFTTKWQESHQLPT
Pygnat_3	IHLYG----FWPFGWDPNTG---KELPYHYYDKKGTKFTTKWQESHQLPT
Astmex_3	IHLYG----FWPFGWDPNTG---KELPYHYYDKKGTKFTTKWQESHQLPT
Caraur_3	VHLYG----FWPFGWDPNTG---KELPYHYYDKKGTKFTTKWQESHQLPT
Danrer_3	VHLYG----FWPFGWDPNTG---KELPYHYYDKKGTKFTTKWQESHQLPT
Ictpun_3	IHLYG----FWPFGWDPNTG---KELPYHYYDKKGTKFTTKWQESHQLPT
Plealt_3	IHLYG----FWPFGWDPNTG---KELPYHYYDKKGTKFTTKWQESHQLPA
Plealt_9	IHLYG----FWPFGWDPNTG---KELPYHYYDKKGTKFTTKWQETHQLPS
Gasacu_9	IHLYG----FWPFGWDPNTG---NDLPYHYYDKKGTKFTTKWQETHQLPS
Parkin_9	VHLYG----FWPFGWDPISG---KGLPYHYFDKKGTKFTTKWQESHQLPA
Acapol_9	IHLYG----FWPFGWDPNTG---KELPYHYYDKKGTKFTTKWQETHQLPS
Larcro_9	IHLYG----FWPFGWDPNTG---KDLPYHYYDKKGTKFTTKWQETHQLPS
Notcor_9	IHLYG----FWPFGWDPNTG---NDLPYHYYDKKGTKFTTKWQETHQLPT
Latcal_9	IHLYG----FWPFGWDPNTG---KELPYHYYDKKGTKFTTKWQETHQLPS
Serlaldor_9	IHLYG----FWPFGWDPNTG---KELPYHYYDKKGTKFTTKWQETHQLPS
Monalb_9	VHLYG----FWPFGWDPNTG---KELPYHYYDKKGTKFTTKWQEAHQLPS
Serdum_9	IHLYG----FWPFGWDPNTG---KELPYHYYDKKGTKFTTKWQETHQLPS
Perflu_9	IHLYG----FWPFGWDPNSG---KDLPYHYYDKKGTKFTTKWQETHQLPS
Perfla_9	IHLYG----FWPFGWDPNSG---KDLPYHYYDKKGTKFTTKWQETHQLPS
Stepar_9	IHLYG----FWPFGWDPNTG---KELPYHYYDKKGTKFTTKWQETHQLPS
Molmol_9	IHLYG----FWPFGWDPNTG---NDLPYHYYDKKGTKFTTKWQETHQLPS
Hipcom_9	IHLYG----FWPFGWDPNTG---KDLPYHYYDKKGTKFTTKWQETHQLPS
Takrub_9 B	IHLYG----FWPFGWDPNTG---KELPYHYYDKKGTKFTTKWQETHQLPT
Takrub_9	IHLYG----FWPFGWDPNTG---KELPYHYYDKKGTKFTTKWQETHQLPT
Cynsem_9	IHLYG----FWPFGWDPNTG---KDLPYHYYDKKGTKFTTKWQETHQLPS
Scomax_9	IHLYG----FWPFGWDPNTG---KDLPYHYYDKKGTKFTTKWQETHQLPS
Corlav_9	IHLYG----FWPFGWDPNTG---KELPYHYYDKKGTKFTTKWQETHQLPS
Oncmyk_9	IHLYG----FWPFGWDPNTG---KELPYHYYDKKGTKFTTKWQETHQLPS
Salsal_9	IHLYG----FWPFGWDPNTG---KELPYHYYDKKGTKFTTKWQESHQLPA
Onctsh_9	IHLYG----FWPFGWDPNTG---KELPYHYYDKKGTKFTTKWQETHQLPS
Salalp_9	IHLYG----FWPFGWDPNTG---KELPYHYYDKKGTKFTTKWQETHQLPS
Ampoce_9	IHLYG----FWPFGWDPNTG---KELPYHYYDKKGTKFTTKWQETHQLPS
Ampper9	IHLYG----FWPFGWDPNTG---KELPYHYYDKKGTKFTTKWQETHQLPS
Labber_9	IHLYG----FWPFGWDPNTG---KDLPYHYYDKKGTKFTTKWQETHQLPT
Umbpyg_3B	IHLYG----FWPFGWDPNTG---KELPYHYYDKKGTKFTTKWQESHQLPA
Esoluc_3b	IHLYG----FWPFGWDPNTG---KELPYHYYDRKGTKFTTKWQESHQLPA
Gadmor_9	IHLYG----FWPFGWDPNTG---KDLPYHYYDKKGTKFTTKWQETHQLPS
Umbpyg_9	IHLYG----FWPFGWDPNTG---KELPYHYYDKKGTKFTTKWQETHQLPS
Esoluc_9	IHLYG----FWPFGWDPNTG---KELPYHYYDKKGTKFTTKWQETHQLPS
Permag_9	IHLYG----FWPFGWDPNTG---KDLPYHYYDRKGTKFTTKWQETHQLPN
Bolpec_9	IHLYG----FWPFGWDPNTG---KDLPYHYYDRKGTKFTTKWQETHQLPN
Poefor9	IHLYG----FWPFGWDPNTG---KELPYHYYDKKGTKFTTKWQETHQLPS
Masarm_9	IHLYG----FWPFGWDPNTG---KELPYHYYDKKGTKFTTKWQETHQLPS
Angjap_9	IHLYG----FWPFGWDPNTG---KELPYHYYDKKGTKFTTKWQESHQLPS
Poelat_9	IHLYG----FWPFGWDPNTG---KELPYHYYDKKGTKFTTKWQETHQLPS
Poeret_9	IHLYG----FWPFGWDPNTG---KELPYHYYDKKGTKFTTKWQETHQLPS
Xipmac_9	IHLYG----FWPFGWDPNTG---KDLPYHYYDKKGTKFTTKWQETHQLPS
Hapbur_9	IHLYG----FWPFGWDPNTG---KDLPYHYYDKKGTKFTTKWQETHQLPS
Orylat_9	IHLYG----FWPFGWDPNTG---KDLPYHYYDKKGTKFTTKWQETHQLPS
Gamaff_9	IHLYG----FWPFGWDPNTG---KDLPYHYYDKKGTKFTTKWQETHQLPS
Neobri_9	IHLYG----FWPFGWDPNTG---KDLPYHYYDKKGTKFTTKWQETHQLPS
Funhet_9	IHLYG----FWPFGWDPNTG---KELPYHYYDKKGTKFTTKWQETHQLPS
Punnye_9	IHLYG----FWPFGWDPNTG---KDLPYHYYDKKGTKFTTKWQETHQLPS
Cypvar_9	IHLYG----FWPFGWDPNTG---KELPYHYYDKKGTKFTTKWQETHQLPS
Krymar_9	IHLYG----FWPFGWDPNTG---KELPYHYYDKKGTKFTTKWQETHQLPS
Notfur_9	IHLYG----FWPFGWDPNTG---KELPYHYYDKKGTKFTTKWQETHQLPS
Notpie_9	IHLYG----FWPFGWDPNTG---KELPYHYYDKKGTKFTTKWQETHQLPS
Orenil_9	IHLYG----FWPFGWDPNTG---KDLPYHYYDKKGTKFTTKWQETHQLPS
Auslim_9	IHLYG----FWPFGWDPNTG---KELPYHYYDKKGTKFTTKWQETHQLPS
Anates_9	IHLYG----FWPFGWDPNTG---KDLPYHYYDKKGTKFTTKWQETHQLPS
Astcal_9	IHLYG----FWPFGWDPNTG---KDLPYHYYDKKGTKFTTKWQETHQLPS
Mayzeb_9	IHLYG----FWPFGWDPNTG---KDLPYHYYDKKGTKFTTKWQETHQLPS
Orymel_9	IHLYG----FWPFGWDPNTG---KELPYHYYDKKGTKFTTKWQETHQLPS
Ampcit9	IHLYG----FWPFGWDPNTG---KDLPYHYYDKKGTKFTTKWQETHQLPS
Macfas_2	IYLYG----FWPFPLDQN-Q---NPVKYHYYDSLKYGYTSQASP-HTMPL
Macmul_2	IYLYG----FWPFPLDQN-Q---NPVKYHYYDSLKYGYTSQASP-HTMPL
Macnem_2	IYLYG----FWPFPLDQN-Q---NPVKYHYYDSLKYGYTSQASP-HTMPL
Musmus_2	IYLYG----FWPFPLDQN-Q---NPVKYHYYDSLKYGYTSQASP-HTMPL
Ponabe_2	IYLYG----FWPFPLDQN-Q---NPVKYHYYDSLKYGYTSQASP-HTMPL
Papanu_2	IYLYG----FWPFPLDQN-Q---NPVKYHYYDSLKYGYTSQASP-HTMPL
Nomleu_2	IYLYG----FWPFPLDQN-Q---NPVKYHYYDSLKYGYTSQASP-HTMPL
Pantro_2	IYLYG----FWPFPLDQN-Q---NPVKYHYYDSLKYGYTSQASP-HTMPL
Latcha_2	IHLYG----FWPFPRDHS-E---RPVKYHYYDSLTYEYTSQASP-HTMPL
Gnapet2	IHLYG----FWPFALGPQ-G---NPVKYHYYDSLTYQYTSQASP-HTMPL
Panbuc_2b	IHLYG----FWPFPRDVG-G---RPVKYHYYDALTYGYTSQAGP-HAMPL

```
Ostbic_2        IHLYG----FWPFPLGPH-G---NPVKYHYYDALTYEYTSQVGP-HTMPL
Sinrhi2         IHLYG----FWPFAHDPD-G---KSVKYHYYDTLTYHYTSGASP-HTMPL
Hetzeb_2        IHLYG----FWPFPKDQK-G---NVVKYHYYDSLTYEYNSRATP-HTMPL
Hipcom_2        IHLYG----FWPFSHDAA-G---KAVKYHYYDTLKYEYTSSSSP-HSMPL
Prigla_2        IHLYG----FWPFPKDQK-G---NVVKYHYYDSLTYEYNSRATP-HTMPL
Scytor_2        IHLYG----FWPFPKDQK-G---NAVKYHYYDSLTYEYNSRATP-HTMPL
Tetcal_2        IHLYG----FWPFSKDPK-G---NAVKYHYYDSLTYEYNSRATP-HTMPL
Rhityp_2        IHLYG----FWPFPKDHK-G---NAVKYHYYDSLTYEYNSRATP-HTMPL
Parkin_2b       IHLYG----FWPFSHDAQ-G---NAVKYHYYDSLTYGYTSRTSP-HTMPL
Angang_2        IHLYG----FWPFPQDPQ-G---NPMKYHYYDTLTYEYDSHSSP-HTMPL
Pygnat_2        IHLYG----FWPFPRDSE-G---KPVKYHYYDTLTYQYTSSSSP-HTMPL
Parkin_2        IHLYG----FWPFALGPQ-G---NPVKYHYYDSLTYEYTSQASP-HTMPL
Gorgor_2        IYLYG----FWPFPLDQN-Q---NPVKYHYYDSLKYGYTSQASP-HTMPL
Thegel_2        IYLYG----FWPFPLDQN-Q---NPVKYHYYDSLKYGYTSQASP-HTMPL
Tupchi_2        IYLYG----FWPFPLDQN-Q---NPVKYHYYDSLKYGYTSQASP-HTMPL
Galgal_2        IHLYG----FWPFPLDQN-Q---QPVKYHYYDSLKYGYTSQASP-HTMPL
Eleele_2        IHLYG----FWPFSHGPE-G---KPVKYHYYDALTYQYTSSSSP-HTMPL
Danrer_2        IHLYG----FWPFAHDPD-G---KPVKYHYYDTLTYHYTSSASP-HTMPL
Canlupfam_2     IYLYG----FWPFPLDQN-Q---NPVKYHYYDSLKYGYTSQASP-HTMPL
Aloalo_2        IHLYG----FWPFHRDHQ-G---KRVKYHYYDTLTYEFTSRASP-HTMPL
Astmex_2        IHLYG----FWPFPRDSE-G---KPVKYHYYDTLKYQYTSSSSP-HTMPL
Sinans_2        IHLYG----FWPFAHDPD-G---KPVKYHYYDTLTYHYTSSASP-HTMPL
Sclfor_2        IHLYG----FWPFLLGPH-G---NPVKYHYYDALTYEYTSQVGP-HTMPL
Ceraty_2        IYLYG----FWPFPLDQN-Q---NPVKYHYYDSLKYGYTSQASP-HTMPL
Anocar_2        IYLYG----FWPFPRDQD-Q---NPVKYHYYDTLKYGYMSQTSP-HTMPL
Anates_2        IHLYG----FWPFPLDPQ-G---KPVKYHYYDALKYEYTSSSSP-HTMPL
Ampbic_2        IHLYG----FWPFPLDPH-G---KPVKYHYYDTLKYEYTSSSSP-HTMPL
Sarpil_2        IHLYG----FWPFHRDHQ-G---KRVKYHYYDTLTYQFTSRASP-HTMPL
Xenlae_2        IYLYG----FWPFPRDLH-Q---NPVKYHYYDSLKYGYTSQAGP-HAMPL
Cluhar_2        IHLYG----FWPFHRDHQ-G---NVVKYHYYDTLTYEFTSRASP-HTMPL
Cypcar_2        IHLYG----FWPFAHDPD-G---KPVKYHYYDMLTYHYTSSASP-HTMPL
Denclu_2        IHLYG----FWPFHRDAQ-G---KPVKYHYYDALTYEYTSHSSP-HTMPL
Notpie_2        IHLYG----FWPFPLDPK-G---KTVKYHYYDTLKYEYTSSSSP-HTMPL
Erpcal2         IHLYG----FWPFLQDQK-G---NSVKYHYYDSLRYEFDSQSSP-HAMPL
Amical_2        IHLYG----FWPFSRGPA-G---NPVKYHYYDSLTYEYTAQSSP-HTMPL
Lepocu_2        IHLYG----FWPFSLGPN-G---LPVKYHYYDSLTYEYRAQSSP-HSMPV
Homsap_2        IYLYG----FWPFPLDQN-Q---NPVKYHYYDSLKYGYTSQASP-HTMPL
Bostau_2        IYLYG----FWPFPLDQN-Q---NPVKYHYYDSLKYGYTSQASP-HTMPL
Caraur_2        IHLYG----FWPFAHDPD-G---KPVKYHYYDTLTYHYTSSASP-HTMPL
Thythy_2B       IHLYG----FWPFPKDSQ-G---KPVKYHYYDTLTYEYTSHASP-HTMPL
Onctsh_2B       IHLYG----FWPFPQDSQ-G---KPVKYHYYDTLTYEYTSHASP-HTMPL
Salsal_2B       IHLYG----FWPFPQDSQ-G---KSVKYHYYDTLTYEYTSHASP-HTMPL
Oncmyk_2B       IHLYG----FWPFPQDSQ-G---KPVKYHYYDTLTYEYTSHASP-HTMPL
Onckis_2B       IHLYG----FWPFPQDSQ-G---KPVKYHYYDTLTYEYTSHASP-HTMPL
Salsal_2A       IHLYG----FWPFPRDSQ-G---KPVKYHYYDTLTYEYTSHASP-HAMPL
Thythy_2A       IHLYG----FWPFPRNSQ-G---KPVKYHYYDTLTYEYTSHASP-HTMPL
Salalp_2A       IHLYG----FWPFPRDSQ-G---KPVKYHYYDTLTYEYTSHASP-HTMPL
Corlav_2A       IHLYG----FWPFPRDSQ-G---IPVKYHYYDTLTYEYTSHASP-HTMPL
Cormar_2A       IHLYG----FWPFPRDSQ-G---IPVKYHYYDTLTYEYTSHASP-HTMPL
Onctsh_2A       IHLYG----FWPFPRDSQ-G---KPVKYHYYDTLTYEYTSHASP-HTMPL
Oncmyk_2A       IHLYG----FWPFPQDSQ-G---KPVKYHYYDTLTYEYTSHASP-HTMPL
Neobri_2        IHLYG----FWPFPLDPQ-G---KPVKYHYYDTLKYEYTSSSSP-HTMPL
Takrub_2        IHLYG----FWPFPSDPH-G---RPVKYHYYDTLKYEYTSSSSP-HTMPL
Tetnig_2        IHLYG----FWPFPLDPR-G---RPVKYHYYDTLKYEYTSSSSP-HTMPL
Orenil_2        IHLYG----FWPFPLDPQ-G---KPVKYHYYDTLKYEYTSSSSP-HTMPL
Orylat_2        IHLYG----FWPFPVDSH-G---KAVKYHYYDSLKYEYTSSSSP-HTMPL
Krymar_2        IHLYG----FWPFALDPR-G---RPVKYHYYDALKYEYTSSSSP-HAMPL
Notfur_2        IHLYG----FWPFPLDPK-G---KTVKYHYYDTLKYEYTSSSSP-HTMPL
Poelat_2        IHLYG----FWPFPLGPQ-G---RPVKYHYYDTLKYEYTSSSSP-HTMPL
Gadmor_2        IHLYG----FWPFPLDPR-G---RPVKYHYYDTLKYDYTSHSSP-HTMPL
Xipmac_2        IHLYG----FWPFPLGPQ-G---RPVKYHYYDTLKYEYTSSSSP-HTMPL
Poeret_2        IHLYG----FWPFPLGPQ-G---RPVKYHYYDTLKYEYTSSSSP-HTMPL
Astcal_2        IHLYG----FWPFSLDPQ-G---KPVKYHYYDTLKYEYTSSSSP-HTMPL
Hapbur_2        IHLYG----FWPFSLDPQ-G---KPVKYHYYDTLKYEYTSSSSP-HTMPL
Acapol_2B       IHLYG----FWPFPLDPH-G---KPVKYHYYDTLKYEYTSSSSP-HTMPL
Ampoce_2A       IHLYG----FWPFPLDPH-G---NPVKYHYYDTLKYEYTSSSSP-HTMPL
Acapol_2A       IHLYG----FWPFPLDPH-G---KPVKYHYYDTLKYEYTSSSSP-HTMPL
Mayzeb_2        IHLYG----FWPFSLDPQ-G---KPVKYHYYDTLKYEYTSSSSP-HTMPL
Punnye_2        IHLYG----FWPFSLDPQ-G---KPVKYHYYDTLKYEYTSSSSP-HTMPL
Latcal_2        IHLYG----FWPFPLDPQ-G---KPVKYHYYDTLKYEYTSSSSP-HTMPL
Singra_2        IHLYG----FWPFAHDPD-G---KPVKYHYYDTLTYHYTSSASP-HTMPL
Auslim_2        IHLYG----FWPFPLDPR-G---RPVKYHYYDTLKYEYTSSSSP-HTMPL
Monalb2         IHLYG----FWPFPLDLQ-G---KPVKYHYYDTLKYEYTSSSSP-HTMPL
Ampoce_2B       IHLYG----FWPFPLDPH-G---NPVKYHYYDTLKYEYTSSSSP-HTMPL
Chiham_2        IHLYG----FWPFSHDPQ-G---KPVKYHYYDTLKYEYTSSSSP-HTMPL
Gasacu_2        IHLYG----FWPFRHDPQ-G---RPVKYHYYDALKYEYTSSSSP-HTMPL
Gymacu_2        IHLYG----FWPFSHDPQ-G---KPVKYHYYDTLKYEYTSSSSP-HTMPL
```

```
Labber_2        IHLYG----FWPFPLDPQ-G---RPVKYHYYDTLKYEYTSSASP-HTMPL
Cypvar_2        IHLYG----FWPFPLDPQ-G---RPVKYHYYDTLKYEYTSSSSP-HTMPL
Funhet_2        IHLYG----FWPFPLGPH-G---RPVKYHYYDTLKYQYTSSSSP-HTMPL
Larcro_2        IHLYG----FWPFPLDPQ-G---KPVKYHYYDTLKYEYTSSSSP-HTMPL
Paroli_2        IHLYG----FWPFALDPQ-G---KPVKYHYYDTLKYEYTSRSSP-HSMPL
Perflu_2        IHLYG----FWPFPLDPQ-G---RSVKYHYYDTLKYEYTSSSSP-HTMPL
Serdum_2        IHLYG----FWPFALDPQ-G---KPVKYHYYDTLKYEYTSSSSP-HTMPL
Stepar_2        IHLYG----FWPFPLDPQ-G---KPVKYHYYDTLKYEYTSSSSP-HTMPL
Notcor_2        IHLYG----FWPFSHDPQ-G---KPVKYHYYDTLKYEYTSSSSP-HTMPL
Treber_2        IHLYG----FWPFSHDPQ-G---KPVKYHYYDTLKYEYTSSSSP-HTMPL
Serlaldor_2     IHLYG----FWPFALDPQ-G---KPVKYHYYDTLKYEYTSSSSP-HTMPL
Plaste_2        IHLYG----FWPFALDPQ-G---KPVKYHYYDTLKYEYTSRSSP-HTMPL
Clabat_4        IHLYG----FWPFPKDNS-G---NPVKYHYYDMLKYRYFSNAGP-HRMPL
Ictpun_4A       IHLYG----FWPFPKDSS-G---NPVKYHYYDMLKYRYFSNASP-HRMPL
Panhyp_4        IHLYG----FWPFPKDAS-G---NPVKYHYYDMLKYRYFSNASP-HRMPL
Umbpyg_4        IHLYG----FWPFPRDAK-G---NAVKYHYYDMLKYRYFSNAGP-HRMPL
Esoluc_4        IHLYG----FWPFPRDAN-G---NVVKYHYYDMLKYSYFSNAGP-HRMPL
Plealt_4        IHLYG----FWPFPRDAN-G---NLVKYHYYDMLKYRYFSNAGP-HRMPL
Onckis_4B       IHLYG----FWPFPRDAN-G---NMVKYHYYDMLKYRYFSNASP-HRMPL
Onckis_4A       IHLYG----FWPFPRDAN-G---NMVKYHYYDMLKYRYFSNASP-HRMPL
Onctsh_4        IHLYG----FWPFPRDAN-G---NMVKYHYYDMLKYRYFSNASP-HRMPL
Oncmyk_4        IHLYG----FWPFPRDAN-G---NMVKYHYYDMLKYRYFSNAGP-HRMPL
Corlav_4        IHLYG----FWPFPRDSN-G---NVVKYHYYDLLKYRYFSNAGP-HRMPL
Astmex_4A       IHLYG----FWPFPKDAN-G---IPVKYHYYDLLKYRYFSSAGP-HRMPL
Pygnat_4        IHLYG----FWPFPKDAA-G---NPVKYHYYDTLKYRYFSRAGP-HRMPL
Aloalo_4        IHLYG----FWPFPKDTH-G---NPVKYHYYDTLKYRYFSNAGP-HRMPL
Cluhar_4        IHLYG----FWPFPKDSQ-G---NPVKYHYYDTIKYRYFSNAGP-HRMPL
Denclu_4        IHLYG----FWPFPRDTQ-G---NPVKYHYYDMLKYRYLSNAAP-HRMPL
Konpun4         IHLYG----FWPFPKDSQ-G---NTVKYHYYDTLKYRYFSNAGP-HRMPL
Sarpil_4        IHLYG----FWPFPKDTH-G---NPVKYHYYDTLKYRYFSNAGP-HRMPL
Caraur_4        IHLYG----FWPFPRDGS-G---NPVKYHYYDGLRYRYFSNAGP-HRMPL
Cteide_4        IYLYG----FWPFPKDAN-G---NPVKYHYYDTLKYRYFSNVGP-HRMPL
Petmar_4        IHLYG----FWPFQYNIA-G---KPVRYHYYEGLRYRYRSRAGP-HTMPL
Cypcar_4        IHLYG----FWPFPKDGN-G---NPVKYHYYDGLKYRYFSNAGP-HRMPL
Danrer_4        IYLYG----FWPFPKDAS-G---NPVQYHYFDGLKYRYFSNAGP-HRMPL
Misang_4        IHLYG----FWPFPKDGN-G---NPVNYHYYDTLKYRYFSNAGP-HRMPL
Angjap_4        IHLYG---------------------------------------------
Cypcar_4A       IHLYG----FWPFPKDGN-G---NPVKYHYYDGLKYRYFSNAGP-HRMPL
Angang_4        IHLYG----FWPFPKDLG-G---NPVKYHYYDALKYRYFSKASP-HRMPL
Cypcar_4B       IHLYG----FWPFPKDGN-G---NPVKYHYYDGLRYRYFSNASP-HRMPL
Sinans_4A       IHLYG----FWPFPKDGN-R---NTVKYHYYDGLKYRYFSSAGP-HRMPL
Sinans_4B       IYLYG----FWPFPKDGN-G---NPVKYHYYDGLRYRYFSNAGP-HRMPL
Singra_4A       IHLYG----FWPFPKDGN-R---NPVKYHYYDGLKYRYFSSAGP-HRMPL
Aptalb_4        IHLYG----FWPFPKDAE-G---NPVKYHYYDSLKYRYFSSAGP-HRMPL
Eigvir_4        IHLYG----FWPFPKDAE-G---NPVKYHYYDTLKYRYFSSAGP-HRMPL
Eleele_4        IHLYG----FWPFPKDAK-G---NLVKYHYYDTIKYRYFSNAGP-HRMPL
Parhas_4        IHLYG----FWPFPKDAE-G---NPVKYHYYDSLKYRYFSSAGP-HRMPL
Sclfor_4        IHLYG----FWPFPKDSK-G---NAVKYHYYDALKYRYFSNASP-HKMPL
Gnapet4         IHLYG----FWPFPKDSS-G---NPVKYHYYDKLKYRYFSNAGP-HRMPL
Ostbic_4        IHLYG----FWPFPKDSK-G---NMVKYHYYDTLKYRYSSKAGP-HRMPL
Parkin_4        IHLYG----FWPFPKDSS-G---NPVKYHYYDKLKYRYFSNAGP-HRMPL
Panbuc_4        IHLYG----FWPFPKDLQ-G---NPVKYHYYDRLKYRYFSKAGP-HRMPL
Erpcal4         IHLYG----FWPFPKDSI-G---KPVKYHYYDELKYRYFSNASP-HRMPL
Salsal_4        IHLYG----FWPFPRDAN-G---NMVKYHYYDMLKYRYFSNAGP-HRMPL
Saltru_4        IHLYG----FWPFPRDAN-G---NMVKYHYYDMLKYRYFSNAGP-HRMPL
Salalp_4        IHLYG----FWPFPRDAN-G---KIVKYHYYDMLKYRYFSNAGP-HRMPL
Salfon_4        IHLYG----FWPFPRDAN-G---KIVKYHYYDMLKYRYFSNAGP-HRMPL
Thythy_4        IHLYG----FWPFPRDAN-G---NVVKYHYYDMLKYRYFSNAGP-HRMPL
Calmil_4        IHLYG----FWPFPKDAN-G---TPVKYHYYDNLKYRYFSNAGP-HRMPL
Hetzeb_4        IHLYG----YWPFPKDAK-G---TPVKYHYYDNLKYRYFSNVGP-HRMPL
Rhityp_4        IHLYG----YWPFPKDSK-G---VPVKYHYYDNLKYRYFSNAGP-HRMPL
Scytor_4        IHLYG----YWPFPKDSK-G---TPVKYHYYDNLKYRYFSNAGP-HRMPL
Squaca_4        IHLYG----YWPFPKDAK-G---APVKYHYYDNLKYRYFSNAGP-HRMPL
Amical_4        IHLYG----FWPFPKDTK-G---RLVKYHYYDELKYRYFSNASP-HRMPL
Lepocu_4        IHLYG----FWPFPKDSQ-G---RPVKYHYYDELKYRYFSNASP-HRMPL
Agema_4         IHLYG----FWPFPKDAS-G---NPVKYHYYDMLKYRYFSNASP-HRMPL
Anocar_4        IHLYG----FWPFPRDIN-G---KTVKYHYYDDLKYRYFSNASP-HRMPL
Canlupfam_4     IHLYG----FWPFPKDLN-G---KAVKYHYYDDLKYRYFSNASP-HRMPL
Crigri_4        IHLYG----FWPFPKDLN-G---KAVKYHYYDDLKYRYFSNASP-HRMPL
Galgal_4        IHLYG----FWPFPKDLH-G---KPVKYHYYDDLKYRYFSNASP-HRMPL
Mesaur_4        IHLYG----FWPFPKDLN-G---KAVKYHYYDDLKYRYFSNASP-HRMPL
Homsap_4        IHLYG----FWPFPKDLN-G---KAVKYHYYDDLKYRYFSNASP-HRMPL
Notscuscu_4     IHLYG----FWPFPKDIN-G---KPVKYHYYDDLKYRYFSNASP-HRMPL
Psetextex_4     IHLYG----FWPFPKDIN-G---KPVKYHYYDDLKYRYFSNASP-HRMPL
Musmus_4        IHLYG----FWPFPKDLN-G---KAVKYHYYDDLKYRYFSNASP-HRMPL
Pogvit_4        IHLYG----FWPFPKDMN-G---KPVKYHYYDDLKYRYFSNASP-HRMPL
Promuc_4        IHLYG----FWPFPKDIN-G---KPVKYHYYDDLKYRYFSNASP-HRMPL
```

```
Pantro_4       IHLYG----FWPFPKDLN-G---KAVKYHYYDDLKYRYFSNASP-HRMPL
Pytbiv_4       IHLYG----FWPFPKDIN-G---KPVKYHYYDDLKYRYFSNASP-HRMPL
Ratnor_2       IYLYG----FWPFPLDQN-Q---NPVKYHYYDSLKYGYTSQASP-HTMPL
Ratnor_4       IHLYG----FWPFPKDLN-G---KAVKYHYYDDLKYRYFSNASP-HRMPL
Siltro_2       IYLYG----FWPFPRDLH-Q---NPVKYHYYDSLKYGYTSQAGP-HAMPL
Siltro_4       IHLYG----FWPFPKDVY-G---NQVKYHYYDELKYKYFSNAGP-HRMPL
Susscr_4       IHLYG----FWPFPKDLN-G---KAVKYHYYDDLKYRYFSNASP-HRMPL
Taegut_4       IHLYG----FWPFPKDLH-G---KPVKYHYYDDLKYRYFSNASP-HRMPL
Termextri_4M   IHLYG----FWPFPKDFY-G---KPVKYHYYDDLKYRYFSNASP-HRMPL
Thasirsir_4    IHLYG----FWPFPKDIN-G---KPVKYHYYDDLKYRYFSNASP-HRMPL
Xenlae_4B      IYLYG----FWPFTKDVY-G---NQVKYHYYDELKYKYFSNAGP-HRMPL
Xenlae_4A      IHLYG----FWPFPKDVH-G---NQVKYHYYDELKYKYFSNAGP-HRMPL
               251
Latcal_3       EFKLLYKMHTEGLLKLTLSHCA---
Perflu_3       EFKLLYKMHTEGLLKLSLSHCA---
Serdum_3       EFKLLYKMHMEGLLKLSLSHCA---
Stepar_3       EFKLLYKMHTEGLLKLSLSHCA---
Tetcal_3       EFKLLFKLHTEGLTKLTLSHCA---
Monalb_3       EFKLLYKMHMEGLLKLTLSRCA---
Molmol_3       EFKLLYRMHTEGLLKLTLSHCA---
Takrub_3       EFKLLYKMHREGLLKLSLSHCA---
Canlupfam_3    EFQLLYRMHGEGLTKLTLSHCA---
Bostau_3       EFQLLYRMHGEGLTKLTLSHCA---
Homsap_3       EFQLLYRMHGEGLTKLTLSHCA---
Siltro_3       EFKLLYKMHREGLTKLTLSQCA---
Anocar_3       EFQQLFRMHSEGLAKLTLSHCA---
Musmus_3       EFQLLYRMHGEGLTKLTLSHCA---
Pantro_3       EFQLLYRMHGEGLTKLTLSHCA---
Ratnor_3       EFQLLYRMHGEGLTKLTLSHCA---
Galgal_3       EFQLLYRMHGEGLAKLTLSRCA---
Lepocu_3       EFKLLYKMHTEGLTKLSLSHCA---
Tetnig_3       EFKLLYKMHREGVLKLSLSHCG---
Squaca_3       EFKLLFKLHTDGLTKLTLSHCA---
Perfla_3       EFKLLYKMHTEGLLKLSLSHCA---
Salsal_3       EFKLLYRMHTEGLIKLTLSHCA---
Serlaldor_3    EFRLLYKMHMEGLLKLSLSHCA---
Treber_3       EFKLLYKLHREGVIKLSVTHCS---
Cynsem_3       EFKLLYKMHTEGLLKLSLSHCA---
Paroli_3       EFKLLYKLHREGVTKLSLTHCT---
Paroli_3b      EFKLLYKMHTEGXLKLSLSHCA---
Plaste_3       EFKLLYQLHREGVTKLSLTHCA---
Plaste_3b      EFKLLYKMHTEGLLKLRLSHCG---
Erpcal3        EFKLLYKMHEEGLTKLTLSHCA---
Calmil_3b      EFKILFKMHTAGLTKLSLSPCV---
Hetzeb_3       EFKLLFKLHTDGLTKLTLSHCA---
Latcha_3       EFKLLYKMHGEGLTKLTLSHCA---
Anates_3       EFKLLYKMHMEGLLKLTLSHCA---
Ampcit3        EFKLLYKMHTEGLLKLSVSHCA---
Auslim_3       EFKLLYKMHMDGLLKLTLSHCA---
Cypvar_3       EFKLLYKMHTEGLLKLSLSHCA---
Krymar_3       EFKLLYRMHTDGLLKLTLSHCA---
Funhet_3       EFKLLYQMHTEGLLKLSLSHCA---
Gamaff3        EFKLLYKMHTEGLLKLSLSHCA---
Orylat_3       EFKLLHKMHTEGLLKLTLSHCA---
Orenil_3       EFKLLYKMHTEGLLKLSLSHCA---
Mayzeb_3       EFKLLYKMHTEGLLKLSLSHCA---
Punnye_3       EFKLLYKMHTEGLLKLSLSHCA---
Punnye_3a      EFKLLYKMHTEGLLKLSLSHCA---
Neobri_3       EFKLLYKMHTEGLLKLSLSHCA---
Misang_3       EFKLLFKMHTEGILKLSLSHCT---
Sinrhi3        EFKLLFKMHADGVLKLSLSHCT---
Singra_3       EFKLLFKMHADGVLKLSLSHCA---
Hapbur_3       EFKLLYKMHTEGLLKLSLSHCA---
Astcal_3       EFKLLYKMHTEGLLKLSLSHCA---
Notfur_3       EFKLLYKMHTDGVLKLSLSHCA---
Cypcar_3       -------------------------
Poefor3        EFKLLYKMHTEGLLKLSLSHCA---
Poelat_3       EFKLLYKMHTEGLLKLSLSHCA---
Poeret_3       EFKLLHRMHSEGLLKLSLSHCA---
Angang_3       EFKLLYKMHTDGVIKLSLSHCA---
Xipmac_3       EFKLLYKMHTEGLLKLSLSHCA---
Masarm_3       EFKLLYKMHMEGLLKLTLSHCA---
Permag_3       EFKLLYRMHTEGLLKLSLSHCA---
Gnapet3        EFKQLYKMHAEGLTKLSLSHCI---
Ostbic_3       EFKLLYKMHEEGLTKLSLARCA---
Gadmor_3       EFKLLYKMHTKGLLKLNLSHCA---
Sclfor_3       EFKLLYKMHEQGLAKLSLARCA---
```

Acapol3	EFKLLYKMHTEGLLKLSLSHCA---
Ampoce_3	EFKLLYKMHMEGLLKLSLSHCA---
Sclfor_3b	EFKLLHSLHMEGVVQLNLSPCA---
Ostbic_3b	EFKLLHSLHMEX-------------
Parkin_3	EFKQLYKMHAEGLTKLSLSHCI---
Ampper3	EFKLLYKMHMEGLLKLSLSHCA---
Oncmyk_3	EFKLLYKMHTEGLIKLTLSHCA---
Gasacu_3	EFKLLYKMHTQGLLKLSLSHCA---
Labber_3	EFKLLYKMHTEGLLKLSLSHCA---
Panbuc_3	EFKFLYKMHTEGLIRLTLTPCI---
Parhas3	EFKLLFKMHTEGVLKLSLSHCT---
Cluhar_3	EFKLLFKMHTEGLLKLTLSHCA---
Aloalo_3	EFKLLFKMHTEGLLKLTLSHCA---
Panhyp_3	EFKLLFKMHTEGILKLSLSHCA---
Eleele_3	EFKLLFRMHTEGVLKLSLSHCT---
Denclu_3	EFKLLFKMHTEGLLKLSLSHCA---
Sarpil_3	EFKLLFKMHTEGLLKLTLTHCA---
Eigvir_3	EFKLLFKMHTEGVLKLSLSHCT---
Aptalb_3	EFKLLFKMHTEGVLKLSLSHCT---
Sinans_3	EFKLLFKMHADGVLKLSLSHCA---
Pygnat_3	EFKLLFKMHTEGVLKLSLSHCT---
Astmex_3	EFKLLFKMHTEGVLKLSLSHCA---
Caraur_3	EFKLLFKMHADGVLKLSLSHCA---
Danrer_3	EFKLLFKMHADGVLKLSLSHCA---
Ictpun_3	EFKLLFKMHTDGILKLSLSHCA---
Plealt_3	EFKLLYKMHTEGLLKLTLSHCA---
Plealt_9	EFKLLYKMHGEGVTRMSLSHCT---
Gasacu_9	EFKLLYKLHRDGVIKLSLTHCS---
Parkin_9	EFKLLHTLHTEGMVRLSVAPCA---
Acapol_9	EFKLLYKLHREGVIKLGLTHCS---
Larcro_9	EFKLLYKLHREGVIKLSLTHCT---
Notcor_9	EFKLLYKLHREGVIKLSVTHCS---
Latcal_9	EFKLLYKLHREGVTKLSLTHCT---
Serlaldor_9	EFKLLYKLHREGVTKLSLTHCT---
Monalb_9	EFKLLYKLYKEGVTKLSLTHCP---
Serdum_9	EFKLLYKLHREGVTKLSLTHCT---
Perflu_9	EFKLLYKLHREGVIKLSLTHCS---
Perfla_9	EFKLLYKLHREGVIKLSLTHCS---
Stepar_9	EFKLLYKLHRDGVIKLSLTHCS---
Molmol_9	EFKLLYKLHREGVIKLSLTHCA---
Hipcom_9	EFKLLYRLHRDGVTKLSLTHCT---
Takrub_9 B	EFKLLYKLHREGVIRLSLTHCT---
Takrub_9	EFKLLYKLHREGVIRLSLTHCT---
Cynsem_9	EFKLLYKLHREGVTKLSLTHCT---
Scomax_9	EFKLLYKLHREGVTKLSLTHCA---
Corlav_9	EFKLLYKMHGEGVTKLSLSHCS---
Oncmyk_9	EFKLLYKMHGEGVTKLSLSHCS---
Salsal_9	EFKLLYRMHTEGLIKLTLSHCA---
Onctsh_9	EFKLLYKMHGEGVTKLSLSHCS---
Salalp_9	EFKLLYKMHGEGVTKLSLSHCS---
Ampoce_9	EFKLLYKLHREGVIKLGLTHCS---
Ampper9	EFKLLYKLHREGVIKLGLTHCS---
Labber_9	EFKLLYKLHREGVIRLSLTHCS---
Umbpyg_3B	EFKLLYKMHTEGLIKLTLSHCA---
Esoluc_3b	EFKLLYKMHTEGLIKLTLSHCA---
Gadmor_9	EFRLLYKMHGEGVTKLSLSHCA---
Umbpyg_9	EFKLLYKMHAEGVTKLSLSHCS---
Esoluc_9	EFKLLYKMHGEGVTKLSLSHCS---
Permag_9	EFKLLYKLHREGAIKLSLTHCS---
Bolpec_9	EFKLLYKLHREGAIKLSLTHCS---
Poefor9	EFKLLYKLHREGVIKLSLTHCT---
Masarm_9	EFKLLYKLHREGVIKLSLTHCI---
Angjap_9	EFKLLYKMHTDGVIKLSLSHCA---
Poelat_9	EFKLLYKLHREGVIKLSLTHCT---
Poeret_9	EFKLLYKLHREGVIKLSLTHCT---
Xipmac_9	EFKLLYKLHREGVIKLSLTHCA---
Hapbur_9	EFKLLYKLHREGVIKLSLTHCS---
Orylat_9	EFKLLYKLHREGVTKLGLSHCT---
Gamaff_9	EFKLLYKLHREGVIKLSLTHCA---
Neobri_9	EFKLLYKLHREGVIKLSLTHCS---
Funhet_9	EFKLLYKLHREGVTKLSLTHCT---
Punnye_9	EFKLLYKLHREGVIKLSLTHCS---
Cypvar_9	EFKLLYKLHREGVTKLSLTHCT---
Krymar_9	EFKLLYKLHREGVIKLSLTHCI---
Notfur_9	EFKLLYKLHREGVTKLSLTHCS---
Notpie_9	EFKLLYKLHREGVTKLSLTRCS---
Orenil_9	EFKLLYKLHREGVIKLSLTHCS---

```
Auslim_9        EFKLLYKLHREGVTKLSLTHCT---
Anates_9        EFKLLYKLHREGVTKLSLTHCT---
Astcal_9        EFKLLYKLHREGVIKLSLTHCS---
Mayzeb_9        EFKLLYKLHREGVIKLSLTHCS---
Orymel_9        EFKLLYKLHREGVTKLGLSHCT---
Ampcit9         EFKLLYKLHREGVIKLSLTHCT---
Macfas_2        EFKALKSLHEQGALKLTVGQCD---
Macmul_2        EFKALKSLHEQGALKLTVGQCD---
Macnem_2        EFKALKSLHEQGALKLTVGQCD---
Musmus_2        EFKALKSLHEQGALKLTVGQCD---
Ponabe_2        EFKALKSLHEQGALKLTVGQCD---
Papanu_2        EFKALKSLHEQGALKLTVGQCD---
Nomleu_2        EFKALKSLHEQGALKLTVGQCD---
Pantro_2        EFKALKSLHEQGALKLTVGQCD---
Latcha_2        EFRTLYGLHQQGALKLTVGECA---
Gnapet2         EFKTLRALHRQGALCLHTEACS---
Panbuc_2b       EFQALKVLHERGALQLHTGLCH---
Ostbic_2        EFETLKALHRQGALRLHTGTCN---
Sinrhi2         EFRTLSALHRQGALRLHTGPCQ---
Hetzeb_2        EFKTLRNMHLQGALKLNVGECN---
Hipcom_2        EFRTLRALHHQGALRLHTGNCH---
Prigla_2        EFKTLRNMHLQGALKLNVGECK---
Scytor_2        EFKTLRNMHLQGALKLNVGECK---
Tetcal_2        EFKTLKNMHLRGALRLNVGECN---
Rhityp_2        EFKTLRNMHLQGALKLNIGACR---
Parkin_2b       EFRTLSRLHALGVLQLHTGSCR---
Angang_2        EFSTLRDLHSQGALRLHIGRCG---
Pygnat_2        EFRTLSMLHRKGALRLHTGTCR---
Parkin_2        EFKTLRALHRQGALCLHTEACS---
Gorgor_2        EFKALKSLHEQGALKLTVGQCD---
Thegel_2        EFKALKSLHEQGALKLTVGQCD---
Tupchi_2        EFKALKSLHEQGALKLTVGQCD---
Galgal_2        EFKALKTLHQQGALKLTVGECE---
Eleele_2        EFRTLSTLHSQGALQLHTGTCE---
Danrer_2        EFRTLSALHRQGALRLHTGPCK---
Canlupfam_2     EFKALKSLHEQGALKLTVGQCD---
Aloalo_2        EFRTLSSLHRQGALRLHTSACS---
Astmex_2        EFRTLSTLHRQGALRLHTETCR---
Sinans_2        EFRTLSALHRQGALQLHTGPCK---
Sclfor_2        EFETLRALHRQGALRLHTGACN---
Ceraty_2        EFKALKSLHEQGALKLTVGQCD---
Anocar_2        EFKALKALHQQGALKLTVGACD---
Anates_2        EFRTLSALHRQGALRLHTGQCD---
Ampbic_2        EFRTLSTLHRQGALRLHTGTCD---
Sarpil_2        EFRTLSSLHRQGALRLHTSACS---
Xenlae_2        EFKALKNLHLQGALKLNVGECE---
Cluhar_2        EFRTLSTLHKQGALRLHTSSCS---
Cypcar_2        EFRTLSALHRQGALRLHTGPCK---
Denclu_2        EFRTLRALHRQGALRLHTGTCS---
Notpie_2        EFRTLSSLHRQGALRLHTGTCD---
Erpcal2         EFQALQRLHQQGALKLNIGDCV---
Amical_2        EFKTLKNLHSQGALQLHTGPCN---
Lepocu_2        EFRALKRLHSQGALKLHTGECN---
Homsap_2        EFKALKSLHEQGALKLTVGQCD---
Bostau_2        EFKALKSLHEQGALKLTVGQCD---
Caraur_2        EFRTLSALHRQGALRLHTGPCK---
Thythy_2B       EFRTLSSLHRQGALRLHTGSCD---
Onctsh_2B       EFRTLSSLHRQGALRLHTGSCD---
Salsal_2B       EFRTLSSLHRQGALRLHTGSCD---
Oncmyk_2B       EFRTLSSLHRQGALRLHTGSCD---
Onckis_2B       EFRTLSSLHRQGALRLHTGSCD---
Salsal_2A       EFRTLSSLHRQGALRLNTGSCD---
Thythy_2A       EFRTLSSLHRQGALQLNTGSCD---
Salalp_2A       EFRTLSSLHRQGALRLNTGSCD---
Corlav_2A       EFRTLSSLHRQGALRLNTGSCD---
Cormar_2A       EFRTLSSLHRQGALRLNTGSCD---
Onctsh_2A       EFRTLSSLHRQGALRLNTGSCD---
Oncmyk_2A       EFRTLSSLHRQGALRLNTGSCD---
Neobri_2        EFRTLSTLHRQGALRLHTGVCG---
Takrub_2        EFRTLSSLHRQGALQLHTGPCD---
Tetnig_2        EFRTLSTLHRQGALRLHSGPCA---
Orenil_2        EFRTLSTLHRQGALRLHTGVCG---
Orylat_2        EFRTLSTLHRQGALRLHTGSCE---
Krymar_2        EFRTLSSLHRQGALRLHTGICH---
Notfur_2        EFRTLSSLHRQGALRLHTGTCD---
Poelat_2        EFRTLSTLHRQGALRLHTGSCD---
Gadmor_2        EFRTLNTLHRKGALRLHTGTCP---
```

```
Xipmac_2        EFRTLSTLHRQGALRLHTGSCD---
Poeret_2        EFRTLSTLHRQGALRLHTGSCD---
Astcal_2        EFRTLSTLHRQGALRLHTGVCG---
Hapbur_2        EFRTLSTLHRQGALRLHTGVCG---
Acapol_2B       EFRTLSALHRQGALRLHTGTCD---
Ampoce_2A       EFRTLSTLHRQGALRLHTGTCD---
Acapol_2A       EFRTLSALHRQGALRLHTGTCD---
Mayzeb_2        EFRTLSTLHRQGALRLHTGVCG---
Punnye_2        EFRTLSTLHRQGALRLHTGVCG---
Latcal_2        EFRTLSTLHRQGALRLHTGTCD---
Singra_2        EFRTLSALHRQGALQLHTGPCK---
Auslim_2        EFRTLSSLHRQGALRLHTGTCD---
Monalb2         EFRTLSTLHRQGALRLHTGPCD---
Ampoce_2B       EFRTLSTLHRQGALRLHTGTCD---
Chiham_2        EFRTLSALHRQGALRLHTGTCD---
Gasacu_2        EFRTLSALHRQGALRLHTGTCG---
Gymacu_2        EFRTLSALHRQGALRLHTGTCD---
Labber_2        EFRTLSALHRQGALRLHTGSCD---
Cypvar_2        EFRTLSTLHRQGALRLHTGSCD---
Funhet_2        EFRTLSTLHRQGALRLHTGHCD---
Larcro_2        EFRTLSALHRQGALQLHTGSCD---
Paroli_2        EFRTLNTLHRQGALRLHTGTCE---
Perflu_2        EFRTLSALHRQGALRLHTGTCD---
Serdum_2        EFRTLSTLHRQGALRLHTGTCD---
Stepar_2        EFRTLSTLHRQGALRLHTGTCD---
Notcor_2        EFRTLSALHRQGALRLHTGTCD---
Treber_2        EFRTLSALHRQGALRLHTGTCD---
Serlaldor_2     EFRTLSTLHRQGALRLHTGTCD---
Plaste_2        EFRTLNALHRQGALRLHTGTCD---
Clabat_4        EFKTLKMLHSKGALKLTTSKCR---
Ictpun_4A       EFKTLKMLHSKGALKLMTSKCT---
Panhyp_4        EFKTLKMLHSKGALKLTTSKCR---
Umbpyg_4        EFKTLKILHSKGALKLTTSKCT---
Esoluc_4        EFKTLKMLHSKGALKLTTSKCA---
Plealt_4        EFKTLKMLHSKGALKLTTSKCI---
Onckis_4B       EFKTLKMLHSKGALKLTTSKCE---
Onckis_4A       EFKTLKMLHSKGALKLTTSKCE---
Onctsh_4        EFKTLKMLHSKGALKLTTSKCE---
Oncmyk_4        EFKTLKMLHSKGALKLTTSKCE---
Corlav_4        EFKTLKMLHSKGALKLTTSKCE---
Astmex_4A       EFKTLKTLHSKGALKLTTSKCR---
Pygnat_4        EFKTLKMLHSKGALKLMTSTCR---
Aloalo_4        EFKTLKTLHSKGALKLTTSKCV---
Cluhar_4        EFKTLKVLHSKGALKLTTSKCM---
Denclu_4        EFKTLKALHYKGALKLTTSKCT---
Konpun4         EFKTLKTLHSKGALKLTTSKCT---
Sarpil_4        EFKTLKTLHSKGALKLTTSKCV---
Caraur_4        EFKTLKRLHSKGALKLTTSKCT---
Cteide_4        EFNTLKSLHNKGAMKLTTSKCT---
Petmar_4        EYRTLHRLHSLGALKLTTGKCT---
Cypcar_4        EFKTLKTLHSKGALKLTTSECT---
Danrer_4        EFQTLQRLHSKGALKLTTSKCT---
Misang_4        EFRTLRTLHNKGALKLTTTKCE---
Angjap_4        -------------------------
Cypcar_4A       EFKTLKTLHSKGALKLTTSECT---
Angang_4        EFKTLKTLHSRGALKLTTSKCA---
Cypcar_4B       EFKTLKTLHSKGALKLTTSKCT---
Sinans_4A       EFKTLKTLHSKGALKLTTSECT---
Sinans_4B       EFKTLKRLHSKGALKLTTSKCT---
Singra_4A       EFKTLKTLHSKGALKLTTSECT---
Aptalb_4        EFKTLKMLHGKGALKLTTSKC----
Eigvir_4        EFKTLKMLHSKGALKLTTSKCP---
Eleele_4        EFKTLKMLHSKGALKLTTSKCP---
Parhas_4        EFKTLKMLHGKGALKLTTSKCR---
Sclfor_4        EFNTLKMLHHKGALKLTTSKCT---
Gnapet4         EFKTLKFLHDRGALKLTTSKCT---
Ostbic_4        EFKTLKMLHNKGALKLTTLKCT---
Parkin_4        EFKTLKFLHERGALKLTTSKCT---
Panbuc_4        EFQTLKALHSKGALKLTTSTCT---
Erpcal4         EFKTLKMLHSKGALKLTTSKCL---
Salsal_4        EFKTLKMLHSKGALKLTTSKCE---
Saltru_4        EFKTLKMLHSKGALKLTTSKCE---
Salalp_4        EFKTLKMLHSKGALKLTTSKCE---
Salfon_4        EFKTLKMLHSKGALKLTTSKCE---
Thythy_4        EFKTLKMLHSKGALKLTTSKCE---
Calmil_4        EFKTLKSLHDRGALKLTTGKCG---
Hetzeb_4        EFKTLKSLHDRGALKLTTGKCV---
```

```
Rhityp_4       EFKTLKSLHDKGALKLTTGKCV---
Scytor_4       EFKTLKSLHDKGALKLTTGKCV---
Squaca_4       EFKTLKSLHDKGALKLTTGKCV---
Amical_4       EFKTLKTLHNKGALKLTTSTCA---
Lepocu_4       EFKTLKMLHNKGALKLTTTKCT---
Agema_4        EFKTLKMLHSKGALKLTTSKCR---
Anocar_4       EFKTLNMLHNRGALKLTTGKCA---
Canlupfam_4    EFKTLNVLHNRGALKLTTGKCV---
Crigri_4       EFKTLNVLHNRGALKLTTGKCM---
Galgal_4       EFKTLYVLHNRGALKLTTGKCV---
Mesaur_4       EFKTLNVLHNRGALKLTTGKCM---
Homsap_4       EFKTLNVLHNRGALKLTTGKCV---
Notscuscu_4    EFKTLNMLHNKGALKLTTGKCI---
Psetextex_4    EFKTLNMLHNKGALKLTTGKCI---
Musmus_4       EFKTLNVLHNRGALKLTTGKCM---
Pogvit_4       EFKTLNVLHNRGALKLTTGKCV---
Promuc_4       EFKTLNMLHNRGALKLTTGKCI---
Pantro_4       EFKTLNVLHNRGALKLTTGKCV---
Pytbiv_4       EFKTLNMLHNRGALKLTTGKCI---
Ratnor_2       EFKALKSLHEQGALKLTVGQCD---
Ratnor_4       EFKTLNVLHNRGALKLTTGKCM---
Siltro_2       EFKALKNLHLQGALKLNVGECR---
Siltro_4       EFKTLNLLHNKGALKLTTGKCI---
Susscr_4       EFKTLNVLHNRGALKLTTGKCV---
Taegut_4       EFKTLYVLHNRGALKLTTGKCI---
Termextri_4M   EFKTLHMLHNQGALKLTTGKCV---
Thasirsir_4    EFKTLNMLHNRGALKLTTGKCI---
Xenlae_4B      EFKTLNLLHNKGALKLTTGKCI---
Xenlae_4A      EFKTLNLLHNKGALKLTTGTCI---
```
