# Supplementary material for: Vertebrate Alpha2,8-Sialyltransferases (ST8Sia): A Teleost Perspective
Source: Int J Mol Sci. 2020 Jan 14;21(2):513. doi: 10.3390/ijms21020513 (PMC7014012; doi:10.3390/ijms21020513)
Supplement: Supplementary file 1 [file ijms-21-00513-s001.zip › ijms-668545-supplementary - final/Supplemental Table 1 Venuto.pdf]

Supplemental Table 1: Distribution of *st8sia* genes in mammals, squamates, sharks, agnathes, Chondrostei, Holostei and Teleosts

	ORDER	SPECIES	*st8sia2*	*st8sia4*	*st8sia3*	*st8sia9 (3-r)*	*st8sia1*	*st8sia5*	*st8sia7*	*st8sia6*	*st8sia8*
**Mammals**	Hominidae	*Homo sapiens*	1	1	1		1	1		1	
**Squamates**	Iguanidae	*Anolis carolinensis*	1	1	1		1	1	1	1	
**Sharks**	Squaliformes	*Squalus acanthias*	1	1	1		1	1		1	
	Heterodontiformes	*Heterodontus zebra*		1	1		1	1		1	
	Chimaeriformes	*Callorhinchus milii*		1	1		1	1		1	
**Agnathes**		*Lethenteron camtschaticum*		1	1		1	1	1		
		Petromyzon marinus		1			1		1	1	
		Eptatretus burgeri			1			1	1		
**Chondrostei**	Acipenseriformes	*Acipenser sinensis*	1	1	1		1	1	1		1
**Holostei**	Lepisoteiformes	*Lepisosteus oculatus*	1	1	1		1	1	A&B		1
	Amiiformes	*Amia calva*	1	1				1	1		1
**TELEOSTS**	Elopomorphes	*Anguilla anguilla*	1	1	1		-r1 -r2	-r1 -r2	A&B		1
		Anguilla japonica		1	1	1	-r1 -r2	1	1		
		Mastacembelus armatus	1		1	1					
	Osteoglossiformes	*Scleropages formosus*	1	1	1	1	1	1			1
		Paramormyrops kingsleyae	1	1	1	1	1	1			A&B
		Pantodon buchholzi		1	1		1	1			1
		Osteoglossum bicirrhosum	1	1	1	1	1	1			1
Otocephala	Clupeiformes	*Clupea harengus*	1	1	1		1	1			1
		Denticeps clupeoides	1	1	1		1	1			1
		Alosa alosa	1	1	1		1	1			1
		Sardina pilchardus	1	1	1		1	1			1
	Cypriniformes	*Danio rerio*	1	1	1		1	1	A&B		1
		Carassius auratus	1	1	1		1	1	A&B		
		Cyprinus carpio	1	-r1 -r2	1		1	1	1		1
		Sinocyclocheilus anshuiensis	1	-r1 -r2	1		1		1		A&B
	Siluriformes	*Ictularus punctatus*		1	1		1	1			
		Pangasianodon hypophthalmus		1	1		1	1			A&B
		Clarias batrachus		1							
		Tachysurus fulvidraco		1			1	1			A&B
	Gymnotiformes	*Eigenmannia virescens*		1	1		1				1
		Apteronotus albifrons		1	1		1				1
		Parapteronotus hasemani		1	1		1				1
		Electrophorus electricus	1	1	1		1	1			1

	Characiforme	*Astyanax mexicanus*	1		1	1		1	1			1
		Pygocentrus nattereri	1		1	1		1	1			1
Neoteleosteii	Perciformes	*Perca flavens*	1			1	1	1				A&B
		Perca fluviatilis	1			1	1	1	1			1
		Notothenia coriiceps	1				1	1				A&B
		Acanthochromis polyacanthus	1			1	1		1			1
		Amphiprion ocellaris	1			1	1	1	1			A&B
		Labrus bergylta	1			1	1	1	1			1
		Larimichthys crocea	1				1	1	1			1
		Lates calcarifer	1			1	1	1	1			A&B
		Monopterus albus	1			1	1	1	1			1
		Seriola dumerili	1			1	1	1				1
		Seriola lalandi dorsalis	1			1	1	1	1			1
		Stegastes partitus	1			1	1	1	1			A&B
		Gasterosteus aculeatus	1			1	1	1	1			1
	Tetraodontiformes	*Tetraodon nigroviridis*	1			1	1	1	1			1
		Takifugu rubripes	1			1	1	1	1			A&B
	Beloniformes	*Oryzias latipes*	1			1	1	1	1			1
		Oryzias melastigma	1			1	1					1
	Cichliiformes	*Haplochromis burtoni*	1			1	1	1	1			A&B
		Astatotilapia calliptera	1			1	1	1	1			A&B
		Maylandia zebra	1			1	1	1	1			1
		Neolamprologus brichardi	1			1	1	1	1			1
		Oreochromis niloticus	1			1	1	1	1			A,B&C
		Pundamilia nyererei	1			1	1	1	1			1
	Cyprinodontiformes	*Cyprinodon variegatus*	1			1	1	1	1			1
		Kryptolebias marmoratus	1			1	1	1	1			A&B
		Nothobranchius furzeri	1			1	1	1	1			1
		Xiphophorus maculatus	1			1	1	1	1			A,B,C&D
		Austrofundulus limnaeus	1			1	1	1	1			
		Fundulus heteroclitus	1				1	1	1			A,B&C
		Poecilia latipinna	1			1	1	1				1
		Poecilia reticulata	1			1	1	1	1			A&B
	Salmoniformes	*Oncorhynchus mykiss*	-r1	-r2	1	1	1	1	1	A&B		1
		Oncorhynchus kisutch	-r1	-r2	1			1	1			A&B
		Oncorhynchus tshawytscha	-r1	-r2	1		1		1	1		1
		Coregonus clupeaformis	-r1	-r2	1					1		
		Coregonus lavaretus	-r1		1	1	1	1	1	1		1
		Salmo salar	-r1	-r2	1	1	1	1	1	A&B		1
		Salmo trutta			1				1			
		Salvelinus alpinus	-r1	-r2	1		1		1	1		1
		Salvelinus fontinalis			1					1		
		Thymallus thymallus	-r1	-r2	1					A&B		
	Esociformes	*Esox lucius*			1	1	1	1	1	1		1
		Umbra pygmaea			1	1	1	1	1	1		1
