# Supplementary figures and images for: Vertebrate Alpha2,8-Sialyltransferases (ST8Sia): A Teleost Perspective
Source: Int J Mol Sci. 2020 Jan 14;21(2):513. doi: 10.3390/ijms21020513 (PMC7014012; doi:10.3390/ijms21020513)

## Poly- $\alpha$ 2,8-sialyltransferases

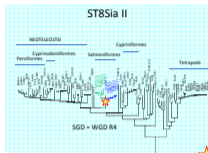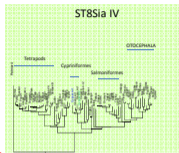

WGD R1  
552 MYA

## Oligo- $\alpha$ 2,8-sialyltransferases

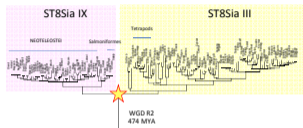

WGD R2  
474 MYA

Supplement: Supplementary file 1 [file ijms-21-00513-s001.zip › ijms-668545-supplementary - final/supplemental figure 1 Venuto.pdf]
